# Design, synthesis, and biological evaluation of multifunctional dispiro chromeno-indenoquinoxaline hybrids as dual anticancer and antibacterial agents with metal-ion sensing ability

**DOI:** 10.1039/d6ra01808d

**Published:** 2026-05-19

**Authors:** Kamalika Prusty, S. S. S. S. Sudha Ambadipudi, Suhasini Mohapatra, Tankadhara Behera, Bhabani Shankar Panda, Gopinatha Panigrahi, Sabita Nayak, Seetaram Mohapatra, Akankhya Mohanty, Jyotiprabha Rout, Chita Ranjan Sahoo, V. Lakhsma Nayak

**Affiliations:** a Organic Synthesis Laboratory, Department of Chemistry, Ravenshaw University Cuttack 753003 Odisha India sabitanayak18@gmail.com; b Applied Biology Department, CSIR- Indian Institute of Chemical Technology Hyderabad-500007 India; c ICMR-Regional Medical Research Centre, Department of Health Research, Ministry of Health & Family Welfare, Govt. of India Bhubaneswar 751023 Odisha India; d School of Chemistry, Sambalpur University, Jyoti Vihar Sambalpur 768019 Odisha India; e Siksha ‘O’ Anusandhan, Deemed to be University Kalinga Nagar, Ghatikia Bhubaneswar Odisha 751003 India

## Abstract

We have developed for the first time an aqueous-mediated 1,3-dipolar cycloaddition between indenoquinoxaline-derived nitrones and structurally diverse 2*H*-chromenes for the efficient construction of dispiro-chromeno-indenoquinoxaline frameworks. The molecular structures of the synthesized compounds were fully characterized by ^1^H and ^13^C NMR spectroscopy and high-resolution mass spectrometry, with the solid-state structure of a representative derivative unambiguously confirmed by single-crystal X-ray diffraction analysis. *In vitro* biological assays indicated that the compounds possess promising activity against both cancer cell lines and pathogenic bacterial strains. Notably, compound 21j exhibited potent cytotoxicity against MCF-7 breast cancer cells (IC_50_ = 1.59 ± 0.11 µM). In antibacterial assays, compound 21k demonstrated enhanced activity against *Escherichia coli*, whereas compound 21b showed superior efficacy against *Staphylococcus aureus*, surpassing the standard drug gentamicin. Molecular docking studies supported these results, indicating strong binding affinities of compounds 21k and 21b toward bacterial DNA gyrase, with docking scores of −9.8 and −9.0 kcal mol^−1^, respectively. Furthermore, the synthesized heterocycles displayed pronounced fluorescence responses upon coordination with Fe^2+^ and Pd^2+^ ions, highlighting their potential utility in biological and analytical sensing applications.

## Abstract 2

Aqueous 1,3-dipolar cycloaddition of indenoquinoxaline nitrones with 2*H*-chromenes affords dispiro chromeno-indenoquinoxaline hybrids with anticancer, antibacterial, and selective Fe^2+^/Pd^2+^ fluorescence sensing properties.

## Graphical abstract

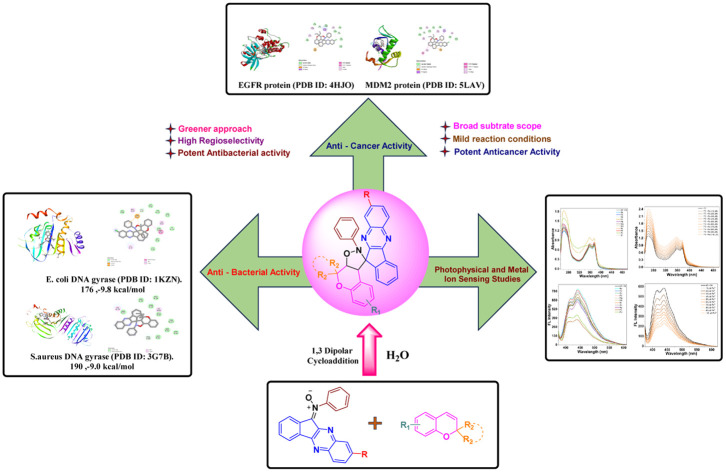

## 1. Introduction

The synthesis of structurally intriguing hybrid heterocyclic architectures has attracted sustained interest from organic and medicinal chemists due to their widespread occurrence in pharmaceutically and biologically relevant compounds.^1^ Among them, spiro-heterocycles represent a privileged class of pharmacophores in modern drug discovery, as their rigid three-dimensional architectures impart high structural complexity, conformational restriction, and well-defined spatial orientation of functional groups.^2–8^ These features often translate into enhanced selective interactions with biological macromolecules when compared to planar aromatic systems, leading to improved biological profiles.^9–12^

**Fig. 1:**
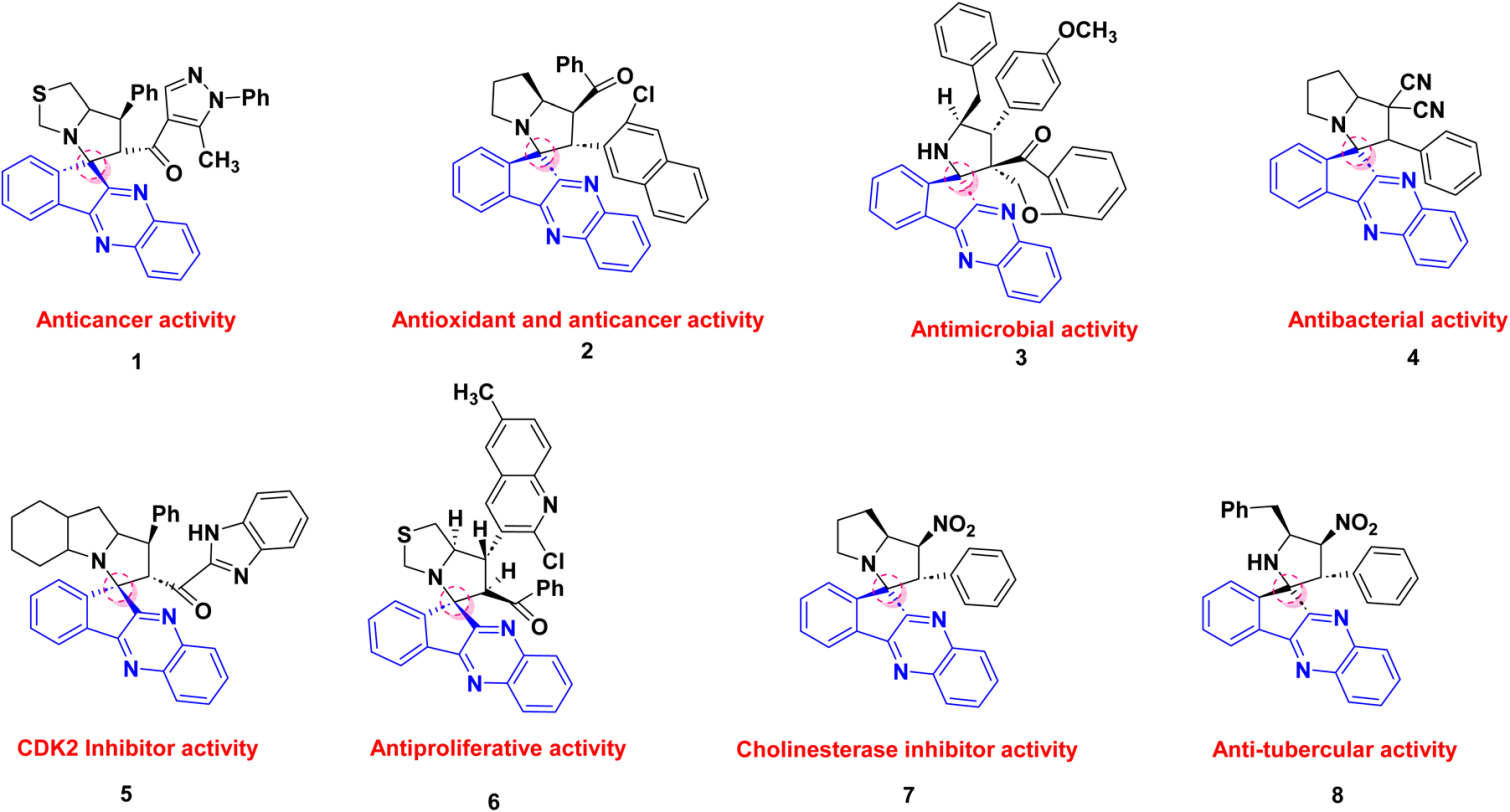
Highly pronounced biological spiro-indenoquinoxaline derivatives.

In this context, heterocyclic frameworks incorporating the indenoquinoxaline nucleus have emerged as particularly valuable scaffolds in medicinal chemistry.^13^ Indenoquinoxaline derivatives constitute an important class of fused nitrogen-containing heterocycles that have found applications in dyes and organic semiconductors and, more importantly, have demonstrated a wide spectrum of pharmacological activities, including anti-HIV, anticancer, antiproliferative, antimicrobial, and cytotoxic properties.^14–16^ The incorporation of spiro junctions into indenoquinoxaline frameworks further enhances molecular rigidity and three-dimensionality, making spiro-indenoquinoxaline hybrids especially attractive candidates for anticancer drug discovery.^17–20^ Indeed, a variety of spiro-indenoquinoxaline-based architectures such as spiro-indenoquinoxaline-pyrrolidines, spiro-indenoquinoxaline-pyrrolizidines, spiro-indenoquinoxaline-oxindoles, and dispiro-indenoquinoxaline derivatives have been reported to exhibit promising anticancer and cytotoxic activities against different human cancer cell lines. These compounds are most commonly synthesized *via* multicomponent reactions and 1,3-dipolar cycloaddition strategies, which enable rapid access to highly functionalized, stereochemically rich frameworks with potential biological relevance (Fig. 1).^1,21–24^

Consequently, there is a growing demand for innovative and efficient synthetic strategies to access such complex molecular architectures. In this direction, Jeon *et al.* reported a one-pot, regio- and diastereoselective 1,3-dipolar cycloaddition protocol for the synthesis of spiro-indenoquinoxaline pyrrolidines and their derivatives in 2022.^23^ During the same period, Hassan *et.al.* introduced a related methodology for constructing spiropyrrolidine-tethered indenoquinoxaline hybrids, which exhibited promising antimicrobial, antioxidant, and antidiabetic activities.^13^ Subsequently, Barakat *et al.* described the combinatorial stereoselective synthesis of rationally designed spiroindeno[1,2-*b*] quinoxaline-based CDK2 inhibitors for non-small cell lung cancer therapy, achieved through a cost-effective one-pot multicomponent 1,3-dipolar cycloaddition approach.^24^ More recently, Kanchrana *et al.* developed an ultrasound-assisted [3 + 2] cycloaddition protocol for the synthesis of spiroquinoxaline-1,2,4-oxadiazoles under mild conditions, eliminating the need for conventional heating and column chromatography while delivering high yields within short reaction times.^25^

Despite these significant advances, the synthesis of dihydrospiro[chromeno-isoxazole-indenoquinoxaline] derivatives has not yet been reported, to the best of our knowledge. Although chromene-based scaffolds are well recognized as privileged pharmacophores exhibiting a wide range of biological activities, including antioxidant, anticancer, antibacterial, and antidiabetic properties, their molecular hybridization with the indenoquinoxaline nucleus within a dispiro framework remains less unexplored. Consequently, the strategic integration of these two biologically significant heterocyclic systems into a single rigid architecture represents an appealing yet underdeveloped approach for the construction of novel functional molecules.

In this context, the present study reports the design and synthesis of a novel series of previously unreported indenoquinoxaline-based nitrones and their subsequent integration with 2*H*-chromene units to construct new dispiro-chromeno-indenoquinoxaline hybrids. The synthesized compounds were further subjected to biological evaluation, along with detailed photophysical investigations and metal-ion sensing studies.

## 2. Result and discussion

### 2.1. Chemistry

#### 2.1.1. Synthesis

In order to develop an efficient protocol for the construction of the targeted dispiro frameworks, indenoquinoxaline-derived nitrones 15(a–c) and 2*H*-chromene derivatives 20(a–f) were first prepared as key reaction components. The synthesis of indenoquinoxaline-based nitrones 15(a–c) was prepared through a sequential condensation strategy. Initially, ninhydrin 9 was reacted with substituted *o*-phenylenediamine 10(a–c) in ethanol at room temperature to afford the substituted indenoquinoxaline scaffold 11(a–c). This intermediate was then subjected to condensation with *p*-toluidine 12 in an ethanol–glacial acetic acid (2 : 1) mixture to generate the corresponding imine derivatives 13(a–c). Subsequent treatment of these imines 13(a–c) with *N*-phenyl hydroxylamine 14 in chloroform furnished the desired indenoquinoxaline nitrones 15(a–c) in excellent yields (Scheme 2).^26^

In parallel, the 2*H*-chromene derivatives 20(a–f) were synthesized following reported literature procedures. Briefly, substituted *o*-hydroxy acetophenones 16(a–b) were subjected to aldol condensation with appropriately substituted ketones 17(a–d), and the resulting adducts underwent intramolecular cyclization to furnish chromone derivatives 18(a–f) which upon subsequent reduction with sodium borohydride formed chroman-4-ol intermediates 19(a–f) and followed by *p*-toluene sulfonic acid (*p*-TsOH) mediated dehydration in THF, smoothly afforded the desired *2H*-chromene derivatives 20(a–f) (Scheme 3).^27–30^

Once the indenoquinoxaline-based nitrones and *2H*-chromene derivatives were synthesized, a systematic investigation of their reactivity toward 1,3 dipolar cycloaddition was undertaken. Key reaction parameters, including solvent, temperature, and reaction time, were carefully evaluated in order to determine the feasibility of the transformation and to identify optimal conditions. The optimization results are compiled in Table 1.

**Table 1:** Optimization of the 1,3-dipolar cycloaddition reaction conditions for the synthesis of dispiro-chromeno-indenoquinoxaline derivatives

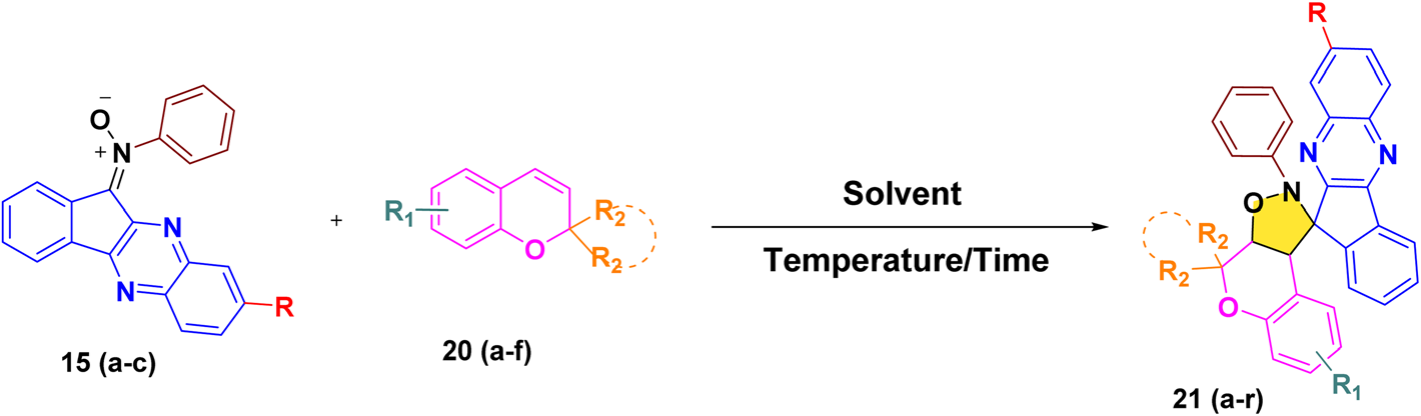				
Entry	Solvent^a^	Temp. (°C)	Time (h)	Yield^b^ (%)
01	EtOH	80	24	10
02	MeOH	70	24	12
03	DCM	40	24	15
04	Acetonitrile	100	12	55
05	DMSO	100	12	35
06	DMF	100	12	39
07	THF	70	12	48
08	H_2_O	100	5	89
09	H_2_O	80	5	81
10	H_2_O	80	8	86
11	Toluene	120	8	82
12	*o*-Xylene	140	7	84

a Reaction condition: indenoquinoxaline nitrone (1.0 mmol), 2*H*-chromene (2 mmol), solvent (2–3 mL), temperature.

b Isolated yields after purification.

Initially, commonly reported solvents in the literature for spiro-indenoquinoxaline synthesis, including protic solvents such as ethanol and methanol, were evaluated under heating conditions. However, both media exhibited poor reactivity, affording only trace to low conversions even after 24 h, and resulted in low isolated yields of 10–12% (entries 1 and 2).^13,23,24^ Subsequently, the reaction was examined in polar aprotic solvents. Dichloromethane at 40 °C furnished only 15% yield after prolonged heating (entry 3). A moderate improvement was observed in acetonitrile at 100 °C, where the desired cycloadduct was obtained in 55% yield within 12 h (entry 4). Nevertheless, other polar aprotic solvents such as DMSO, DMF, and THF provided only moderate to low yields (35–48%), indicating that these solvents are not optimal for this transformation (entries 5–7).

Considering the potential benefits of green and sustainable media, water was then investigated. Gratifyingly, performing the reaction in water at 100 °C significantly enhanced the efficiency, delivering the target dispiro-chromeno-indenoquinoxaline in an excellent 89% yield within only 5 h (entry 8). Reducing the temperature to 80 °C slightly decreased the yield (81%), which could be partially recovered by extending the reaction time to 8 h (86%) (entries 9 and 10), demonstrating the crucial role of thermal activation in aqueous medium. For comparison, non-polar solvents such as toluene and *o*-xylene also afforded good yields (82–84%, entries 11 and 12). However, these conditions required higher temperatures and longer reaction times, making them less favorable from both environmental and practical perspectives.

Overall, water at 100 °C for 5 h was identified as the optimal reaction condition. Upon successful optimisation of reaction conditions, compound 21a was subsequently purified and fully characterized using ^1^H NMR, ^13^C NMR, and HRMS analyses.

#### 2.1.2. Structural elucidation

**Fig. 2:**
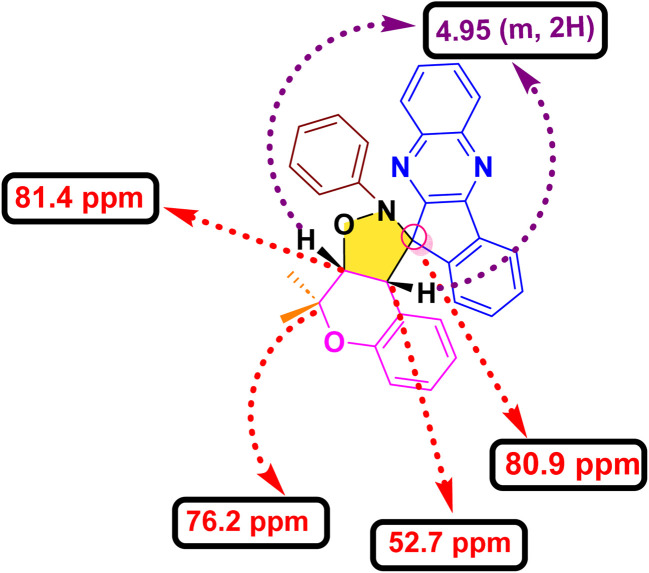
^1^H NMR (deep purple) and ^13^C NMR (red) chemical shifts (ppm): spectral data analysis of compound 21a.

The structure of the newly synthesized dispiro-chromeno-indenoquinoxaline derivative 21a was unambiguously established by detailed spectroscopic analysis, particularly ^1^H and ^13^C NMR spectroscopy and HRMS analysis. In the ^1^H NMR spectrum, the newly formed isoxazolidine ring was clearly evidenced by the appearance of a characteristic multiplet(m) at *δ* 4.95 ppm integrating for two protons, which can be assigned to the diastereotopic methylene protons adjacent to the N–O moiety. The aromatic region displayed a set of well-resolved signals corresponding to the chromene, indenoquinoxaline, and phenyl rings, confirming the successful assembly of the dispiro-chromeno-indenoquinoxaline derivative. The ^13^C NMR spectrum further supported the proposed structure. Two downfield signals at *δ* 80.9 and 76.2 ppm were attributed to the quaternary spiro carbon and quaternary alkyl carbon confirming the presence of the dispiro junctions. Another signals resonance at *δ* 52.7 ppm and 81.4 ppm were assigned to the saturated carbons of the newly formed isoxazolidine ring, consistent with cycloaddition of the nitrone across the *2H*-chromene double bond. The remaining aromatic carbons signals appeared in their expected regions. In the HRMS spectrum, the experimental molecular ion peak appeared at 484.2025 [M + H]^+^,which is equivalent to the calculated ion peak at 484.2018 which conclusively confirm the formation of the targeted dispiro-chromeno-indenoquinoxaline framework 21a*via* regiospecific 1,3-dipolar cycloaddition (Fig. 2).

#### 2.1.3. Substrate scope analysis

**Scheme 1:**
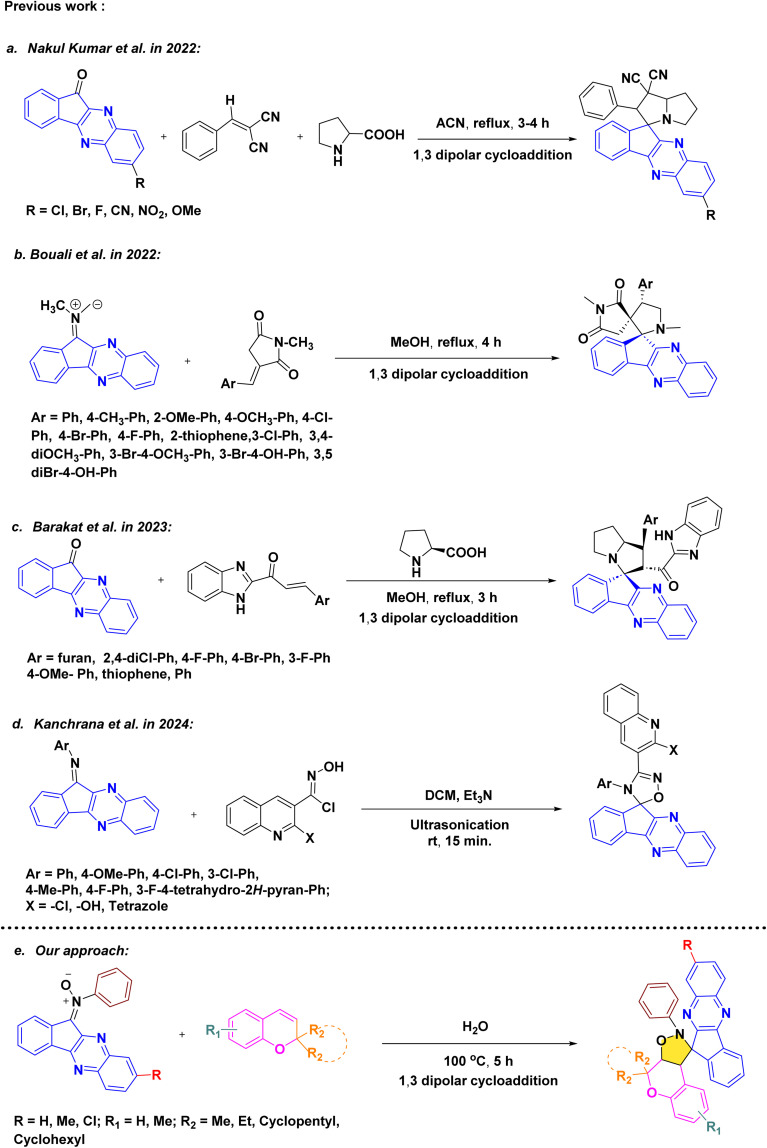
Synthesis of dihydrospiro[chromeno-isoxazole-indenoquinoxaline] derivatives.

After, establishing the appropriate reaction conditions (Table 1, entry 8) and spectroscopic characterisation of compound 21a, we turned our attention to evaluating the scope and versatility of this aqueous-mediated 1,3 dipolar cycloaddition by examining a diverse set of indenoquinoxaline nitrones and 2*H*-chromene derivatives (Scheme 1). We first explored the influence of substituents on the 2-position of the chromene framework. A 2-methyl substituent underwent smooth cycloaddition to furnish the corresponding dispiro adduct in excellent yield (89%). Increasing the chain length to ethyl slightly diminished the yield (83%). In contrast, chromenes bearing conformationally restricted cyclic substituents exhibited enhanced reactivity. Both cyclopentyl- and cyclohexyl-substituted substrates delivered the desired dispiro frameworks in high yields, with the cyclohexyl derivative giving particularly favorable results. In addition, substitution on the aromatic ring of the chromene was well tolerated. Introduction of a methyl group on the benzene ring consistently provided high yields, indicating that electron-donating substitution on the chromene scaffold facilitates the cycloaddition process (Schemes 4–7).

**Scheme 2:**
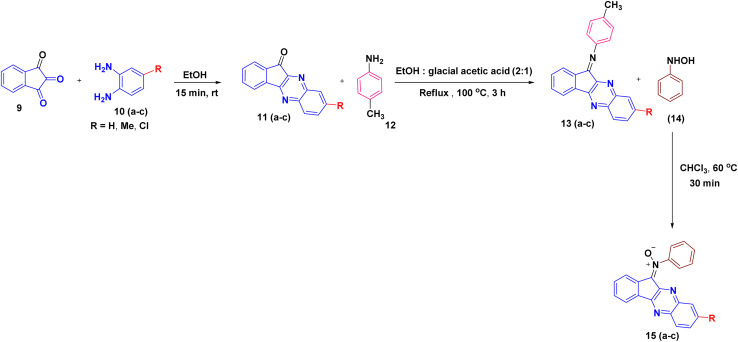
Synthesis of *N*-phenyl indenoquinoxaline nitrones 15(a–c).

**Scheme 3:**
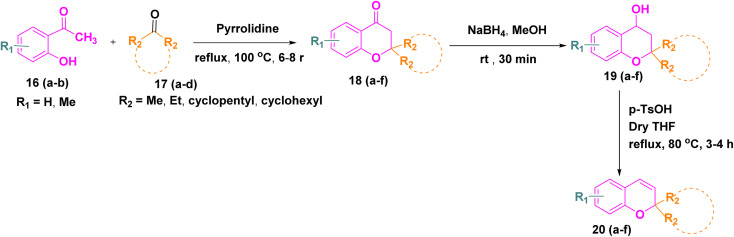
Synthesis of *2H*-chromene derivatives 20(a–f).

**Scheme 4:**
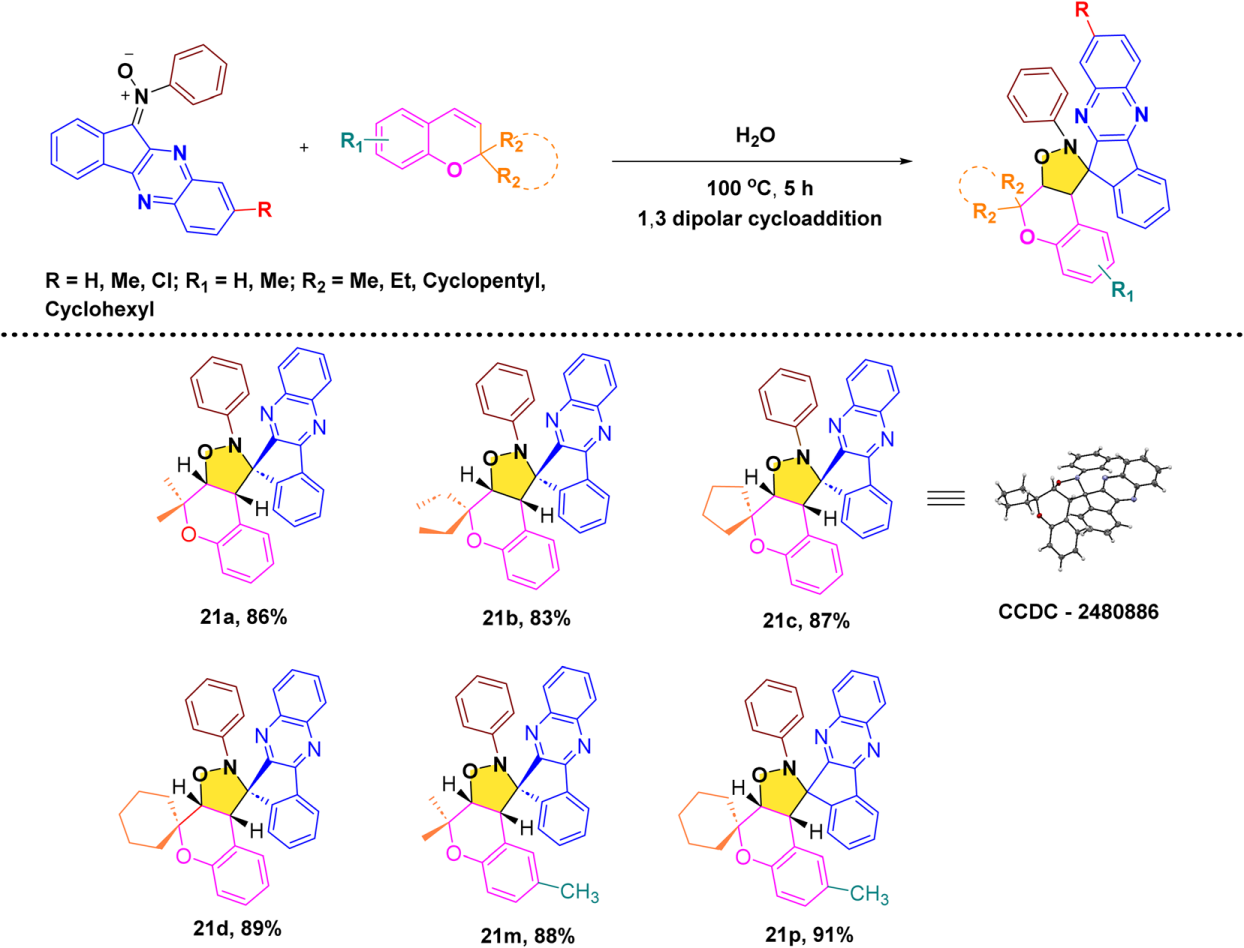
Synthesis of dispiro-chromeno-indenoquinoxaline derivatives.

**Scheme 5:**
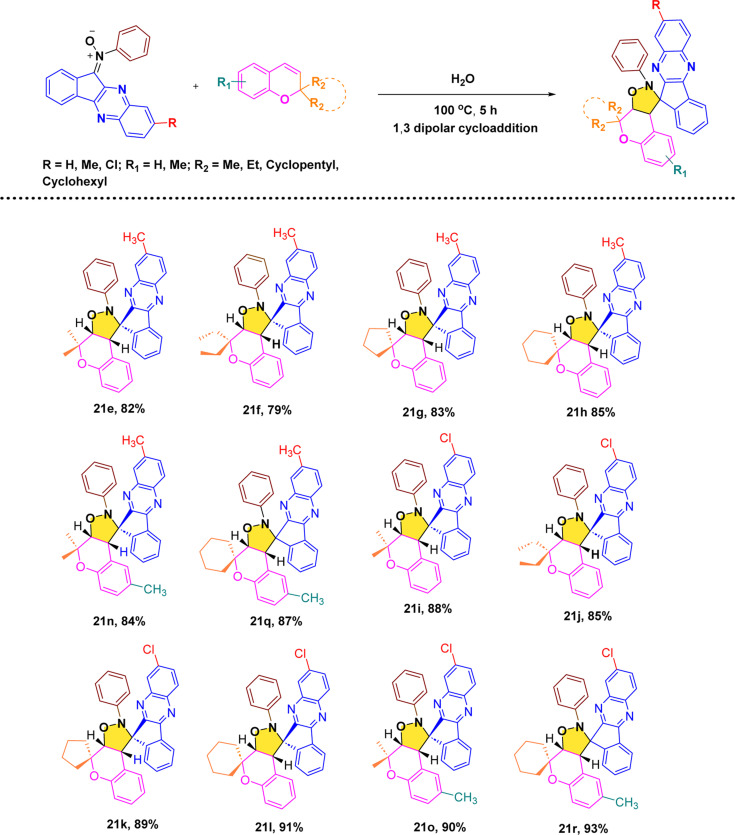
Reaction of indenoquinoxaline based nitrones with *2H*-chromenes.

**Scheme 6:**
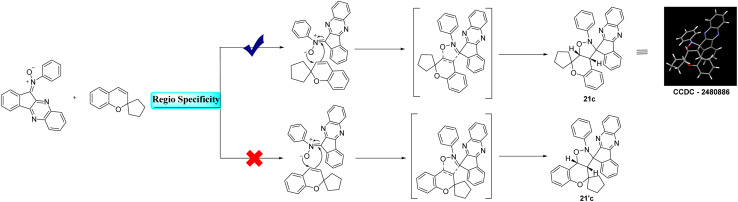
Proposed 1,3 dipolar cycloaddition mechanism.

**Scheme 7:**
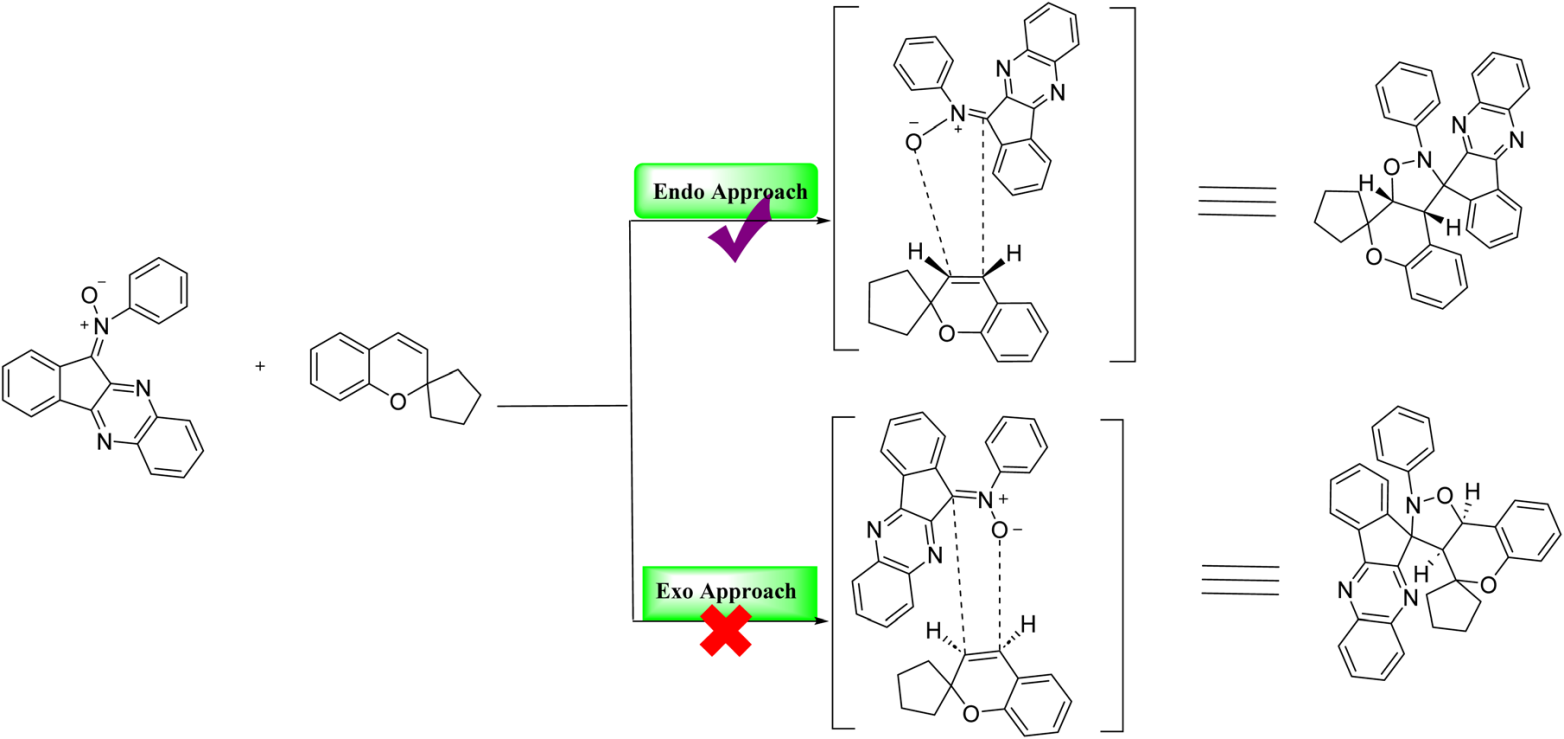
Proposed endo approach mechanism.

We then further extend the scope of this reaction by using different substituted indenoquinoxaline framework with a series of different substituted 2*H*-chromenes. Both electron-donating and electron-withdrawing substituents on the indenoquinoxaline framework were examined to evaluate their electronic influence on the cycloaddition. It was observed that indenoquinoxaline-derived nitrones bearing electron-donating methyl groups generally afforded the corresponding dispiro products in slightly reduced yields compared to the unsubstituted system. By comparison, an electron-withdrawing chloro substituent on the indenoquinoxaline core led to improved reaction efficiencies, consistently providing the cycloadducts in higher yields. These results suggest that electron-deficient indenoquinoxaline-derived nitrones favor the cycloaddition process, likely by enhancing the electrophilic character of the dipole and facilitating interaction with the chromene dipolarophile.

#### 2.1.4. X-ray crystallographic analysis

**Fig. 3:**
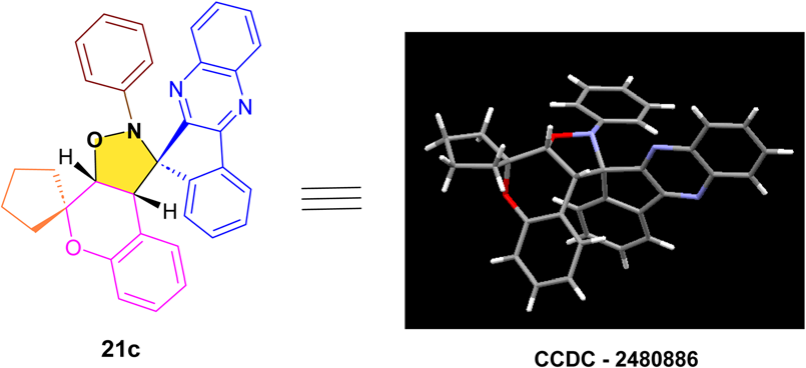
Single crystal X-ray representation of compound 21c.

To establish the relative configuration of the synthesized compounds 21(a–r), single-crystal X-ray diffraction analysis was performed on compound 21c. Suitable crystals were obtained by slow evaporation from an CDCl_3_/hexane solvent system. The solid-state structure of 21c (Fig. 3) clearly confirms the proposed dispiro-chromeno-indenoquinoxaline framework and establishes the relative configuration of the newly formed stereocenters.

#### 2.1.5. Regiospecificity and endo selective approach

The reaction between indenoquinoxaline based nitrone and 2*H*-chromene proceeds *via* a 1,3 dipolar cycloaddition pathway. Theoretically this reaction can yield two regioisomeric chromene fused spiro isoxazolidine derivatives 21c and 21′c. However the reaction exhibited complete regiospecificity affording exclusively product 21c. Wherein the oxygen centered site of the nitrone moiety attacks the carbon of the chromene double bond adjacent to the spiro center, while the carbon – centered site of the nitrone simultaneously attacks the other carbon of the chromene double bond, leading to the observed regiospecific cycloaddition product. Structural elucidation by single-crystal X-ray diffraction analysis confirmed that the cycloaddition occurs through a preferred endo approach, resulting in an endo specific cycloaddition. The proposed 1,3 dipolar cycloaddition mechanism and the endo approach are illustrated below.

## 3. Biological studies

### 3.1. Anticancer assessment

#### 3.1.1. Cytotoxicity MTT assay

All synthesized compounds were evaluated for their *in vitro* anticancer activity against three human breast cancer cell lines, namely MDA-MB-468, MDA-MB-231, and MCF-7, using the MTT assay.^31^ The obtained IC_50_ values are summarized in Table 2. In addition, cytotoxicity toward normal human embryonic kidney cells (HEK-293) was also examined. Overall, several compounds displayed moderate to good growth inhibition against the tested cancer cell lines, while most of them showed minimal toxicity toward normal HEK-293 cells. Among the series, 21j emerged as the most potent compound, exhibiting excellent anticancer activity with IC_50_ values of 3.56 ± 0.29 µM (MDA-MB-468), 5.91 ± 0.61 µM (MDA-MB-231), and 1.59 ± 0.11 µM (MCF-7), comparable to the standard drug doxorubicin. Importantly 21j showed low toxicity toward HEK-293 cells, indicating good selectivity. Compounds 21r, 21o, 21n, 21i, 21l, and 21f also demonstrated significant cytotoxicity, particularly against the MCF-7 cell line, with IC_50_ values below 10 µM. Several other derivatives showed moderate or weak activity, while compounds such as 21g and 21h were essentially inactive (IC_50_ > 100 µM).

**Table 2:** IC_50_ values^a^ (in µM) for compounds 21(a–r)

Compound	MDA MB-468^b^	MDA MB-231^c^	MCF-7^d^	HEK-293^e^
21a	22.29 ± 1.22	32.49 ± 0.98	25.72 ± 0.70	>100
21b	19.52 ± 1.35	35.61 ± 0.55	12.89 ± 0.80	>100
21c	38.73 ± 0.39	40.22 ± 2.13	30.94 ± 1.39	>100
21d	45.39 ± 2.21	46.21 ± 0.74	38.34 ± 1.99	84.50 ± 1.14
21e	>100	48.68 ± 2.52	28.71 ± 0.65	>100
21f	11.69 ± 1.04	23.31 ± 0.60	8.78 ± 0.23	95.38 ± 3.86
21g	>100	>100	>100	>100
21h	>100	>100	>100	>100
21i	45.50 ± 0.98	25.91 ± 0.54	6.08 ± 0.46	94.02 ± 3.89
21j	**3.56 ± 0.29**	**5.91 ± 0.61**	**1.59 ± 0.11**	**93.04 ± 2.07**
21k	34.30 ± 1.98	27.66 ± 0.51	32.97 ± 4.41	>100
21l	9.08 ± 0.85	25.62 ± 0.55	6.92 ± 0.16	80.62 ± 3.05
21m	>100	46.45 ± 2.74	24.36 ± 0.68	>100
21n	28.75 ± 0.47	19.06 ± 0.88	3.69 ± 0.26	77.63 ± 3.19
21o	13.80 ± 0.32	29.93 ± 1.0	3.56 ± 0.37	84.70 ± 0.78
21p	62.28 ± 0.99	44.23 ± 0.68	22.29 ± 0.31	78.80 ± 0.98
21q	13.81 ± 0.64	31.72 ± 2.20	12.30 ± 0.28	>100
21r	4.52 ± 0.52	8.32 ± 0.34	2.69 ± 0.06	83.72 ± 3.27
Doxorubicin	1.67 ± 0.08	1.59 ± 0.13	1.63 ± 0.34	NT

a 50% inhibitory concentration (IC_50_) after 48 h of drug treatment.

b Breast cancer cells (Estrogen receptor-negative (ER−)).

c Breast cancer cells (Estrogen receptor-negative (ER−)).

d Breast cancer cells (Estrogen receptor-positive (ER+)).

e Normal human embryonic kidney 293 cells NT: not tested.

#### 3.1.2. Molecular docking studies against anticancer target proteins

**Fig. 4:**
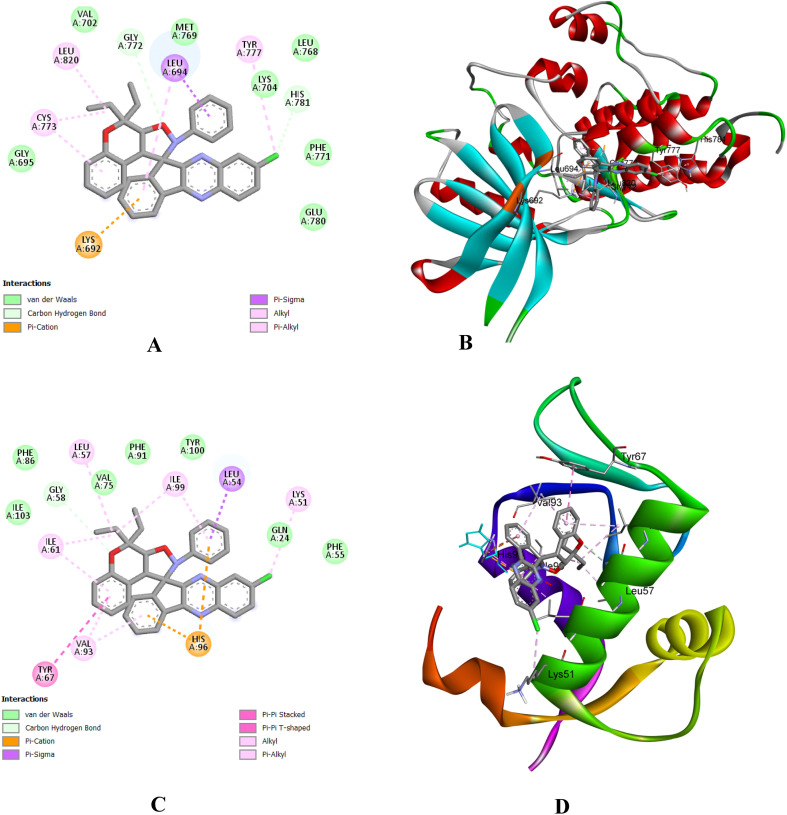
The 2D and 3D docking illustrations of compound 21j in the active sites of (A and B): EGFR protein (PDB ID: 4HJO) and (C and D): MDM2 protein (PDB ID: 5LAV).

The molecular docking outcomes for the synthesized dihydrospiro[chromeno-isoxazole-indenoquinoxaline] derivatives 21(a–r) against the active sites of epidermal growth factor receptor (EGFR) tyrosine kinase protein (PDB ID: 4HJO) and E3 ubiquitin ligase tumor suppressor p53 regulator (MDM2) protein (PDB ID: 5LAV) targets demonstrated good docking scores ranging from −7.47 to −10.24 kcal mol^−1^ and −10.26 to −12.05 kcal mol^−1^, compared to the standard drug Doxorubicin with docking scores of −8.49 kcal mol^−1^ and −8.87 kcal mol^−1^ respectively (Table S1) [SI]. The docking illustrations highlighted that all compounds commonly showed various types of protein–ligand interactions, including conventional-H bonds, C–H bonds, van der Waals, alkyl, π–σ, π–cation, π–anion, π–alkyl, π–π stacked, π–π T-shaped, *etc.*, of different categories (hydrogen bond, hydrophobic and electrostatic) (Table S1) [SI]. Among all the docked compounds, compound 21j exhibited the highest docking scores of −10.24 kcal mol^−1^ and −12.05 kcal mol^−1^ against the active pockets of EGFR and MDM2 proteins respectively (Table 3). In the active site of EGFR protein compound 21j established a single conventional hydrogen bond interaction (with GLN24), two alkyl interactions (with MET62 and VAL93), four π–alkyl interactions (with ILE61, LEU57, PHE86 and ILE103), two π–σ interactions (with LEU54 and ILE99), one π–cation interaction (with HIS96) and a π–π T-shaped interaction with PHE91 amino acid residue (Fig. 4). Similarly, in the active site of MDM2 protein compound 21j established a conventional hydrogen bond interaction (with GLN24), six alkyl interactions (with VAL75, VAL93, ILE61, ILE99, LEU57 and LEU54), three π–alkyl interactions (PHE86, PHE91 and TYR67), a π–σ interaction (with LEU54) and a π–cation interaction with HIS96 amino acid residue (Fig. 4). 2D and 3D molecular docking visualisations of investigated compounds 21j and Doxorubicin in the active sites of EGFR protein (PDB ID: 4HJO) and MDM2 protein (PDB ID: 5LAV) are as displayed in Fig. 4.

**Table 3:** Molecular docking analysis of most potent compound 21j and standard drug Doxorubicin as ligand with EGFR (PDB ID: 4HJO) and MDM2 (PDB ID: 5LAV) target proteins

Ligands	EGFR (PDB ID: 4HJO)		MDM2 (PDB ID: 5LAV)	
	Docking score (kcal mol^−1^)	Molecular interaction type with amino acid residues	Docking score (kcal mol^−1^)	Molecular interaction type with amino acid residues
21j	−10.24	Hydrogen bond interactions: CYS773 (conventional–H bond), GLY772 (C–H bond); hydrophobic interactions: VAL702, MET769, LEU768, LEU820 (alkyl), LEU694 (π–σ), PHE771, TYR777 (π–alkyl); electrostatic interactions: LYS692 (π–cation)	−12.05	Hydrogen bond interactions: GLN24 (conventional–H bond); hydrophobic interactions: VAL75, VAL93, ILE61, ILE99, LEU57, LEU54 (alkyl), PHE86, PHE91, TYR67 (π–alkyl), LEU54 (π–σ); electrostatic interactions: (HIS96) π–cation
Doxorubicin	−8.49	Hydrogen bond interactions: LYS721, ASN 818, CYS773 (conventional–H bond), GLY697 (C–H bond); hydrophobic interactions: VAL702, LEU820 (π–σ), ALA719 (π–alkyl)	−8.87	Hydrogen bond interactions: HIS 96 (conventional–H bond); hydrophobic interactions: LEU57, LEU54 (alkyl), ILE99, GLN24 (π–alkyl), PHE55 (π–π T-shaped)

#### 3.1.3. Anticancer structure–activity relationships (SARs)

**Fig. 5:**
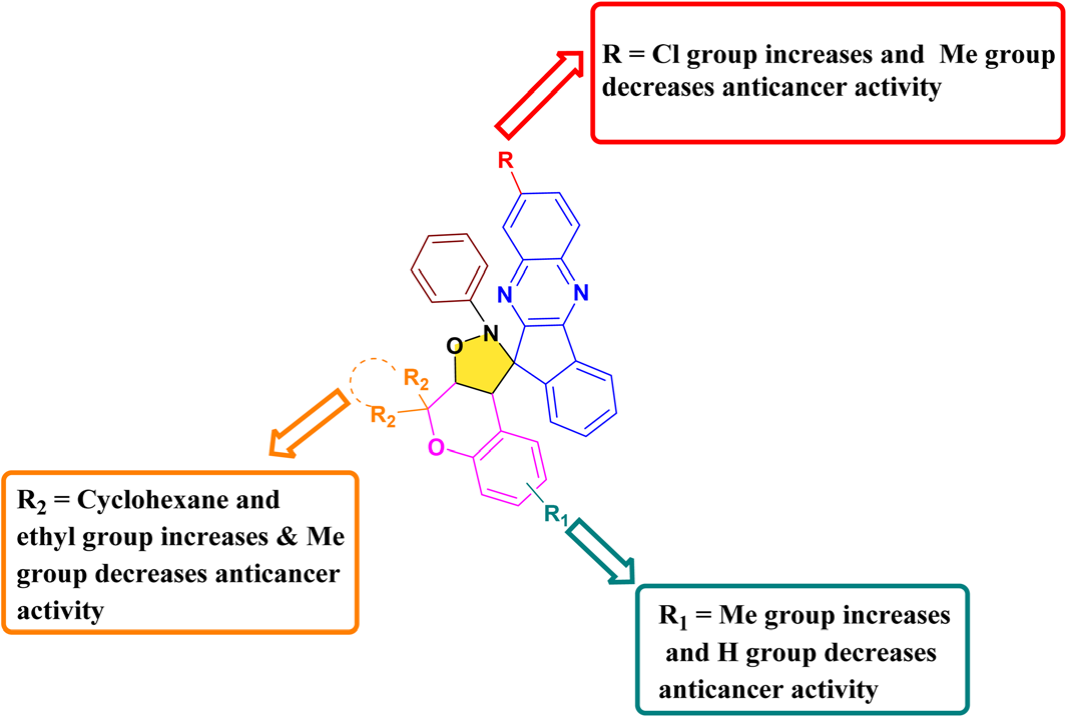
Pictorial representation of the SAR of the compound 21(a–r).

The anticancer structure–activity relationship was interpreted by correlating the *in vitro* cytotoxicity results against the MCF-7 cell line with *in silico* molecular docking analysis of the synthesized hybrids. The results clearly indicate that variations in substitution on both the chromene and indenoquinoxaline frameworks significantly influence cytotoxic potency. Among the series, compound 21j emerged as the most active derivative, where the presence of two ethyl groups at the R_2_ position of the chromene scaffold along with a chloro (–Cl) substituent on the indenoquinoxaline ring led to better anticancer activity (IC_50_ = 1.59 ± 0.11 µM), supported by strong binding interactions with the target anticancer protein. A derivative incorporating a cyclohexane ring at the R_2_ position, a methyl group at R_1_, and a chloro substituent on the indenoquinoxaline core also demonstrated good activity (IC_50_ = 2.69 ± 0.06 µM). In contrast, compounds bearing a cyclohexane ring at R_2_ without substitution on the indenoquinoxaline, and compounds containing methyl substituted indenoquinoxaline with two methyl group at R_2_ position exhibited comparatively weak anticancer activity. Overall, these findings emphasize that both electronic effects and steric contributions of substituents on the two pharmacophoric units play a decisive role in modulating anticancer efficacy (Fig. 5).

### 3.2. Antibacterial assessment

**Fig. 6:**
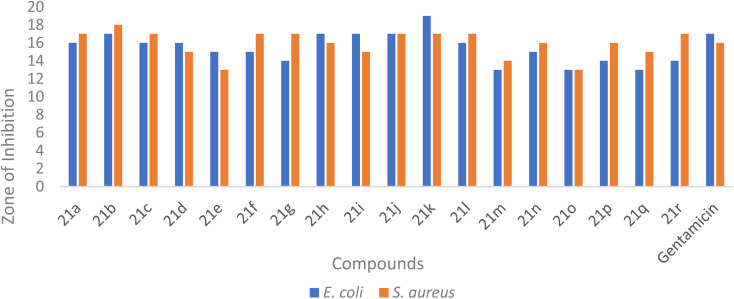
Graphical representation of the *in vitro* antimicrobial (ZI) assay of the synthesized dihydrospiro[chromeno-isoxazole-indenoquinoxaline]derivatives 21(a–r) against *E. coli* and *S. aureus*.

**Fig. 7:**
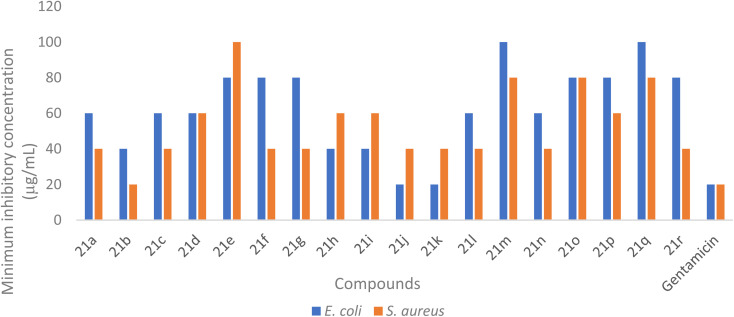
Graphical demonstration of the *in vitro* antimicrobial (MIC) assay of the synthesized dihydrospiro[chromeno-isoxazole-indenoquinoxaline]derivatives 21(a–r) against *E. coli* and *S. aureus*.

A novel series of dihydrospiro[chromeno-isoxazole-indenoquinoxaline] derivatives 21(a–r) was thoroughly examined for their *in vitro* antibacterial activity against two clinically important bacterial strains. The synthesized compounds were evaluated against *Escherichia coli* (Gram-negative) and *Staphylococcus aureus* (Gram-positive) using the agar well diffusion technique, with Gentamicin employed as the reference standard. In this study, the antibacterial performance of the tested compounds were determined in DMSO at a concentration of 20 µg mL^−1^. Among the tested derivatives, compound 21k exhibited the most pronounced inhibitory effect against *E. coli*, showing a 19 mm zone of inhibition and a MIC value of 20 µg mL^−1^ (Table 4, Fig. 6 and 7). Conversely, compound 21b showed a zone of inhibition of 18 mm against *S. aureus*, also with a MIC value of 20 µg mL^−1^. The zone of inhibition (ZI) and minimum inhibitory concentration (MIC) values were determined to assess antibacterial potency, and the results confirmed that most derivatives exhibited moderate to good inhibitory activity against both bacterial strains, as summarized in Table 4.

**Table 4:** Determination of zones of inhibition (ZI) and minimum inhibitory concentration (MIC) of compounds against *E. coli* and *S. aureus*

Compounds	*E. coli*		*S. aureus*	
	ZI (mm)	MIC (µg mL^−1^)	ZI (mm)	MIC (µg mL^−1^)
21a	16	60	17	40
21b	17	40	**18**	**20**
21c	16	60	17	40
21d	16	60	15	60
21e	15	80	13	100
21f	15	80	17	40
21g	14	80	17	40
21h	17	40	16	60
21i	17	40	15	60
21j	17	20	17	40
21k	**19**	**20**	17	40
21l	16	60	17	40
21m	13	100	14	80
21n	15	60	16	40
21o	13	80	13	80
21p	14	80	16	60
21q	13	100	15	80
21r	14	80	17	40
Gentamicin	17	20	16	20

Among the tested molecules, compound 21k and compound 21b demonstrated the highest antibacterial efficacy, exhibiting ZI values of 19 mm and 18 mm against *E. coli* and *S. aureus* respectively. Furthermore, MIC determinations through cell viability assays revealed potent inhibition, with both compounds displaying MIC values of 20 µg mL^−1^ toward *E. coli* and *S. aureus*. In contrast, compound 21e and compound 21q exhibited relatively weak antibacterial activity, with ZI values of 13 mm and MIC values of 100 µg mL^−1^ against both bacterial strains. The comparative ZI and MIC values of all synthesized compounds are illustrated graphically in Fig. 6 and 7, respectively.

#### 3.2.1. Molecular docking investigation

**Fig. 8:**
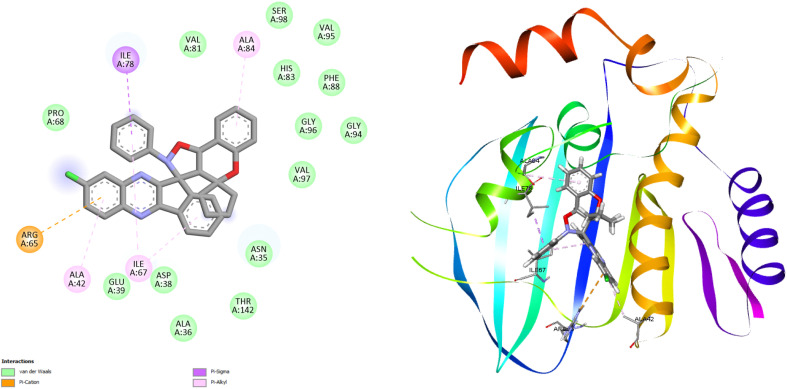
2D binding interaction and 3D binding image of compound 21k with *E. coli* DNA gyrase (PDB ID: 1KZN).

**Fig. 9:**
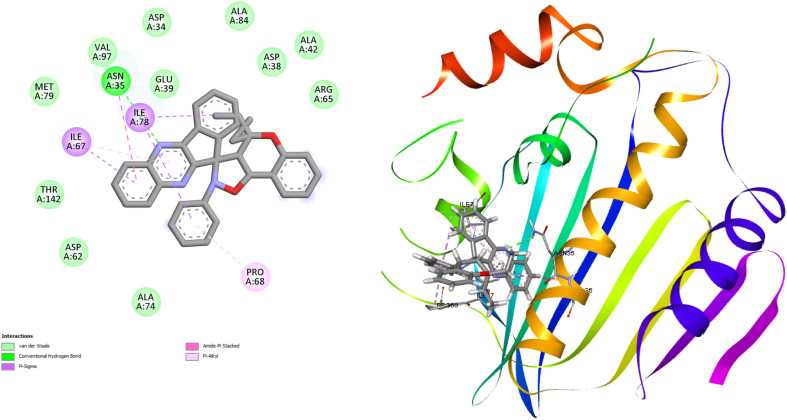
2D binding interaction and 3D binding image of compound 21b with *S. aureus* DNA gyrase (PDB ID: 3 G7B).

Molecular docking investigations of the newly synthesized of dihydrospiro[chromeno-isoxazole-indenoquinoxaline] derivatives 21(a–r) were performed to identify the binding interaction with the DNA gyrase inhibitor. The Gram-negative bacterial strain *E. coli* and Gram-positive bacterial strain *S. aureus* are two human pathogenic bacterial strains of the DNA gyrase inhibitor used for molecular modelling. The crystal structures of *E. coli* (PDB ID: 1KZN) and *S. aureus* (PDB ID: 3G7B) were retrieved from RCSB protein bank in (.pdb) format. After retrieving the heteroatoms, water molecules were removed by using PyMOL. AutoDock was used for the determination of binding interactions in molecular docking studies, and BIOVIA Discovery Studio v2025 was used for visualizing intermolecular binding interactions of docked complexes and to generate the 2D and 3D structures of the docked complexes of the tested compounds. In the present study, eighteen compounds were used for docking studies, and the docking scores ranged from −7.0 to −9.8 kcal mol^−1^ for *E. coli* and from −8.2 to −9.0 kcal mol^−1^ for *S. aureus*. The results demonstrated that compound 21k showed better binding efficacy, with a docking score of −9.8 kcal mol^−1^, against *E. coli* DNA gyrase. Compound 21b exhibited good binding affinity, with a docking energy of −9.0 kcal mol^−1^, against *S. aureus* DNA gyrase. The binding interactions between bacterial DNA gyrase and the most potent compound 21k and 21b include van der Waals force, conventional hydrogen bond, carbon–hydrogen bond, alkyl, Pi–alkyl, Pi–anion, Pi–cation, and Pi–sigma as illustrated in Table 5 and the remaining are illustrated in Table S2 [SI]. The 2D binding interaction and 3D binding image of the most potent compounds 21k and 21b against *E. coli* DNA gyrase and *S. aureus* DNA gyrase are represented in Fig. 8 and 9 respectively.

**Table 5:** Molecular docking studies of bacterial DNA gyrase with compound 21b & 21k

Compounds	*E. coli* DNA gyrase (PDBID-1KZN)		*S. aureus* DNA gyrase (PDBID-3 G7B)	
	Score (kcal mol^−1^)	Binding interactions	Score (kcal mol^−1^)	Binding interactions
21k	−9.8	Hydrophobic contacts: PRO68, ILE78(Pi-sigma), VAL81, ALA84, SER98, VAL95, HIS83, PHE88, GLY96, GLY94, VAL97, ASN35, THR142, ALA36, ASP38, ILE67 (Pi–alkyl), GLU39, ALA42 (Pi–alkyl), ARG65 (Pi–cation)	−8.2	Hydrogen bond: GLU35
				Hydrophobic contacts: ARG98, PRO64, ARG61, ASP58, THR127, SER32, ILE63 (Pi–alkyl), ASN31, GLY62 (Amide pi-stacked), ILE129, ASP34, ALA38, ILE79 (Pi–alkyl)
21b	−7.0	Hydrogen bond: ASN35	−9.0	Hydrogen bond: PRO64
		Hydrophobic contacts: MET79, ILE67 (Pi–sigma), ILE78 (Pi–sigma), VAL97, GLU39, ASP34, ALA84, ASP38, ALA42, ARG65, THR142, ASP62, ALA74, PRO68		Hydrophobic contacts: ILE79 (Pi–alkyl), ARG61, ASP34, ASN31, ALA38, ILE63 (Pi–alkyl), GLU35, THR127, SER32, ASP58, GLY62

Furthermore, Gentamicin was docked with bacterial proteins of *E. coli* DNA gyrase and *S. aureus* DNA gyrase as a standard drug. Docking results of the standard drug with bacterial proteins demonstrated a better binding affinity with *E. coli* DNA gyrase, with a docking score of −6.2 kcal mol^−1^ (Table 6). The intermolecular binding affinities that exist between the standard drug and bacterial proteins include conventional hydrogen bonds, carbon–hydrogen bonds, Pi–alkyl interactions, alkyl interactions and van der Waals interactions.

**Table 6:** Molecular docking studies of standard drug with bacterial DNA gyrase

Standard drug	*E. coli* DNA gyrase (PDBID- 1KZN)		*S. aureus* DNA gyrase (PDBID- 3 G7B)	
	Score (kcal mol^−1^)	Intermolecular binding interactions	Score (kcal mol^−1^)	Intermolecular binding interactions
Gentamicin	−6.2	Hydrogen bond: ALA82, ASN32, GLU28	−6.0	Hydrogen bond: SER82, GLU35, SER83
		Hydrophobic interactions: PRO65, GLY63, THR132, GLU36, ARG62, ASP59, ALA33, GLY86, ASP35, ASP31, GLY84, HIS81, VAL87, ILE76 (Pi–alkyl), ILE64 (Pi–alkyl)		Hydrophobic interactions: GLU27, VAL85, ILE79, ASN31, ILE129 (alkyl), THR127, SER32, ASP58, ILE63, GLY62, ARG61, PRO64, ASP34

#### 3.2.2. Antibacterial structure–activity relationship studies (SARs)

**Fig. 10:**
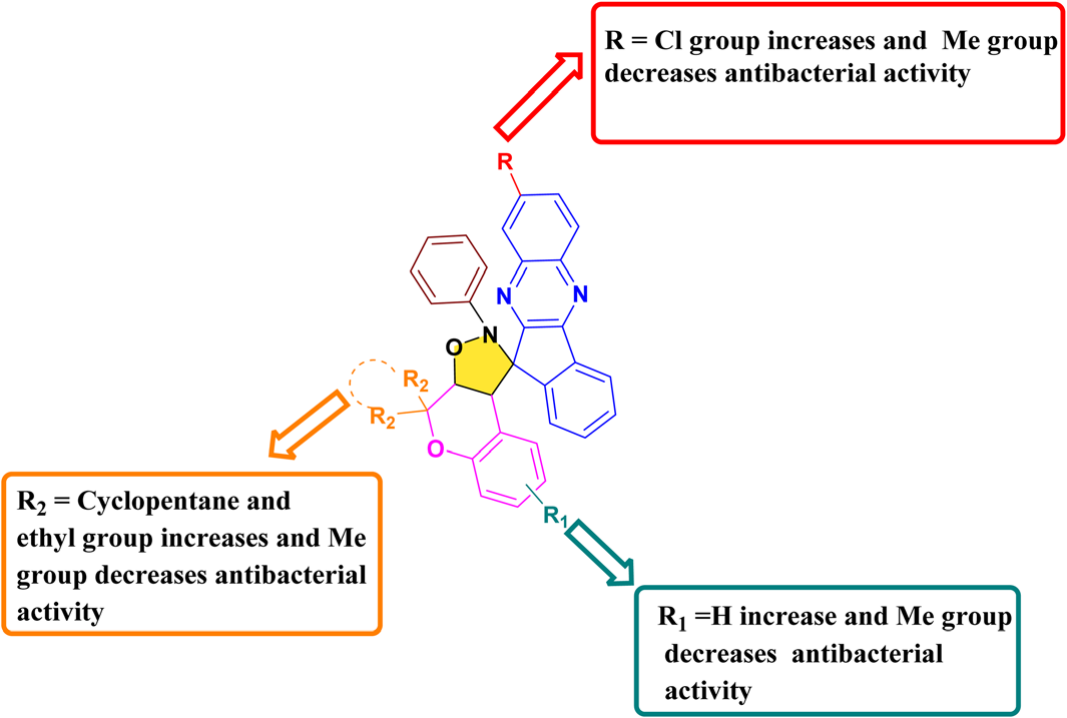
Pictorial representation of the SAR of compound 21(a–r).

Structure–activity relationship (SAR) investigations indicated that the antibacterial efficiency of the synthesized compounds was markedly influenced by the substitution pattern on both the chromene and indenoquinoxaline moieties. Among all the derivatives, the presence of a cyclopentane ring at the R_2_ position of the chromene scaffold in combination with a chloro (–Cl) substituent on the indenoquinoxaline ring significantly enhanced the inhibitory potential against *E. coli* DNA gyrase, affording the highest binding score of −9.8 kcal mol^−1^. Furthermore, the introduction of two ethyl groups at the R_2_ position of the chromene scaffold along with a –Cl substituent on the indenoquinoxaline core also improved antibacterial potency, showing good binding affinity (−8.5 kcal mol^−1^) toward *E. coli* DNA gyrase. Similarly, when the chromene ring was substituted with two ethyl groups at the R_2_ position while the indenoquinoxaline moiety remained unsubstituted, the compounds exhibited enhanced inhibitory activity against *S. aureus* DNA gyrase, with a binding score of −9.0 kcal mol^−1^. In contrast, the presence of a cyclohexane ring at the R_2_ position or dimethyl substitution on the chromene scaffold, particularly in combination with a methyl (–Me) or unsubstituted (–H) indenoquinoxaline ring, resulted in significantly reduced inhibitory potential against *E. coli* DNA gyrase. Likewise, derivatives bearing multiple methyl groups in R, R_1_ and R_2_ position showed poor activity against *S. aureus*. These findings suggest that both the electronic nature of substituents and the steric environment around the spiro framework play crucial roles in enhancing ligand–protein interactions and consequently antibacterial activity (Fig. 10).

### 3.3. Estimation of physicochemical, pharmacokinetic and ADMET properties

#### 3.3.1. *In silico* ADMET studies

*In silico* ADMET analysis was integrated with our experimental assays to better interpret the screening results and to guide the selection of promising analogs with favorable pharmacological profiles for drug development process.^32–34^ These computational assessments provide rapid and cost-efficient predictions of key physicochemical and pharmacokinetic parameters encompassing absorption, distribution, metabolism, excretion, and toxicity, which are commonly associated with attrition in advanced stages of drug discovery.

#### 3.3.2. Physicochemical and medicinal chemistry property prediction

Determining the physio-chemical and medicinal chemistry characteristics of all newly formed compounds will provide a basis to understand how each compound behaves in terms of drug-likeness. Calculated parameters such as lipophilicity, topological polar surface area, molecular flexibility, and hydrogen-bonding capacity were consistent with the structural features observed across the series and supported their overall drug-like profiles.^33–35^ These predictions have helped rationalize the variability in biological activity that is due to the small differences in both polarity and molecular weight that could affect both permeability and target binding. Some important drug-likeness rules include; Lipinski's rule, Veber's rule and Egan's rule which help predict the oral activity of drugs before clinical trials. Lipinski's rule of five suggests that a candidate molecule is more likely to exhibit oral activity if it meets the following criteria: (a) MW ≤ 500 g mol^−1^, (b) Log *P* ≤ 4.15, (c) ^*n*^HD ≤ 5, (d) ^*n*^HA ≤ 10, and (e) no more than one of these criteria is violated.^34–36^ The Veber's rule says that, a molecule is more likely to be orally active if: (a) ^*n*^RB ≤ 10 and (b) TPSA ≤ 140 Å^2^.^37^ Similarly, according to Egan's rule a candidate molecule is more likely to be orally active if: (a) log *P* ≤ 5.88 and (b) TPSA is ≤131.6 Å^2^.^38^

**Fig. 11:**
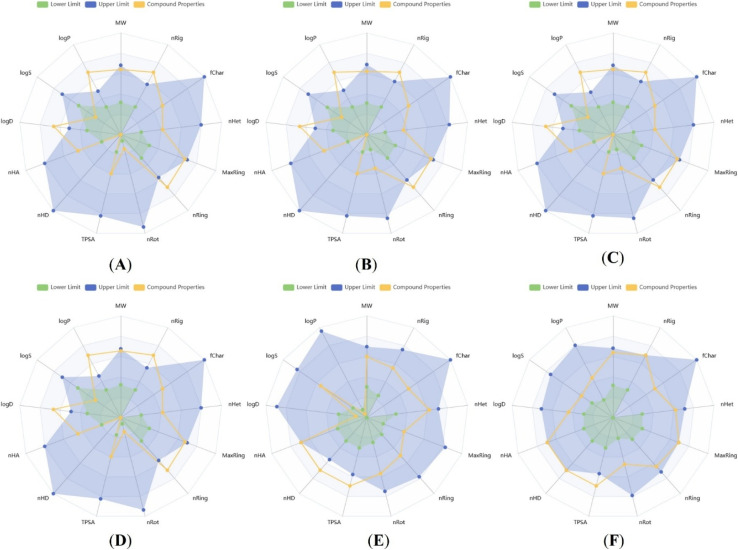
(A–F) Physicochemical radar plots of the four potent compounds (21k, 21b, 21j, and 21r) and two standard drugs (Gentamicin and Doxorubicin) showing the *in silico* measurements of the various descriptors.

The predicted physico-chemical and medicinal chemistry properties of four potent compounds 21k, 21b, 21j, 21r and two standard drugs Gentamicin, Doxorubicin can be seen from the information listed on Table S3 [SI]. As indicated by the results for each compound, each has a total polar surface area (TPSA) less than 140 Å^2^ and no more than 11 rotatable bonds which suggests good oral bioavailability.^34–39^ Also, the molecular weight, hydrogen-bond acceptor count and hydrogen-bond donor count for the selected compounds was found to be in line with what is recommended. The absorption rate two potent compounds and standard drug were calculated using the formula (% ABS = 109 − (0.345 × TPSA)).^32–34^ The evaluated potent compounds exhibit high permeability, absorption, and membrane transport, along with 156.44 to 169.11 m^3^ mol^−1^ molar refractivity and oral absorption percentages more than 50%. Moreover, the physicochemical properties prediction indicated that nearly all the properties of four potent compounds 21k, 21b, 21j, and 21r aligned well with the lower and upper optimal reference values, as depicted in its radar plots Fig. 11(A–F). These four potent compounds exhibited good synthetic accessibility score, bioavailability score, pan assay interference structures, medicinal chemistry evolution, drug-likeness model score and promiscuous compound score in comparison with the standard drugs. Overall, the predictions reveled that all four potent compounds 21k, 21b, 21j, and 21r followed the Veber's rule, whereas the standard drugs Gentamicin and Doxorubicin did not satisfy any of three drug likeness rules (Lipinski's rule, Veber's rule and Egan's rule).

**Fig. 12:**
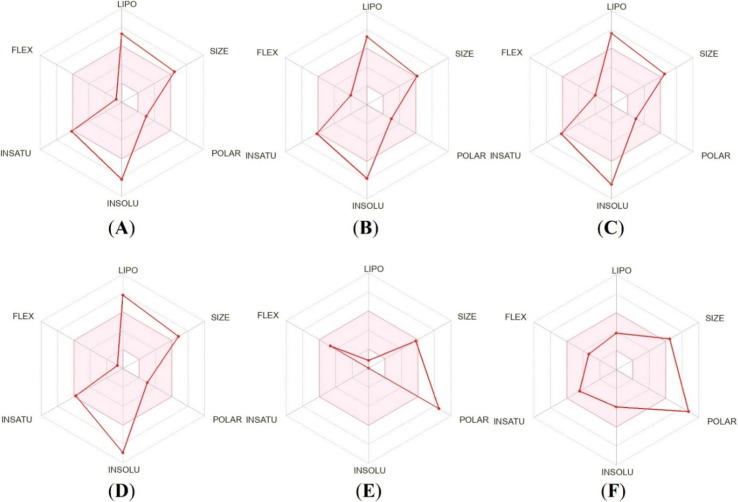
(A–F) Bioavailability radar of the four potent compounds (21k, 21b, 21j, and 21r) and two standard drugs (Gentamicin and Doxorubicin).

The bioavailability radar plot serves as a crucial graphical assessment that shows how well a compound fits with important drug-likeness properties, integrating six key physicochemical descriptors such as lipophilicity (LIPO; 0.7 < log *P* < 5.0), molecular size (150 < MW < 500 g mol^−1^), polarity (POLAR; 20 < TPSA < 130 Å^2^), solubility (INSOLU; 0 < log *S* < 6), insaturation (INSATU; 0.25 < fraction C sp^3^ < 1), and molecular flexibility (FLEX; 0 < number of rotatable bonds <9).^38,40–42^Fig. 12(A–F) presents the bioavailability radar profiles of 21k, 21b, 21j, 21r, Gentamicin, and Doxorubicin. The examined potent compounds display well-balanced physicochemical properties, with most of the parameters lying within the optimal bioavailability region, which indicated favorable drug-likeness and predicted oral bioavailability. In contrast, both the standard drugs Gentamicin and Doxorubicin show pronounced deviations, particularly in polarity and solubility, consistent with their known pharmacokinetic limitations. Overall, the radar plot analysis supports the bioavailability potential of the potent compounds, particularly 21b and 21j.

#### 3.3.3. ADMET property prediction

**Fig. 13:**
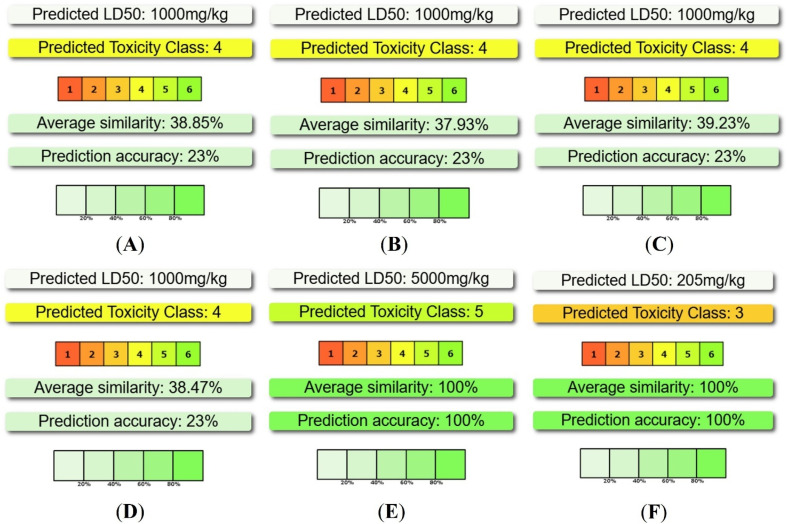
(A–F) Predicted LD_50_ and classes of toxicity the four potent compounds (21k, 21b, 21j, and 21r) and standard drugs (Gentamicin and Doxorubicin).

The *in silico* ADMET profiling of four potent compounds (21k, 21b, 21j, and 21r) and two standard drugs (Gentamicin and Doxorubicin) were examined using AdmetLab 3.0, SwissADME, ProTox 3.0 and pkCSM web tools to assess their suitability as drug-like candidates (Table S4) [SI]. All four compounds demonstrated acceptable Caco-2 and MDCK permeability values, indicating efficient membrane transport, and importantly, were predicted to be non-substrates of P-glycoprotein, suggesting a reduced risk of efflux-mediated bioavailability loss relative to the standard drugs. The HIA scores for all four potent compounds are zero and the predicted GI absorption of all potent compounds as well as standard drugs were low. Therefore, if the oral administration is intended, structural refinements may be needed to enhance permeability. The plasma protein bound percentages for all four compounds fell within the expected ranges. Furthermore, the calculated volumes of distribution indicate that these compounds will distribute moderately throughout the body. All potent compounds and standard drugs showed blood–brain barrier permeability, highlighting their ability to access the central compartment. The CYP interaction profiles reveal notable differences between the four potent compounds and standard drugs Gentamicin and Doxorubicin. Metabolic predictions suggested that the compounds were predicted to interact with several major cytochrome P450 isoforms, including CYP1A2, CYP2C9, CYP2C19, and CYP3A4, functioning as both inhibitors and substrates. These predictions indicate possible metabolic complexity and warrant further experimental evaluation of CYP-mediated drug–drug interaction potential. The predicted plasma clearance values of potent compounds fall below the recommended range (5–15 mL min^−1^ kg^−1^), suggesting slow systemic clearance. Their half-lives (0.344–0.677 h) are shorter but remain within a workable range for compounds intended for short-duration exposure. The toxicity properties prediction of the potent compounds showed no AMES mutagenicity or hepatotoxicity for any of the compounds. Although hERG II inhibition was predicted, the absence of hERG I inhibition suggests a lower risk of severe cardiotoxicity. Furthermore, all four potent compounds were classified under toxicity class 4 with predicted LD_50_ values of 1000 mg kg^−1^, indicating a safer acute toxicity profile than Doxorubicin (Fig. 13(A–F)). Collectively, these findings provide evidence for the drug-likeness and preliminary safety of the synthesized potent compounds, justifying their progression toward experimental pharmacokinetic and biological studies.

## 4. Photophysical and metal ion sensing studies

### 4.1. Photophysical studies

#### 4.1.1. UV-visible and fluorescence study

**Fig. 14:**
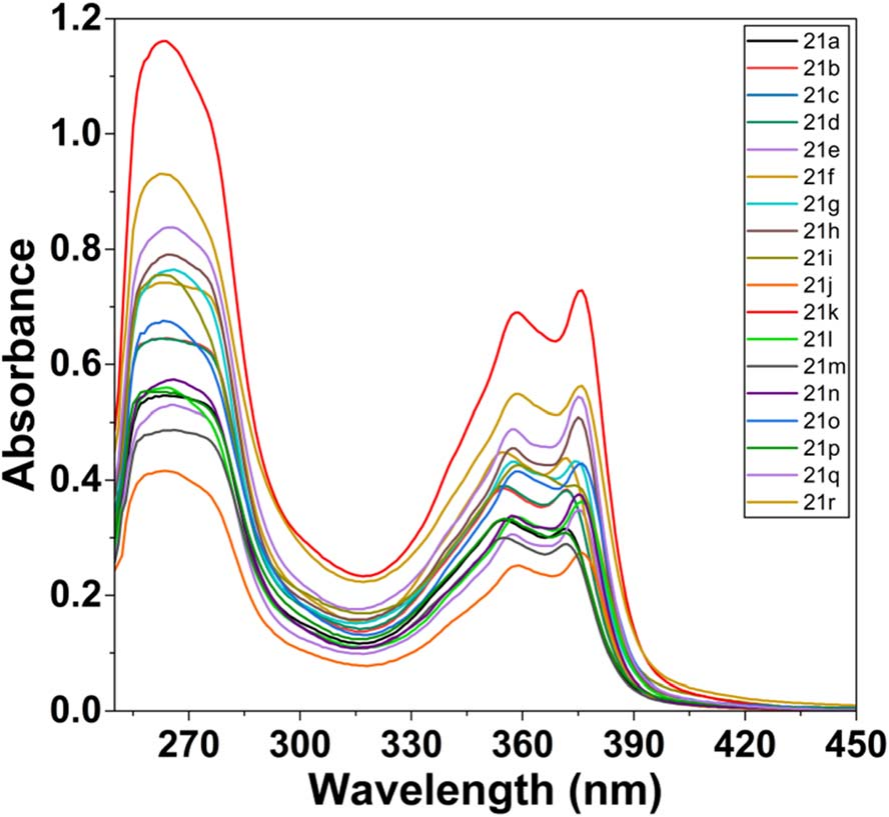
UV-visible spectra of all ligands 21(a–r) (20 µM) in DMSO.

**Fig. 15:**
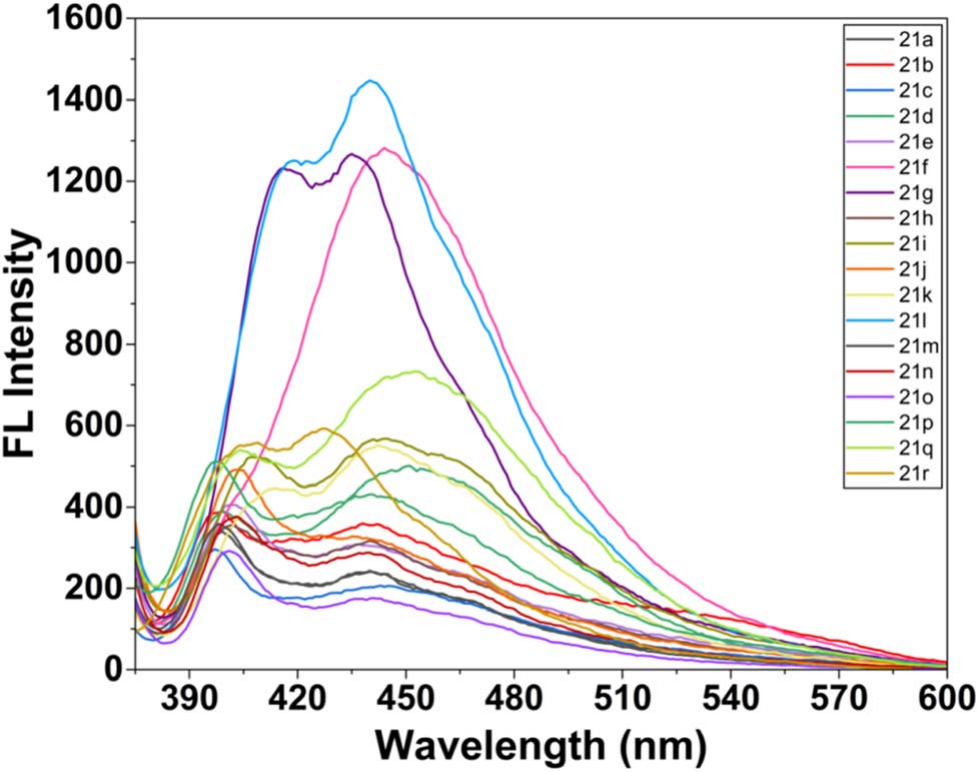
Fluorescence emission spectra of all ligands 21(a–r) (20 µM) in DMSO.

The optical properties of the synthesized dihydrospiro[chromeno-isoxazole-indenoquinoxaline] derivatives were systematically examined using UV-visible spectroscopy (UV-2600). All compounds were recorded in DMSO at a fixed concentration of 20 µM to maintain uniform experimental conditions and allow reliable comparison of their absorption maxima. All the synthesized derivatives 21(a–r) showed characteristic absorption bands in the UV region, indicating effective π-conjugation within the heterocyclic framework. Among the series, compound 21k exhibited the most intense and well-defined absorption maxima, suggesting enhanced electronic delocalization and improved photophysical properties.^43^ Therefore, 21k was selected for further metal-ion sensing studies (Fig. 14). Further, the fluorescence emission properties of all compounds (20 µM, DMSO) were also investigated. The derivatives displayed noticeable emission bands, confirming their intrinsic fluorescent nature (Fig. 15). Most of the compounds showed good fluorescence intensity, indicating their potential suitability for sensing applications.^44^

### 4.2. Metal ion sensing studies

#### 4.2.1. UV-visible study

**Fig. 16:**
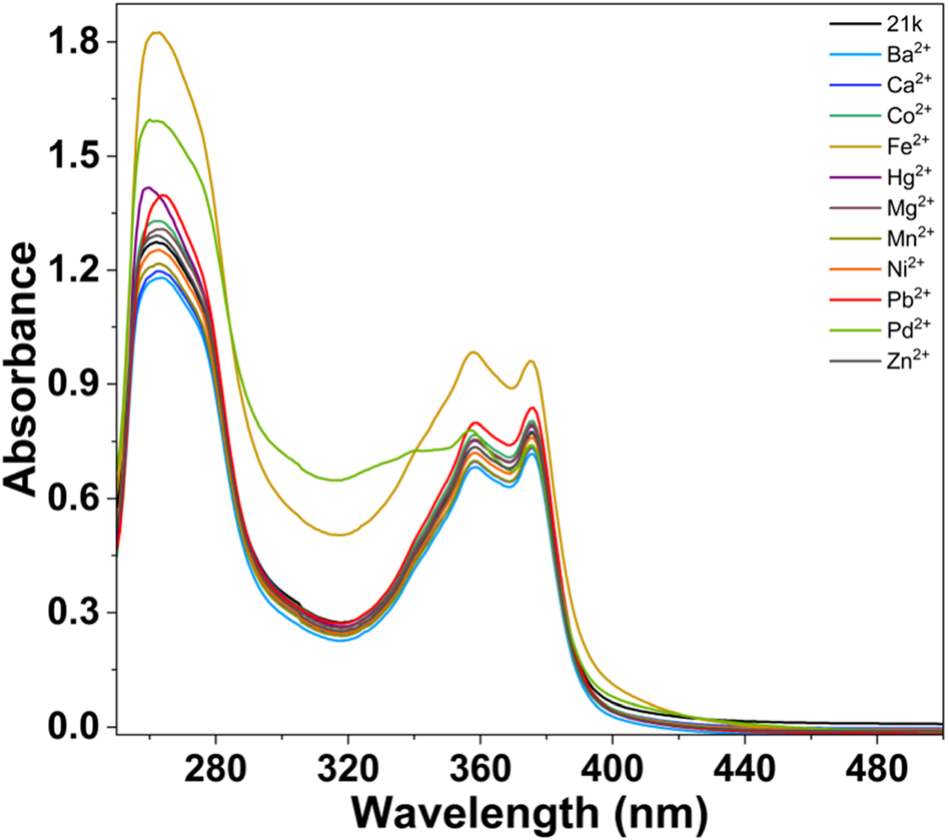
UV-visible spectra of 21k in DMSO upon addition of different metal ions.

The metal-ion sensing ability of ligand 21k was first examined by recording its UV-Vis absorption spectrum in absence and presence of various relevant metal ions (Ba^2+^, Ca^2+^, Co^2+^, Fe^2+^, Hg^2+^, Mg^2+^, Mn^2+^, Ni^2+^, Pb^2+^, Pd^2+^, Zn^2+^). The free ligand displayed a major absorption band centred at 262 nm, corresponding to π–π* transitions, with shoulder absorption bands at 358 and 378 nm, attributes to n–π* transitions of the heteroaromatic core. It has been observed that no other metal ions produced any marginal spectral perturbation *via* interacting with ligand. In contrast, Fe^2+^ and Pd^2+^ ions produced a significant optical response with 21k ligand, and discriminating them out from the other metal ions (Fig. 16).^45^

**Fig. 17:**
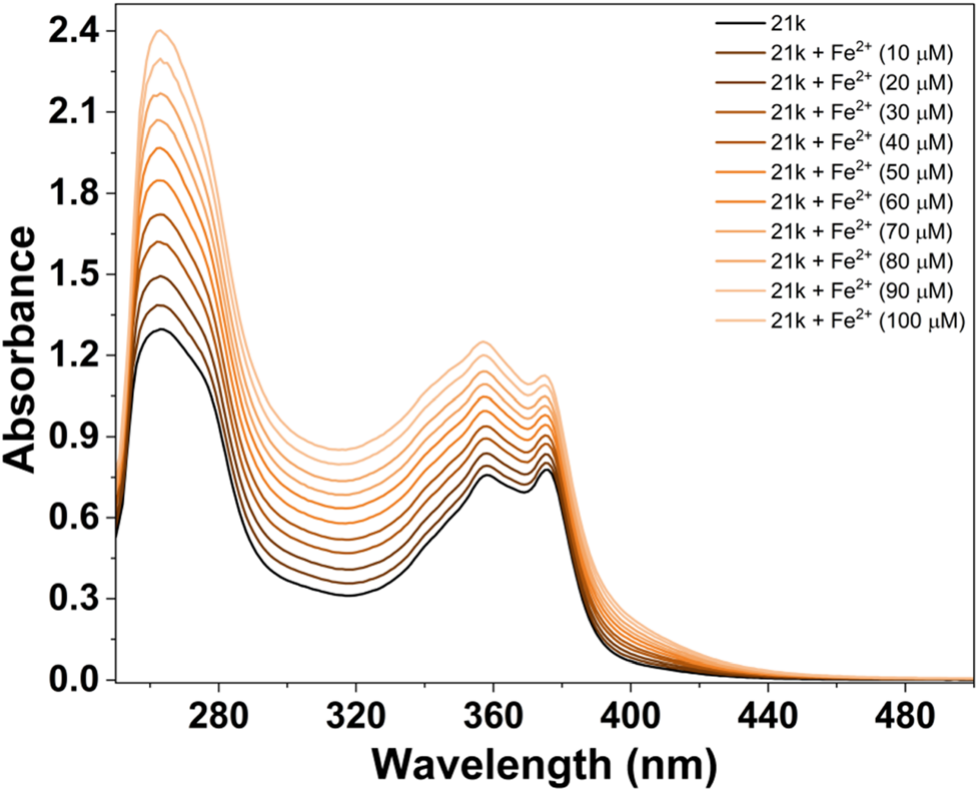
UV-visible titration spectra of 21k (20 µM) in DMSO with addition of the Fe^2+^ ion solutions ([Fe^2+^] = 0–100 µM).

Upon addition of Fe^2+^, 21k exhibited a noticeable hyperchromic shift, with the absorbance intensity at the high-energy band increasing significantly and a new shoulder appearing around 365 nm. This strong enhancement in absorbance intensity indicates robust coordination between Fe^2+^ and the ligand, most likely facilitated by the high Lewis acidity of Fe^2+^ and its strong affinity for O/N donor sites.^46^ The nature of the spectral changes as absorbance intensity growth without major band shifting suggests the formation of a stable 21k–Fe^2+^ complex accompanied by ligand-to-metal charge transfer (LMCT).^47^ The sharp deviation of the Fe^2+^ spectrum from those of other ions confirms Fe^2+^ as a high-affinity, selectively sense analyte. To further validate the Fe^2+^ sensing ability of 21k, a systematic UV-Vis titration experiment was carried out by gradually addition of Fe^2+^ into the ligand solution. Upon incremental additions of Fe^2+^, the absorption profile displayed clear and reproducible spectral changes. A steady, concentration-dependent enhancement in absorbance was observed across the main π → π* transition band, accompanied by a progressive increase in the shoulder region around 356 nm. Notably, no significant bathochromic or hypsochromic shifts occurred during titration, indicating that the ligand maintains a stable electronic environment upon metal coordination. This behavior suggests the formation of a structurally robust Fe^2+^-ligand adduct without alteration of the fundamental chromophoric framework (Fig. 17).^48^

In contrast to Fe^2+^, the interaction of 21k with Pd^2+^ produced a well-defined and reproducible spectral response, confirming that the ligand is also capable of selectively sensing Pd^2+^. Unlike Fe^2+^, which induces strong hyperchromism, Pd^2+^ causes a moderate but clearly distinguishable enhancement in absorbance, accompanied by characteristic band broadening and re-shaping in the region of 329 nm. This alteration in peak profile, particularly the rise and widening of the n–π* band does not occur with any other competing metal ions, making the Pd^2+^ spectrum uniquely identifiable. The specific spectral perturbation suggests that Pd^2+^ binds to 21k through a coordination mode that partially modifies the ligand's conjugated framework, likely involving d–π orbital interactions. This interaction stabilizes new electronic transitions, resulting in selective intensity enhancement.^49^

To examine the Pd^2+^ sensing performance of 21k, a UV-Vis titration experiment was carried out by gradually increasing the concentration of Pd^2+^ in the ligand solution. Unlike Fe^2+^, the addition of Pd^2+^ did not induce hyperchromism of the same magnitude; however, it produced a clear, concentration-dependent enhancement in specific spectral regions, confirming a distinct interaction between 21k and Pd^2+^.

**Fig. 18:**
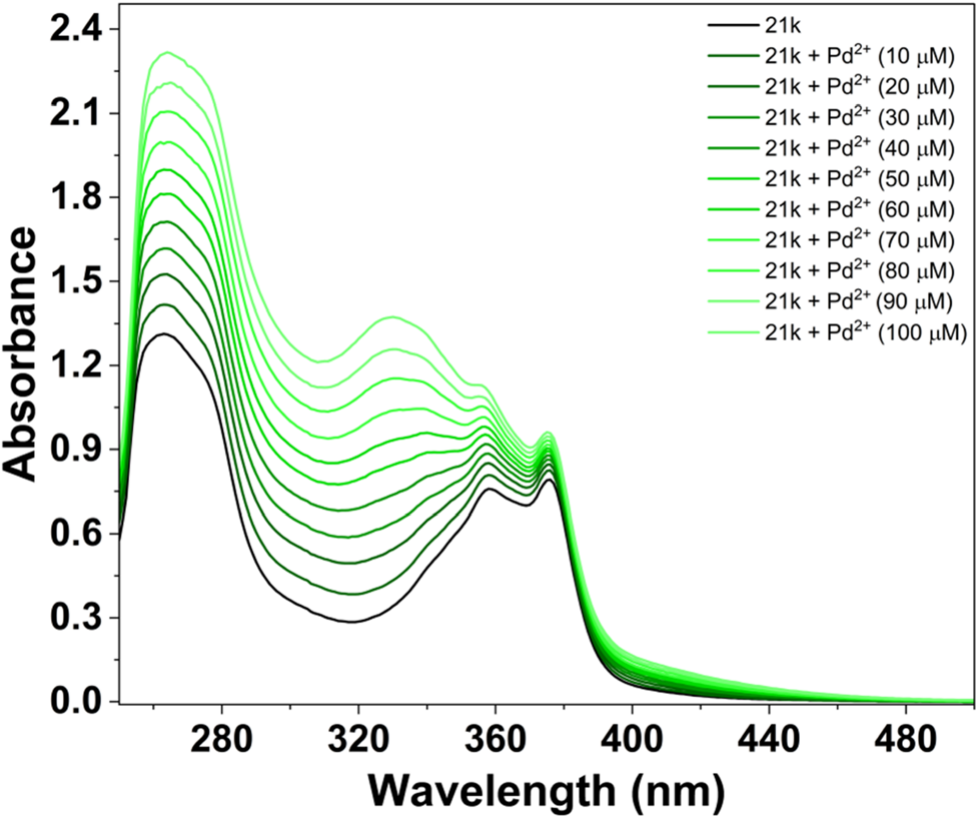
UV-visible titration spectra of 21k (20 µM) in DMSO with addition of the Pd^2+^ ion solutions ([Pd^2+^] = 0–100 µM).

With successive additions of Pd^2+^, the π → π* transition band exhibited a moderate but steady increase in absorbance, while the n → π* band showed noticeable broadening and elevation, particularly in the 350–390 nm range. The development of this broadened feature is a characteristic signature of Pd^2+^ binding and demonstrates that the ligand undergoes measurable electronic reorganization upon coordination with Pd^2+^. Importantly, the absence of wavelength shifts suggests that the fundamental chromophoric framework remains intact, and the spectral changes arise primarily from modification of transition intensities rather than alteration of the electronic transition energy (Fig. 18).^50^

#### 4.2.2. Fluorescence study

**Fig. 19:**
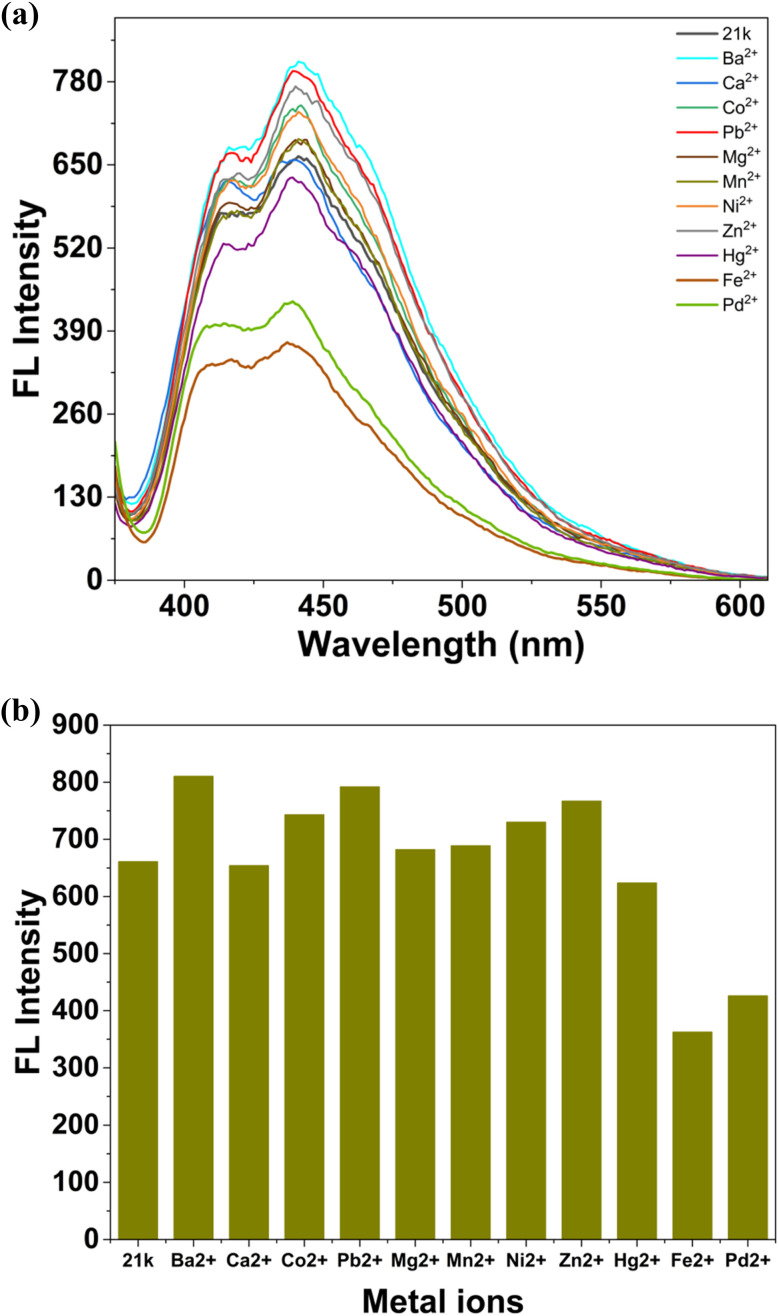
(a) Fluorescence emission spectra of 21k in DMSO (b) histogram of fluorescence intensities of 21k with different metal ions at 442 nm.

Besides UV-visible spectroscopy, fluorescence spectroscopy was also used to evaluate the change in optical properties of 21k and environmentally relevant metal ions (Ba^2+^, Ca^2+^, Co^2+^, Pb^2+^, Mg^2+^, Mn^2+^, Ni^2+^, Zn^2+^, Hg^2+^, Fe^2+^, Pd^2+^). The free ligand exhibits a strong emission band centered around 442 nm in pure DMSO solvent. Upon addition of most competing ions, only minor changes in emission intensity were recorded, indicating negligible interaction with the fluorophore. In contrast, Fe^2+^ and Pd^2+^ produced noticeable fluorescence quenching, with Fe^2+^ showing the strongest suppressive effect, reducing the emission intensity by more than 40%. Pd^2+^ also induced a significant downward shift in fluorescence intensity, though slightly weaker than Fe^2+^. The selective quenching effect for these two ions clearly demonstrates that 21k possesses dual-sensing capability in fluorometric mode. The minimal interference from other ions highlights the high selectivity of 21k toward Fe^2+^ and Pd^2+^, further supporting its potential application in metal-ion detection (Fig. 19(a) and (b)).^51–53^

**Fig. 20:**
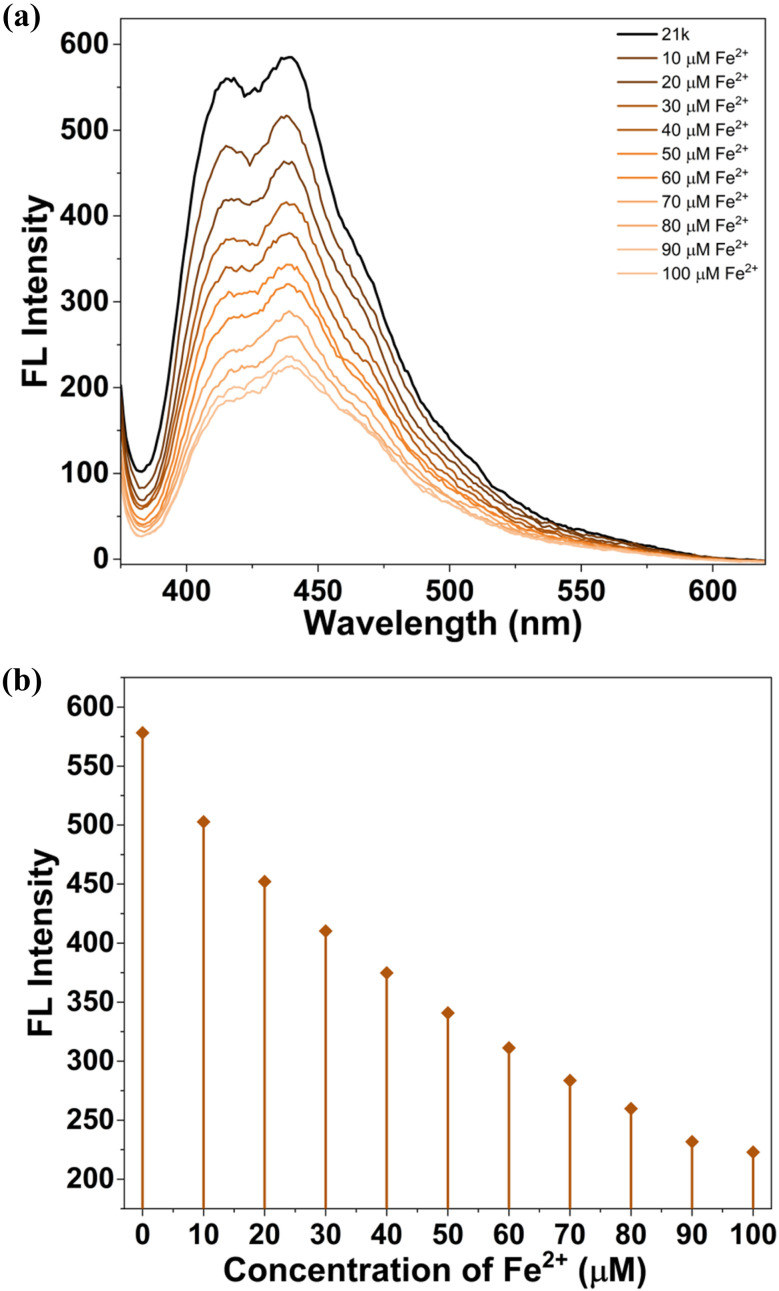
(a) Fluorescence titration spectra of 21k in DMSO (20 µM) at different iron ion concentrations ([Fe^2+^] = 0–100 µM). (b) Change of fluorescence intensities at 442 nm while adding Fe^2+^ ions to 21k.

**Fig. 21:**
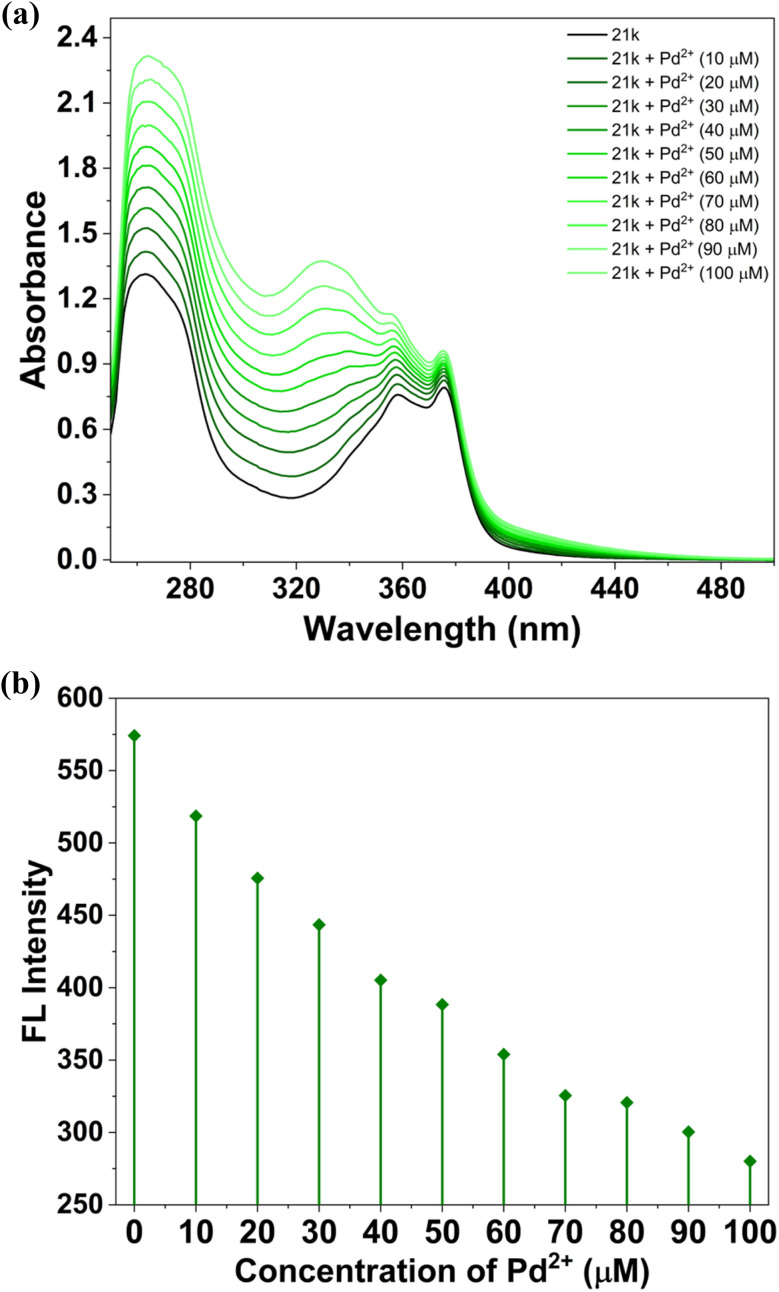
(a) Fluorescence titration spectra of 21k in DMSO (20 µM) at different iron ion concentrations ([Pd^2+^] = 0–100 µM). (b) Change of fluorescence intensities at 442 nm while adding Pd^2+^ ions to 21k.

The spectroscopic investigation based on concentration-dependent fluorescence titration of 21k with Fe^2+^ and Pd^2+^ ions (0–100 µM) revealed a clear and progressive quenching of the primary emission band at 442 nm, accompanied by a gradual suppression of the minor shoulder around 415 nm. The absence of any measurable wavelength shift throughout the titration indicates that Fe^2+^ and Pd^2+^ ions binding does not alter the chromophore environment, instead forming a stable ground-state complex (Fig. 20(a, b), 21(a and b)).^54^

From both UV-visible and fluorescence titration studies it was undoubtedly confirmed that 21k strongly interacts with Fe^2+^ and Pd^2+^ ions as a remarkable sensor which could be meticulously employed for the detection and discrimination of Fe^2+^ and Pd^2+^ions.

## 5. Conclusion

In conclusion, we have successfully designed and synthesized a new series of structurally complex dispiro chromeno-indenoquinoxaline heterocyclic hybrids through an efficient and greener strategy. The structures of all compounds were unambiguously confirmed using comprehensive spectroscopic and analytical techniques. *In vitro* biological evaluation demonstrated that most derivatives exhibited significant anticancer and antibacterial activities against the tested cancer cell lines and pathogenic bacterial strains, identifying this scaffold as a promising platform for multifunctional drug development. In addition, the synthesized compounds displayed notable fluorescence responses toward selected metal ions, highlighting their potential utility in metal sensing and related analytical applications.

## 6. Experimental section

Reactants and reagents were purchased from standard suppliers (Sigma-Aldrich, TCI) and were used without further purification. *o*-Xylene, MeOH and THF were dried according to conventional methods. Reaction progress was monitored by Thin Layer Chromatography (TLC) on silica gel 60 (F254) coated aluminium plates. Visualization of spots on TLC plate was accomplished with UV light (254 nm). Column chromatography purifications were performed over silica gel (mesh 100–200). NMR spectra were recorded on JEOL FT (400 MHz for ^1^H, 100 MHz for ^13^C) spectrometers. Chemical shifts are expressed in parts per million (ppm) and coupling constants are in Hz and splitting pattern abbreviations are: s, singlet; d, doublet; dd, doublet of doublets; t, triplet; m, multiplet. High-resolution mass spectra (HRMS) were recorded using a Bruker micro TOF-QII mass spectrometer at IIT-Bombay. Melting points (mp) were determined on a SMP10 apparatus and were uncorrected. The UV visible, fluorescent spectra and metal sensing properties were taken in a Shimadzu single monochromator UV-2600 spectrophotometer and HITACHI F-7000 fluorescence spectrometer machine at Sambalpur University.

## 7. Experimental procedure

### 7.1. General experimental procedure for the preparation of substituted indenoquinoxaline derivatives 11(a–c)

In a clean and dry 100 mL round-bottom flask, a mixture of ninhydrin 9 (1 equiv.) and substituted 1,2-phenylenediamine 10(a–c) (1.2 equiv.) was introduced into ethanol. The resulting solution was stirred at ambient temperature for approximately 15 minutes. The progress of the reaction was monitored by TLC, upon completion, the resulting indenoquinoxaline derivatives 11(a–c) were obtained by filtration and subsequently purified by washing with ethanol.

### 7.2. General experimental procedure for the preparation of substituted indenoquinoxaline imine 13(a–c)

Substituted indenoquinoxaline derivatives 11(a–c) (1.0 equiv.) and *p*-toluidine 12 (2.0 equiv.) were subjected to a condensation reaction in the mixture of ethanol and glacial acetic acid (2 : 1). The reaction mixture were refluxed with continuous stirring for approximately 3 hours. The progress of the reaction was monitored by TLC, and was found to be completed after 3 h. After completion, the reaction mixture was cooled in an ice bath, and the resulting red-coloured precipitate 13(a–c) was collected by filtration and subsequently purified by washing with ethanol.

### 7.3. General experimental procedure for the preparation of substituted indenoquinoxaline based nitrone 15(a–c)

Substituted indenoquinoxaline imine 13(a–c) (1 equiv.) and phenyl hydroxylamine 14 (1.5 equiv.) was introduced into a clean, dry round-bottom flask and dissolved in 8–10 mL of chloroform. The resulting reaction mixture was stirred under heating at 50–60 °C for 30–45 min. The progress of the reaction was monitored by TLC until completion. After completion, the mixture was cooled in an ice bath, and the yellow precipitate formed was collected by filtration. The crude product was then purified by washing with cold ethanol to afford the corresponding novel indenoquinoxaline nitrone derivatives 15(a–c).

### 7.4. General experimental procedure for preparation of substituted *2H*-chromene 20(a–f)

In a thoroughly cleaned and dried 100 mL round-bottom flask, substituted *o*-hydroxyacetophenone 16(a–b) (1.0 equiv.), substituted ketone 17(a–d) (3.0 equiv.), and pyrrolidine (0.5 equiv.) were introduced and subjected to reflux with constant stirring for 6–8 h. The reaction progress was monitored by TLC. After completion, the reaction mixture was allowed to cool to room temperature, diluted with water, and extracted with ethyl acetate. The organic layer was separated, washed with a brine solution, dried over anhydrous sodium sulfate (Na_2_SO_4_), and concentrated under reduced pressure. The resulting crude product was purified through column chromatography using ethyl acetate/hexane as the mobile phase to afford the pure compound Chroman-4-one 18(a–f).

Chroman-4-one 18(a–f) was dissolved in 5 mL of methanol, and sodium borohydride (NaBH_4_) (1.3 equiv.) was introduced to the solution at room temperature. The mixture was stirred continuously for about 30 minutes to facilitate complete reduction, and the progress of the reaction was monitored by TLC. Upon completion, the reaction mixture was quenched with water and extracted with ethyl acetate. The combined organic layers were dried over anhydrous sodium sulfate (Na_2_SO_4_), filtered, and concentrated under reduced pressure using a rotary evaporator to yield the corresponding chroman-4-ol 19(a–f) as a crude product. The obtained intermediate 19(a–f) was directly utilized in the subsequent reaction step without further purification.

The crude chroman-4-ol 19(a–f) was treated with *p*-toluenesulfonic acid (*p*-TsOH) (1.2 equiv.) in 2 mL of dry THF under reflux condition for 3–4 hours. The reaction progress was monitored by TLC, which confirmed the successful formation of the substituted *2H*-chromene derivatives 20(a–f). After completion, the mixture was allowed to cool to room temperature and then extracted with ethyl acetate and water. The organic layer was separated, dried over anhydrous sodium sulfate (Na_2_SO_4_), filtered, and concentrated under reduced pressure. The crude product thus obtained was purified by column chromatography using an ethyl acetate/hexane mixture to afford the pure *2H*-chromene derivatives 20(a–f).

### 7.5. General experimental procedure for preparation of chromene-fused spiro cyclic indenoquinoxaline-derived isoxazolidine derivatives 21(a–r)

To an oven-dried sealed tube fitted with a magnetic stir bar were added *N*-phenyl indenoquinoxaline-based nitrone 15(a–c) (1.0 equiv.) and the corresponding *2H*-chromene 20(a–f) (2.0 equiv.). The mixture was suspended in water (5 mL), sealed, and heated at 100 °C in an oil bath under continuous stirring for about 4–5 h. The progress of the reaction was periodically checked by TLC, and full conversion was achieved within 5 h. After completion of reaction, the reaction mixture was cooled to room temperature, concentrated under reduced pressure, and the crude product was purified by silica gel column chromatography using a gradient of hexane/ethyl acetate to afford the desired novel chromene-fused spiro indenoquinoxaline-derived isoxazolidine derivatives 21(a–r). The structures of the products were confirmed by ^1^H NMR, ^13^C NMR, and HRMS analyses.

#### 7.5.1. 4,4-Dimethyl-2-phenyl-3*a*,9*b*-dihydro-2*H*,4*H*-spiro[chromeno[4,3-*d*]isoxazole-1,11′-indeno[1,2-*b*]quinoxaline] (21a)

Yield (86%) as a yellow solid, M. P. = 205–207 °C, ^1^H NMR (400 MHz, CDCl_3_): *δ* (ppm) 8.28–8.26 (m, 1H), 8.15–8.12 (m, 1H), 7.79–7.69 (m, 3H), 7.36 (d, *J* = 8.0 Hz, 1H), 7.04–7.00 (m, 1H), 6.93–6.89 (m, 1H), 6.80–6.71 (m, 3H), 6.66 (dd, *J*_12_ = 1.2 Hz, *J*_13_ = 8.4 Hz, 1H), 6.59 (t, *J* = 7.6 Hz, 1H), 6.41–6.39 (m, 2H), 6.27–6.23 (m, 1H), 5.88–5.86 (m, 1H), 5.01–4.90 (m, 2H), 1.76 (s, 3H), 1.22 (s, 3H). ^13^C NMR (100 MHz, CDCl_3_): *δ* (ppm) 160.4, 154.9, 153.1, 146.0, 143.3, 143.0, 141.9, 136.5, 130.8, 130.3, 129.8, 129.2 (2C), 128.9, 128.2, 128.0 (3C), 127.4, 123.1, 122.1, 121.3, 121.0, 118.0, 117.1 (2C), 81.3, 80.9, 76.2, 52.7, 25.0, 23.7. HRMS (ESI) calculated for C_32_H_25_N_3_O_2_ [M + H]^+^ 484.2025, found 484.2018.

#### 7.5.2. 4,4-Diethyl-2-phenyl-3*a*,9*b*-dihydro-2*H*,4*H*-spiro[chromeno[4,3-*d*]isoxazole-1,11′-indeno[1,2-*b*]quinoxaline] (21b)

Yield (83%) as a yellow solid, M. P. = 188–190 °C, ^1^H NMR (400 MHz, CDCl_3_): *δ* (ppm) 8.29–8.27 (m, 1H), 8.14 (dd, *J*_12_ = 1.2 Hz, *J*_13_ = 8. Hz, 1H), 7.80–7.69 (m, 3H), 7.38 (d, *J* = 7.6 Hz, 1H), 7.04–6.99 (m, 1H), 6.93–6.89 (m, 1H), 6.79–6.72 (m, 3H), 6.67 (d, *J* = 7.2 Hz, 1H), 6.62–6.58 (m, 1H), 6.39 (d, *J* = 7.6 Hz, 2H), 6.26–6.22 (m, 1H), 5.86–5.84 (m, 1H), 5.01–4.94 (m, 2H), 2.30–2.24 (m, 1H), 2.07–2.02 (m, 1H), 1.67–1.45 (m, 2H), 1.12 (t, *J* = 7.6 Hz, 3H), 0.97 (t, *J* = 7.2 Hz, 3H). ^13^C NMR (100 MHz, CDCl_3_): *δ* (ppm) 160.4, 155.0, 152.5, 146.1, 143.4, 143.0, 141.9, 136.5, 130.8, 130.3, 129.8 (2C), 129.2, 128.8, 128.1, 128.0 (2C), 127.6, 127.4, 123.1, 122.9, 121.2, 121.0, 118.0, 117.1 (2C), 81.2, 80.7, 79.1, 52.5, 25.4, 23.1, 8.3, 6.1. HRMS (ESI) calculated for C_34_H_29_N_3_O_2_ [M + H]^+^ 512.2338, found 512.2335.

#### 7.5.3. 2′-Phenyl-3*a*′,9*b*′-dihydro-2′*H*-dispiro[cyclopentane-1,4′-chromeno[4,3-*d*]isoxazole-1′,11″-indeno[1,2-*b*]quinoxaline] (21c)

Yield (87%) as a bright yellow solid, M. P. = 190–192 °C, ^1^H NMR (400 MHz, CDCl_3_): *δ* (ppm) 8.35–8.33 (m, 1H), 8.22–8.19 (m, 1H), 7.86–7.77 (m, 3H), 7.46 (d, *J* = 8.0 Hz, 1H), 7.11–7.07 (m, 1H),7.01–6.96 (m, 1H), 6.87–6.78 (m, 3H), 6.73 (dd, *J*_12_ = 0.8 Hz, *J*_13_=8.4 Hz, 1H), 6.69–6.64 (m, 1H), 6.49–6.46 (m, 2H), 6.35–6.31 (m, 1H), 5.90–5.87 (m, 1H), 5.07 (s, 2H), 2.45–2.42 (m, 1H), 2.33–2.27 (m, 1H), 2.10–1.98 (m, 1H), 1.93–1.68 (m, 4H), 1.65–1.59 (m, 1H). ^13^C NMR (100 MHz, CDCl_3_): *δ* (ppm) 160.5, 154.9, 153.7, 146.1, 143.4, 143.0, 141.9, 136.5, 130.8, 130.3, 129.8, 129.3, 129.2, 128.8, 128.3, 128.0 (3C), 127.6, 123.1, 122.9, 121.4, 121.0, 118.3, 117.1 (2C), 87.5, 82.2, 81.1, 53.2, 35.8, 34.6, 23.9, 23.7. HRMS (ESI) calculated for C_34_H_27_N_3_O_2_ [M + H]^+^ 510.2181, found 510.2175.

#### 7.5.4. 2′-Phenyl-3*a*′,9*b*′-dihydro-2′*H*-dispiro[cyclohexane-1,4′-chromeno[4,3-*d*]isoxazole-1′,11″-indeno[1,2-*b*]quinoxaline] (21d)

Yield (89%) as a bright yellow solid, M. P. = 185–187 °C, ^1^H NMR (400 MHz, CDCl_3_): *δ* (ppm) 8.14 (dd, *J*_12_ = 2 Hz, *J*_13_ = 1.2 Hz, 1H), 8.28–8.26 (m, 1H), 7.79–7.69 (m, 3H), 7.37 (d, *J* = 8.0 Hz, 1H), 7.03–6.99 (m, 1H), 6.94–6.89 (m, 1H), 6.81–6.67 (m, 4H), 6.61–6.57 (m, 1H), 6.41–6.38 (m, 2H), 6.27–6.22 (m, 1H), 5.90–5.87 (m, 1H), 4.99–4.93 (m, 2H), 2.37–2.24 (m, 1H), 2.11–1.91 (m, 2H), 1.76–1.53 (m, 3H), 1.49–1.35 (m, 4H). ^13^C NMR (100 MHz, CDCl_3_): *δ* (ppm) 160.6, 154.9, 152.6, 146.1, 143.4, 143.0, 141.9, 136.5, 130.8, 130.3, 129.9, 129.2, 128.8, 128.1 (2C), 127.9 (3C), 127.5, 123.1, 122.8, 121.3, 121.0, 118.1, 117.2 (2C), 80.7, 80.6 (2C), 52.6, 33.0, 31.0, 25.5, 21.4, 21.0. HRMS (ESI) calculated for C_35_H_29_N_3_O_2_ [M + H]^+^ 524.2338, found 524.2348.

#### 7.5.5. 4,4,8′-Trimethyl-2-phenyl-3*a*,9*b*-dihydro-2*H*,4*H*-spiro[chromeno[4,3-*d*]isoxazole-1,11′-indeno[1,2-*b*]quinoxaline] (21e)

Yield (82%) as a light yellow solid, M. P. = 178–180 °C, ^1^H NMR (400 MHz, CDCl_3_): *δ* (ppm) 8.15 (d, *J* = 8.4 Hz, 1H), 7.9 (s, 1H), 7.68 (d, *J* = 7.2 Hz, 1H), 7.56 (dd, *J*_12_ = 2.0 Hz, *J*_13_ = 8.8 Hz, 1H), 7.35 (d, *J* = 7.6 Hz, 1H), 7.00–6.98 (m, 1H), 6.92–6.88 (m, 1H), 6.79–6.70 (m, 3H), 6.66 (d, *J* = 7.6 Hz, 1H), 6.59 (t, *J* = 7.6 Hz, 1H), 6.39 (d, *J* = 8.0 Hz, 2H), 6.26–6.22 (m, 1H), 5.86 (d, *J* = 6.8 Hz, 1H), 5.00–4.88 (m, 2H), 2.58 (s, 3H), 1.76 (s, 3H), 1.20 (s, 3H). ^13^C NMR (100 MHz, CDCl_3_): *δ* (ppm) 159.4, 154.8, 153.1, 146.0, 143.3, 143.0, 141.0, 140.3, 136.7, 131.4, 130.6, 129.3, 128.8, 128.3, 128.1, 128.0, 127.9 (2C), 127.4, 123.1, 122.2, 121.3, 120.9, 118.0, 117.1 (2C), 81.3, 80.9, 76.2, 52.6, 25.0, 23.7, 21.8. HRMS (ESI) calculated for C_33_H_27_N_3_O_2_ [M + H]^+^ 498.2181, found 498.2179.

#### 7.5.6. 4,4-Diethyl-8′-methyl-2-phenyl-3*a*,9*b*-dihydro-2*H*,4*H*-spiro[chromeno[4,3-*d*]isoxazole-1,11′-indeno[1,2-*b*]quinoxaline] (21f)

Yield (79%) as a light yellow solid, M. P. = 178–180 °C, ^1^H NMR (400 MHz, CDCl_3_): *δ* (ppm).16 (d, *J* = 8.8 Hz, 1H), 7.92 (s, 1H), 7.68 (d, *J* = 7.2 Hz, 1H), 7.57 (dd, *J*_12_ = 2 Hz, *J*_13_ = 8.8 Hz, 1H), 7.38 (d, *J* = 7.6 Hz, 1H), 7.02–6.98 (m, 1H), 6.92–6.88 (m, 1H), 6.79–6.72 (m, 3H), 6.67 (t, *J* = 1.2 Hz, 1H), 6.59 (t, *J* = 7.6 Hz, 1H), 6.38 (dd, *J*_12_ = 1.2 Hz, *J*_13_=3.2 Hz, 2H), 6.25–6.21 (m, 1H), 5.86 (d, *J* = 6.8 Hz, 2H), 5.00–4.93 (m, 2H), 2.59 (s, 3H), 2.29–2.24 (m, 1H), 2.07–2.02 (m, 1H), 1.69–1.49 (m, 2H), 1.12 (t, *J* = 7.6 Hz, 3H), 0.77 (t, *J* = 7.2 Hz, 3H). ^13^C NMR (100 MHz, CDCl_3_): *δ* (ppm) 159.4, 154.9, 152.5, 146.1, 143.4, 143.0, 141.0, 140.3, 136.6, 131.4, 130.6, 129.3, 128.7, 128.3, 128.1, 127.9 (2C), 127.8, 127.3, 123.1, 122.9, 121.2, 120.9, 118.0, 117.1 (2C), 81.2, 80.6, 79.1, 52.4, 25.4, 23.1, 21.8, 8.3, 6.1. HRMS (ESI) calculated for C_35_H_31_N_3_O_2_ [M + H]^+^ 526.2494, found 526.2498.

#### 7.5.7. 8″-Methyl-2′-phenyl-3*a*′,9*b*′-dihydro-2′*H*-dispiro[cyclopentane-1,4′-chromeno[4,3-*d*]isoxazole-1′,11″-indeno[1,2-*b*]quinoxaline] (21g)

Yield (83%) as a light yellow solid, M. P. = 206–208 °C, ^1^H NMR (400 MHz, CDCl_3_): *δ* (ppm) 8.15 (d, *J* = 8.8 Hz, 1H), 7.91 (s, 1H), 7.68 (d, *J* = 8.0 Hz, 1H), 7.57 (dd, *J*_12_ = 1.6 Hz, *J*_13_ = 8.0 Hz, 1H), 7.38 (d, *J* = 8.0 Hz, 1H), 7.02–6.98 (m, 1H), 6.92–6.88 (m, 1H), 6.79–6.71 (m, 3H), 6.65 (dd, *J*_12_ = 0.8 Hz, *J*_13_=8.0 Hz, 1H), 6.59 (t, *J* = 7.6 Hz, 1H), 6.41 (t, *J* = 0.8 Hz, 2H), 6.28–6.24 (m, 1H), 5.89–5.88 (m, 1H), 4.98 (s, 2H), 2.59 (s, 3H), 2.43–2.35 (m, 1H), 2.28–2.20 (m, 1H), 2.00–1.94 (m, 1H), 1.83–1.52 (m, 5H). ^13^C NMR (100 MHz, CDCl_3_): *δ* (ppm) 159.5, 154.8, 153.7, 146.1, 143.3, 143.0, 141.0, 140.3, 136.6, 131.4, 130.6, 129.3, 128.8, 128.3, 128.2, 128.0, 127.9 (2C), 127.5, 123.0, 122.9, 121.4, 120.9, 118.3, 117.1 (2C), 87.5, 82.2, 81.1, 53.1, 35.7, 34.6, 23.9, 23.7, 21.8. HRMS (ESI) calculated for C_35_H_29_N_3_O_2_ [M + H]^+^ 524.2338, found 524.2331.

#### 7.5.8. 8″-Methyl-2′-phenyl-3*a*′,9*b*′-dihydro-2′*H*-dispiro[cyclohexane-1,4′-chromeno[4,3-*d*]isoxazole-1′,11″-indeno[1,2-*b*]quinoxaline] (21h)

Yield (85%) as a pale yellow solid, M. P. = 200–202 °C, ^1^H NMR (400 MHz, CDCl_3_): *δ* (ppm) 8.22 (d, *J* = 8.8 Hz, 1H), 7.98 (s, 1H), 7.75 (d, *J* = 8.0 Hz, 1H), 7.64 (dd, *J*_12_ = 2 Hz, *J*_13_ = 8.8 Hz, 1H), 7.44 (d, *J* = 8.0 Hz, 1H), 7.09–7.05 (m, 1H), 7.00–6.96 (m, 1H), 6.88–6.77 (m, 4H), 6.66 (t, *J* = 7.6 Hz, 1H), 6.47 (t, *J* = 1.6 Hz, 2H), 6.34–6.30 (m, 1H), 5.94–5.93 (m, 1H), 5.05–499 (m, 2H), 2.66 (s, 3H), 2.44–2.36 (m, 1H), 2.08–1.96 (m, 2H), 1.80–1.67 (m, 3H), 1.53–1.42 (m, 3H), 1.28–1.24 (m, 1H). ^13^C NMR (100 MHz, CDCl_3_): *δ* (ppm) 159.5, 154.9, 152.5, 146.1, 143.3, 143.0, 141.0, 140.3, 136.6, 131.4, 130.6, 129.3, 128.7, 128.3, 128.0 (2C), 127.9 (2C), 127.4, 123.1, 122.9, 121.2, 120.9, 118.0, 117.1 (2C), 80.6, 80.6 (2C), 52.5, 32.9, 31.0, 25.5, 21.8, 21.3, 21.0. HRMS (ESI) calculated for C_36_H_31_N_3_O_2_ [M + H]^+^ 538.2494, found 538.2487.

#### 7.5.9. 8′-Chloro-4,4-dimethyl-2-phenyl-3*a*,9*b*-dihydro-2*H*,4*H*-spiro[chromeno[4,3-*d*]isoxazole-1,11′-indeno[1,2-*b*]quinoxaline] (21i)

Yield (87%) as a bright yellow solid, M. P. = 202–204 °C, ^1^H NMR (400 MHz, CDCl_3_): *δ* (ppm) 8.19 (d, *J* = 8.8 Hz, 1H), 8.12 (d, *J* = 2.4 Hz, 1H), 7.69–7.66 (m, 2H), 7.36 (d, *J* = 7.6 Hz, 1H), 7.05–7.01 (m, 1H), 6.96–6.91 (m, 1H), 6.82–6.72 (m, 3H), 6.67 (dd, *J*_12_ = 0.8 Hz, *J*_13_ = 7.6 Hz, 1H), 6.61 (t, *J* = 7.2 Hz, 1H), 6.40–6.36 (m, 2H), 6.29–6.25 (m, 1H), 5.87–5.85 (m, 1H) 4.93 (m, 2H), 1.76 (s, 3H), 1.22 (s, 3H). ^13^C NMR (100 MHz, CDCl_3_): *δ* (ppm) 160.6, 155.7, 153.1, 145.9, 143.5, 143.4, 140.3, 136.1, 131.2, 130.9, 130.0, 129.0, 128.2 (2C), 128.0 (3C), 127.5, 123.3,121.9, 121.4, 121.2, 118.1, 117.1 (3C), 81.3, 80.9, 76.2, 52.6, 25.0, 23.7. HRMS (ESI) calculated for C_32_H_24_ClN_3_O_2_ [M + H]^+^ 518.1635, found 518.1634.

#### 7.5.10. 8′-Chloro-4,4-diethyl-2-phenyl-3*a*,9*b*-dihydro-2*H*,4*H*-spiro[chromeno[4,3-*d*]isoxazole-1,11′-indeno[1,2-*b*]quinoxaline] (21j)

Yield (85%) as a straw yellow solid, M. P. = 178–180 °C, ^1^H NMR (400 MHz, CDCl_3_): *δ* (ppm) 8.20 (d, *J* = 9.2 Hz, 1H), 8.12 (d, *J* = 2.4 Hz,1H), 7.68 (dd, *J*_12_ = 2.8 Hz, *J*_13_ = 8.8 Hz, 2H), 7.39 (d, *J* = 7.6 Hz, 1H), 7.04–7.00 (m, 1H), 6.96–6.92 (m, 1H), 6.81–6.73 (m, 3H), 6.67 (d, *J* = 7.2 Hz, 1H), 6.61 (t, *J* = 7.2 Hz, 1H), 6.38 (d, *J* = 7.6 Hz, 2H), 6.25 (t, *J* = 7.6 Hz, 1H), 5.85–5.83 (m, 1H), 4.99–4.92 (m, 2H), 2.31–2.22 (m, 1H), 2.09–1.97 (m, 1H), 1.57–1.45 (m, 2H), 1.14–1.10 (t, *J* = 15.2 Hz, 3H), 0.77 (t, *J* = 7.2 Hz, 3H). ^13^C NMR (100 MHz, CDCl_3_): *δ* (ppm) 160.6, 155.7, 152.5, 145.9, 143.6, 143.5, 140.3, 136.2, 136.1, 131.2, 130.9, 130.1, 129.0, 128.3, 128.0 (2C), 127.8 (2C), 127.4, 123.3, 122.7, 121.3, 121.2, 118.1, 117.1 (2C), 81.3, 80.7, 79.1, 52.4, 25.4, 23.0, 8.3, 6.1. HRMS (ESI) calculated for C_34_H_28_ClN_3_O_2_ [M + H]^+^ 546.1948, found 546.1942.

#### 7.5.11. 8″-Chloro-2′-phenyl-3*a*′,9*b*′-dihydro-2′*H*-dispiro[cyclopentane-1,4′-chromeno[4,3-*d*]isoxazole-1′,11″-indeno[1,2-*b*]quinoxaline] (21k)

Yield (89%) as a bright yellow solid, M. P. = 206–208 °C, ^1^H NMR (400 MHz, CDCl_3_): *δ* (ppm) 8.19 (d, *J* = 8.8 Hz, 1H), 8.12 (d, *J* = 2.4 Hz, 1H), 7.69–7.66 (m, 2H), 7.38 (d, *J* = 8.0 Hz, 1H), 7.05–7.01 (m, 1H), 6.96–6.92 (m, 1H), 6.81–6.72 (m, 3H), 6.67 (dd, *J*_12_ = 0.8 Hz, *J*_13_ = 8.4 Hz, 1H), 6.61 (t, *J* = 7.2 Hz, 1H), 6.40 (t, *J* = 1.6 Hz, 2H), 6.30–6.26 (m, 1H), 5.88 (dd, *J*_12_ = 1.2 Hz, *J*_13_ = 7.2 Hz, 1H), 4.97 (s, 2H), 2.41–2.35 (m, 1H), 2.27–2.19 (m, 1H), 2.03–1.91 (m, 1H), 1.86–1.62 (m, 2H),1.61–1.53 (m, 3H). ^13^C NMR (100 MHz, CDCl_3_): *δ* (ppm) 160.7, 155.6, 153.7, 145.9, 143.5, 143.4, 140.3, 136.1, 136.1, 131.1, 130.9, 130.0, 129.0, 128.3, 128.2, 128.1, 128.0 (2C), 127.6, 123.2, 122.7, 121.5, 121.2, 118.4, 117.1 (2C), 87.5, 82.3, 81.1, 53.1, 35.7, 34.6, 23.9, 23.7. HRMS (ESI) calculated for C_34_H_26_ClN_3_O_2_ [M + H]^+^ 544.1792, found 544.1783.

#### 7.5.12. 8″-Methyl-2′-phenyl-3*a*′,9*b*′-dihydro-2′*H*-dispiro[cyclopentane-1,4′-chromeno[4,3-*d*]isoxazole-1′,11″-indeno[1,2-*b*]quinoxaline] (21l)

Yield (91%) as a pale yellow solid, M. P. = 195–197 °C, ^1^H NMR (400 MHz, CDCl_3_): *δ* (ppm) 8.19 (d, *J* = 8.8 Hz, 1H), 8.11 (d, *J* = 2.4 Hz, 1H), 7.69–7.66 (m, 2H), 7.37 (d, *J* = 8.0 Hz, 1H), 7.04–7.00 (m, 1H), 6.96–6.92 (m, 1H), 6.82–6.71 (m, 4H), 6.60 (t, *J* = 7.2 Hz, 1H), 6.38 (d, *J* = 7.6 Hz, 2H), 6.28–6.24 (m, 1H), 5.86 (t, *J* = 0.8 Hz, 1H), 4.96–4.91 (m, 2H), 2.36–2.28 (m, 1H), 2.00–1.83 (m, 2H), 1.72–1.61 (m, 3H), 1.47–1.35 (m, 4H). ^13^C NMR (100 MHz, CDCl_3_): *δ* (ppm) 160.7, 155.7, 152.5, 145.9, 143.6, 143.4, 140.3, 136.1, 136.1, 131.2, 130.9, 130.0, 128.9, 128.3, 128.2, 128.0 (3C), 127.5, 123.3, 122.7, 121.3, 121.2, 118.1, 117.2 (2C), 80.7, 80.6 (2C), 52.5, 32.9, 31.0, 25.5, 21.3, 21.0. HRMS (ESI) calculated for C_35_H_28_ClN_3_O_2_ [M + H]^+^ 558.1948, found 558.1946.

#### 7.5.13. 4,4,8-Trimethyl-2-phenyl-3*a*,9*b*-dihydro-2*H*,4*H*-spiro[chromeno[4,3-*d*]isoxazole-1,11′-indeno[1,2-*b*]quinoxaline] (21m)

Yield (88%) as a light yellow solid, M. P. = 185–187 °C, ^1^H NMR (400 MHz, CDCl_3_): *δ* (ppm) 8.29–8.27 (m, 1H), 8.16–8.13 (m, 1H), 7.80–7.72 (m, 3H), 7.31 (d, *J* = 8 Hz, 1H), 7.08–7.01 (m, 1H), 6.93–6.89 (m, 1H), 6.75–6.70 (m, 2H), 6.61–6.55 (m, 3H), 6.40–6.37 (m, 2H), 5.63 (s, 1H), 4.88 (s, 2H), 1.73 (s, 3H), 1.67 (s, 3H), 1.21 (s, 3H). ^13^C NMR (100 MHz, CDCl_3_): *δ* (ppm) 160.6, 154.9, 150.8, 146.0, 143.5, 142.9, 141.9, 136.5, 130.7, 130.5, 130.3, 129.9, 129.2 (2C), 128.9, 128.8, 128.4, 127.9 (2C), 127.5, 123.1, 121.7, 120.9, 117.7, 117.1 (2C), 81.3, 80.8, 75.9, 56.6, 25.0, 23.6, 20.1. HRMS (ESI) calculated for C_33_H_27_N_3_O_2_ [M + H]^+^ 498.2181, found 498.2199.

#### 7.5.14. 4,4,8,8′-Tetramethyl-2-phenyl-3*a*,9*b*-dihydro-2*H*,4*H*-spiro[chromeno[4,3-*d*]isoxazole-1,11′-indeno[1,2-*b*]quinoxaline] (21n)

Yield (84%) as a light yellow solid, M. P. = 176–178 °C, ^1^H NMR (400 MHz, CDCl_3_): *δ* (ppm) 8.16 (d, *J* = 8 Hz, 1H), 7.92 (s, 1H), 7.71 (d, *J* = 8 Hz, 1H), 7.57 (dd, *J*_12_ = 1.6 Hz, *J*_13_ = 8.8 Hz, 1H), 7.31 (d, *J* = 7.6 Hz, 1H), 7.04–7.00 (m, 1H), 6.92–6.88 (m, 1H), 6.74–6.70 (m, 2H), 6.61–6.54 (m, 3H), 6.39 (t, *J* = 1.2 Hz, 2H), 5.64 (s, 1H), 4.89 (s, 2H), 2.59 (s, 3H), 1.74 (s, 3H), 1.67 (s, 3H), 1.21 (s, 3H). ^13^C NMR (100 MHz, CDCl_3_): *δ* (ppm) 159.6, 154.8, 150.8, 146.1, 143.4, 143.0, 141.0, 140.3, 136.6, 131.4, 130.5 (2C), 129.3, 128.8, 128.7, 128.4, 128.3, 127.9 (2C), 127.4, 123.0, 121.7, 120.7, 117.6, 117.1 (2C), 81.3, 80.8, 75.9, 52.4, 25.0, 23.6, 21.8, 20.1. HRMS (ESI) calculated for C_34_H_29_ClN_3_O_2_ [M + H]^+^ 512.2338, found 512.2337.

#### 7.5.15. 8′-Chloro-4,4,8-trimethyl-2-phenyl-3*a*,9*b*-dihydro-2*H*,4*H*-spiro[chromeno[4,3-*d*]isoxazole-1,11′-indeno[1,2-*b*]quinoxaline] (21o)

Yield (90%) as a bright yellow solid, M. P. = 200–202 °C, ^1^H NMR (400 MHz, CDCl_3_): *δ* (ppm) 8.19 (d, *J* = 8.8 Hz, 1H), 8.12 (d, *J* = 2 Hz, 1H), 7.72–7.66 (m, 2H), 7.30 (d, *J* = 8 Hz, 1H), 7.06–7.02 (m, 1H), 6.95–6.91 (m, 1H), 6.75–6.71 (m, 2H), 6.62–6.55 (m, 3H), 6.39–6.36 (m, 2H),5.62 (d, *J* = 1.2 Hz, 1H), 4.87 (s, 2H), 1.74 (s, 3H), 1.68 (s, 3H), 1.21 (s, 3H). ^13^C NMR (100 MHz,CDCl_3_): *δ* (ppm) 160.9, 155.7, 150.9, 146.0, 143.7, 143.5,1 40.4, 136.2, 136.1, 131.1, 130.9, 130.6, 130.0, 129.0, 128.9, 128.4, 128.3, 128.0 (2C), 127.6, 123.2, 121.5, 121.1, 117.7, 117.2 (2C), 81.4, 80.8, 75.9, 52.6, 25.0, 23.6, 21.0. HRMS (ESI) calculated for C_33_H_26_ClN_3_O_2_ [M + H]^+^ 532.1792, found 532.1785.

#### 7.5.16. 8′-Methyl-2′-phenyl-3*a*′,9*b*′-dihydro-2′*H*-dispiro[cyclohexane-1,4′-chromeno[4,3-*d*]isoxazole-1′,11″-indeno[1,2-*b*]quinoxaline] (21p)

Yield (91%) as a straw yellow solid, M. P. = 180–182 °C, ^1^H NMR (400 MHz, CDCl_3_): *δ* (ppm) 8.29–8.27 (m, 1H), 8.16–8.13 (m, 1H), 7.80–7.72 (m, 3H), 7.33 (d, *J* = 8.0 Hz, 1H), 7.05–7.01 (m, 1H), 6.94–6.90 (m, 1H), 6.74–6.68 (m, 2H), 6.63–6.57 (m, 3H), 6.39 (t, *J* = 1.2 Hz, 2H), 5.64 (s, 1H), 4.93–4.87 (m, 2H), 2.34–2.10 (m, 1H), 1.97–1.90 (m, 2H), 1.68–1.61 (m, 5H), 1.48–1.32 (m, 5H). ^13^C NMR (100 MHz, CDCl_3_): *δ* (ppm) 160.8, 154.9, 150.3, 146.1, 143.6, 143.0, 141.9, 136.5, 130.7, 130.5, 130.3, 129.9, 129.2 (2C), 128.8, 128.7, 128.5, 127.9 (3C), 127.5, 123.1, 122.4, 120.8, 117.7, 117.1 (2C), 80.6 (2C), 52.5, 33.0, 30.8, 25.5, 21.3, 21.0, 20.1. HRMS (ESI) calculated for C_36_H_31_N_3_O_2_ [M + H]^+^ 538.2494, found 538.2497.

#### 7.5.17. 8′,8″-Dimethyl-2′-phenyl-3*a*′,9*b*′-dihydro-2′*H*-dispiro[cyclohexane-1,4′-chromeno[4,3-*d*]isoxazole-1′,11″-indeno[1,2-*b*]quinoxaline] (21q)

Yield (87%) as a yellow solid, M. P. = 173–175 °C, ^1^H NMR (400 MHz, CDCl_3_): *δ* (ppm) 8.16 (d, *J* = 18 Hz, 1H), 7.92 (s, 1H), 7.70 (d, *J* = 7.2 Hz, 1H), 7.57 (dd, *J*_12_ = 2 Hz, *J*_13_=8.8 Hz, 1H), 7.33 (d, *J* = 7.6 Hz, 1H), 7.03–6.99 (m, 1H), 6.92–6.88 (m, 1H), 6.71 (t, *J* = 8 Hz, 2H), 6.62–6.57 (m, 3H), 6.37 (d, *J* = 8 Hz, 2H), 5.64 (s, 1H), 4.92–4.96 (m, 2H), 2.59 (s, 3H), 2.35–2.29 (m, 1H), 1.97–1.90 (m, 2H), 1.67 (s, 6H), 1.45–1.33 (m, 4H). ^13^C NMR (100 MHz, CDCl_3_): *δ* (ppm) 159.7, 154.9, 150.2, 146.1, 143.5, 143.0, 140.9, 140.3, 136.6, 131.3, 130.6, 130.5, 129.4, 128.7, 128.6, 128.4, 128.3, 127.9 (3C), 127.5, 123.0, 122.5, 120.7, 117.7, 117.1 (3C), 80.6, 52.4, 33.0, 30.8, 25.5, 21.8, 21.3, 21.0, 20.1. HRMS (ESI) calculated for C_37_H_33_N_3_O_2_ [M + H]^+^ 552.2651, found 552.2658.

#### 7.5.18. 8″-Chloro-8′-methyl-2′-phenyl-3*a*′,9*b*′-dihydro-2′*H*-dispiro[cyclohexane-1,4′-chromeno[4,3-*d*]isoxazole-1′,11″-indeno[1,2-*b*]quinoxaline] (21r)

Yield (93%) as a bright yellow solid, M. P. = 182–184 °C, ^1^H NMR (400 MHz, CDCl_3_): *δ* (ppm) 8.19 (d, *J* = 9.2 Hz, 1H), 8.12 (d, *J* = 2. Hz, 1H), 7.17–7.66 (m, 2H), 7.33 (d, *J* = 7.6 Hz, 1H), 7.05–7.01 (m, 1H), 6.96–6.92 (m, 1H), 6.75–6.71 (m, 2H), 6.63–6.58 (m, 3H), 6.38 (t, *J* = 1.2 Hz, 2H), 5.63 (s, 1H), 4.91–4.84 (m, 2H), 2.35–2.29 (m, 1H), 2.19–2.09 (m, 1H), 1.97–1.89 (m, 2H), 1.68–1.61 (m, 5H), 1.47–1.34 (m, 4H). ^13^C NMR (100 MHz, CDCl_3_): *δ* (ppm) 160.9, 155.7, 150.7, 146.0, 143.7, 143.4, 140.3, 136.1, 131.1, 130.9, 130.5, 130.0, 128.9, 128.8, 128.4, 128.3, 128.0 (3C), 127.6, 123.2, 122.2, 121.1, 117.8, 117.2 (3C), 80.6 (2C), 52.5, 33.0, 30.8, 25.5, 21.3, 21.0, 20.1. HRMS (ESI) calculated for C_36_H_30_ClN_3_O_2_ [M + H]^+^ 572.2105, found 572.2096.

## 8. Method of crystal growth

Compound 21c was initially dissolved in a minimal amount of CDCl_3_, followed by the addition of drops of hexane. This solution was then placed in a sample vial and stored in a dark area for 4 to 5 weeks to enable gradual evaporation. After 5 weeks, pale yellow coloured needle-like crystals of compound 21c were formed.

## 9. Anticancer assessment of compound 21(a–r)

All the cell lines (MDA MB-468, MDA MB-231, MCF-7, and HEK-293) were procured from IICT, Hyderabad, India. The cell proliferation was evaluated using the MTT colorimetric assay. The cells were seeded in 100 µL DMEM, supplemented with 10% FBS in each well of 96-well microculture plates at a density of 1 × 10^4^ cells per well and incubated for 24 h at 37 °C in a CO_2_ humidified incubator. Following the initial incubation, the synthesized compounds were added to the respective wells and the cells were further incubated for 48 h. After drug treatment, 10 µL of MTT solution (3-(4,5-dimethylthiazol-2-yl)-2,5-diphenyl tetrazolium bromide; 5 mg mL^−1^) was added to each well, and the plates were incubated for an additional 4 h to allow for formazan formation. The culture medium was then carefully removed, and the resulting formazan crystals were solubilized in 100 µL of DMSO. Absorbance was measured at 570 nm using a microplate reader (BioTek, Winooski, USA). The cell viability was calculated as percent cell viability, using the following formula:% of cell viability = [*A*_1_ − *A*_0_/*A*_u_ − *A*_0_] × 100where *A*_1_ is the absorbance of the treated cells, *A*_0_ is the absorbance of the blank cells, and *A*_u_ is the absorbance of the untreated cells.

The IC_50_ value represents the concentration of the derivative that results in 50% cell death. The anticancer activity of the synthesized compound and standard drug were evaluated by calculating the IC_50_ using the GraphPad Prism software (version 9.0). All experiments were performed in triplicate, and results are presented as mean ± standard deviation (*n* = 3).^31^

## 10. *In silico* molecular docking studies

*In silico* molecular studies of synthesized compounds and standard drug were performed against the epidermal growth factor receptor (EGFR) tyrosine kinase protein^55^ and E3 ubiquitin ligase tumor suppressor p53 regulator (MDM2) protein^56^ using AutoDock Vina software.^33,57,58^ In this study, the target proteins were identified by employing a disease-focused approach supported by extensive literature screening and data-mining analyses across relevant biological databases.^34,57^. The crystal structures of EGFR protein (PDB ID: 4HJO) with the resolution of 2.75 Å and MDM2 protein (PDB ID: 5LAV) with the resolution of 1.73 Å, were obtained from RCSB protein data bank (https://www.rcsb.org) in “.pdb” format. Then, retrieved protein underwent a process where water molecules and heteroatoms were removed and polar hydrogens were added to it. The ligand structures were designed using Chem3D tool of PerkinElmer ChemDraw professional 19.0 software and exported in MDL format, followed by conversion to “.pdb” format using PyMOL software.^58^ Subsequently, molecular docking was executed in AutoDock Vina employing the Lamarckian Genetic Algorithm (LGA)^33^ and all generated interaction profiles were analyzed and visualized through BIOVIA Discovery Studio 2025 software.^59^

## 11. Antibacterial assessment of compound 21(a–r) against bacterial DNA gyrase of *E. coli* and *S. aureus*

The *in vitro* antibacterial assessment was performed using the agar well diffusion method against *E. coli* and *S. aureus*, considering Gentamicin as the standard drug. The compounds were dissolved in DMSO for the agar well diffusion method with a concentration of 20 µg mL^−1^ on a nutrient agar plate and then incubated for 24 hours at 37 °C. After incubation, zones of inhibition (ZI) were measured and tabulated. For the determination of the minimum inhibitory concentration (MIC), a 96-well plate (Hi-Media) was used. For MIC, the compound concentration was 20 µg mL^−1^, which was then serially diluted and incubated at 37 °C for 24 hours. These two methods were employed to evaluate the bacterial inhibitory properties, using methods in accordance with the standard protocols of the CLSI Guidelines.^60^

## 12. Molecular docking study of compound 21(a–r) against bacterial DNA gyrase of *E. coli* and *S. aureus*

*In silico* studies of compounds 21(a–r) were performed with the DNA gyrase inhibitor of the Gram-positive bacterial strain *S. aureus* and another Gram-negative bacterial strain *E. coli*. Both these bacterial strains were known for causing notorious nosocomial infections. The crystal structures of *E. coli* (PDB ID: 1KZN) and *S. aureus* (PDB ID: 3 G7B) were retrieved from RCSB protein bank in (.pdb) format. After retrieving the heteroatoms, water molecules were removed by using PyMOL. AutoDock was used for the determination of binding interactions in molecular docking studies, and BIOVIA Discovery Studio v2025 was used for visualizing intermolecular binding interactions of docked complexes and to generate the 2D and 3D structures of the docked complexes of the tested compounds.^61^

## 13. Determination of physicochemical, pharmacokinetic, and ADME properties

The physicochemical, medicinal chemistry and ADME (Absorption, Distribution, Metabolism and Excretion) properties of four potent compounds (21k, 21b, 21j, and 21r) and two standard drugs (Gentamicin and Doxorubicin) were predicted by using web-based analytical tools, including SwissADME (https://www.swissadme.ch/; last accessed on 25^th^ November 2025)^62^ and ADMETlab 3.0 (https://admetlab3.scbdd.com/; last accessed on 25^th^ November 2025).^63^ The toxicity properties were predicted by web-based softwares pkCSM (https://biosig.lab.uq.edu.au/pkcsm/; last accessed on 27^th^ November 2025)^64^ and Pro Tox 3.0 (https://tox.charite.de/protox3/index.php?; last accessed on 27^th^ November 2025).^65^

## 14. Photophysical and metal sensing study

The photophysical (UV-visible and fluorescence) and metal sensing studies were performed for all the synthesized compounds using the Shimadzu single monochromator UV-2600 spectrophotometer and HITACHI F-7000 fluorescence spectrometer. Initially, the compounds were dissolved in DMSO at a uniform concentration (20 µm), and the spectra were recorded.^43–54^^.^

## Conflicts of interest

The authors declare no conflict of interest.

## Supplementary Material

RA-016-D6RA01808D-s001

RA-016-D6RA01808D-s002

## Data Availability

CCDC 2480886 (21c) contains the supplementary crystallographic data for this paper.^66^ Spectral data for new compounds 21(a–r) are available in the supplementary information (SI). Supplementary information: experimental procedures, characterization data, copies of NMR and HRMS spectra, molecular docking studies, photophysical studies, and crystallographic data. See DOI: https://doi.org/10.1039/d6ra01808d.

## References

[cit1] Arumugam N., Soliman S. M., Viswanathan V., Almansour A. I., Kumar R. S., Mahalingam S. M., Krishnamoorthy B. S., Dege N., Karuppiah P., Perumal K. (2023). J. Mol. Struct..

[cit2] Marson C. M. (2011). Chem. Soc. Rev..

[cit3] Zheng Y. C., Tice M., Singh S. B. (2014). Bioorg. Med. Chem. Lett..

[cit4] Kotha S., Panguluri N. R., Ali R. (2017). Eur. J. Org. Chem..

[cit5] Redkin R. G., Syumka E. I., Shemchuk L. A., Chernykh V. P. (2017). J. Appl. Pharm. Sci..

[cit6] Almansour A. I., Arumugam N., Kumar R. S., Al-thamili D. M., Periyasami G., Ponmurugan K., Al-Dhabi N. A., Perumal K., Premnath D. (2019). Molecules.

[cit7] El-Sharief A. M. S., Ammar Y. A., Belal A., El-Sharief M. A. M. S., Mohamed Y. A., Mehany A. B. M., Elhag Ali G. A. M., Ragab A. (2019). Bioorg. Chem..

[cit8] Lotfy G., El Sayed H., Said M. M., Aziz Y. M. A., Al-Dhfyan A., Al-Majid A. M., Barakat A. (2018). J. Photochem. Photobiol., B.

[cit9] Bouhenna M. M., Orlikova B., Talhi O., Schram B., Pinto D. C. G. A., Taibi N., Bachari K., Diederich M., Silva A. M. S., Mameri N. (2017). Anticancer Res..

[cit10] Raju S. K., Almansour A. I., Natarajan A., Mohammad F. (2019). Molecules.

[cit11] Galvez J., Polo S., Insuasty B., Gutierrez M., Cáceres D., Alzate-Morales J. H., De-la-Torre P., Quiroga J. (2018). Comput. Biol. Chem..

[cit12] Arumugam N., Almansour A. I., Kumar R. S., Kotresha D., Saiswaroop R., Venketesh S. (2019). Bioorg. Med. Chem..

[cit13] Bouali N., Hammouda M. B., Ahmad I., Ghannay S., Thouri A., Dbeibia A., Patel H., Hamadou W. S., Hosni K., Snoussi M., Adnan M., Hassan M. I., Noumi E., Aouadi K., Kadri A. (2022). Molecules.

[cit14] Tseng C. H., Chen Y. R., Tzeng C. C., Liu W., Chou C. K., Chiu C. C., Chen Y. L. (2016). Eur. J. Med. Chem..

[cit15] Tseng C. H., Tzeng C. C., Yang C. L., Lu P. J., Chen H. L., Li H. Y., Chuang Y. C., Yang C. N., Chen Y. L. (2010). J. Med. Chem..

[cit16] Schepetkin I. A., Khlebnikov A. I., Potapov A. S., Kovrizhina A. R., Matveevskaya V. V., Belyanin M. L., Atochin D. N., Zanoza S. O., Gaidarzhy N. M., Lyakhov S. A., Kirpotina L. N., Quinn M. T. (2019). Eur. J. Med. Chem..

[cit17] Asif M., Alvi S. S., Azaz T., Khan A. R., Tiwari B., Hafeez B. B., Nasibullah M. (2023). Int. J. Mol. Sci..

[cit18] Zimnitskiy N. S., Barkov A. Y., Ulitko M. V., Kutyashev I. B., Korotaev V. Y., Sosnovskikh V. Y. (2020). New J. Chem..

[cit19] Banerjee B., Sharma A., Kaur M., Singh A., Priya A. (2024). ChemistrySelect.

[cit20] Singh R., Bhardwaj D., Saini M. R. (2021). RSC Adv..

[cit21] Alayyaf A. A., Alamary A. S., Islam M. S., Ali M., Barakat A., Bari A., Domingo L. R., Darwish K. M., Khamis M. A., Emara M., Elshamy A. M., Mirgany T. O., Nafie M. S. (2026). Chem. Biodiversity.

[cit22] Zimnitskiy N. S., Barkov A. Y., Ulitko M. V., Kutyashev I. B., Korotaev V. Y., Sosnovskikh V. Y. (2020). New J. Chem..

[cit23] Kumar N., Inwati G. K., Ahmed E. M., Lal C., Makwana B., Yadav V. K., Islam S., Ahn H. J., Yadav K. K., Jeon B. H. (2022). Catalysts.

[cit24] Barakat A., Alshahrani S., Al-Majid A. M., Alamary A. S., Haukka M., Abu-Serie M. M., Domingo L. R., Ashraf S., Ul-Haq Z., Nafie M. S., Teleb M. (2023). J. Enzyme Inhib. Med. Chem..

[cit25] Kanchrana M., Gamidi R. K., Kumari J., Sriram D., Basavoju S. (2024). Mol. Diversity.

[cit26] Lavanya M., Sundararaj R., Mani K. S., Balu K., Durai M., Alam P., Priya S. D., Durai M., Ahn Y. H. (2025). ChemistrySelect.

[cit27] Wang Q., She X., Ren X., Ma J., Pan X. (2004). Tetrahedron:Asymmetry.

[cit28] Ashok D., Kumar R. S., Gandhi D. M., Jayashree A. (2016). Russ. J. Chem..

[cit29] Ashok D., Kumar R. S., Gandhi D. M., Sarasija M., Jayashree A., Adam S. (2016). Heterocycl. Commun..

[cit30] Mohapatra S., Parida S. P., Nayak S., Behera K., Mohapatra S. (2025). Asian J. Chem..

[cit31] Meerloo J. V., Kaspers G. J., Cloos J. (2011). Cell sensitivity assays: the MTT assay. Methods Mol. Biol..

[cit32] Panda B. S., Samanta B., Sudha Ambadipudi S. S. S. S., Nayak S., Nayak V. L., Ramakrishna S., Mohapatra S., Behera P. M., Samanta L. (2024). ChemistrySelect.

[cit33] Panda B. S., Ahemad M. A., Mohapatra S., Naik E., Nayak S., Mohapatra S., Naik P. K., Bhattacharya D., Sahoo C. R., Sahoo M. K. (2024). J. Mol. Struct..

[cit34] Panda B. S., Samanta B., Naik E., Nayak S., Pragyandipta P., Mohapatra S., Naik P. K. (2025). J. Mol. Struct..

[cit35] Kralj S. , Jukic M. and Bren U., Encyclopedia, 2023, vol. 3, pp. 501–511, 10.3390/encyclopedia3020035

[cit36] Belay Y., Muller A., Mokoena F. S., Adeyinka A. S., Motadi L. R., Oyebamiji A. K. (2024). Sci. Rep..

[cit37] Reddy T. S., Rai S., Koppula S. K. (2022). J. Mol. Struct..

[cit38] Ruswanto R., Miftah A. M., Tjahjono D. H., Siswandono (2021). Chem. Data Collect..

[cit39] Xavier M. R., Marinho M. M., Juliao M. S. S., Marinho E. S., Almeida-Neto F. W. Q., deCastro K. K. A., Hora J. P. D., Rocha M. N. D., Barreto A. C. H., Saraiva G. D., Bandeira P. N., Araujo I. M., Coutinho H. D. M., dos Santos H. S., Teixeira A. M. R. (2024). J. Mol. Struct..

[cit40] Park S. J., Song I. H., Yeom G. S., Nimse S. B. (2024). Biomed. Pharmacother..

[cit41] Panigrahi G., Sudha Ambadipudi S. S. S. S., Ahemad M. A., Panda B. S., Mohapatra S., Prusty K., Nayak S., Mohapatra S., Nayak V. L., Andugulapati S. B. (2025). ChemistrySelect.

[cit42] Abo-Salem H. M., Souda S. S. M. E., Shafey H. I., Zoheir K. M. A., Ahmed K. M., Mahmoud K., Mahrous K. F., Fawzy N. M. (2024). Sci. Rep..

[cit43] Parida S. P., Mohapatra S., Mohapatra S., Behera T., Nayak S., Sahoo C. R. (2025). RSC Adv..

[cit44] Behera T., Sethi S., Rout J., Bag B. P., Behera N. (2024). Phys. Chem. Chem. Phys..

[cit45] Mohapatra S., Pradhan A., Parida S. P., Behera T., Sahoo S., Mohapatra S., Nayak S., Samanta L. (2025). ChemistrySelect.

[cit46] Behera T., Rout J., Bhoi N., Mallik S., Nag S., Sethi S., Pragyandipta P., Nanda P. K., Naik P. K., Behera N. (2025). ACS Appl. Bio Mater..

[cit47] Behera T., Sarangi B., Mishra D., Pattnaik S., Parhi P., Behera N. (2024). ChemistrySelect.

[cit48] Tamilselvan S., Soniya R. M., Vasantharaja R., Kannan M., Supriya S., Batvari B. P. D., Ramesh T., Govindaraju K. (2022). Environ. Res..

[cit49] Shrivas K., Sahu B., Deb M. K., Thakur S. S., Sahu S., Kurrey R., Kant T., Patle T. K., Jangde R. (2019). Microchem. J..

[cit50] Firdaus M. L., Fitriani I., Wyantuti S., Hartati Y. W., Khaydarov R., Mcalister J. A., Obata H., Gamo T. (2017). Anal. Sci..

[cit51] Taufiq M., Eden W. T., Sumarni W., Alauhdin M. (2021). J. Phys.:Conf. Ser..

[cit52] Lee S., Nam Y. S., Lee H. J., Lee Y., Lee K. B. (2016). Sens. Actuators, B.

[cit53] Zwolak A., Sarzyńska M., Szpyrka E., Stawarczyk K. (2019). Water, Air, Soil Pollut..

[cit54] Musikavanhu B., Zhang Y., Zhu D., Xue Z., Yuan R., Wang S., Zhao L. (2022). Spectrochim. Acta, Part A.

[cit55] Atmaca H., Ilhan S., Çamli Pulat C., Dundar B. A., Zora M. (2024). ACS Omega.

[cit56] Gouda M., Bawzeer M., Hegazy L., Azab M., Elagawany M., Rateb M., Yaseen M., Elgendy B. (2022). Curr. Pharm. Des..

[cit57] Eberhardt J., Santos-Martins D., Tillack A. F., Forli S. (2021). J. Chem. Inf. Model..

[cit58] Morris G. M., Goodsell D. S., Halliday R. S., Huey R., Hart W. E., Belew R. K., Olson A. J. (1998). J. Comput. Chem..

[cit59] D. Studio , Discov. Studio, Accelrys [2.1], 2008, vol. 420, pp. 1–9

[cit60] Chandrasekaran B. , Abed S. N., Al-Attraqchi O., Kuche K. and Tekade R. K., Dosage Form Design Parameters, Academic Press, Elsevier, 2018, vol. 2, pp. 731–755, 10.1016/B978-0-12-814421-3.00021-X

[cit61] Ivanovic V., Rancic M., Arsic B., Pavlovic A. (2020). Chem. Naiss..

[cit62] Daina A., Michielin O., Zoete V. (2017). Sci. Rep..

[cit63] Fu L., Shi S., Yi J., Wang N., He Y., Wu Z., Peng J., Deng Y., Wang W., Wu C., Lyu A., Zeng X., Zhao W., Hou T., Cao D. (2024). Nucleic Acids Res..

[cit64] Pires D. E. V., Blundell T. L., Ascher D. B. (2015). J. Med. Chem..

[cit65] Banerjee P., Eckert A. O., Schrey A. K., Preissner R. (2018). Nucleic Acids Res..

[cit66] CCDC 2480886: Experimental Crystal Structure Determination, 2026, 10.5517/ccdc.csd.cc2p8kl4

