# Supplementary material for: Design, synthesis, and biological evaluation of multifunctional dispiro chromeno-indenoquinoxaline hybrids as dual anticancer and antibacterial agents with metal-ion sensing ability
Source: RSC Adv. 2026 May 19;16(29):26805–31. doi: 10.1039/d6ra01808d (PMC13187895; doi:10.1039/d6ra01808d)
Supplement: RA-016-D6RA01808D-s001 [file RA-016-D6RA01808D-s001.pdf]

Supplementary Information (SI) for RSC Advances.
This journal is © The Royal Society of Chemistry 2026

Design, Synthesis, and Biological Evaluation of Multifunctional Dispiro Chromeno-Indenoquinoxaline Hybrids as Dual Anticancer and Antibacterial Agents with Metal-Ion Sensing Ability

Kamalika Prusty[a], S.S.S.S Sudha Ambadipudi[b], Suhasini Mohapatra[a], Tankadhara Behera[d], Bhabani shankar Panda[a], Gopinatha Panigrahi[a], Sabita Nayak*[a], Seetaram Mohapatra[a], Akankhya Mohanty[c,e], Jyotiprabha Rout[d], Chitta Ranjan Sahoo[c], V. Lakhsma Nayak[b]

[a] *Organic Synthesis Laboratory, Department of Chemistry, Ravenshaw University, Cuttack 753003, Odisha, India*

[b] *Applied Biology Department, CSIR- Indian Institute of Chemical Technology, Hyderabad-500007, India*

[c] *ICMR-Regional Medical Research Centre, Department of Health Research, Ministry of Health & Family Welfare, Govt. of India, Bhubaneswar 751023, Odisha, India*

[d] *School of Chemistry, Sambalpur University, Jyoti Vihar, Sambalpur 768019, Odisha, India*

[e] *Siksha O Anusandhan, Deemed to be University, Kalinga Nagar, Ghatikia, Bhubaneswar, Odisha,751003, India*

Prof. Sabita Nayak, Department of Chemistry, Ravenshaw University, Cuttack-753003, Odisha, India,
Email: sabitanayak18@gmail.com (*Corresponding author)

Supporting Information

Table of contents

1. **Characterisation Data**
2. **^{1}H NMR, ^{13}C NMR & HRMS data**
3. **Crystallographic data for 21c**
4. **Molecular docking analysis of synthesized compounds and standard drug with anticancer target protein**
5. **Molecular docking analysis of synthesized compounds with bacterial DNA gyrase**
6. **Physicochemical properties, medicinal chemistry properties and physicochemical properties-based drug likeness rules, bioavailability score and drug-likeness model score of the four potent compounds data**
7. **ADMET properties of the four potent compounds data**
8. **DFT studies**
9. **References**

Characterization Data.

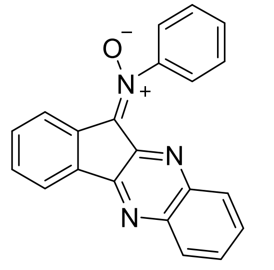

15a

(E)-N-phenyl-11H-indeno[1,2-b]quinoxalin-11-imine oxide (15a) Yield (87%) as a yellow solid, M.P. = 192-194 °C, ^{1}H NMR (400MHz, $CDCl_3$): δ (ppm) 9.00-8.97 (m, 1H), 8.15-8.12 (m, 1H), 8.24-8.21(m, 1H), 7.64-7.56 (m, 3H), 7.54- 7.47 (m, 6H), 7.45- 7.43 (m, 1H).^{13}C NMR (100MHz, $CDCl_3$): δ (ppm) 151.1, 147.5, 145.6, 141.0, 140.7, 139.8, 135.1, 134.4, 132.4, 131.9, 130.3, 130.2, 130.1, 129.2, 128.8 (2C), 128.8, 126.3, 124.4(2C), 122.4.

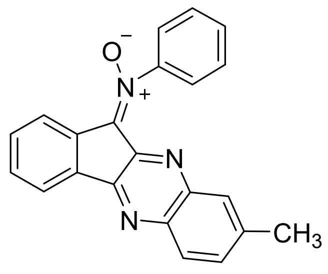

15b

(E)-8-methyl-N-phenyl-11H-indeno[1,2-b]quinoxalin-11-imine oxide (15b) Yield (85%) as a yellow solid, M.P. = 212-214 °C, ^{1}H NMR (400MHz, $CDCl_3$): δ (ppm) 9.08-9.04 (m, 1H), 8.24-8.16 (m, 1H), 7.83(s, 1H), 7.69-7.67 (m, 2H), 7.63- 7.54 (m, 5H), 7.41-7.36 (m, 2H). ^{13}C NMR (100MHz, $CDCl_3$): δ (ppm) 151.6, 147.4, 144.8, 141.3, 141.0, 140.1,139.4, 134.9, 132.0, 131.9, 131.3, 130.2, 129.6, 128.8 (2C), 128.2, 126.3, 124.4(2C), 121.9, 21.8.

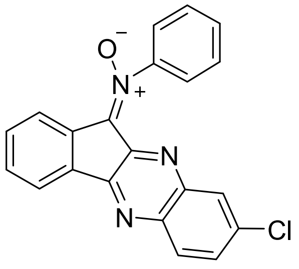

15c

(E)-8-chloro-N-phenyl-11H-indeno[1,2-b]quinoxalin-11-imine oxide (15c) Yield (82%) as a yellow solid, M.P. = 189-191 °C, ^{1}H NMR (400MHz, $CDCl_3$): δ (ppm) 8.98 (d, J = 5.6Hz, 1H), 8.16(d, J = 5.2Hz, 1H), 7.97 (d, J = 1.2Hz, 1H), 7.63(t, J = 3.6Hz, 2H), 7.57-7.50 (m, 5H), 7.43-7.34 (m, 2H). ^{13}C NMR (100MHz, $CDCl_3$): δ (ppm) 152.2, 147.4, 145.5, 141.6,139.6, 139.4, 135.8, 135.2, 134.2, 132.6, 131.9, 131.1, 130.3, 129.9, 128.9 (2C), 128.1, 126.3, 124.3(2C), 122.3.

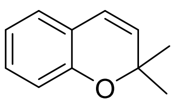

20a

2,2-dimethyl-2H-chromene (20a). Yield (85%) as a liquid, ^{1}H NMR (400MHz, $CDCl_3$): δ (ppm) 7.15-7.11 (m, 1H), 7.00 (dd, J_{12} = 2Hz, J_{13} = 7.2Hz, 1H), 6.87 (dd, J_{12} = 9.2Hz, J_{13} = 7.2Hz, 1H), 6.84- 6.80 (m, 1H), 6.34 (d, J = 9.6Hz, 1H), 5.62 (d, J = 10Hz, 1H), 1.46(s, 6H). ^{13}C NMR (100MHz, $CDCl_3$): δ (ppm) 152.8, 130.5, 128.9, 126.2, 122.2, 121.1, 120.6, 116.2, 75.9, 27.9(2C).

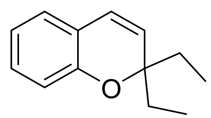

20b

2,2-diethyl-2H-chromene (20b). Yield (84%) as a liquid, ^{1}H NMR (400MHz, $CDCl_3$): δ (ppm) 7.19-7.12 (m, 1H), 7.00-6.98(m, 1H), 6.87-6.83(m, 2H), 6.47 (d, *J* = 10Hz, 1H), 5.51 (d, *J* = 10Hz, 1H),1.88- 1.79 (s, 6H),1.75- 1.66 (m, 2H), 1.04 (t, *J* = 8Hz, 6H), ^{13}C NMR (100MHz, $CDCl_3$): δ (ppm) 153.9, 128.9, 127.9, 126.2, 123.7, 120.8, 120.0, 115.5, 81.6, 32.3(2C), 7.9(2C).

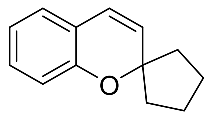

20c

spiro[chromene-2,1'-cyclopentane] (20c). Yield (87%) as a liquid, ^{1}H NMR (400MHz, $CDCl_3$): δ (ppm) 7.00-6.95(m, 1H), 6.85 (d, *J* = 7.2Hz, 1H), 6.74- 6.70 (m, 1H), 6.65 (d, *J* = 8Hz, 1H), 6.24 (d, *J* = 10Hz, 1H), 5.53 (d, *J* = 10Hz, 1H), 2.09- 2.03 (m, 2H),1.86- 1.76 (m, 2H), 1.63- 1.55 (m, 2H),1.51- 1.44 (m, 2H). ^{13}C NMR (100MHz, $CDCl_3$): δ (ppm) 152.9, 129.7, 128.7, 126.1, 123.0, 121.9, 120.6, 116.2, 86.9, 39.3(3C), 23.5(2C).

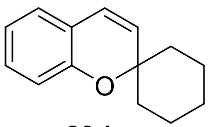

20d

spiro[chromene-2,1'-cyclohexane] (20d). Yield (88%) as a liquid, ^{1}H NMR (400MHz, $CDCl_3$): δ (ppm) 6.99-6.55(m, 1H), 6.82-6.80(m, 1H), 6.70-6.66 (m, 2H), 6.18 (d, *J* = 10.4Hz, 1H), 5.47(d, *J* = 10Hz, 1H), 1.89- 1.80 (m, 2H), 1.69- 1.59 (m, 2H), 1.49- 1.35 (m, 5H), 1.24- 1.09 (m, 1H). ^{13}C NMR (100MHz, $CDCl_3$): δ (ppm) 152.7, 130.3, 128.8, 126.1,122.6, 122.0, 120.5, 116.3, 76.5, 35.8, 25.2, 21.2.

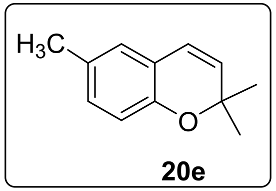

2,2,6-trimethyl-2H-chromene (20e). Yield (86%) as a liquid, ^{1}H NMR (400MHz, $CDCl_3$): δ (ppm) 7.06-7.03(m, 1H), 6.92 (d, *J* = 2Hz, 1H), 6.85 (d, *J* = 8Hz, 1H), 6.41 (d, *J* = 9.6Hz, 1H), 5.71(d, *J* = 10Hz, 1H), 2.39 (s, 3H), 1.57(s, 6H). ^{13}C NMR (100MHz, $CDCl_3$): δ (ppm) 150.5, 130.6, 129.5, 129.3, 126.6, 122.3, 120.9, 115.9, 75.6, 27.7(2C), 20.3.

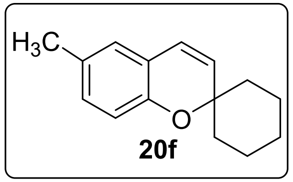

6-methylspiro[chromene-2,1'-cyclohexane] (20f). Yield (87%) as a liquid, ^{1}H NMR (400MHz, $CDCl_3$): δ (ppm) 7.03 (d, *J* = 8Hz, 1H), 6.90-6.87(m, 2H), 6.42 (d, *J* = 10Hz, 1H), 5.73(d, *J* = 10Hz, 1H), 2.38 (s, 3H), 2.10- 2.07 (m, 3H), 1.96- 1.87 (m, 3H), 1.67- 1.60 (m, 3H), 1.52- 1.37 (m, 1H). ^{13}C NMR (100MHz, $CDCl_3$): δ (ppm) 150.5, 130.4, 129.4, 129.2, 126.6, 122.7, 121.8, 116.0, 76.2, 35.6, 25.2, 21.2, 20.3.

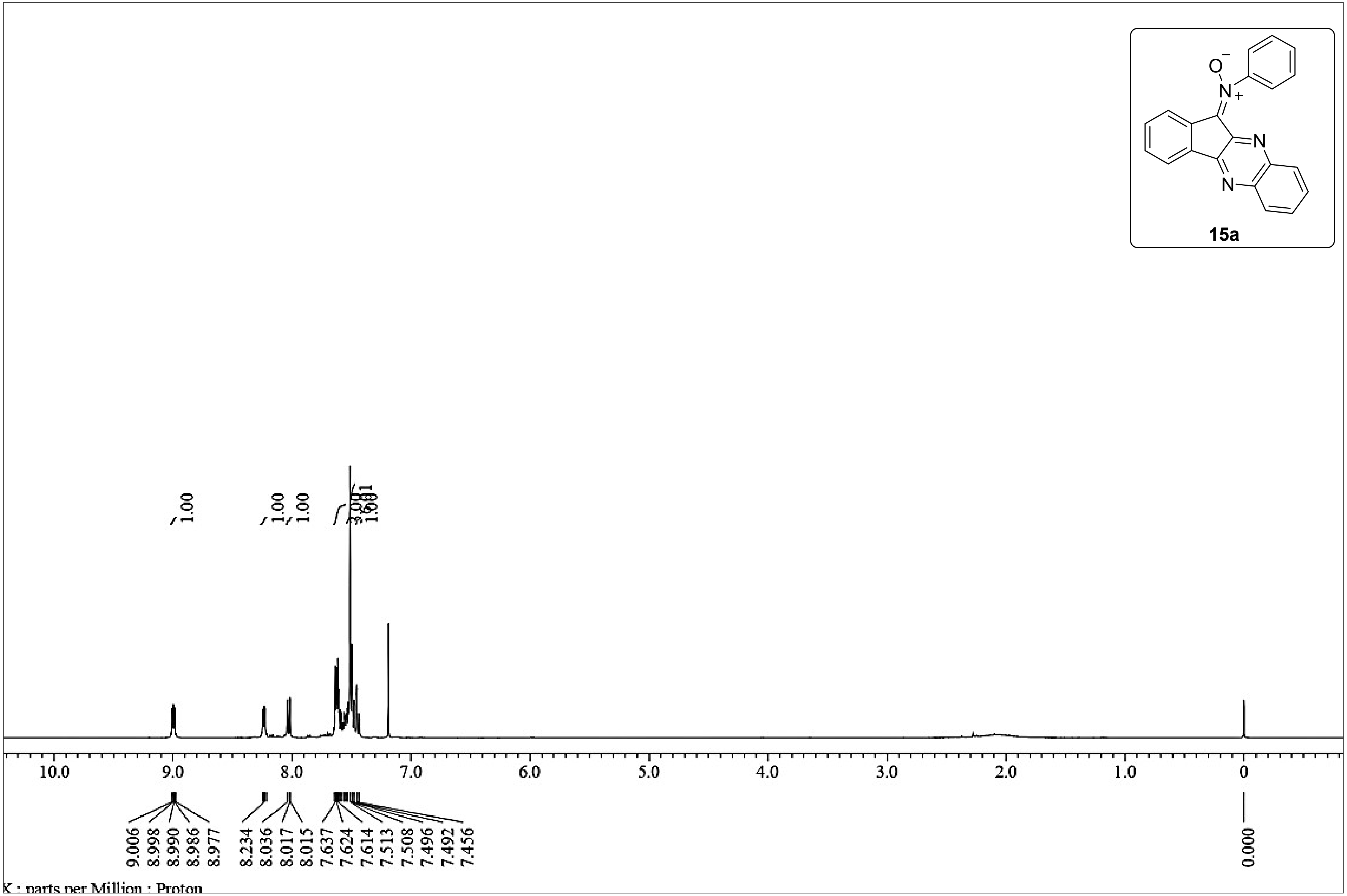

Fig. S1: ^{1}H NMR of (E)-N-phenyl-11H-indeno[1,2-b]quinoxalin-11-imine oxide (15a)

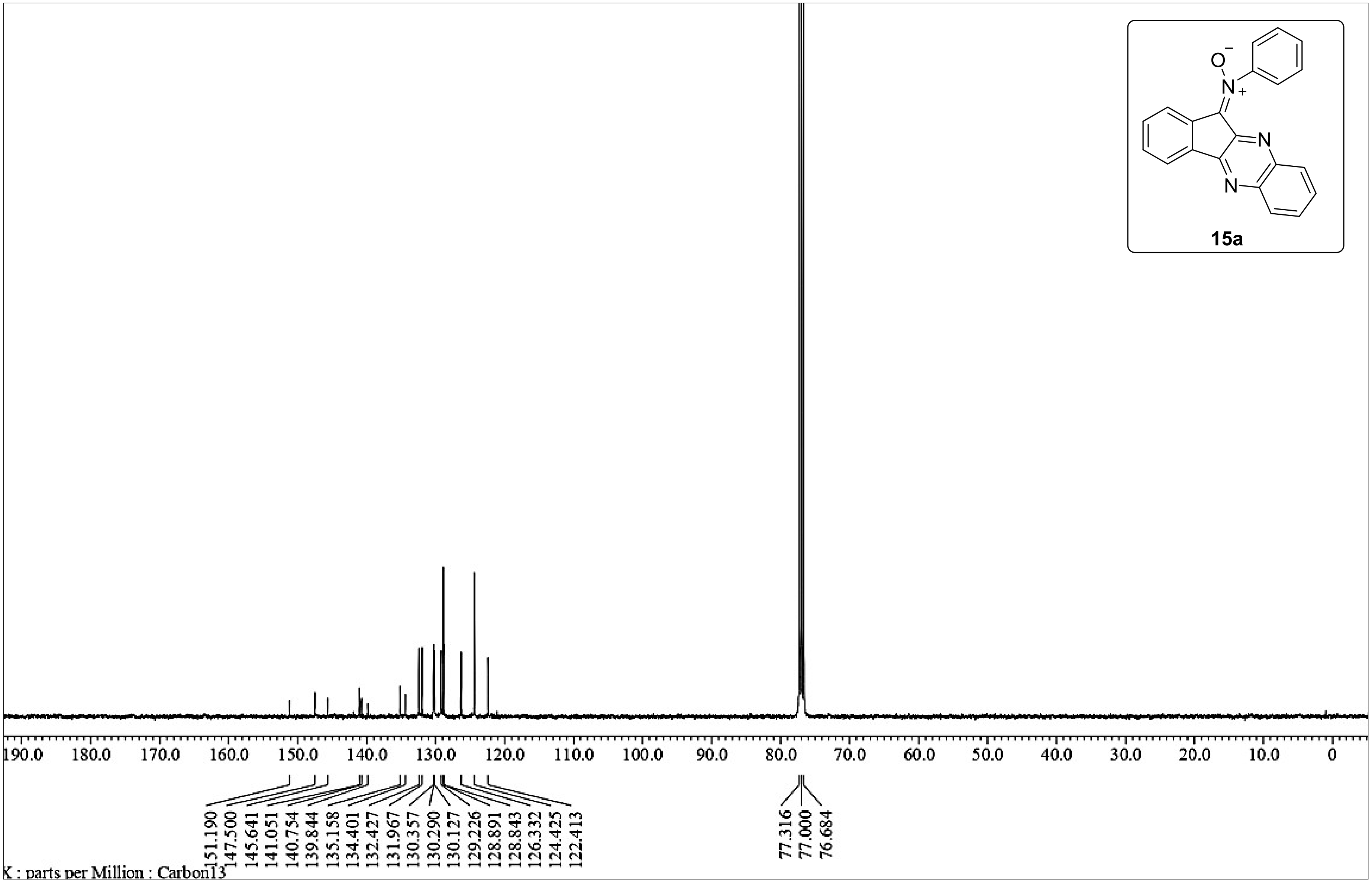

Fig. S2: ^{13}C NMR of (E)-N-phenyl-11H-indeno[1,2-b]quinoxalin-11-imine oxide (15a)

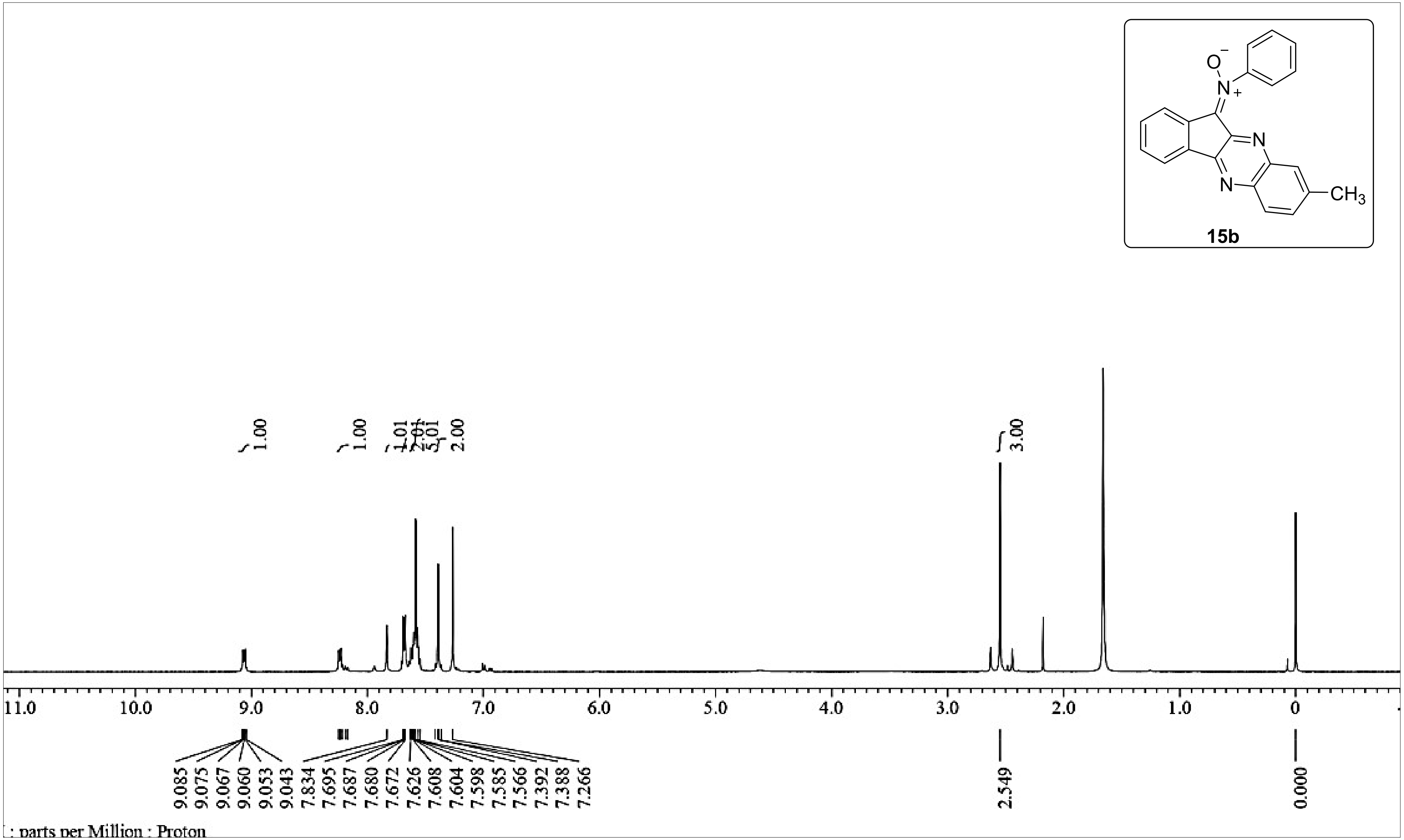

Fig. S3: ^{1}H NMR of (E)-8-methyl-N-phenyl-11H-indeno[1,2-b]quinoxalin-11-imine oxide (15b)

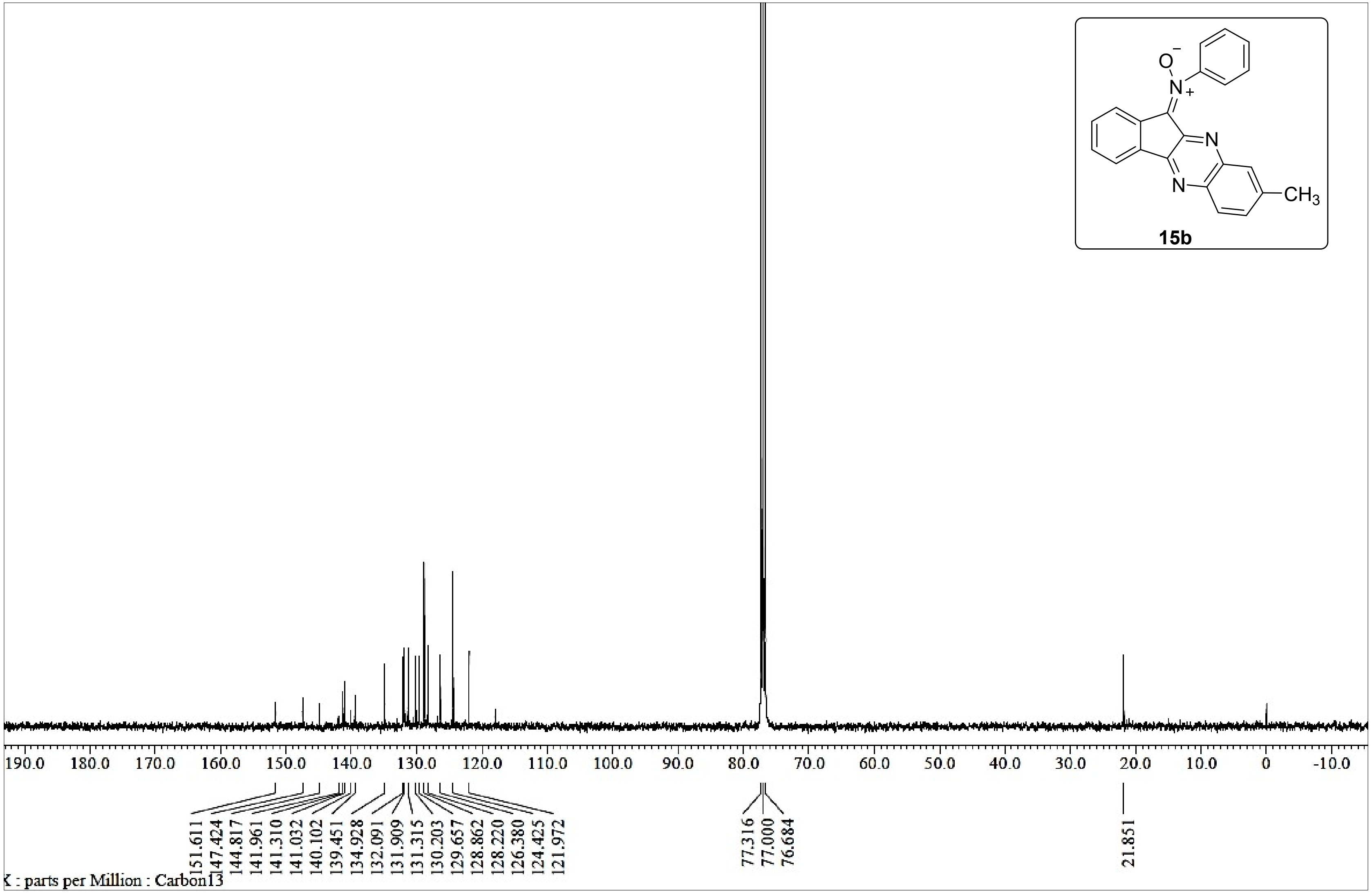

Fig. S4: ^{13}C NMR of (E)-8-methyl-N-phenyl-11H-indeno[1,2-b]quinoxalin-11-imine oxide (15b)

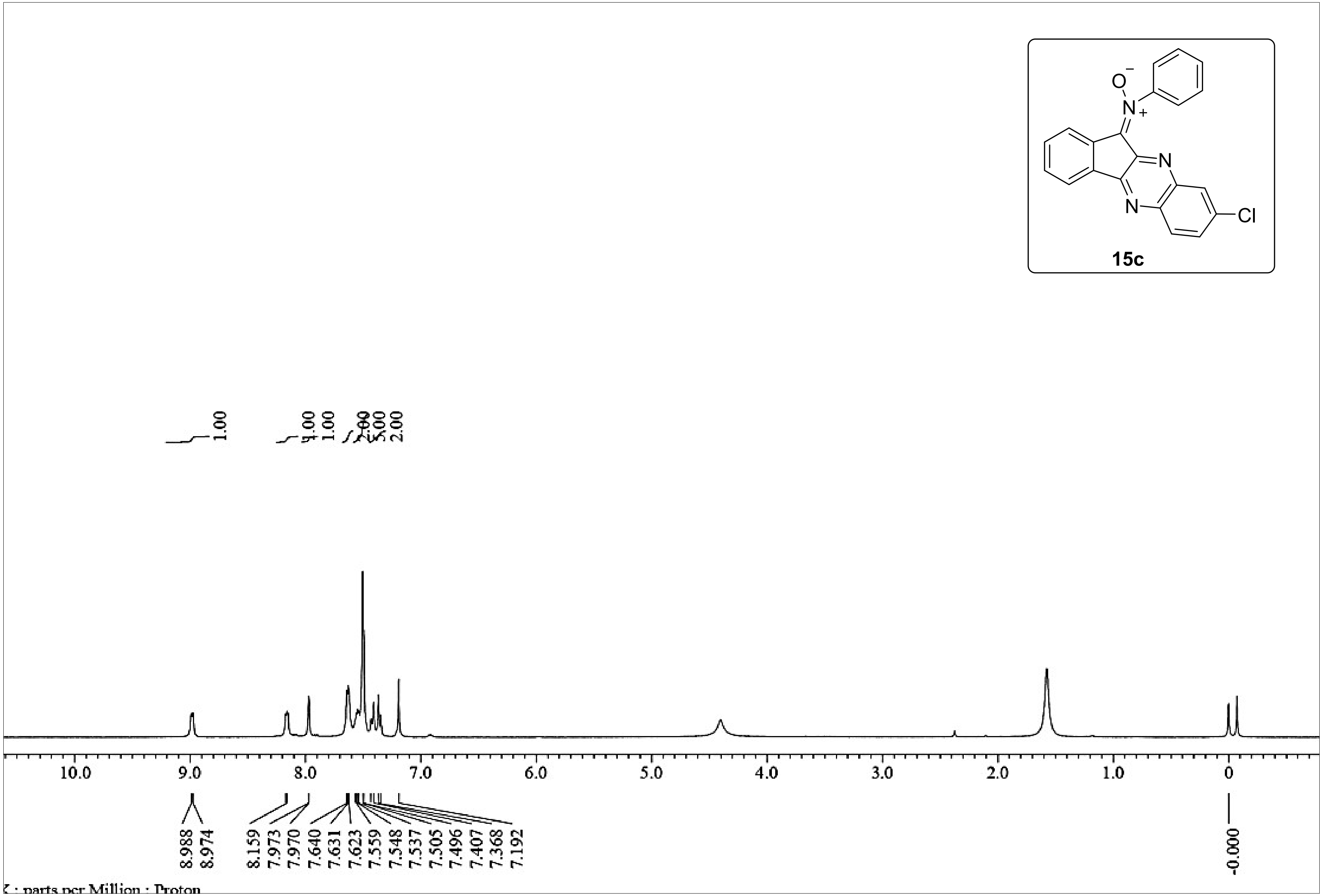

Fig. S5: ^{1}H NMR of (E)-8-chloro-N-phenyl-11H-indeno[1,2-b]quinoxalin-11-imine oxide (15c)

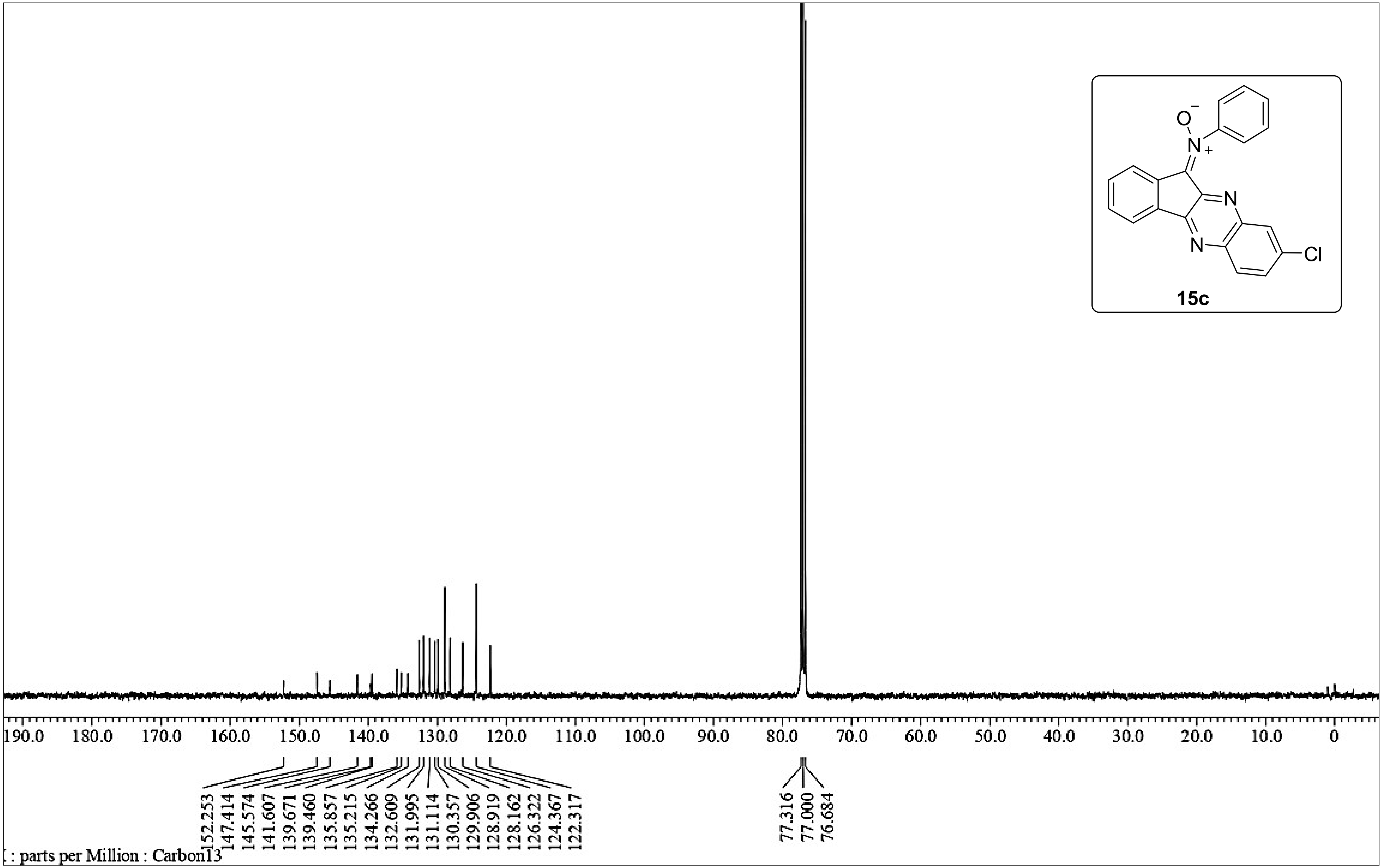

Fig. S6: ^{13}C NMR of (E)-8-chloro-N-phenyl-11H-indeno[1,2-b]quinoxalin-11-imine oxide (15c)

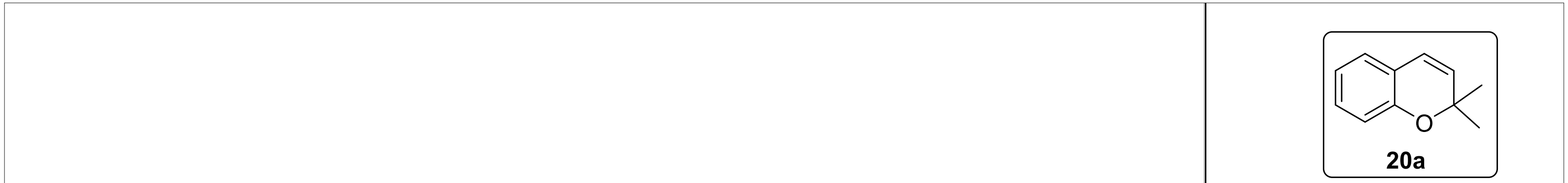

Fig. S7: ^{1}H NMR of 2,2-dimethyl-2H-chromene (20a)

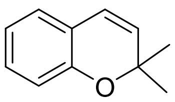

20a

Fig. S8: ^{13}C NMR of 2,2-dimethyl-2H-chromene (20a)

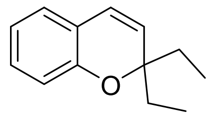

20b

Fig. S9: ^{1}H NMR of 2,2-diethyl-2H-chromene (20b)

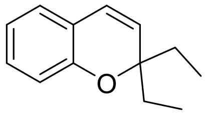

20b

Fig. S10: [13] C NMR of 2,2-diethyl-2H-chromene (20b)

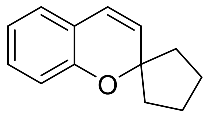

20c

Fig. S11: ^{1}H NMR of spiro[chromene-2,1'-cyclopentane] (20c)

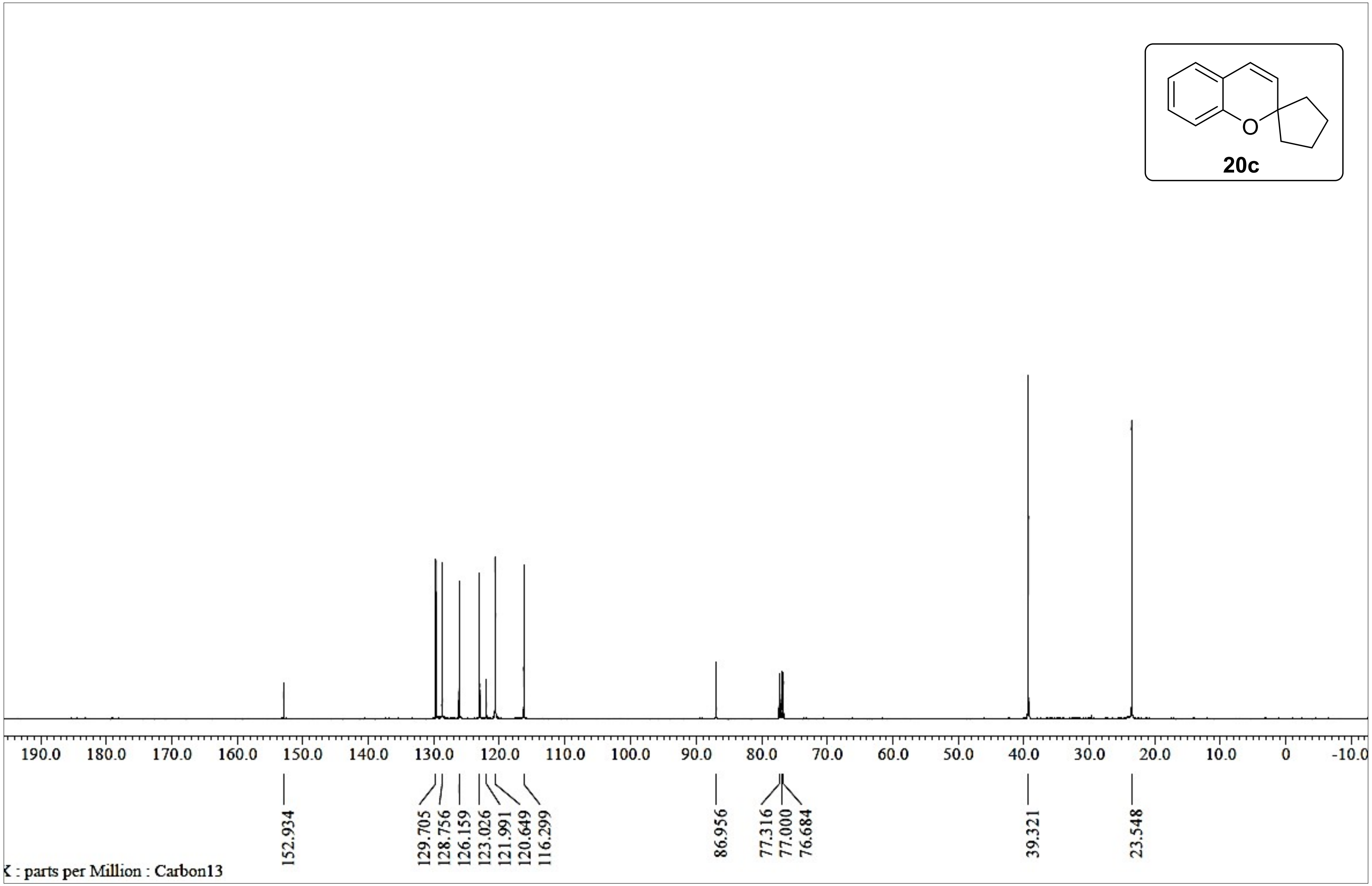

Fig. S12: ^{13}C NMR of spiro[chromene-2,1'-cyclopentane] (20c)

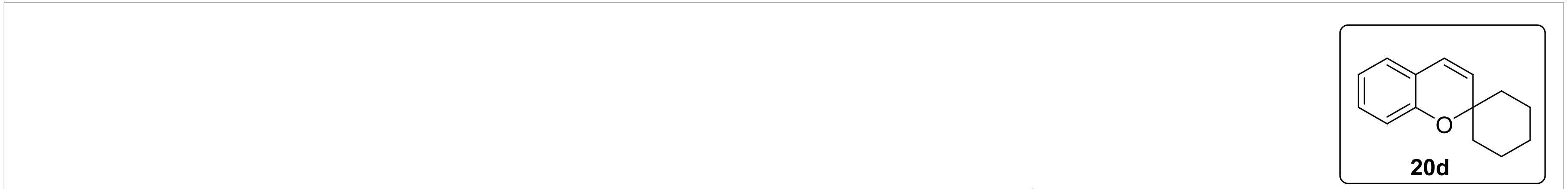

Fig. S13: ^{1}H NMR of spiro[chromene-2,1'-cyclohexane] (20d)

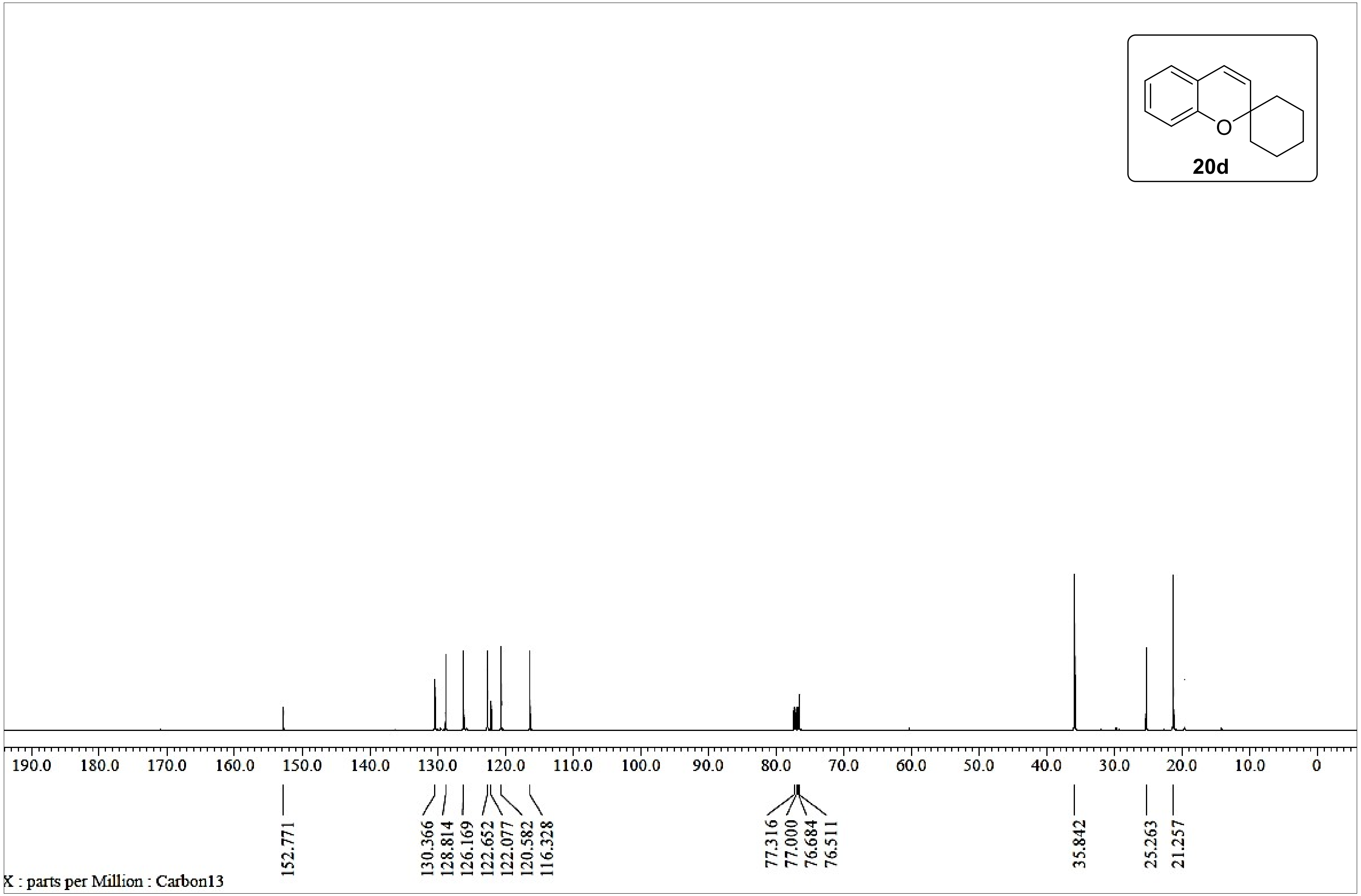
20d
190.0 180.0 170.0 160.0 150.0 140.0 130.0 120.0 110.0 100.0 90.0 80.0 70.0 60.0 50.0 40.0 30.0 20.0 10.0 0
152.771
130.366
128.814
126.169
122.652
122.077
120.582
116.328
77.316
77.000
76.684
76.511
35.842
25.263
21.257
X : parts per Million : Carbon13

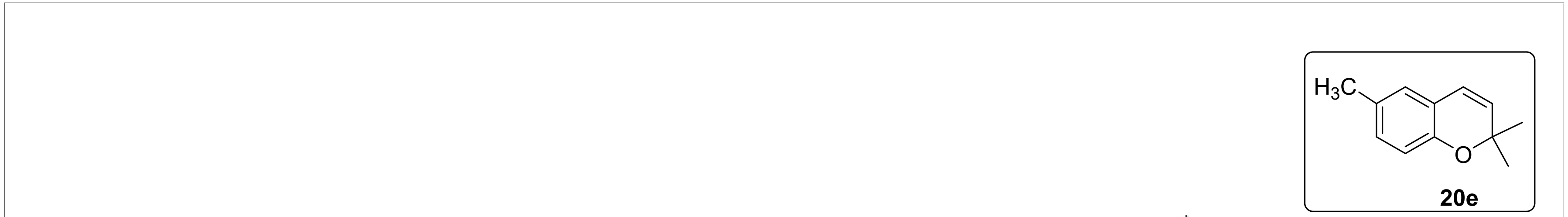
H3C
O
20e

Fig. S15: ¹H NMR of 2,2,6-trimethyl-2H-chromene (20e)

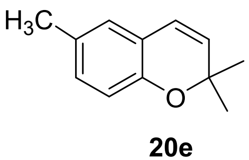

20e

Fig. S16: ^{13}C NMR of 2,2,6-trimethyl-2H-chromene (20e)

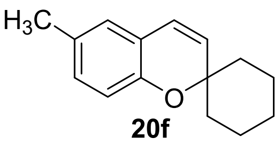

20f

Fig. S17: ^{1}H NMR of 6-methylspiro[chromene-2,1'-cyclohexane] (20f)

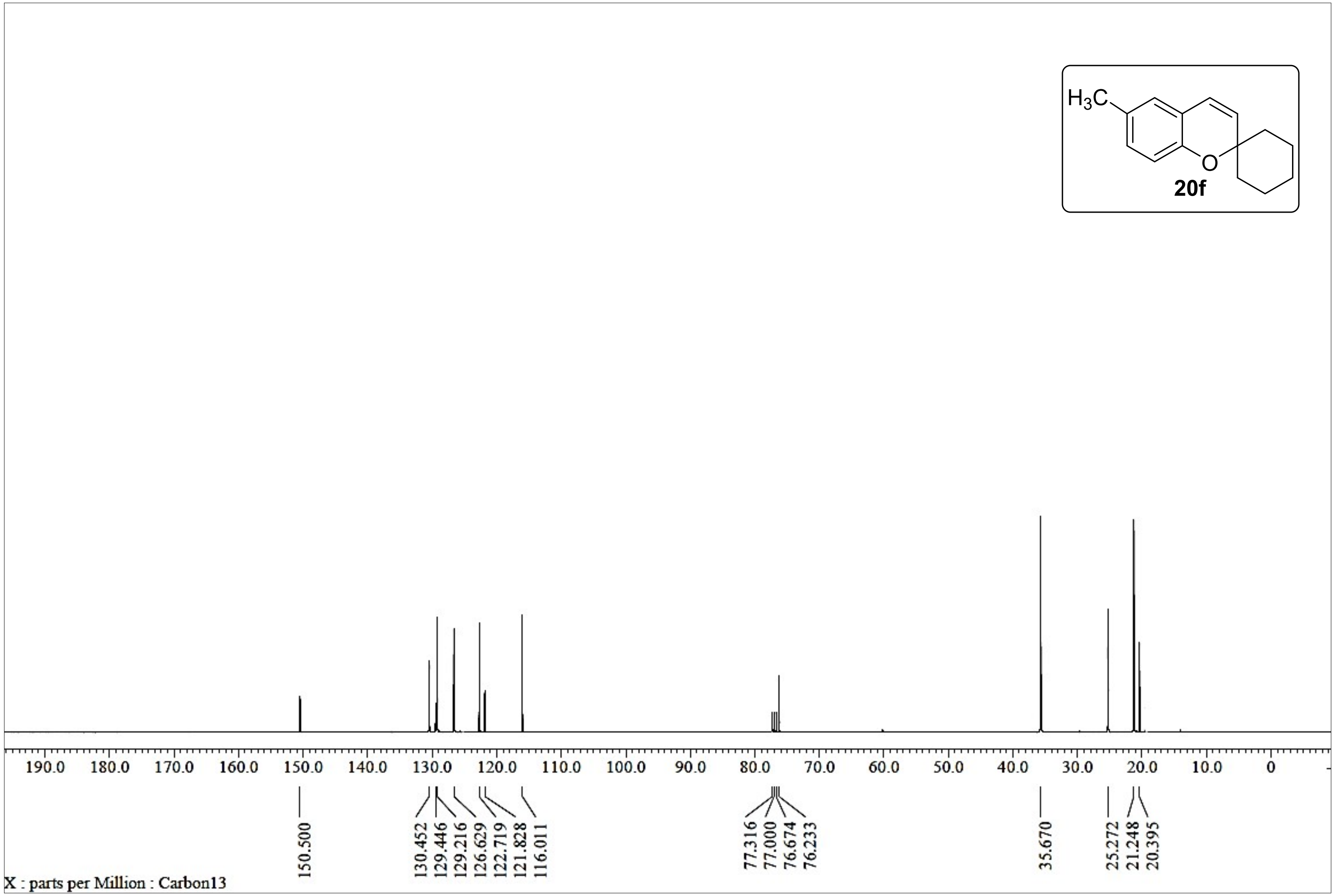

Fig. S18: ^{13}C NMR of 6-methylspiro[chromene-2,1'-cyclohexane] (20f)

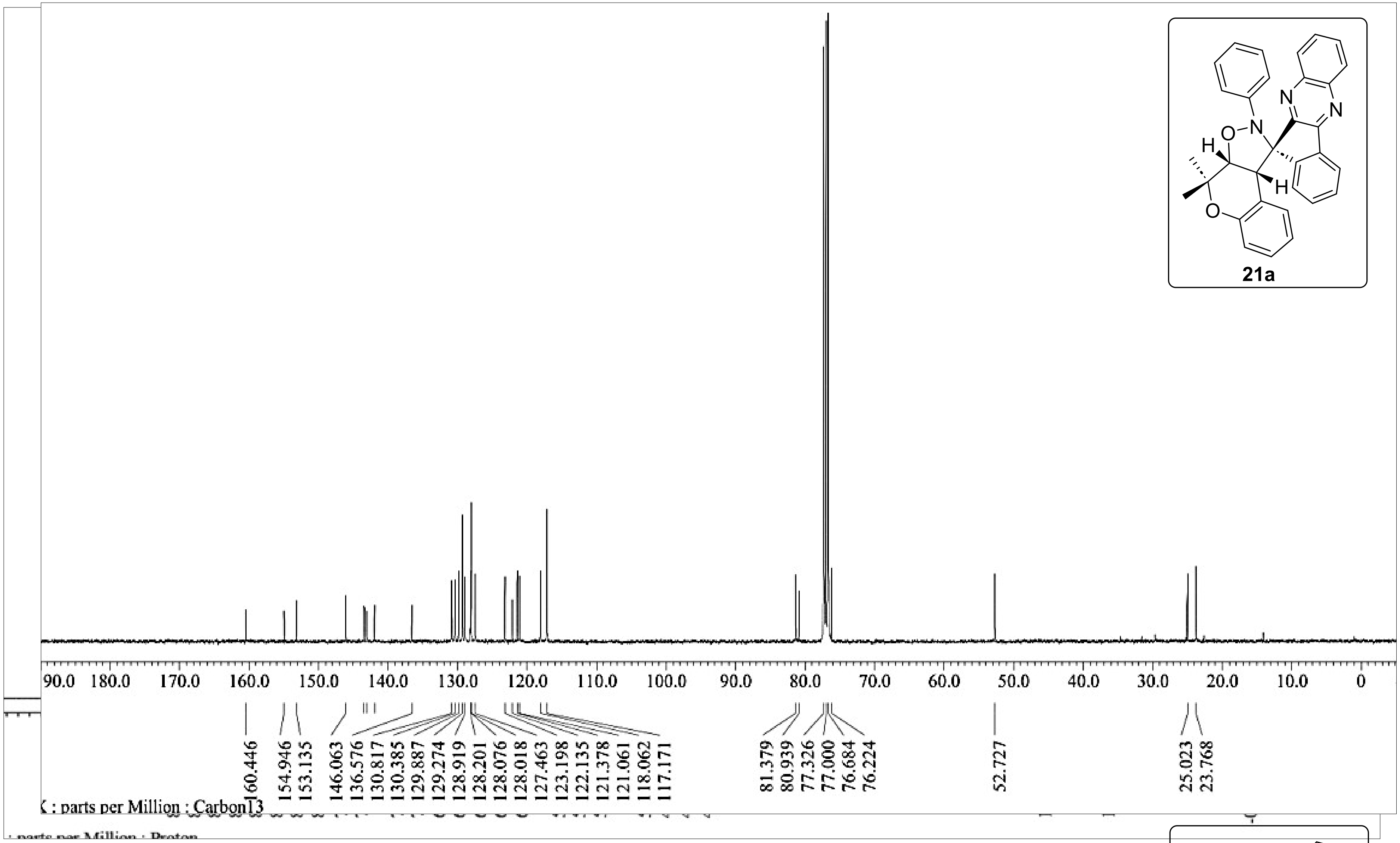

Fig. S19: ^{1}H NMR of 4,4-dimethyl-2-phenyl-3a,9b-dihydro-2H,4H-spiro[chromeno[4,3-d]isoxazole-1,11'-indeno[1,2-b]quinoxaline] (21a)

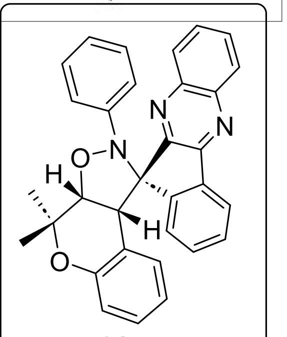

21a

Fig. S20: ^{13}C NMR of 4,4-dimethyl-2-phenyl-3a,9b-dihydro-2H,4H-spiro[chromeno[4,3-d]isoxazole-1,11'-indeno[1,2-b]quinoxaline] (21a)

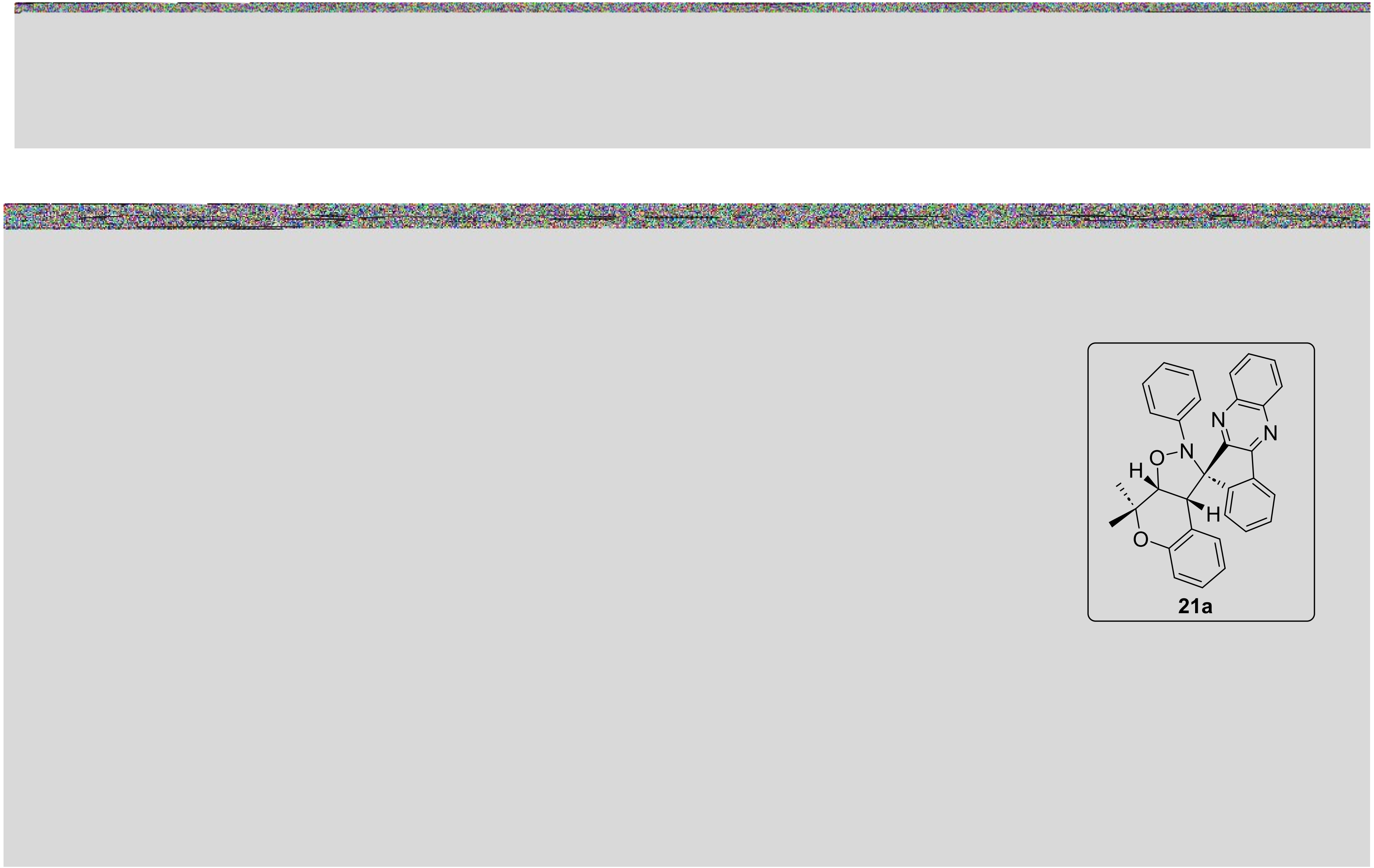

Fig. S21: HRMS of 4,4-dimethyl-2-phenyl-3a,9b-dihydro-2H,4H-spiro[chromeno[4,3-d]isoxazole-1,11'-indeno[1,2-b]quinoxaline] (21a)

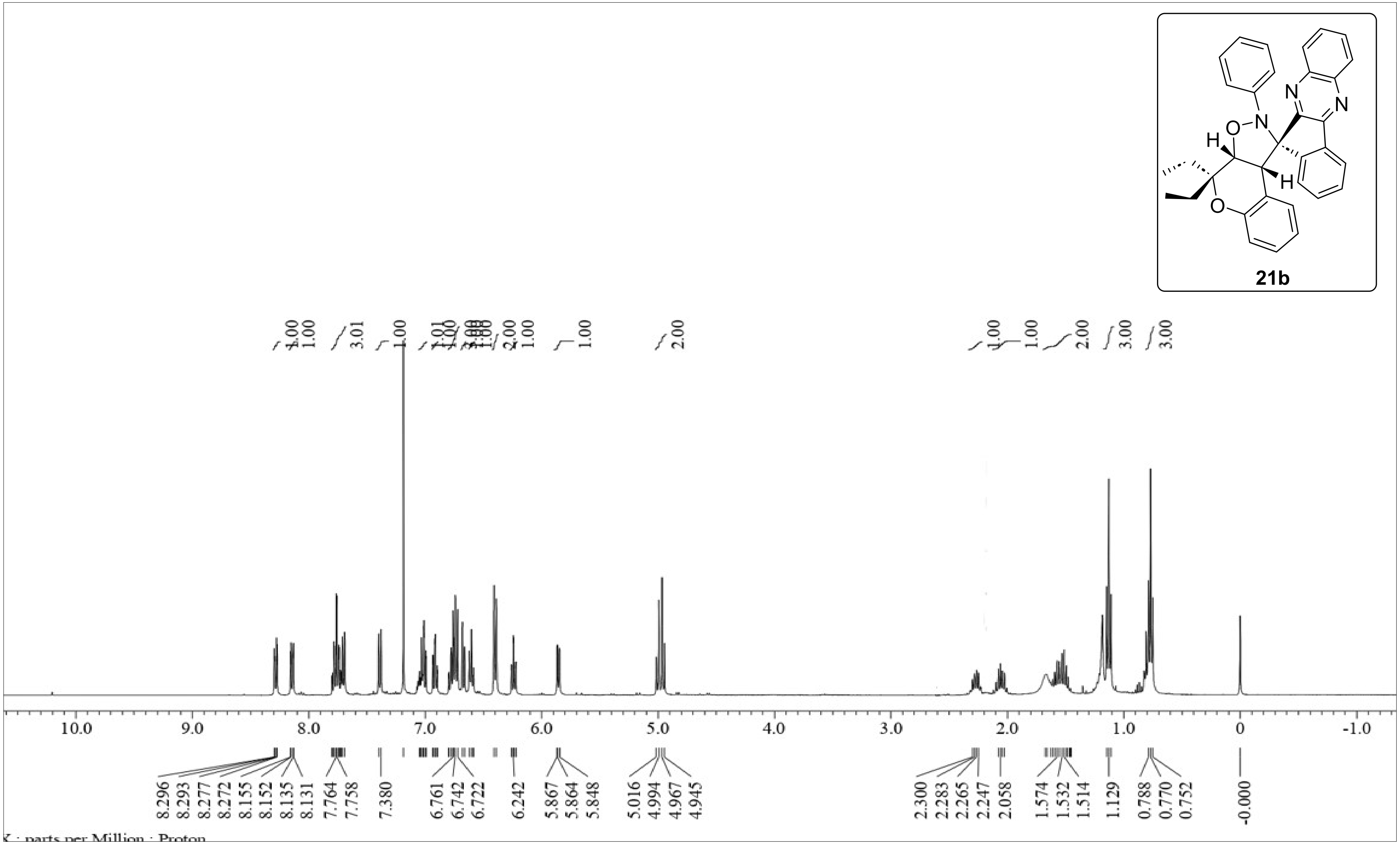

Fig. S22: ^{1}H NMR of 4,4-diethyl-2-phenyl-3a,9b-dihydro-2H,4H-spiro[chromeno[4,3-d]isoxazole-1,11'-indeno[1,2-b]quinoxaline] (21b)

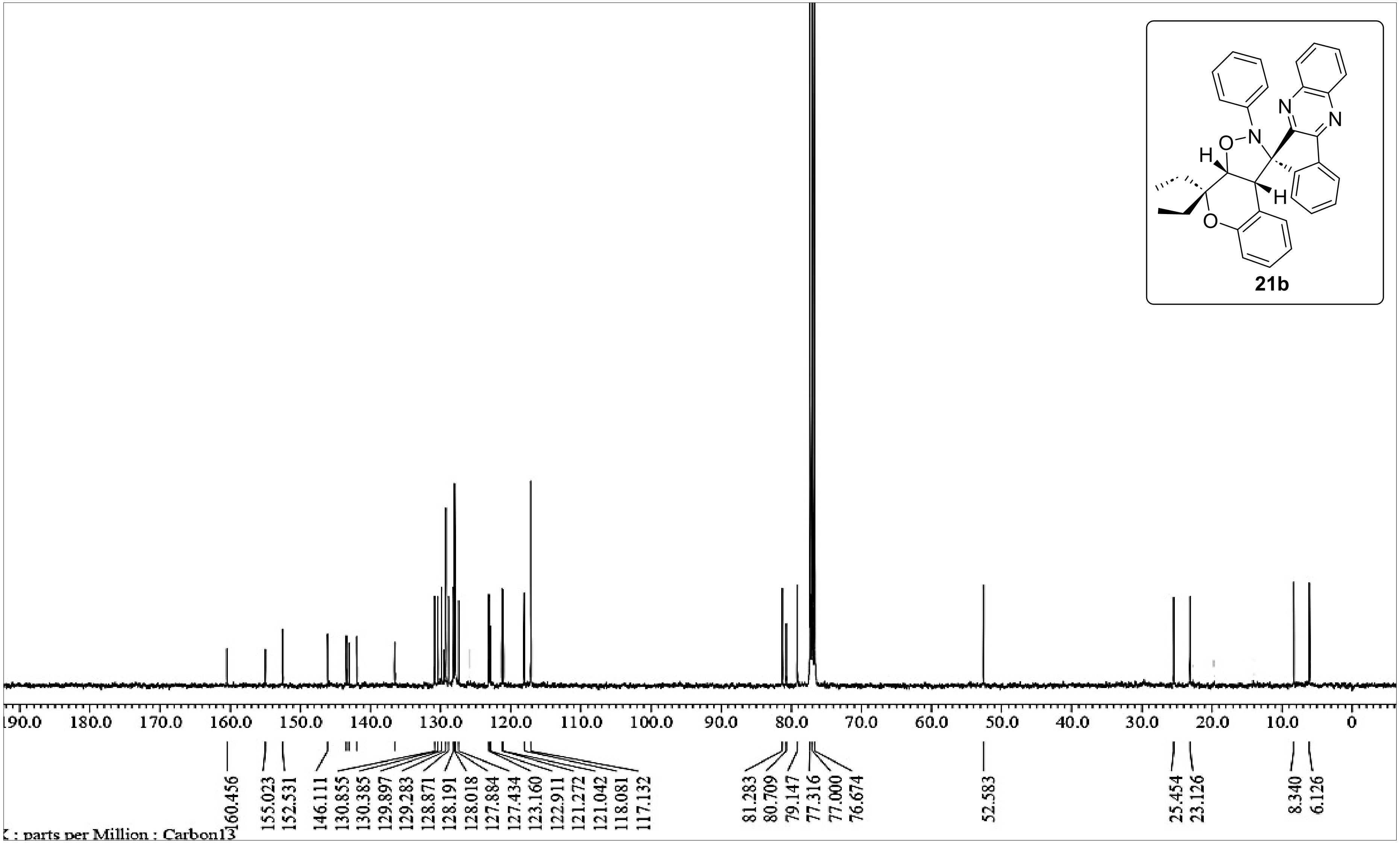

Fig. S23: ^{13}C NMR of 4,4-diethyl-2-phenyl-3a,9b-dihydro-2H,4H-spiro[chromeno[4,3-d]isoxazole-1,11'-indeno[1,2-b]quinoxaline] (21b)

Compound Details

Cpd. 1: C34 H29 N3 O2

Formula	m/z	Observed M/Z	Difference_Da	Difference_PPM	Score
C34 H29 N3 O2	512.2335	512.233488313142	0.330392930663947	0.646275708599071	99.60

Compound Spectra (Zoomed)

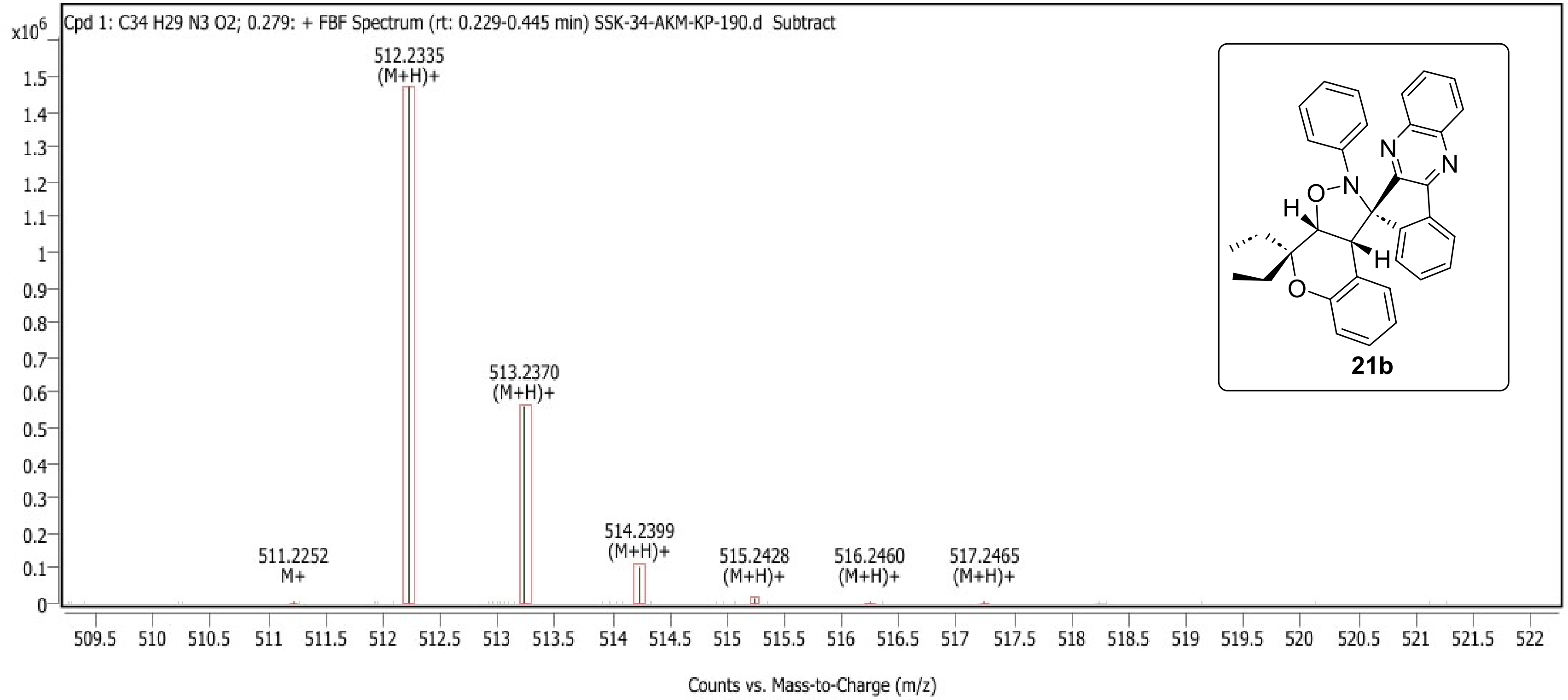

Fig. S24: HRMS of 4,4-diethyl-2-phenyl-3a,9b-dihydro-2H,4H-spiro[chromeno[4,3-d]isoxazole-1,11'-indeno[1,2-b]quinoxaline] (21b)

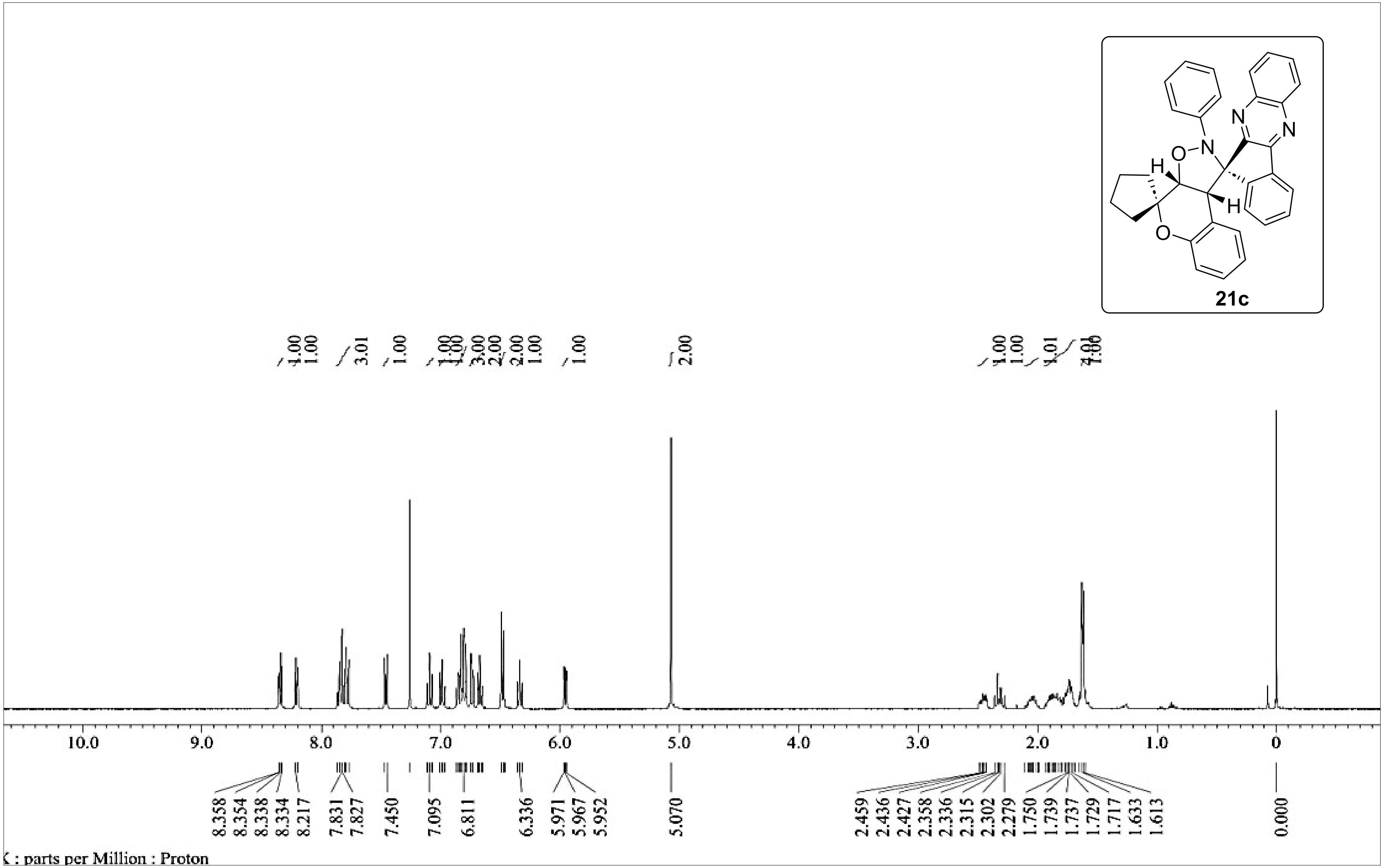

Fig. S25: ^{1}H NMR of 2'-phenyl-3a',9b'-dihydro-2'H-dispiro[cyclopentane-1,4'-chromeno[4,3-d]isoxazole-1',11''-indeno[1,2-b]quinoxaline] (21c)

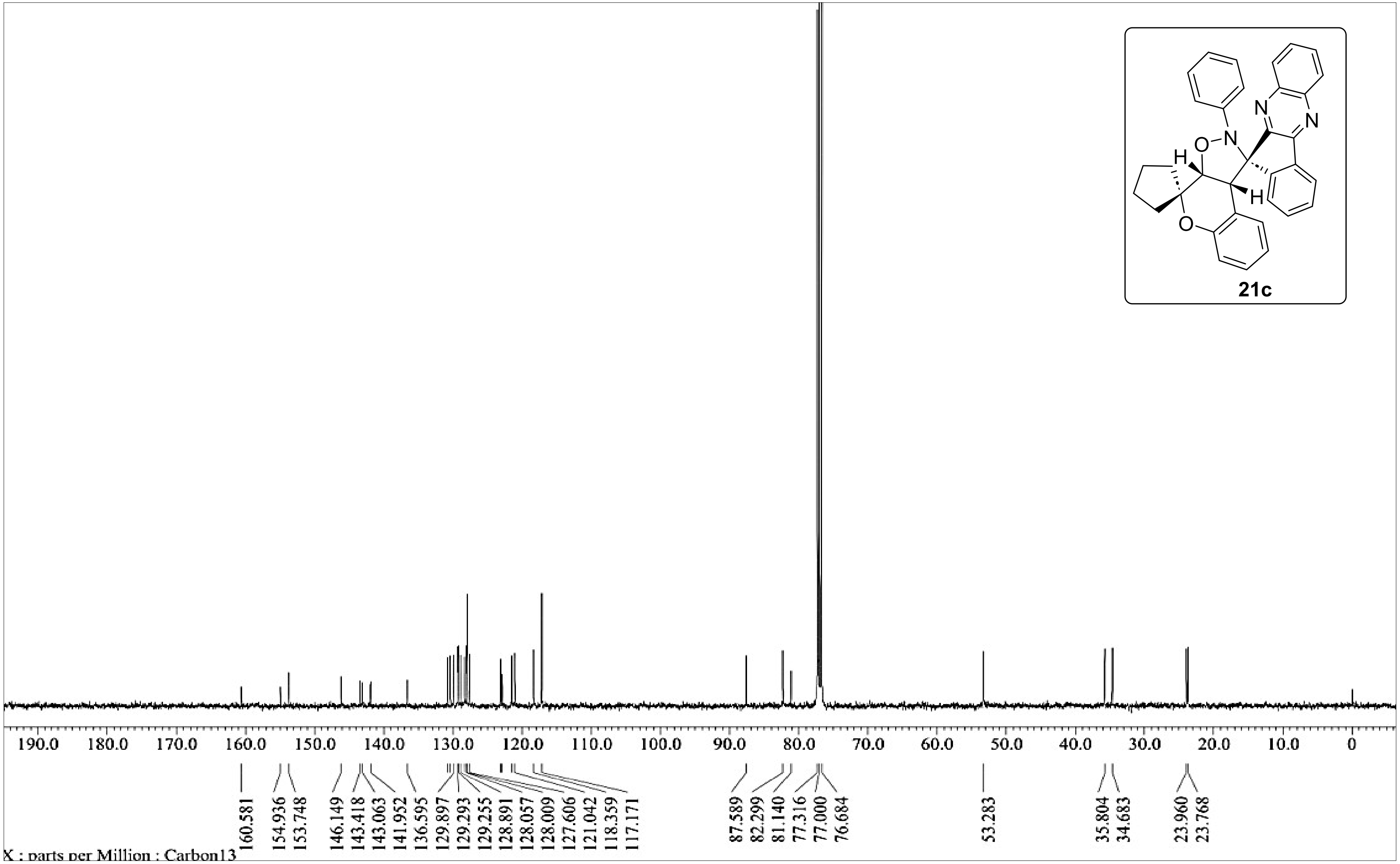

Fig. S26: ^{13}C NMR of 2'-phenyl-3a',9b'-dihydro-2'H-dispiro[cyclopentane-1,4'-chromeno[4,3-d]isoxazole-1',11''-indeno[1,2-b]quinoxaline] (21c)

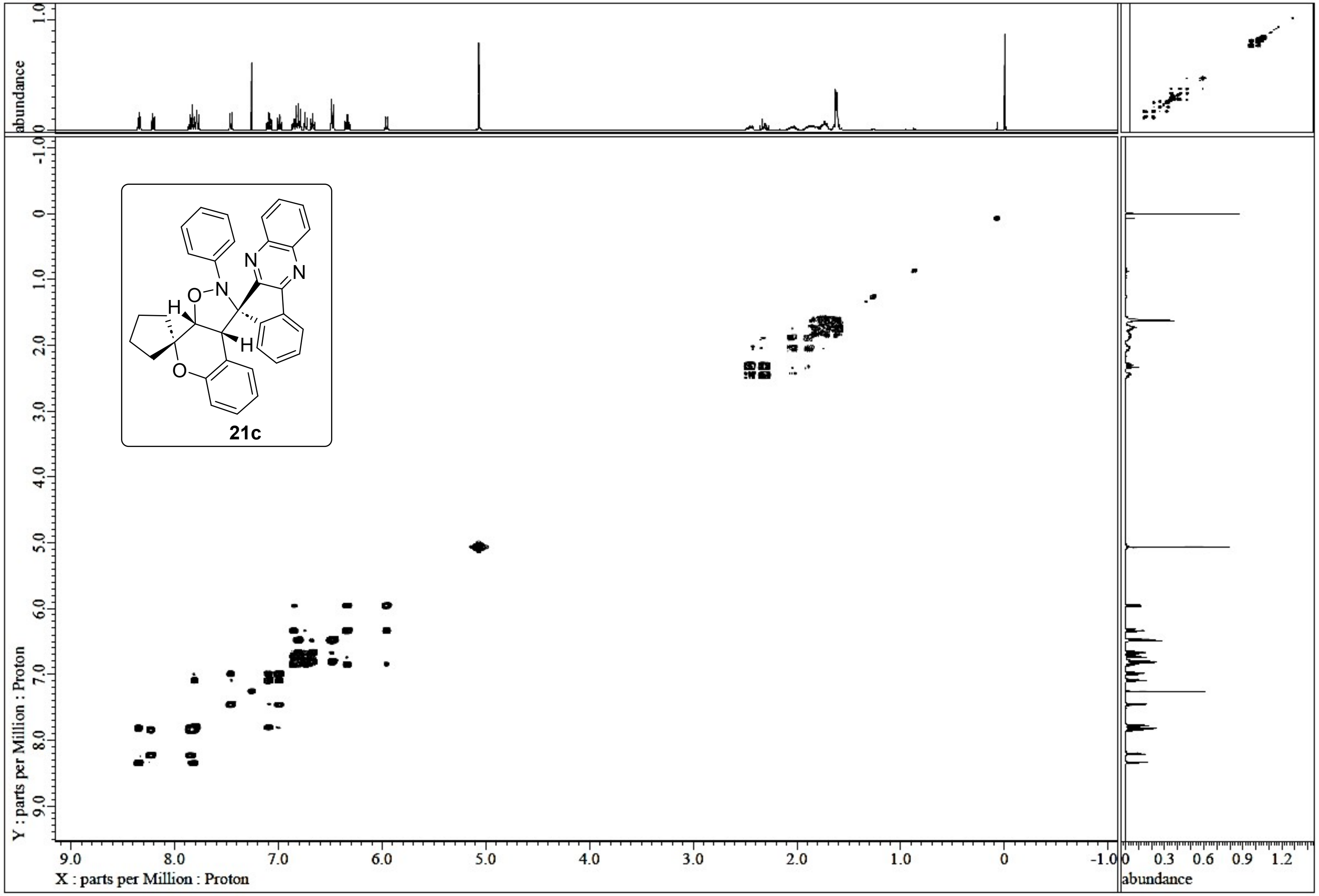

Fig. S27: COSY of 2'-phenyl-3a',9b'-dihydro-2'H-dispiro[cyclopentane-1,4'-chromeno[4,3-d]isoxazole-1',11''-indeno[1,2-b]quinoxaline] (21c)

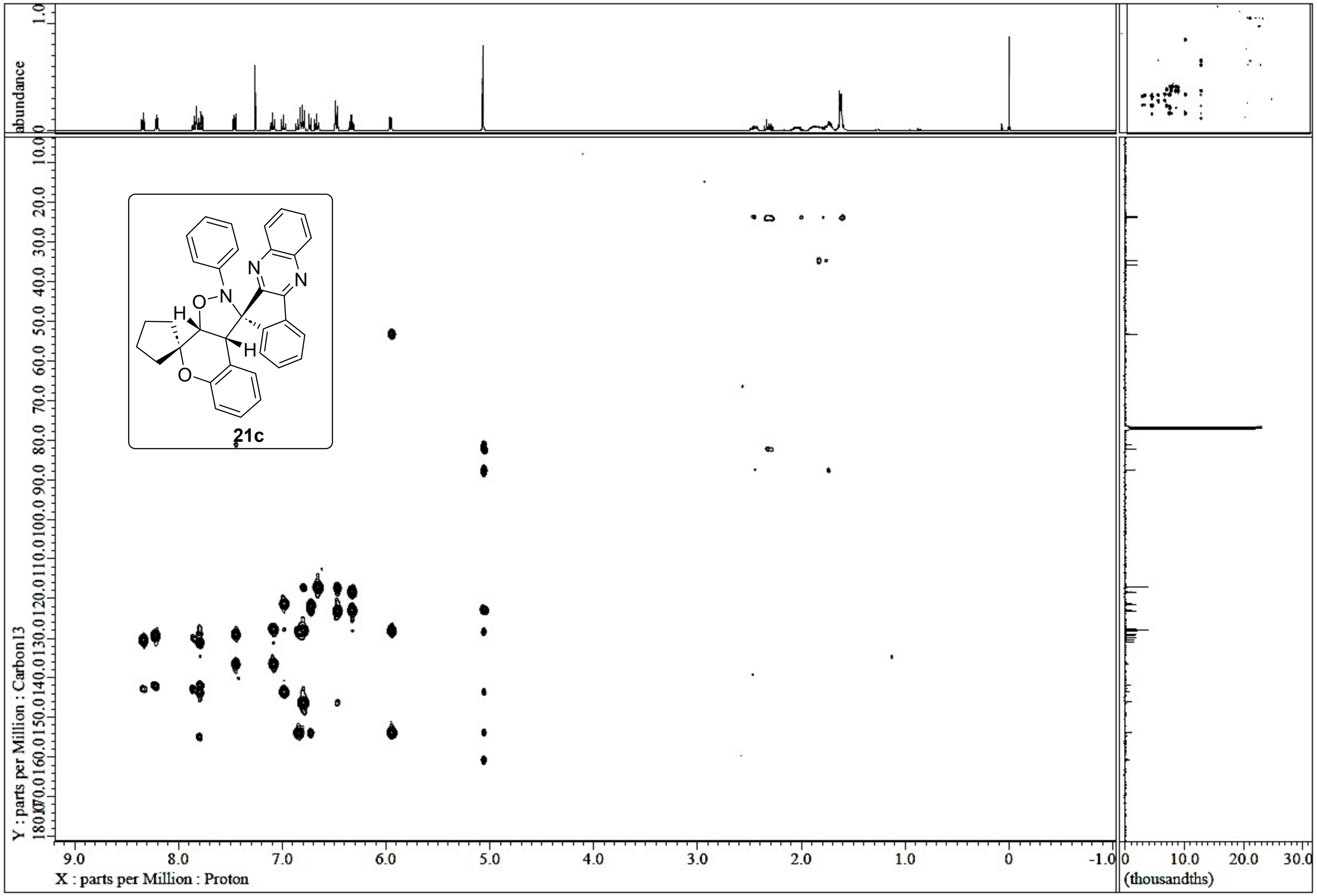

Fig. S28: HMBC of 2'-phenyl-3a',9b'-dihydro-2'H-dispiro[cyclopentane-1,4'-chromeno[4,3-d]isoxazole-1',11''-indeno[1,2-b]quinoxaline] (21c)

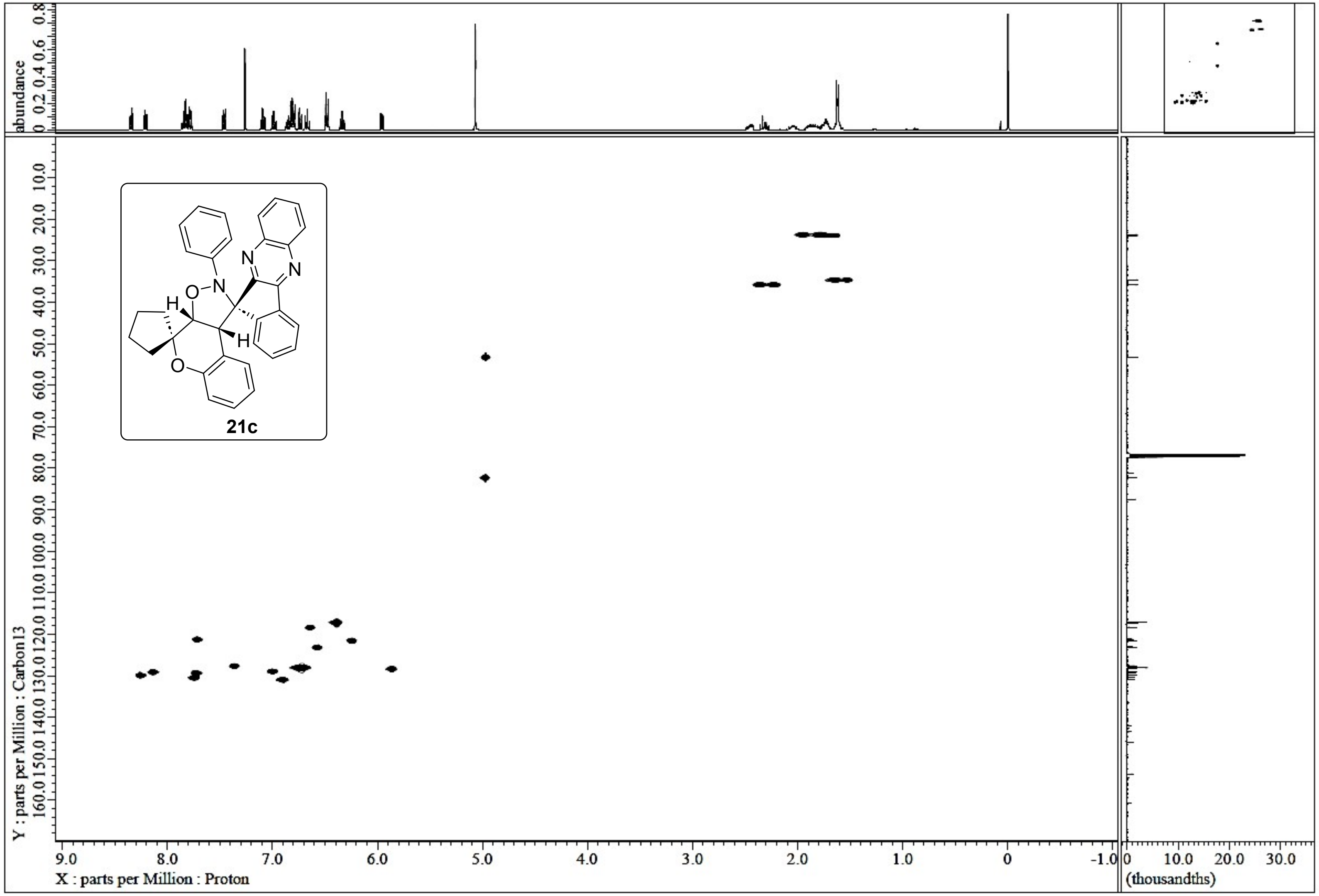

Fig. S29: HSQC of 2'-phenyl-3a',9b'-dihydro-2'H-dispiro[cyclopentane-1,4'-chromeno[4,3-d]isoxazole-1',11''-indeno[1,2-b]quinoxaline] (21c)

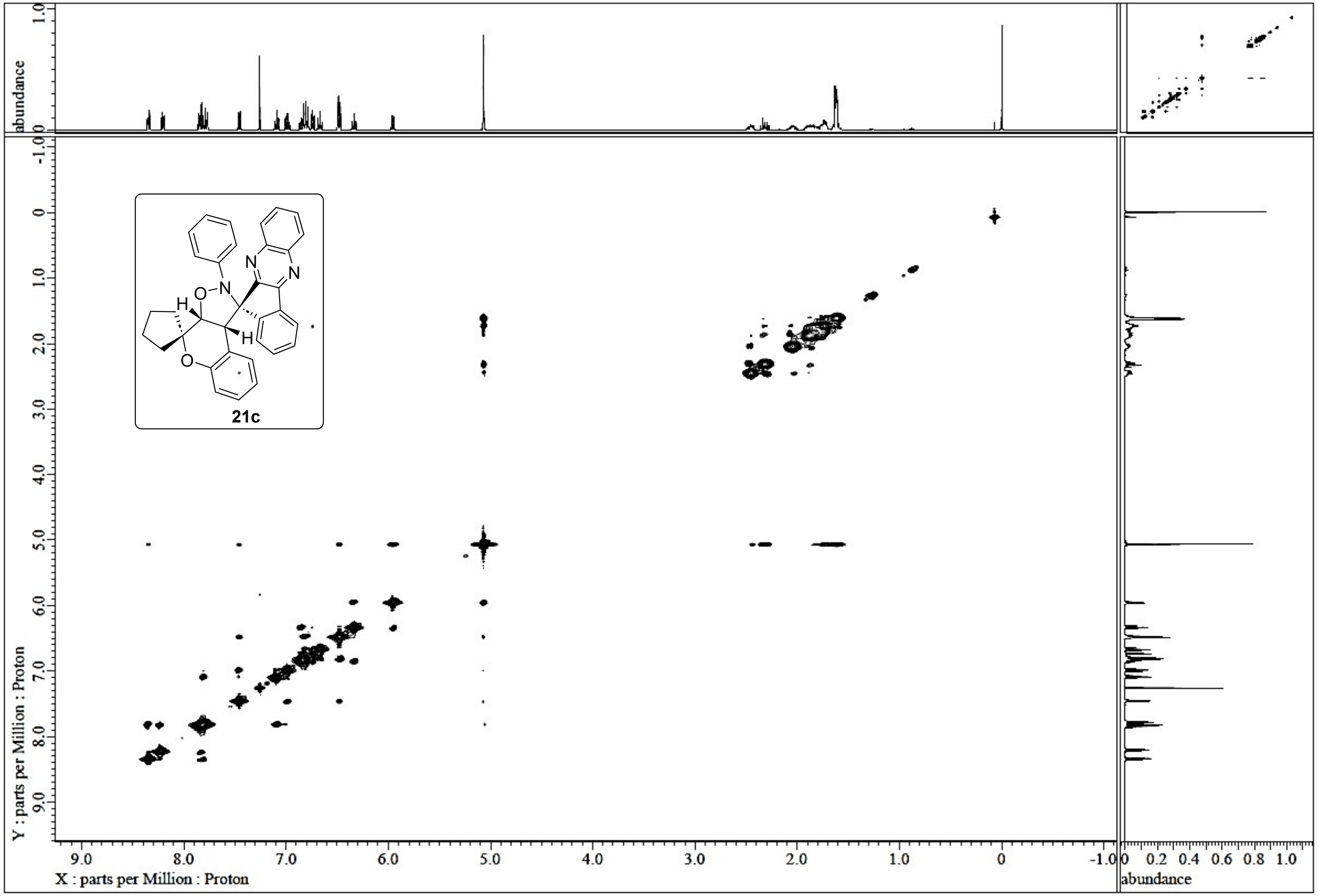

Fig. S30: NOESY of 2'-phenyl-3a',9b'-dihydro-2'H-dispiro[cyclopentane-1,4'-chromeno[4,3-d]isoxazole-1',11''-indeno[1,2-b]quinoxaline] (21c)

Compound Details

Cpd. 1: C34 H27 N3 O2

Formula	m/z	Observed M/Z	Difference_Da	Difference_PPM	Score
C34 H27 N3 O2	510.2175	510.217545190887	-0.27459431527177	-0.539255197003676	99.23

Compound Spectra (Zoomed)

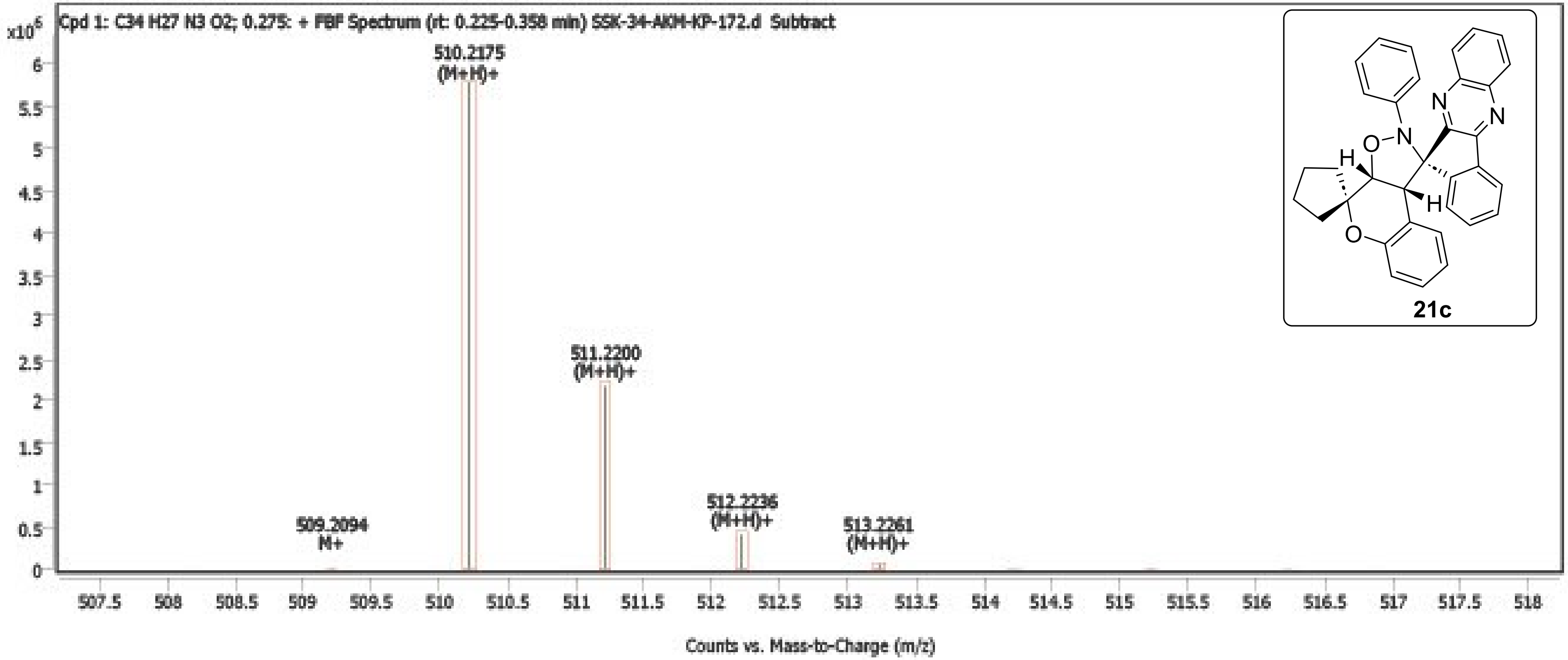

Fig. S31: HRMS of 2'-phenyl-3a',9b'-dihydro-2'H-dispiro[cyclopentane-1,4'-chromeno[4,3-d]isoxazole-1',11''-indeno[1,2-b]quinoxaline] (21c)

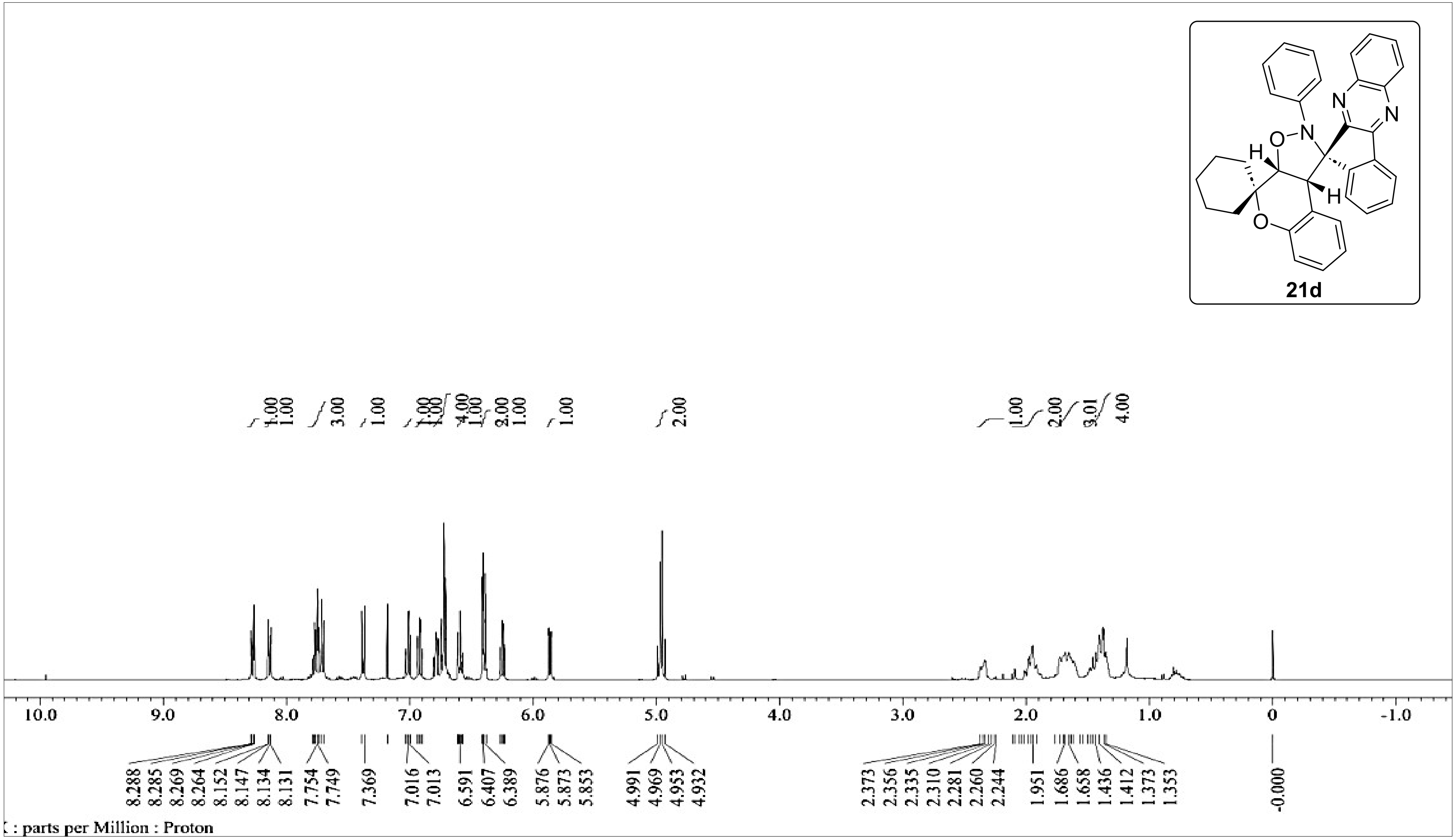

Fig. S32: **^{1}H NMR of 2'-phenyl-3a',9b'-dihydro-2'H-dispiro[cyclohexane-1,4'-chromeno[4,3-d]isoxazole-1',11''-indeno[1,2-b]quinoxaline] (21d)**

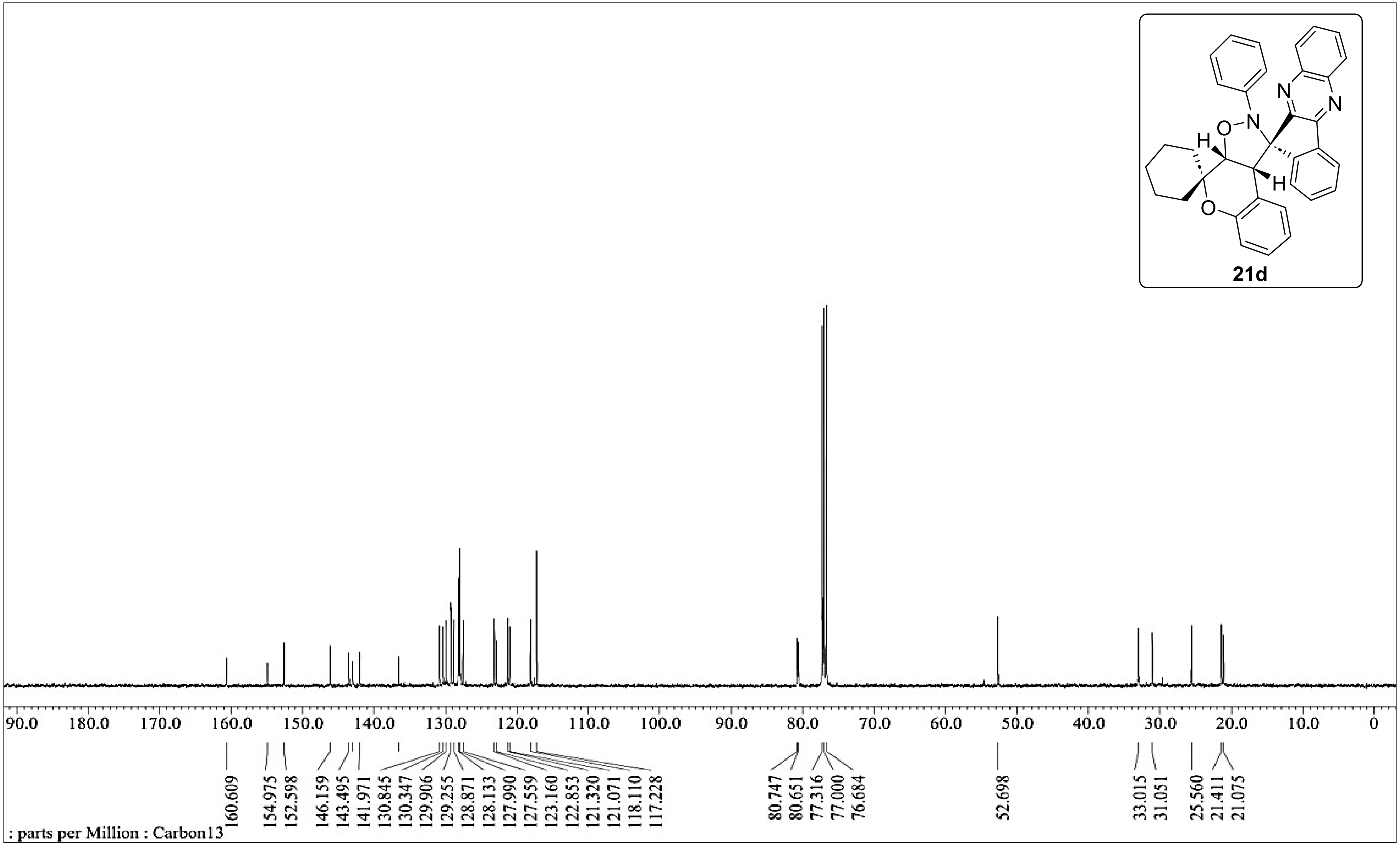

Fig. S33: ^{13}C NMR of 2'-phenyl-3a',9b'-dihydro-2'H-dispiro[cyclohexane-1,4'-chromeno[4,3-d]isoxazole-1',11''-indeno[1,2-b]quinoxaline] (21d)

Compound Details

Cpd. 1: C35 H29 N3 O2

Formula	m/z	Observed M/Z	Difference_Da	Difference_PPM	Score
C35 H29 N3 O2	524.2348	524.234831328131	0.851746782245755	1.62787556309225	85.29

Compound Spectra (Zoomed)

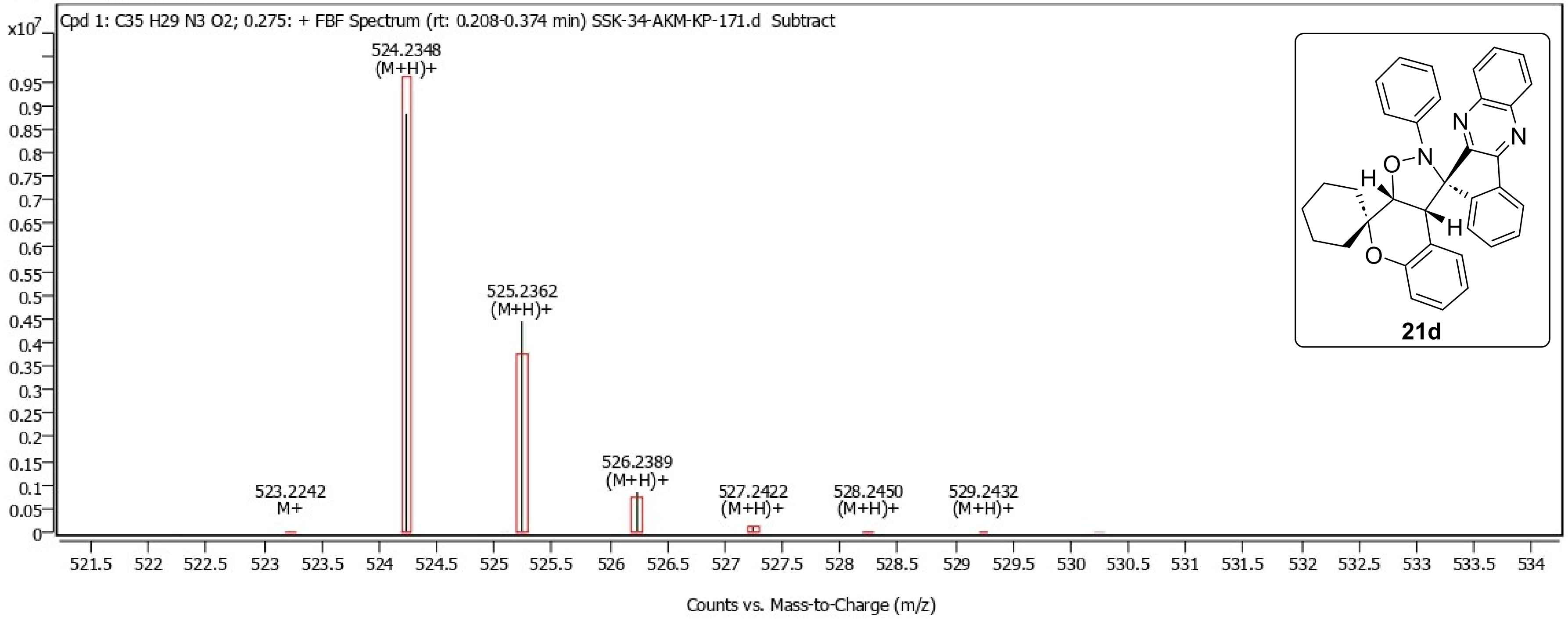

Fig. S34: HRMS of 2'-phenyl-3a',9b'-dihydro-2'H-dispiro[cyclohexane-1,4'-chromeno[4,3-d]isoxazole-1',11''-indeno[1,2-b]quinoxaline] (21d)

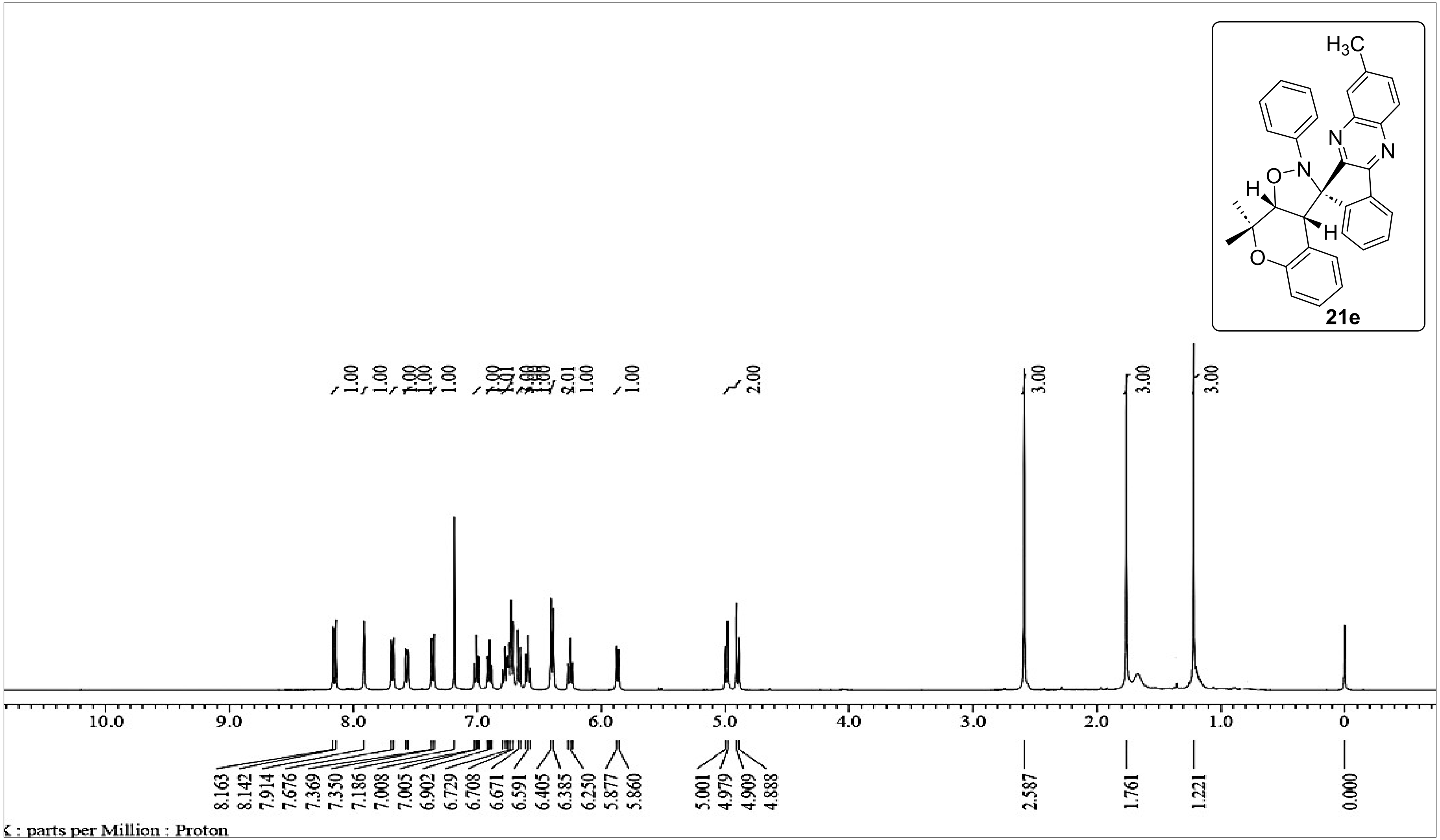

Fig. S35: ^{1}H NMR of 4,4,8'-trimethyl-2-phenyl-3a,9b-dihydro-2H,4H-spiro[chromeno[4,3-d]isoxazole-1,11'-indeno[1,2-b]quinoxaline] (21e)

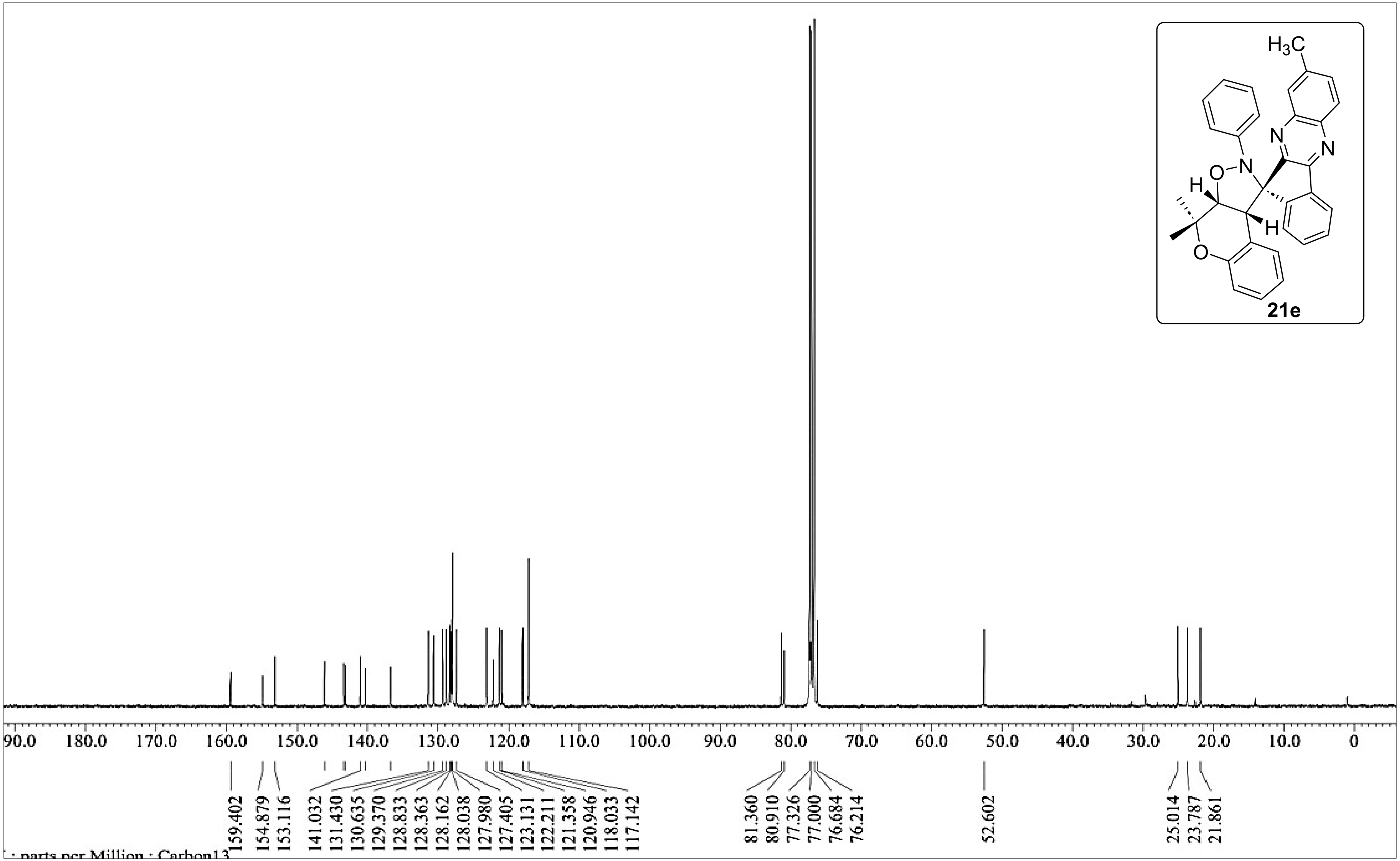

Fig. S36: ^{13}C NMR of 4,4,8'-trimethyl-2-phenyl-3a,9b-dihydro-2H,4H-spiro[chromeno[4,3-d]isoxazole-1,11'-indeno[1,2-b]quinoxaline] (21e)

Compound Details

Cpd. 1: C33 H27 N3 O2

Formula	m/z	Observed M/Z	Difference_Da	Difference_PPM	Score
C33 H27 N3 O2	498.2179	498.217864519811	0.304899848401874	0.613221069166008	99.11

Compound Spectra (Zoomed)

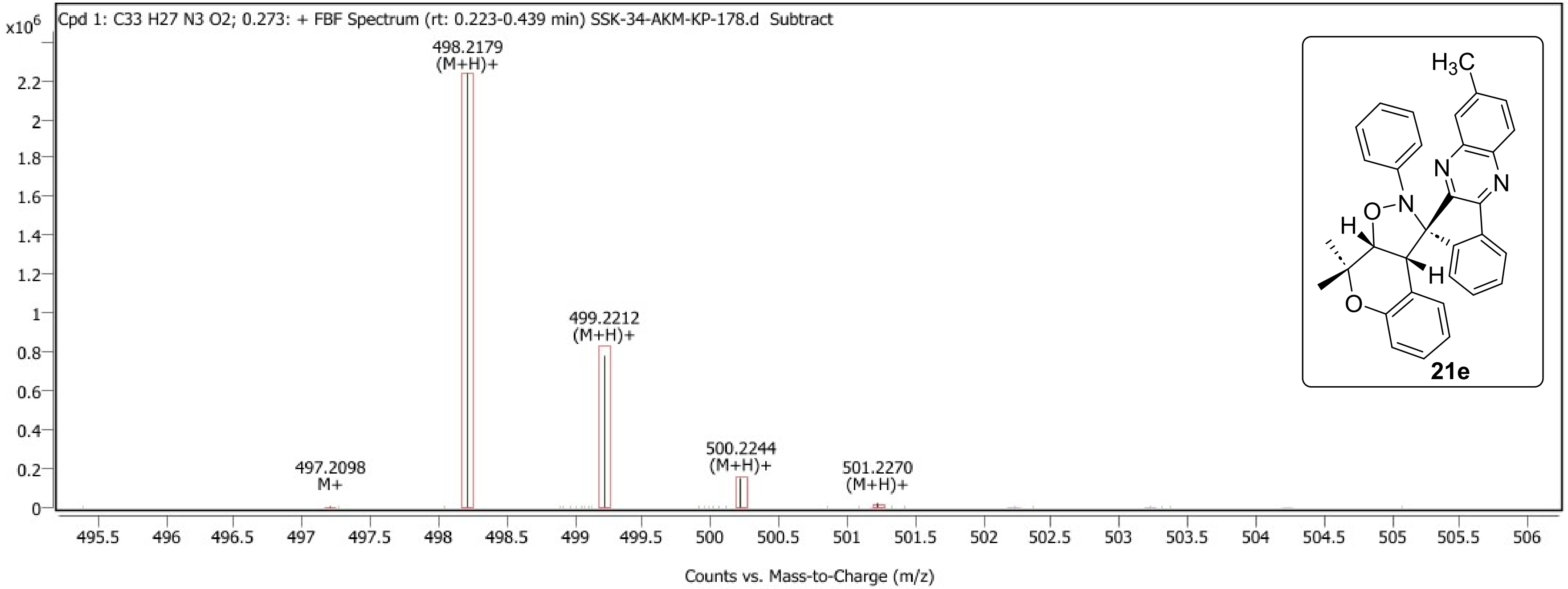

Fig. S37: HRMS of 4,4,8'-trimethyl-2-phenyl-3a,9b-dihydro-2H,4H-spiro[chromeno[4,3-d]isoxazole-1,11'-indeno[1,2-b]quinoxaline] (21e)

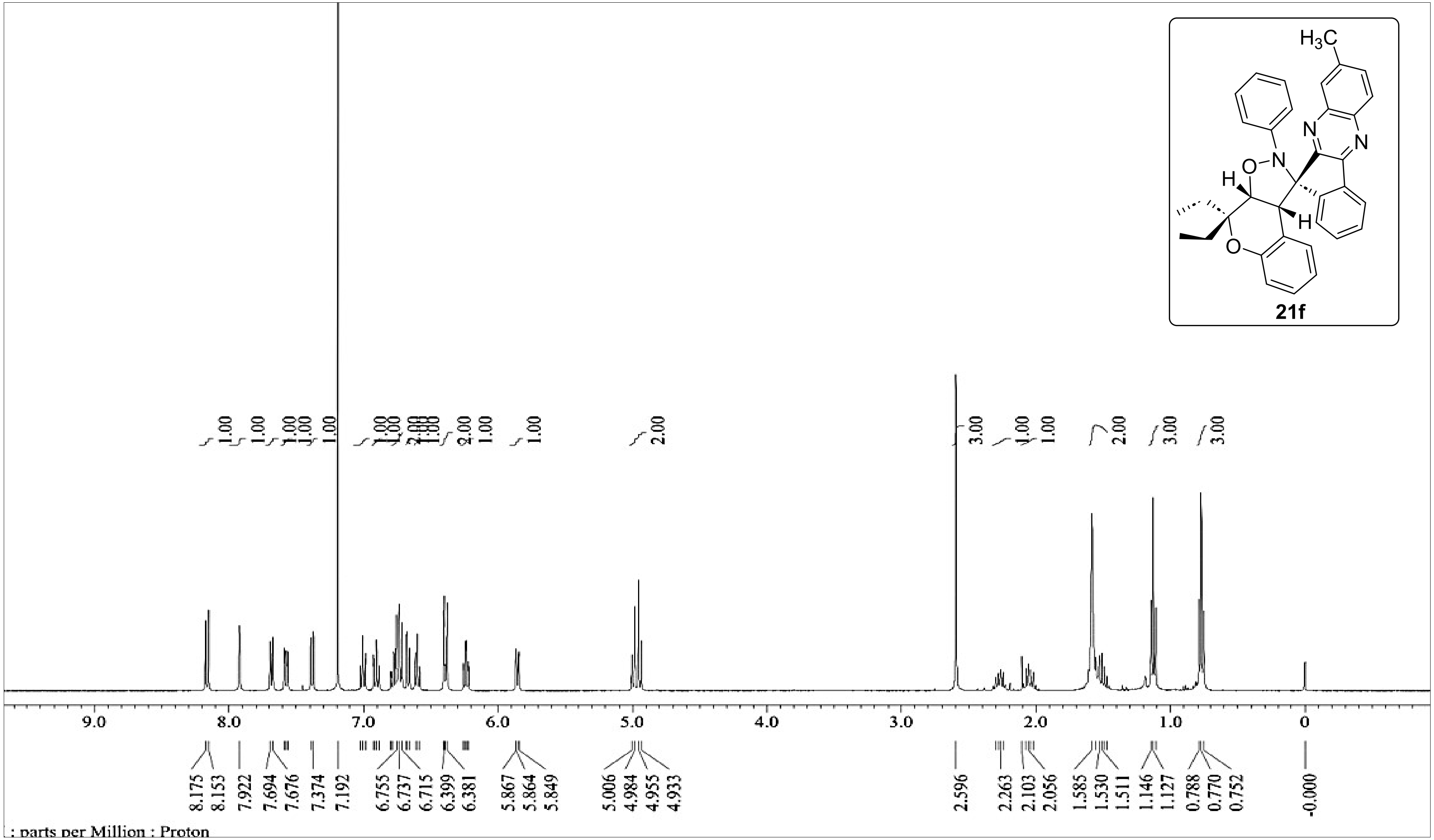

Fig. S38: ^{1}H NMR of 4,4-diethyl-8'-methyl-2-phenyl-3a,9b-dihydro-2H,4H-spiro[chromeno[4,3-d]isoxazole-1,11'-indeno[1,2-b]quinoxaline] (21f)

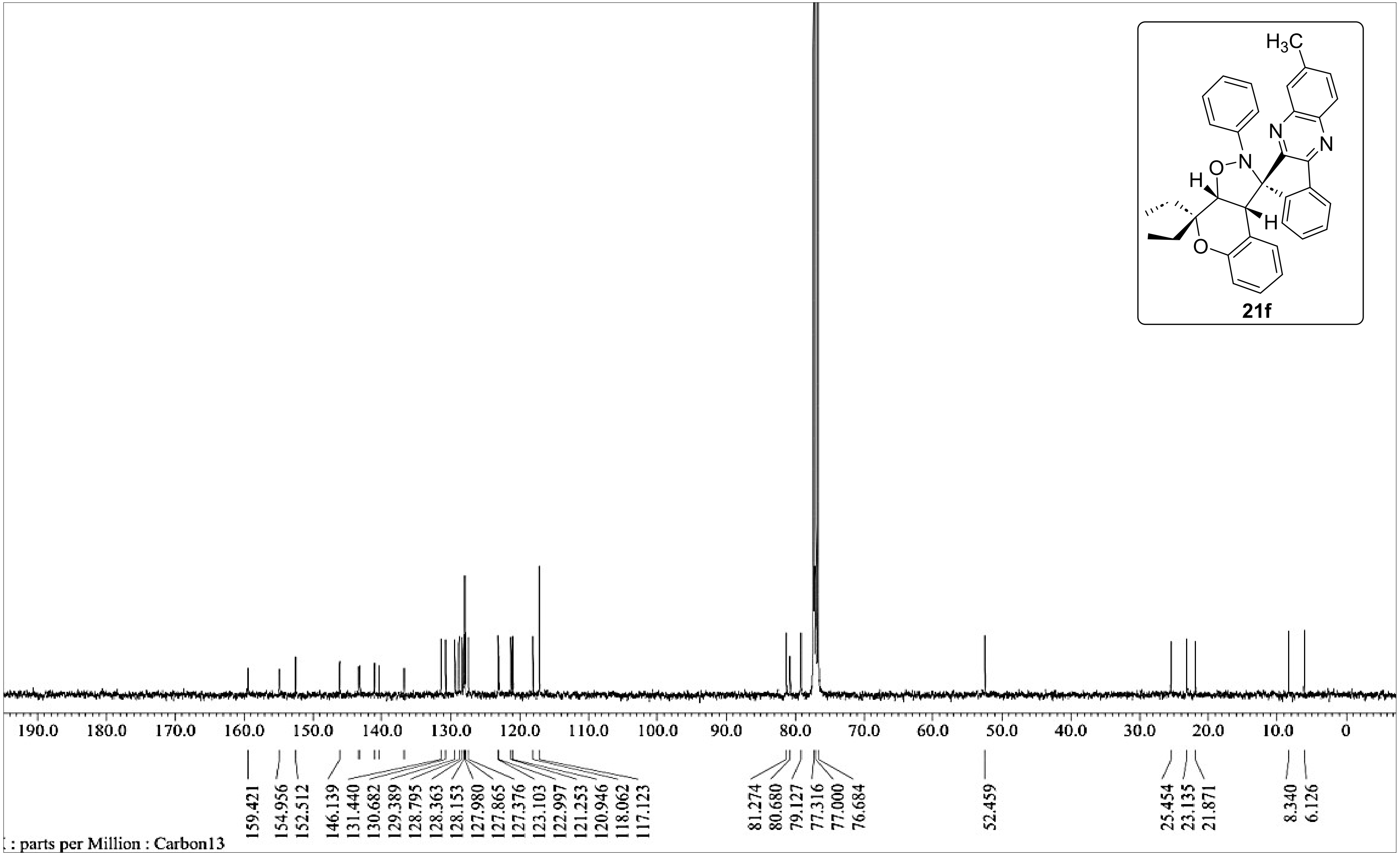

Fig. S39: ^{13}C NMR of 4,4-diethyl-8'-methyl-2-phenyl-3a,9b-dihydro-2H,4H-spiro[chromeno[4,3-d]isoxazole-1,11'-indeno[1,2-b]quinoxaline] (21f)

Compound Details

Cpd. 1: C35 H31 N3 O2

Formula	m/z	Observed M/Z	Difference_Da	Difference_PPM	Score
C35 H31 N3 O2	526.2498	526.249784642332	0.628918662982869	1.19738922116448	97.83

Compound Spectra (Zoomed)

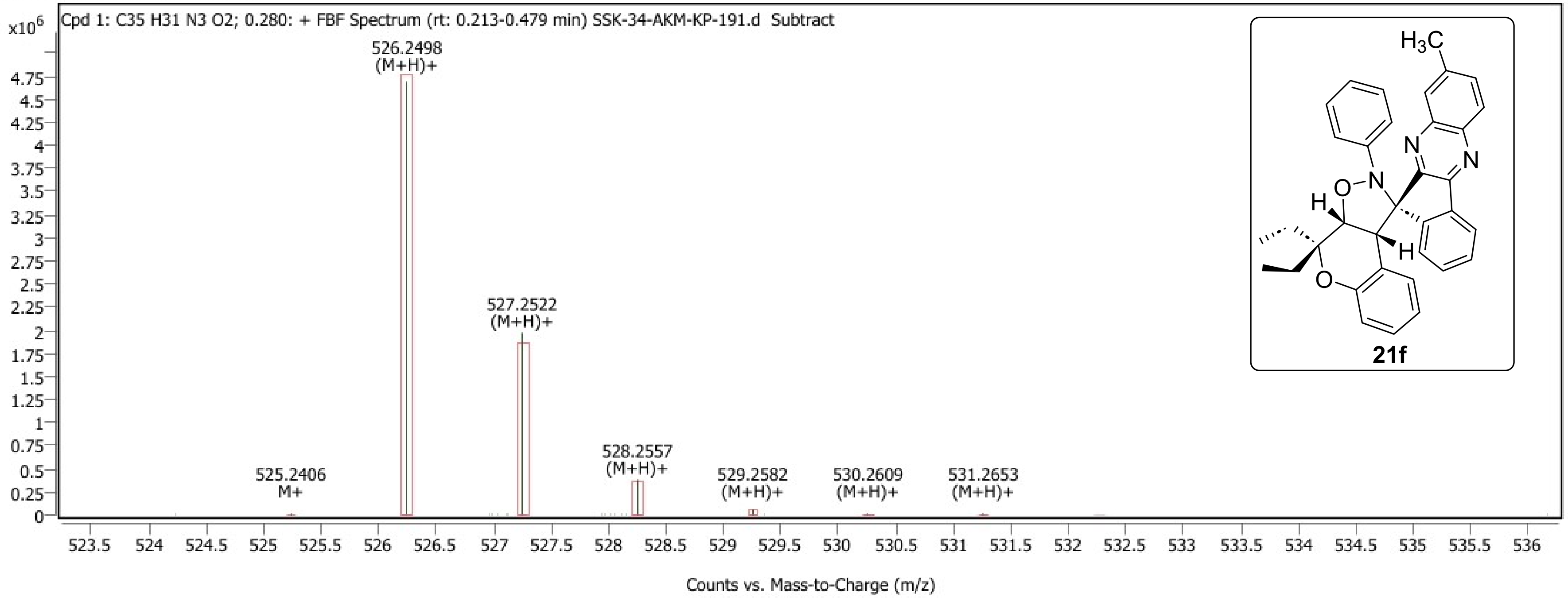

Fig. S40: HRMS of 4,4-diethyl-8'-methyl-2-phenyl-3a,9b-dihydro-2H,4H-spiro[chromeno[4,3-d]isoxazole-1,11'-indeno[1,2-b]quinoxaline] (21f)

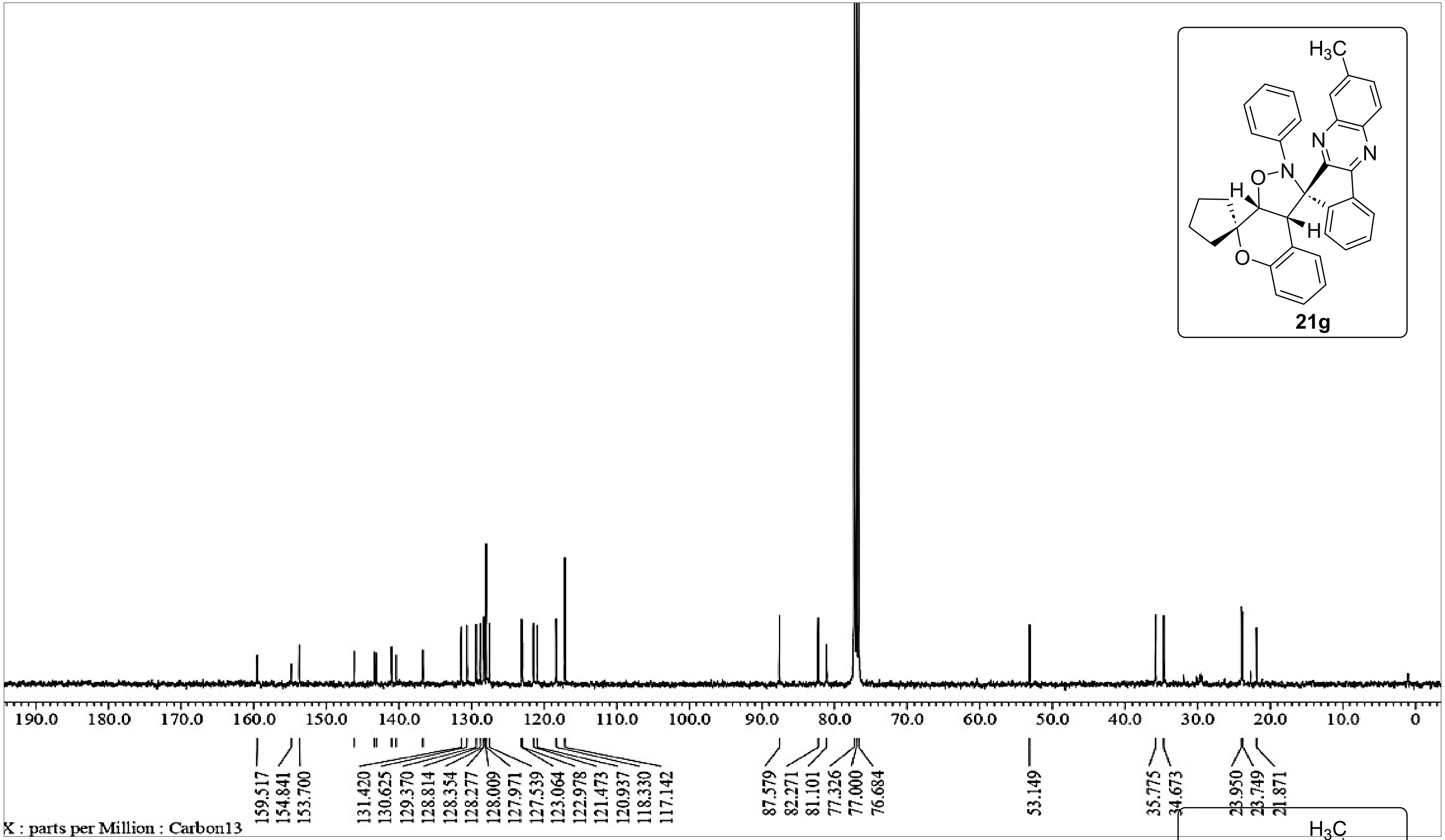

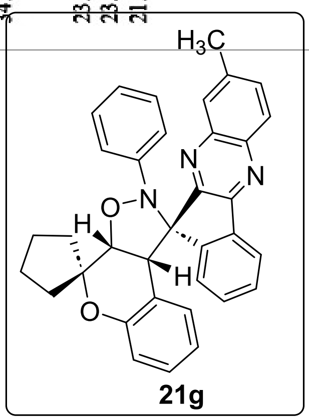

Fig. S41: ^{1}H NMR of 8''-methyl-2'-phenyl-3a',9b'-dihydro-2'H-dispiro[cyclopentane-1,4'-chromeno[4,3-d]isoxazole-1',11''-indeno[1,2 b]quinoxaline] (21g)

Compound Details

Cpd. 1: C35 H29 N3 O2

Formula	m/z	Observed M/Z	Difference_Da	Difference_PPM	Score
C35 H29 N3 O2	524.2331	524.233072117657	-0.048750661107988	-0.0931732429831328	99.48

Compound Spectra (Zoomed)

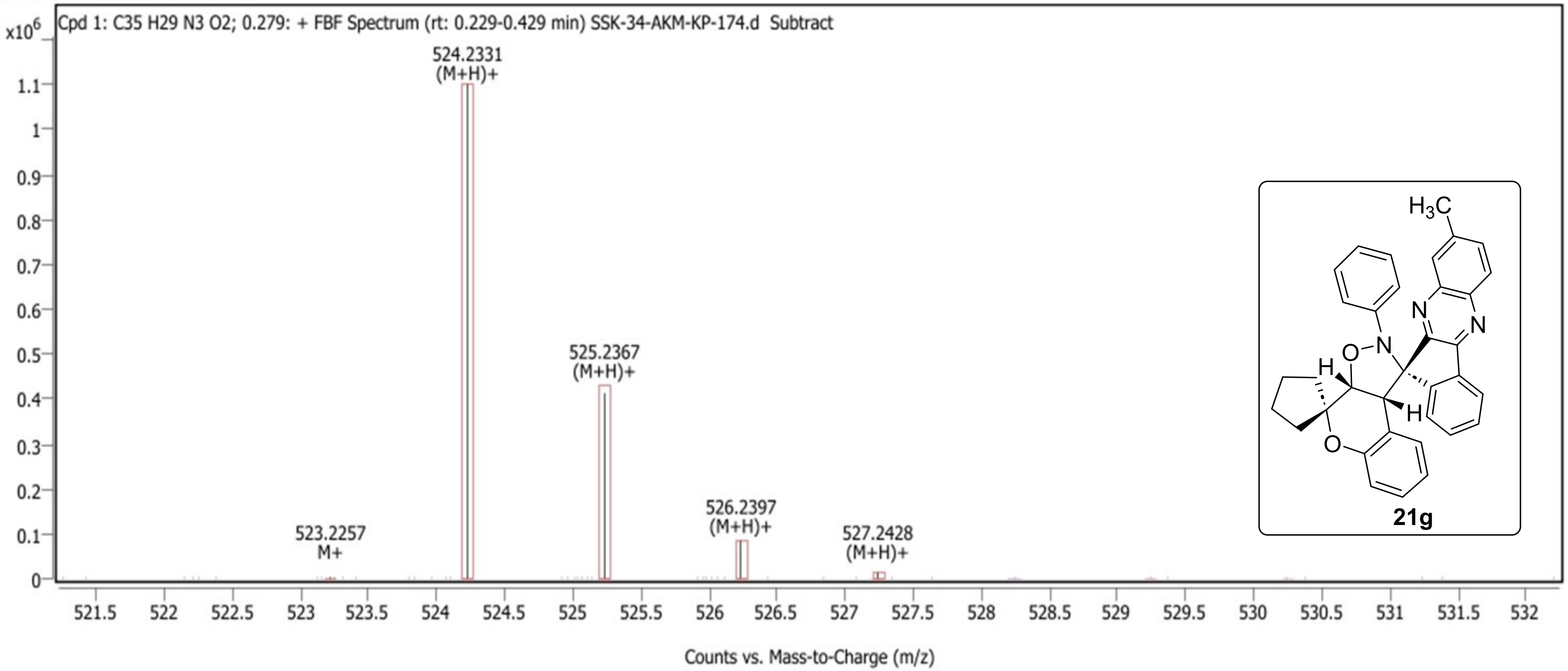

Fig. S42: ^{13}C NMR of 8''-methyl-2'-phenyl-3a',9b'-dihydro-2'H-dispiro[cyclopentane-1,4'-chromeno[4,3-d]isoxazole-1',11''indeno[1,2-b]quinoxaline] (21g)

Fig. S43: HRMS of 8''-methyl-2'-phenyl-3a',9b'-dihydro-2'H-dispiro[cyclopentane-1,4'-chromeno[4,3-d]isoxazole-1',11''-indeno[1,2-b]quinoxaline] (21g)

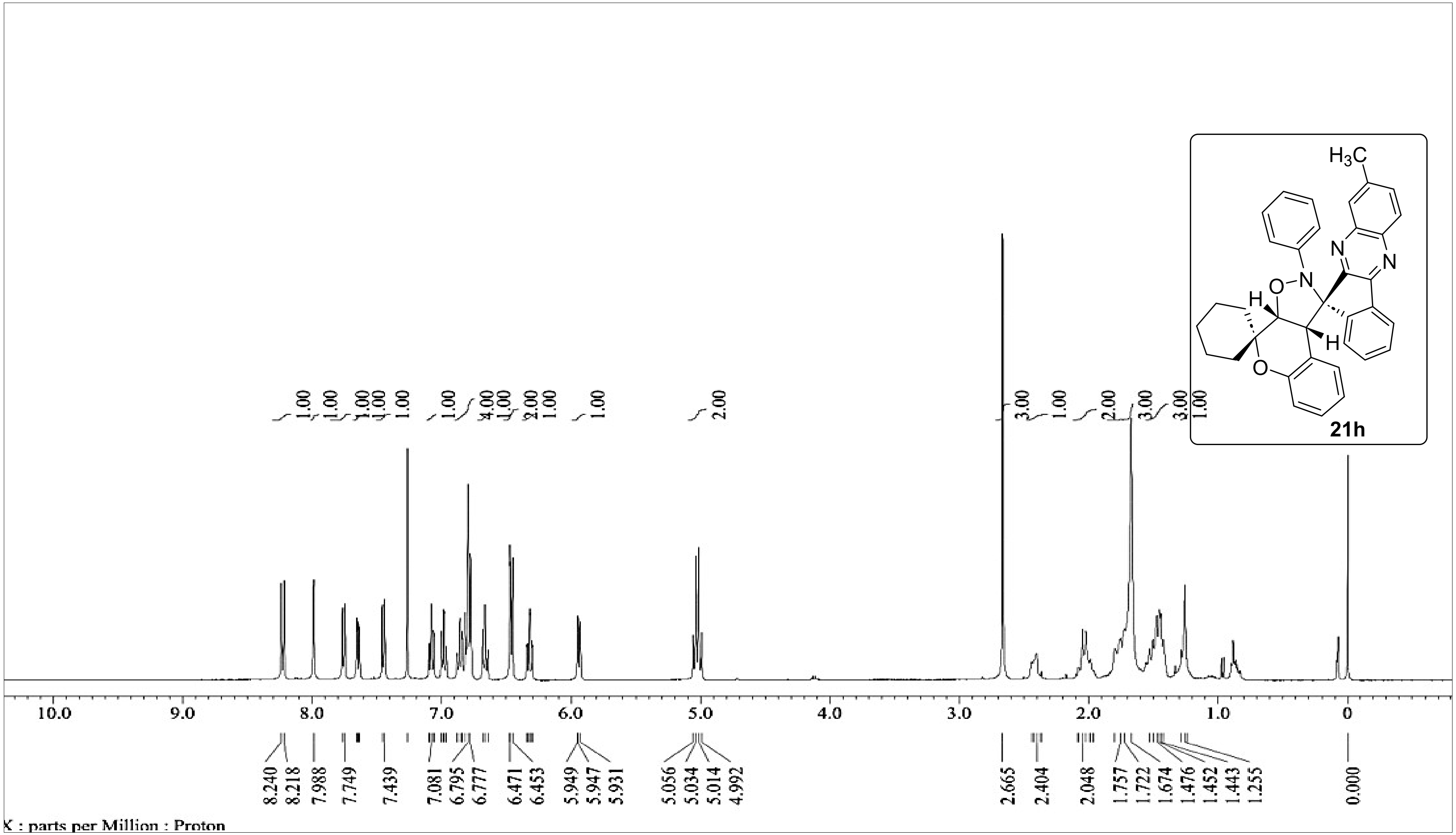

Fig. S44: ¹H NMR of 8''-methyl-2'-phenyl-3a',9b'-dihydro-2'H-dispiro[cyclohexane-1,4'-chromeno[4,3-d]isoxazole-1',11''-indeno[1,2-b]quinoxaline] (21h)

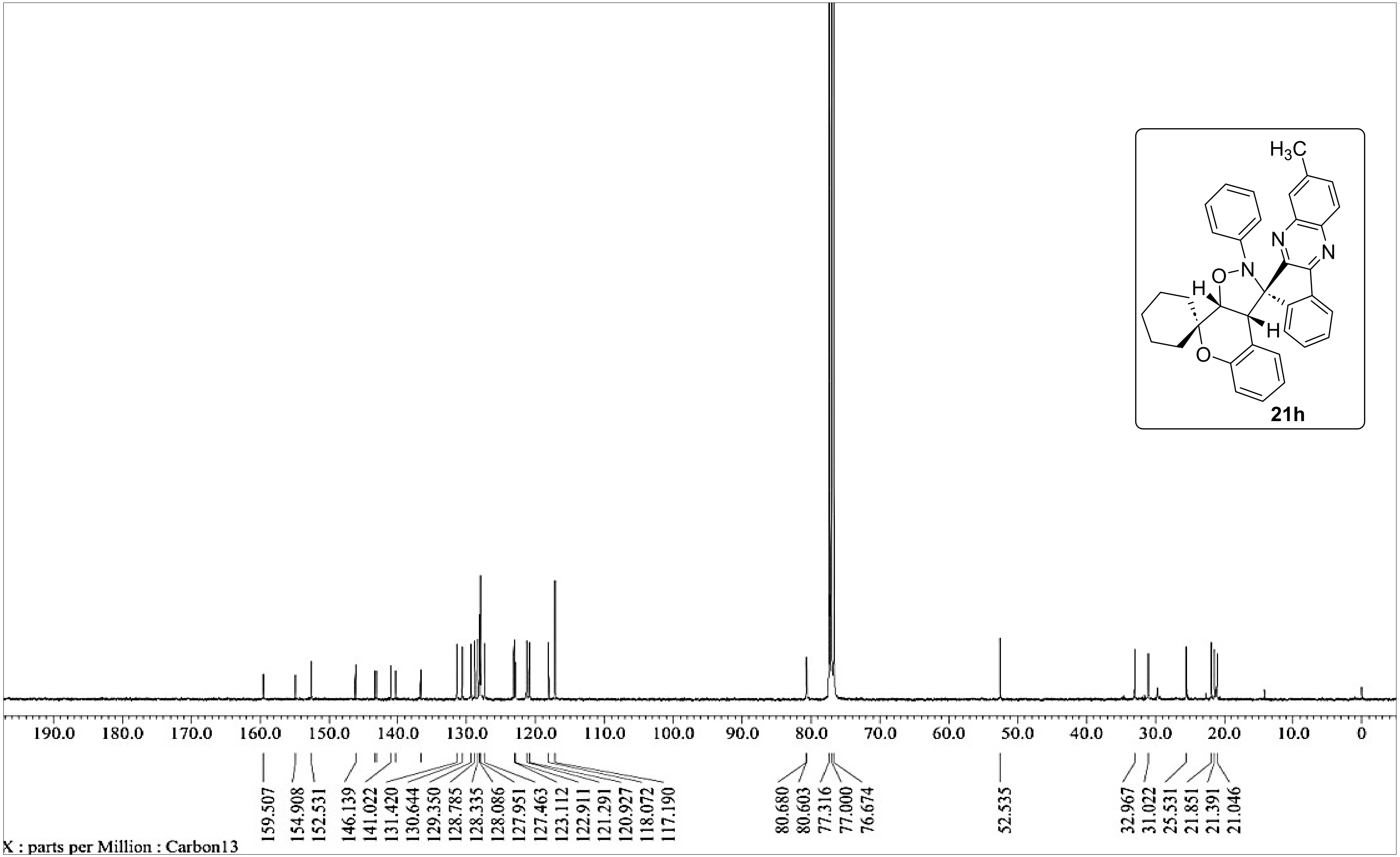

Fig. S45:^{13}C NMR of of 8''-methyl-2'-phenyl-3a',9b'-dihydro-2'H-dispiro[cyclohexane-1,4'-chromeno[4,3-d]isoxazole-1',11''- indeno[1,2-b]quinoxaline] (21h)

Compound Details

Cpd. 1: C36 H31 N3 O2

Formula	m/z	Observed M/Z	Difference_Da	Difference_PPM	Score
C36 H31 N3 O2	538.2487	538.248692871275	-0.124547566429101	-0.231827840789825	99.70

Compound Spectra (Zoomed)

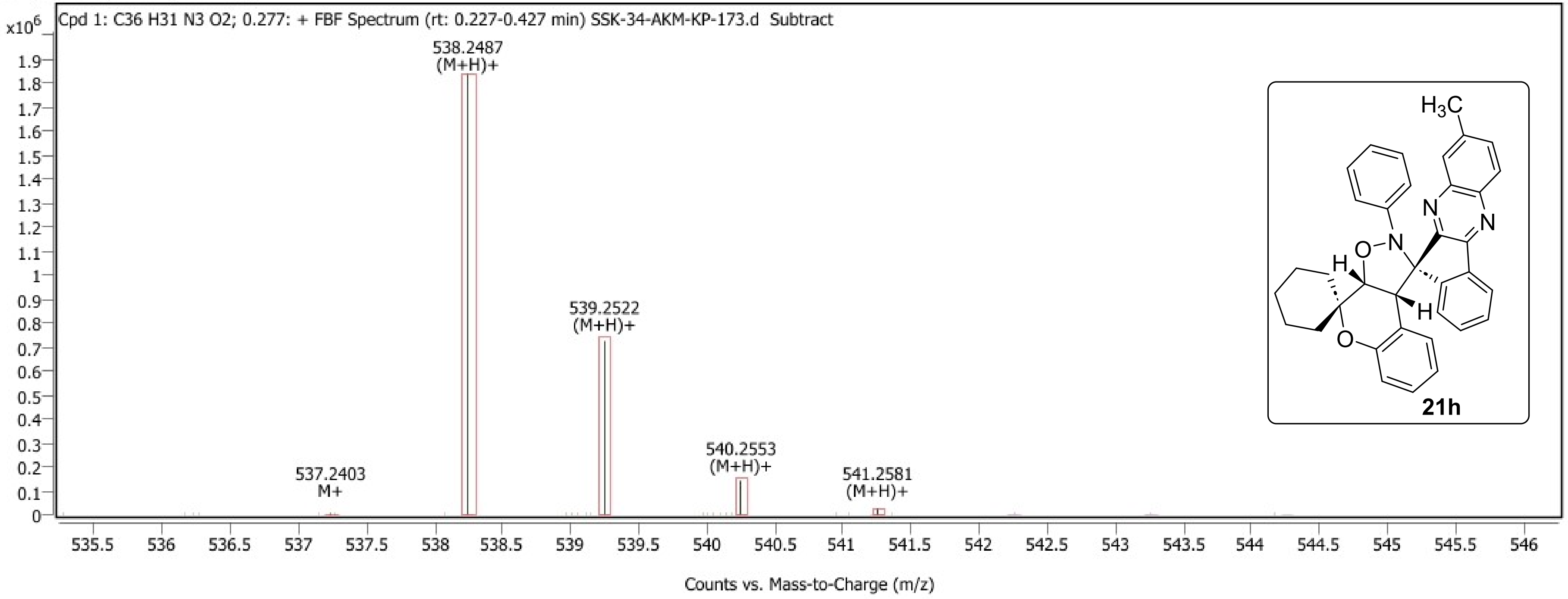

Fig. S46: HRMS of 8''-methyl-2'-phenyl-3a',9b'-dihydro-2'H-dispiro[cyclohexane-1,4'-chromeno[4,3-d]isoxazole-1',11''-indeno[1,2-b]quinoxaline] (21h)

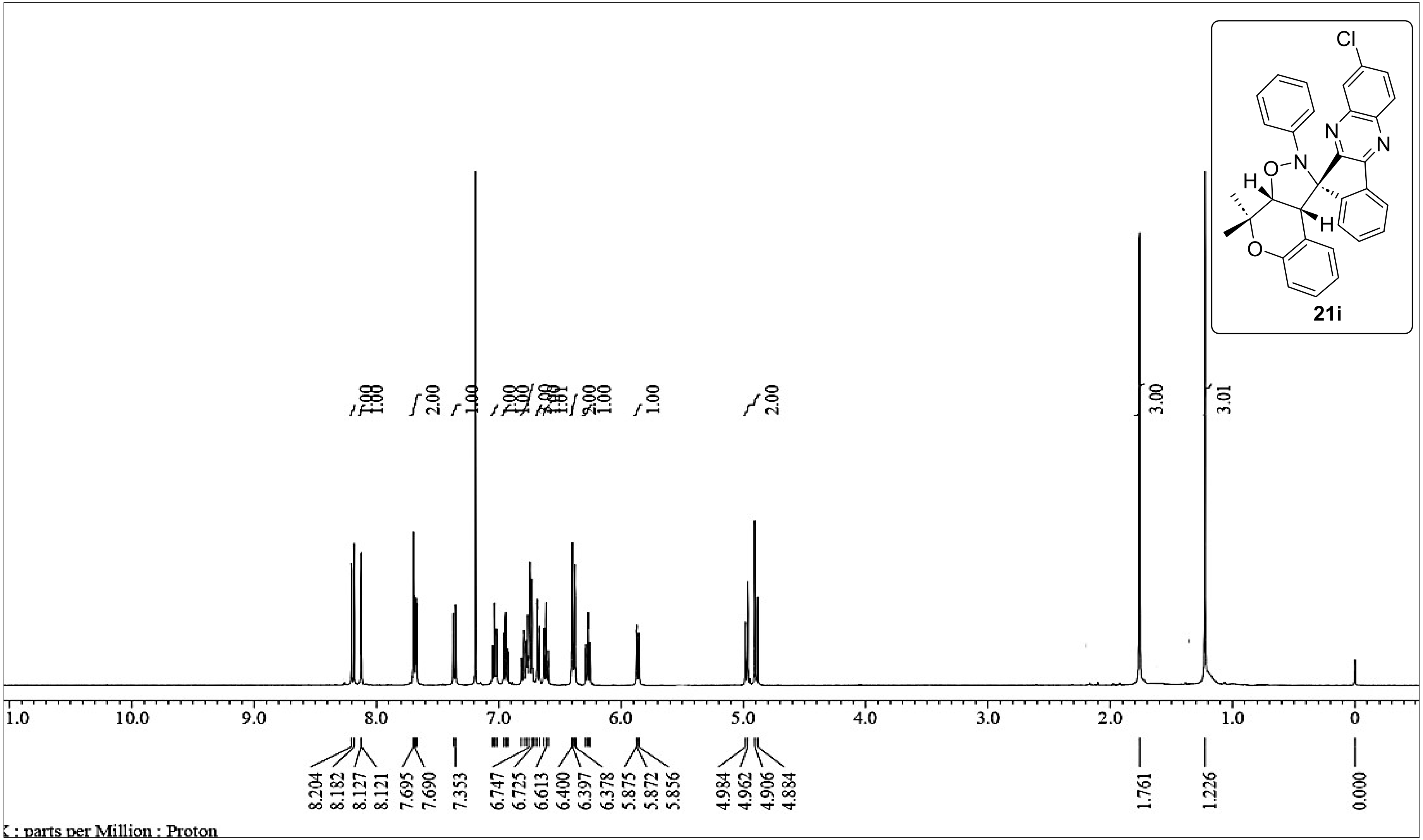

Fig. S47: ¹H NMR 8'-chloro-4,4-dimethyl-2-phenyl-3a,9b-dihydro-2H,4H-spiro[chromeno[4,3-d]isoxazole-1,11'-indeno[1,2-b]quinoxaline] (21i)

Compound Details

Cpd. 1: C32 H24 Cl N3 O2

Formula	m/z	Observed M/Z	Difference_Da	Difference_PPM	Score
C32 H24 Cl N3 O2	518.1634	518.163363898749	0.245574900191059	0.474856794460736	99.00

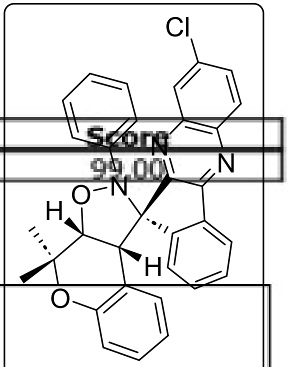

Compound Spectra (Zoomed)

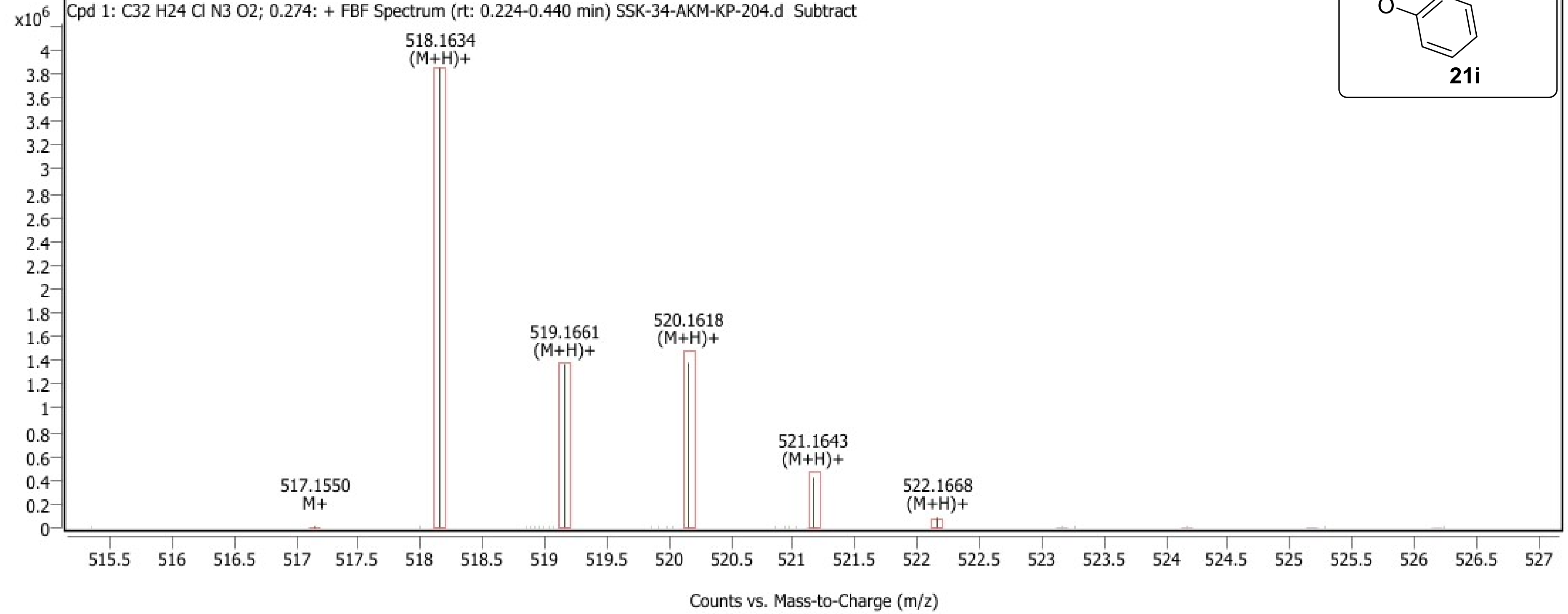

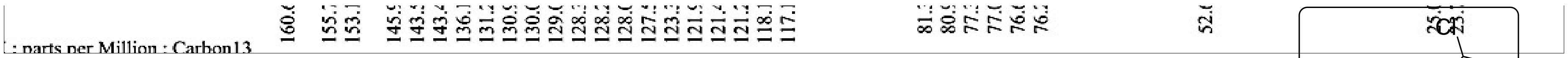

Fig. S48:^{13}C NMR 8'-chloro-4,4-dimethyl-2-phenyl-3a,9b-dihydro-2H,4H-spiro[chromeno[4,3-d]isoxazole-1,11'-indeno[1,2-b]quinoxaline] (21i)

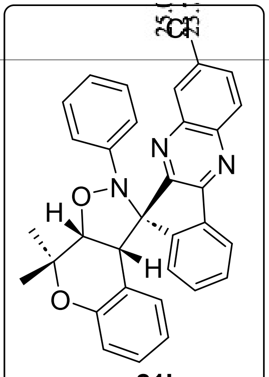

Fig. S49: HRMS of 8'-chloro-4,4-dimethyl-2-phenyl-3a,9b-dihydro-2H,4H-spiro[chromeno[4,3-d]isoxazole-1,11'-indeno[1,2-b]quinoxaline] (21i)

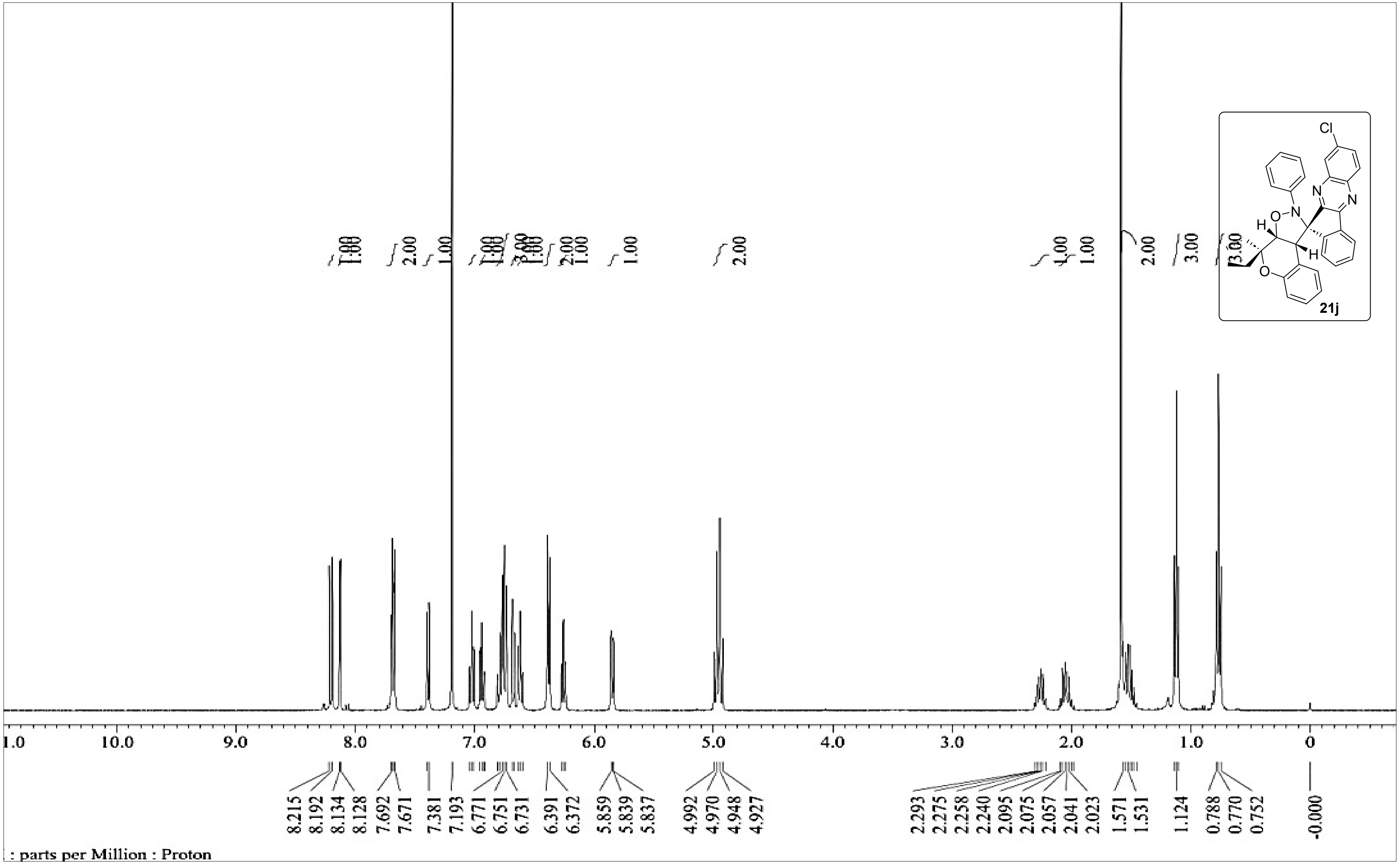

Fig. S50: ^{1}H NMR of 8'-chloro-4,4-diethyl-2-phenyl-3a,9b-dihydro-2H,4H-spiro[chromeno[4,3-d]isoxazole-1,11'-indeno[1,2-b]quinoxaline] (21j)

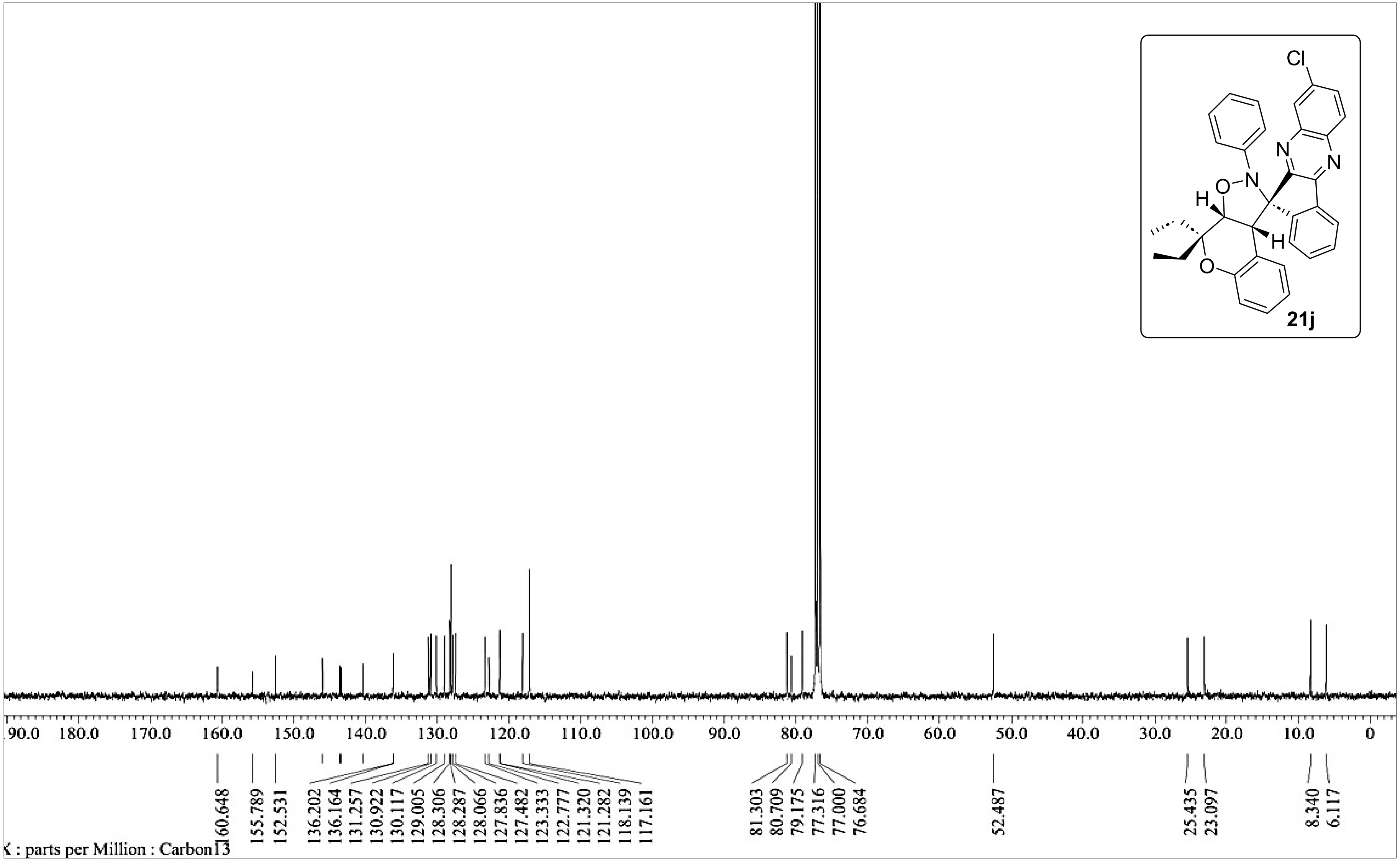

Fig. S51: ^{13}**C NMR of 8'-chloro-4,4-diethyl-2-phenyl-3a,9b-dihydro-2H,4H-spiro[chromeno[4,3-d]isoxazole-1,11'-indeno[1,2-b]quinoxaline] (21j)**

Compound Details

Cpd. 1: C34 H28 Cl N3 O2

Formula	m/z	Observed M/Z	Difference_Da	Difference_PPM	Score
C34 H28 Cl N3 O2	546.1942	546.194229241026	-0.0294728955623214	-0.0540601578890333	98.78

Compound Spectra (Zoomed)

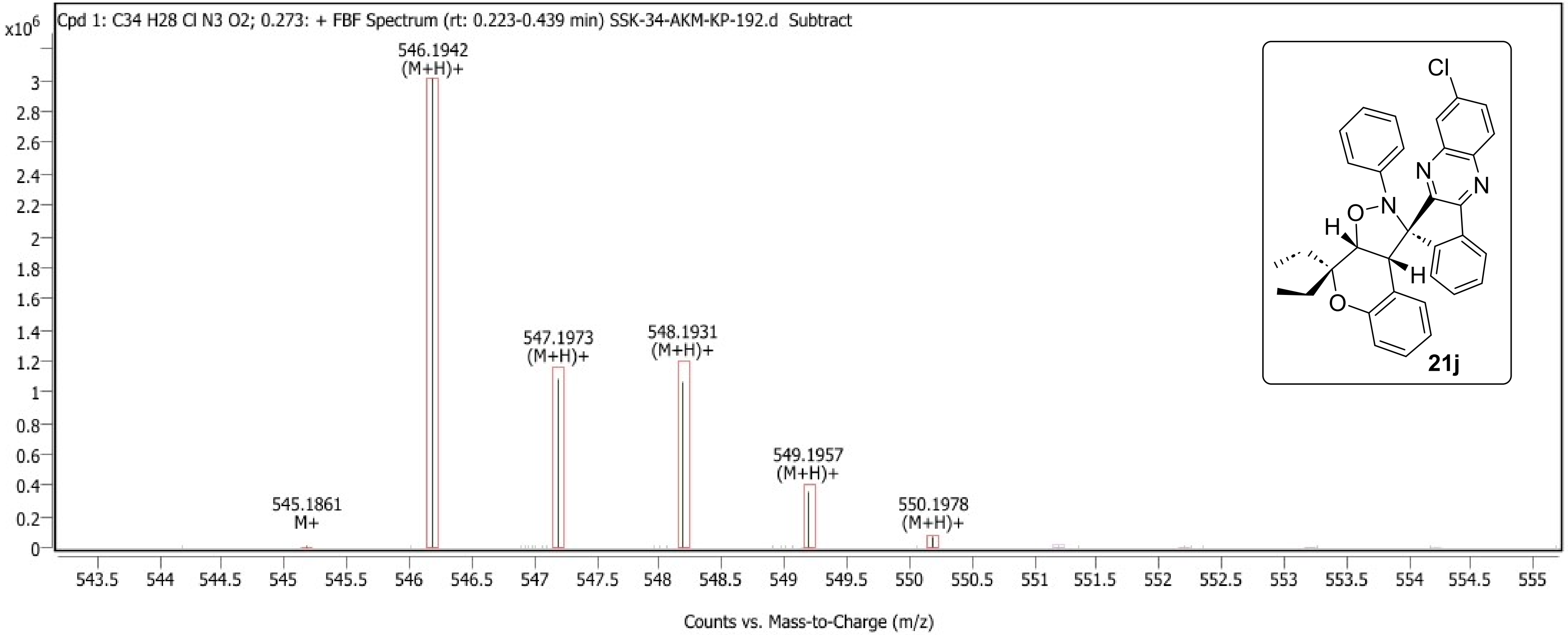

Fig. S52: HRMS of 8'-chloro-4,4-diethyl-2-phenyl-3a,9b-dihydro-2H,4H-spiro[chromeno[4,3-d]isoxazole-1,11'-indeno[1,2-b]quioxaline] (21j)

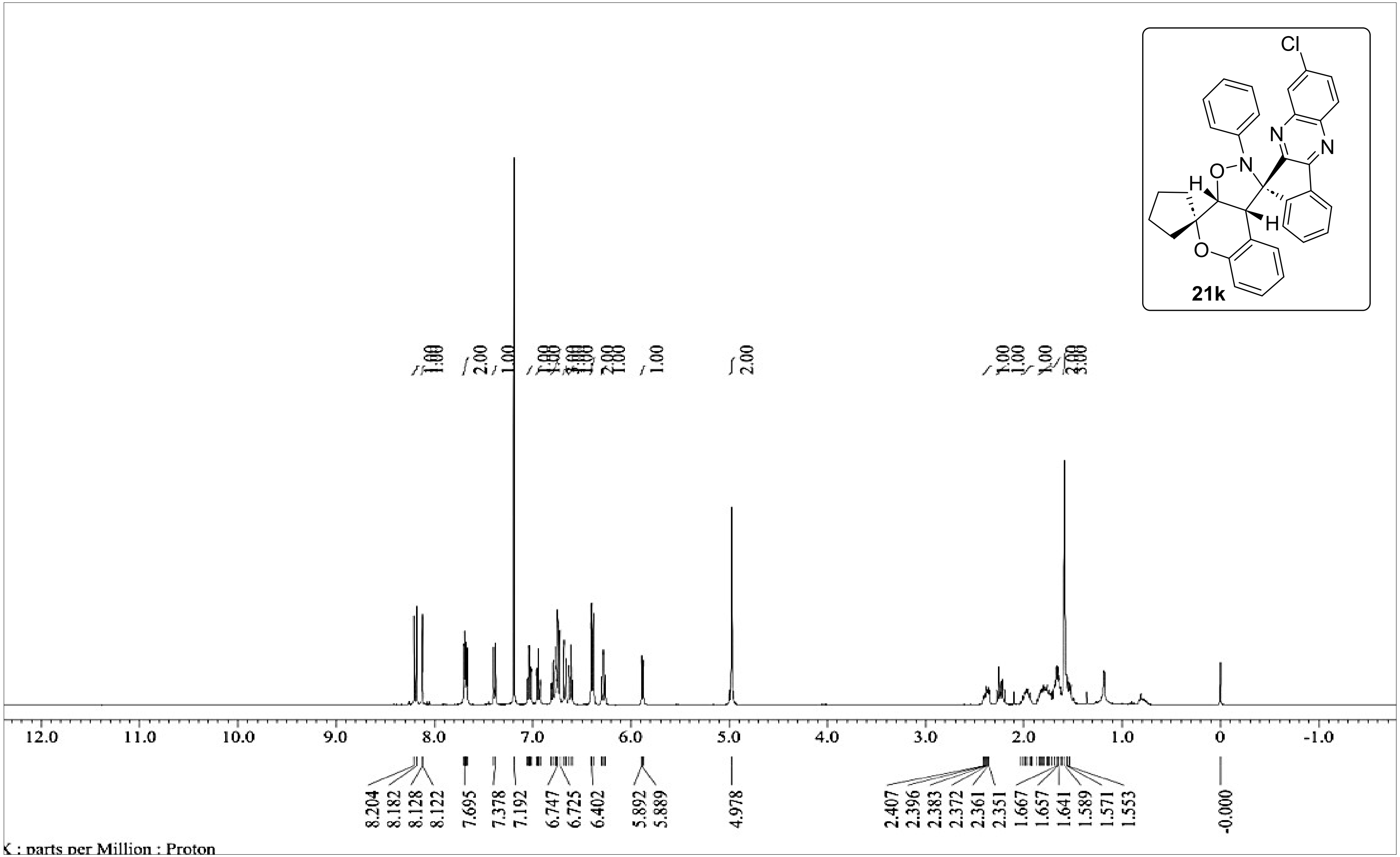

Fig. S53: ^{1}H NMR of 8''-chloro-2'-phenyl-3a',9b'-dihydro-2'H-dispiro[cyclopentane-1,4'-chromeno[4,3-d]isoxazole-1',11''-indeno[1,2-b]quinoxaline] (21k)

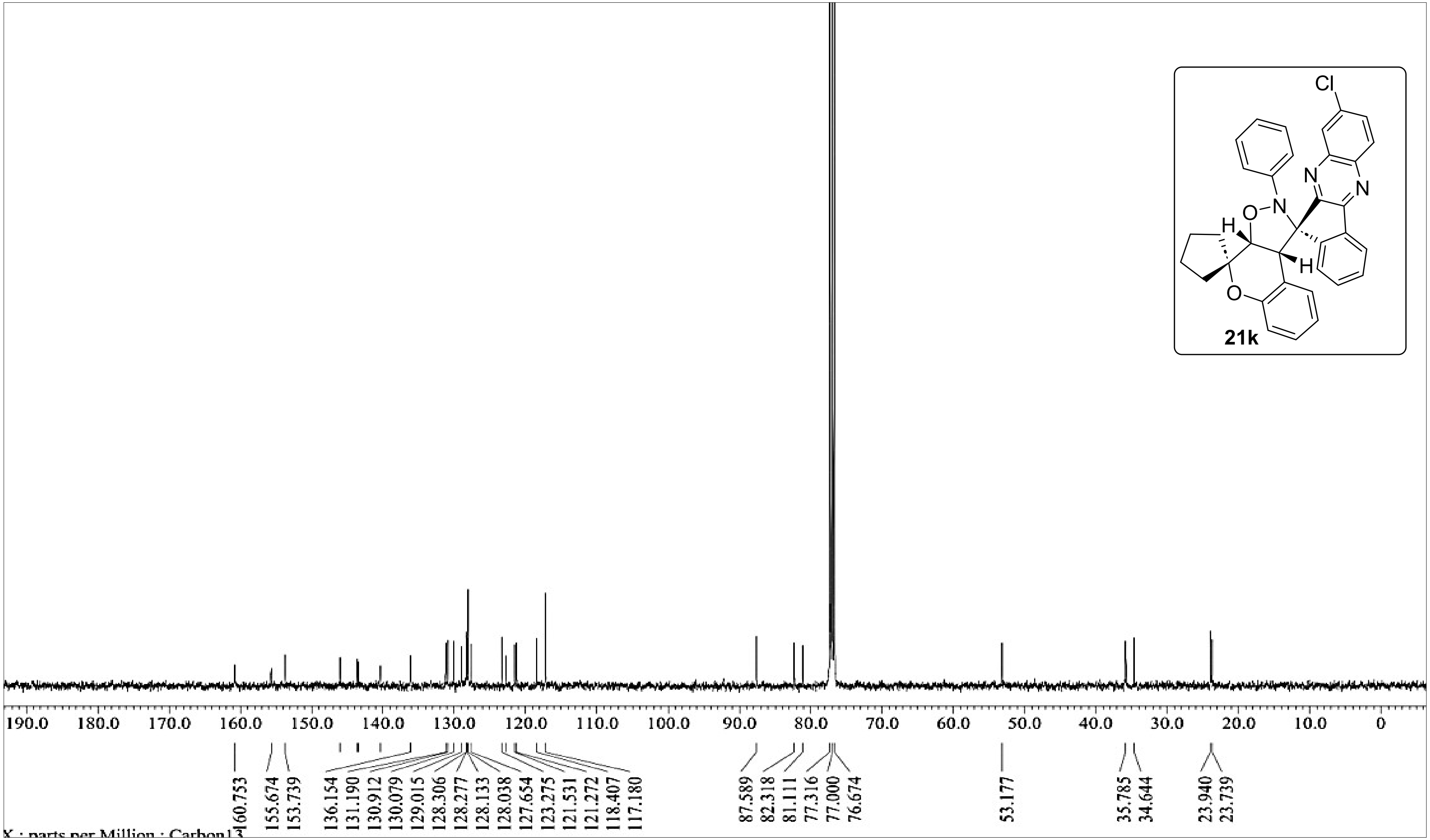

Fig. S54: ^{13}C NMR of 8''-chloro-2'-phenyl-3a',9b'-dihydro-2'H-dispiro[cyclopentane-1,4'-chromeno[4,3-d]isoxazole-1',11''-indeno[1,2-b]quinoxaline] (21k)

Compound Details

Cpd. 1: C34 H26 Cl N3 O2

Formula	m/z	Observed M/Z	Difference_Da	Difference_PPM	Score
C34 H26 Cl N3 O2	544.1783	544.178278119671	-0.521198948263191	-0.959547928386524	98.63

Compound Spectra (Zoomed)

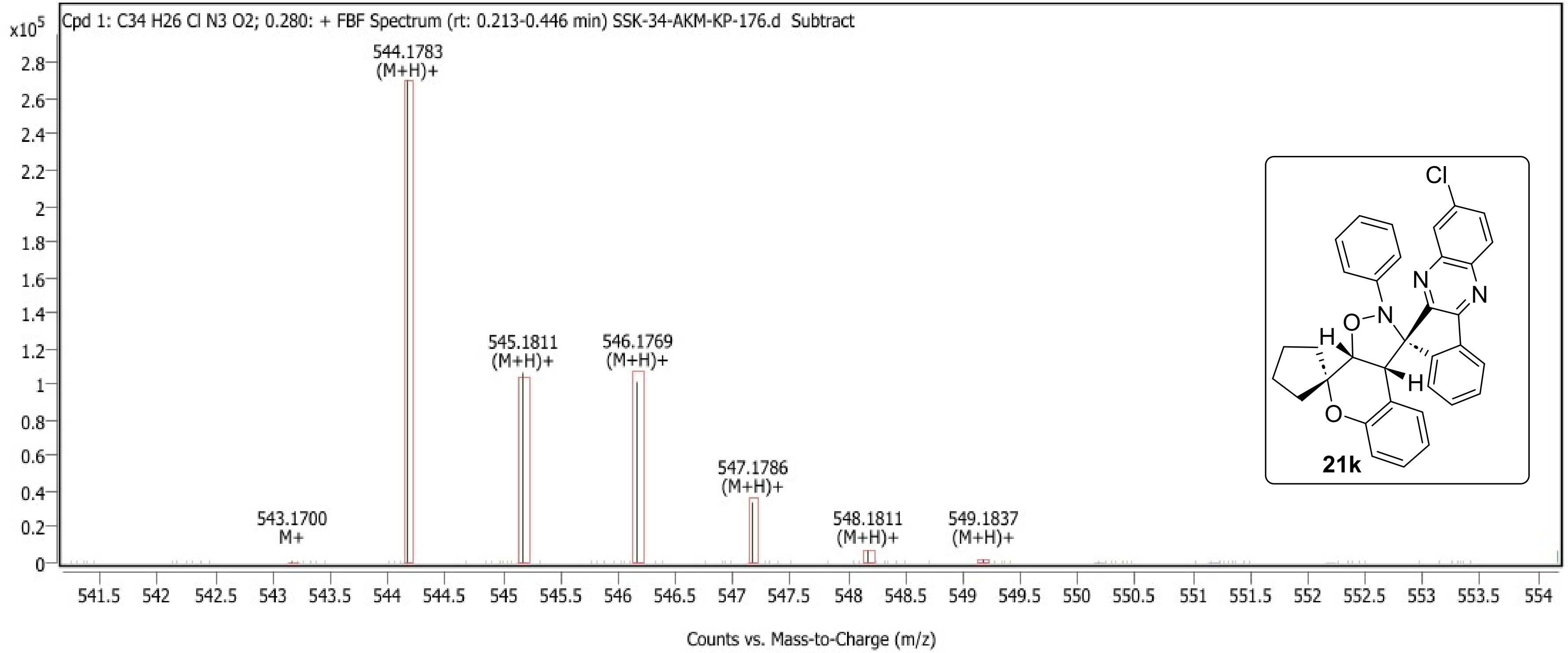

Fig. S55: HRMS of 8''-chloro-2'-phenyl-3a',9b'-dihydro-2'H-dispiro[cyclopentane-1,4'-chromeno[4,3-d]isoxazole-1',11''-indeno[1,2-b]quinoxaline] (21k)

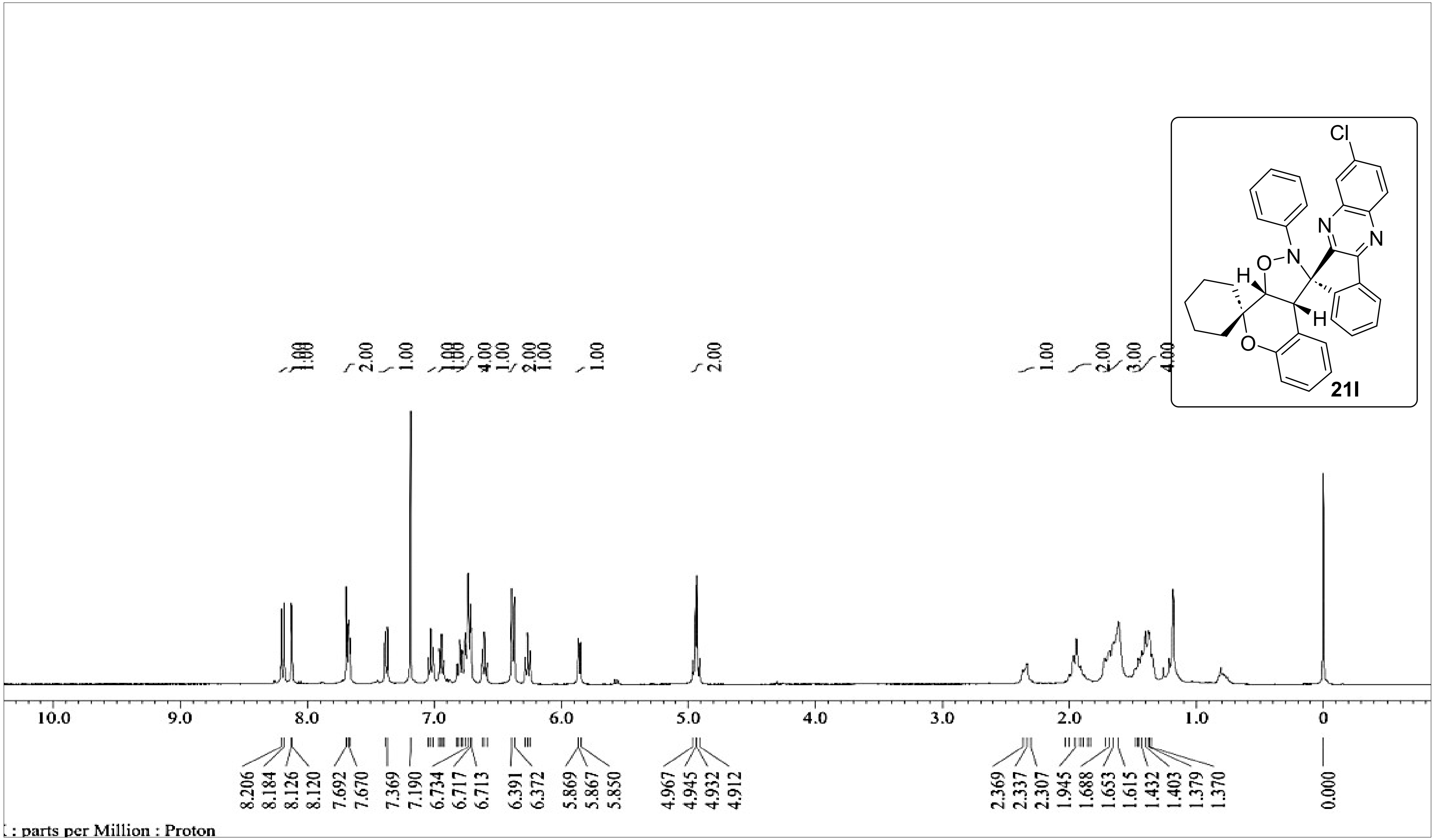

Cl
N
N
O
N
H
H
O
21l
1.00
2.00
1.00
4.00
1.00
2.00
1.00
1.00
2.00
1.00
2.00
3.00
4.00
10.0
9.0
8.0
7.0
6.0
5.0
4.0
3.0
2.0
1.0
0
8.206
8.184
8.126
8.120
7.692
7.670
7.369
7.190
6.734
6.717
6.713
6.391
6.372
5.869
5.867
5.850
4.967
4.945
4.932
4.912
2.369
2.337
2.307
1.945
1.688
1.653
1.615
1.432
1.403
1.379
1.370
0.000
: parts per Million : Proton

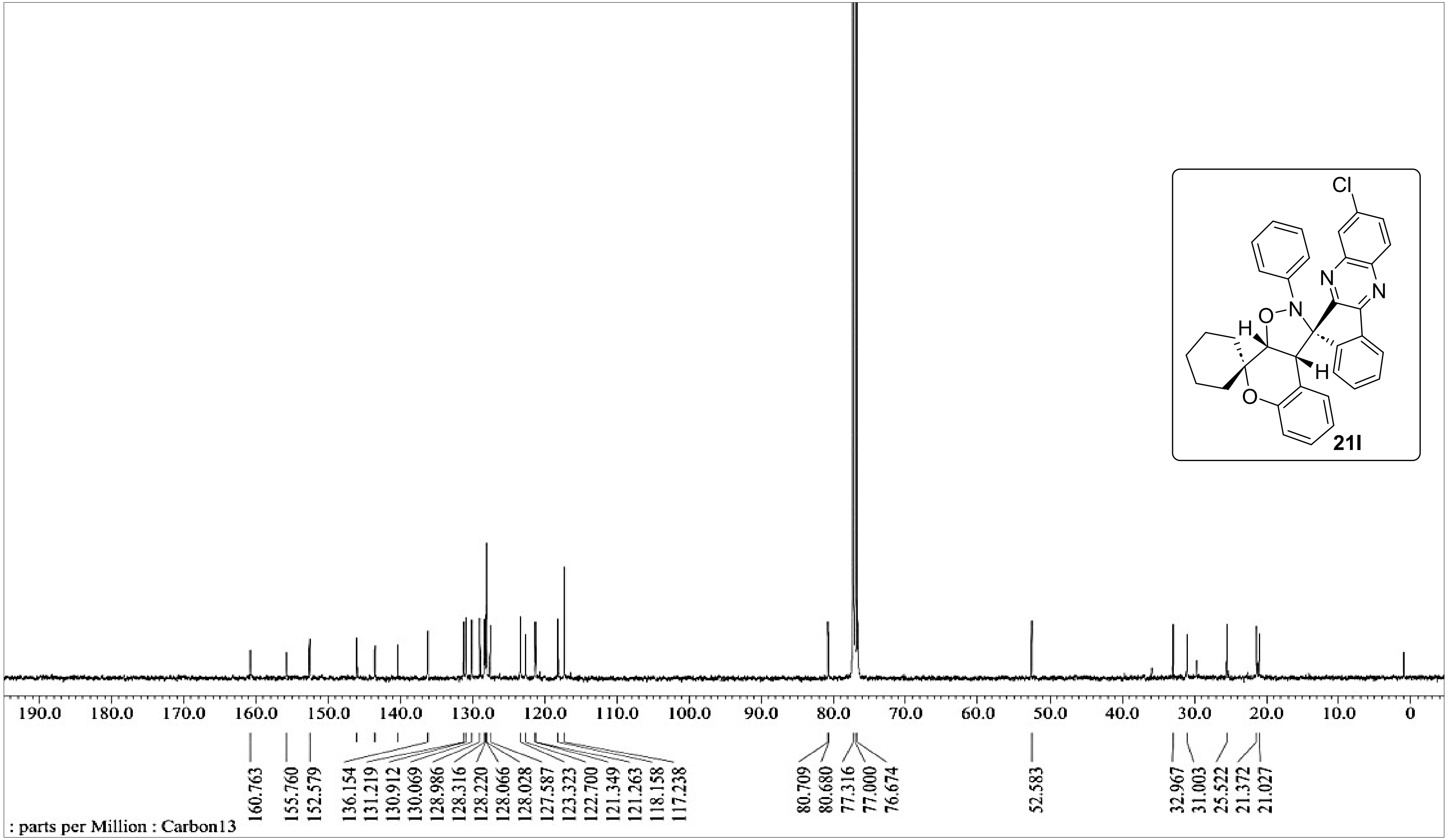

Fig. S56: ^{1}H NMR of 8''-chloro-2'-phenyl-3a',9b'-dihydro-2'H-dispiro[cyclohexane-1,4'-chromeno[4,3-d]isoxazole-1',11''-indeno[1,2-b]quinoxaline] (21l)

Fig. S57: ^{13}C NMR of 8''-chloro-2'-phenyl-3a',9b'-dihydro-2'H-dispiro[cyclohexane-1,4'-chromeno[4,3-d]isoxazole-1',11''-indeno[1,2-b]quinoxaline] (21l)

Compound Details

Cpd. 1: C35 H28 Cl N3 O2

Formula	m/z	Observed M/Z	Difference_Da	Difference_PPM	Score
C35 H28 Cl N3 O2	558.1946	558.194591207979	0.352701157680713	0.633003201076097	98.25

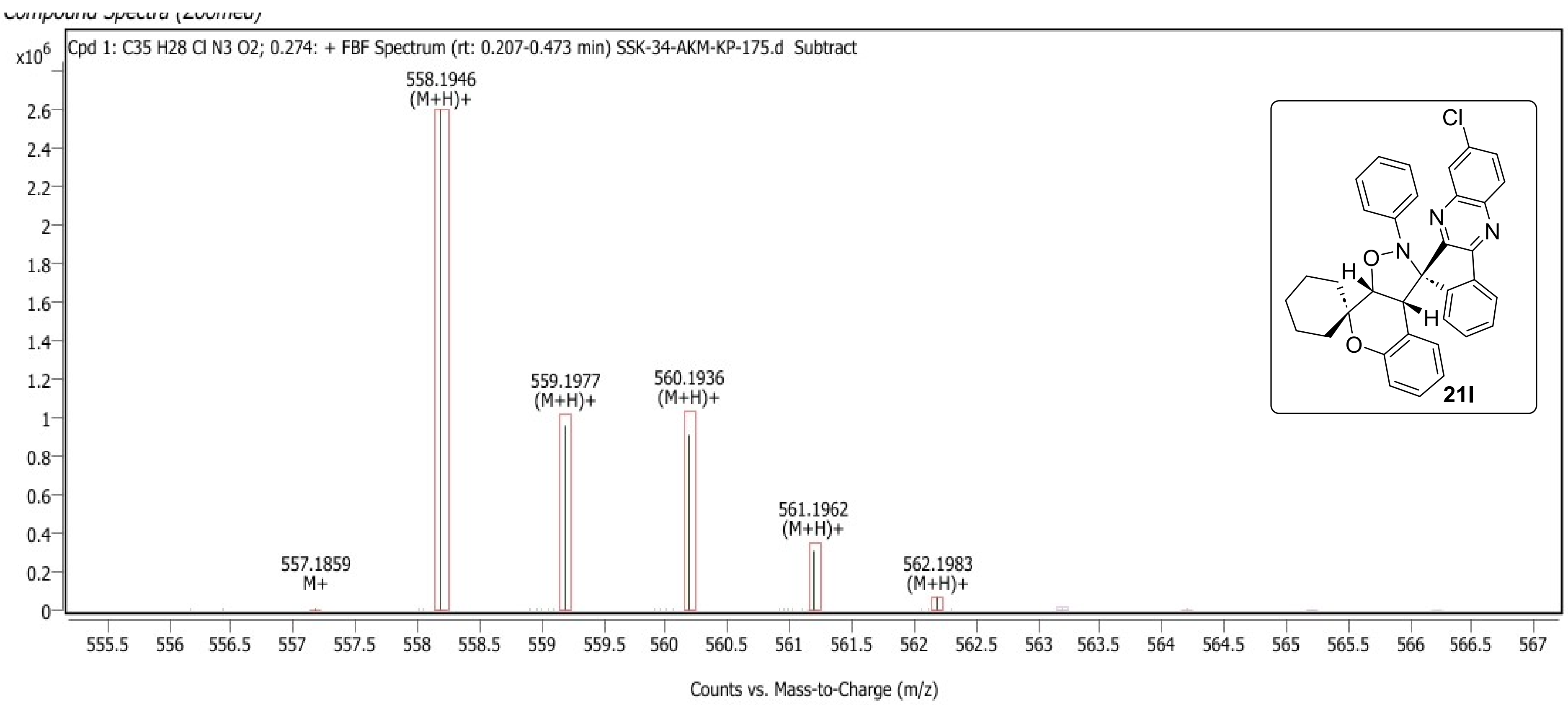

Fig. S58: HRMS of 8''-chloro-2'-phenyl-3a',9b'-dihydro-2'H-dispiro[cyclohexane-1,4'-chromeno[4,3-d]isoxazole-1',11''-indeno[1,2-b]quinoxaline] (21l)

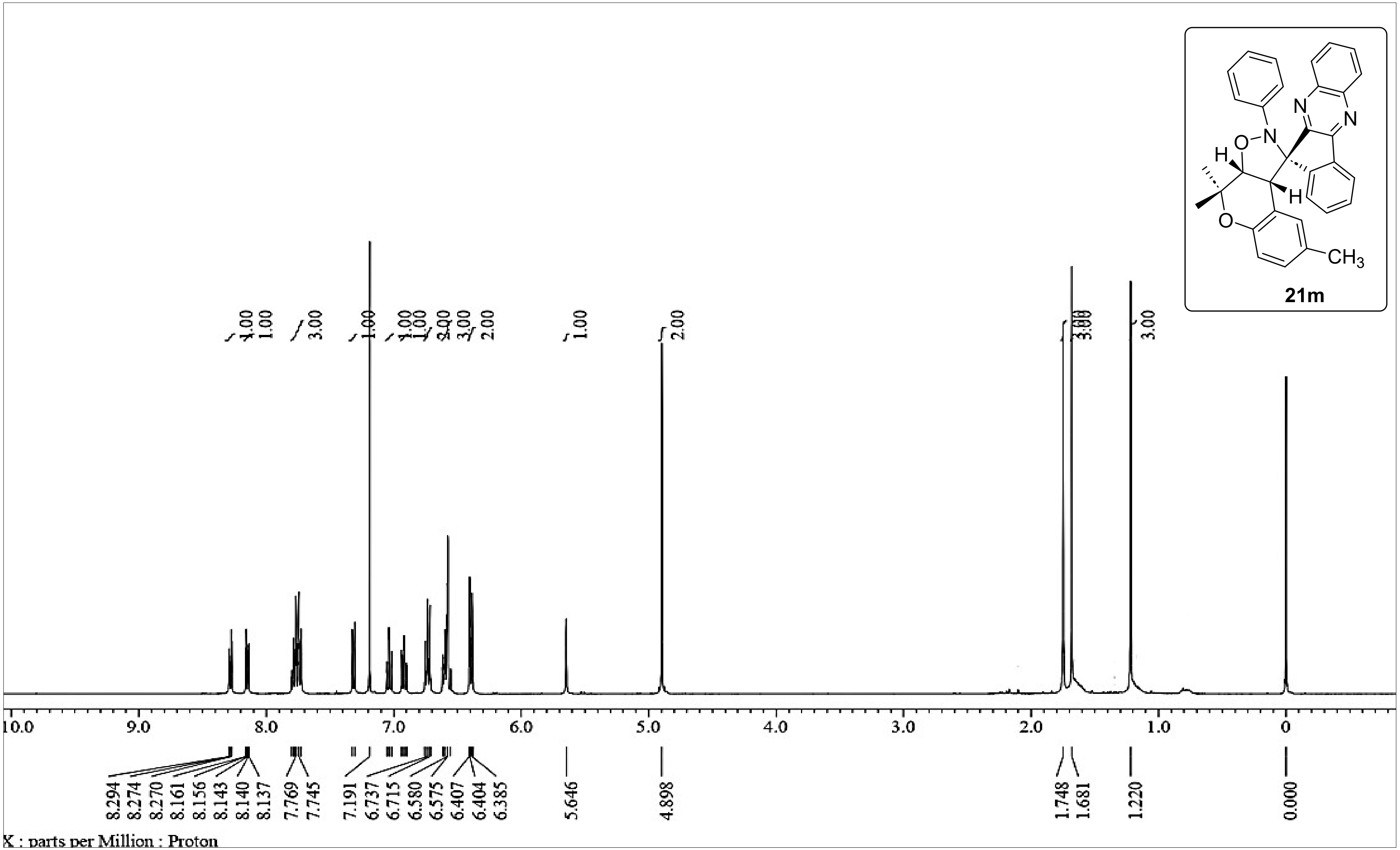

Fig. S59: ^{1}H NMR of 4,4,8-trimethyl-2-phenyl-3a,9b-dihydro-2H,4H-spiro[chromeno[4,3-d]isoxazole-1,11'-indeno[1,2-b]quinoxaline] (21m)

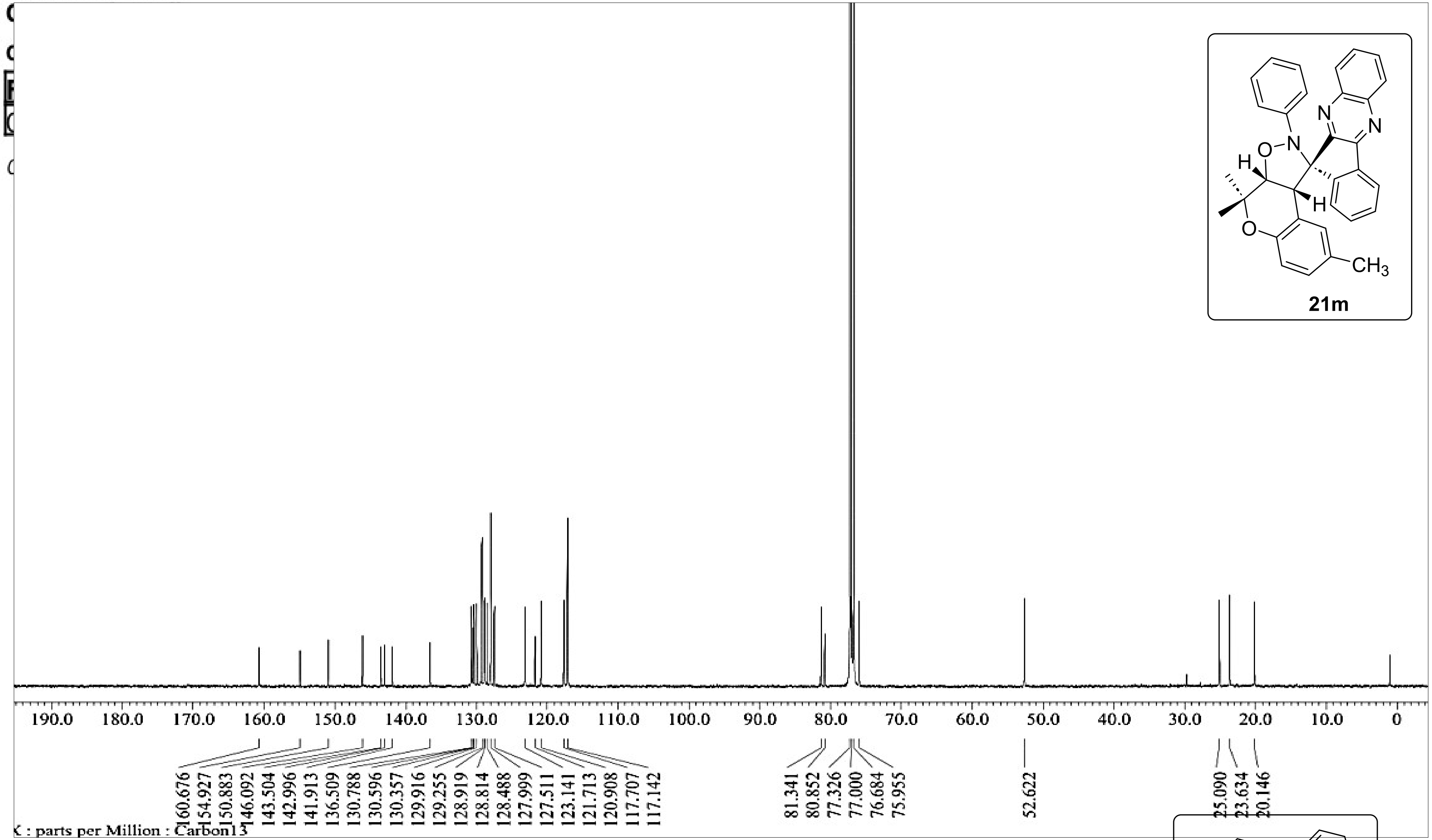

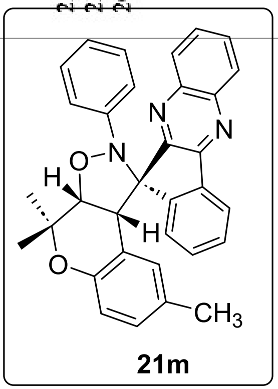

Fig. S60: ^{13}C NMR of 4,4,8-trimethyl-2-phenyl-3a,9b-dihydro-2H,4H-spiro[chromeno[4,3-d]isoxazole-1,11'-indeno[1,2-b]quinoxaline] (21m)

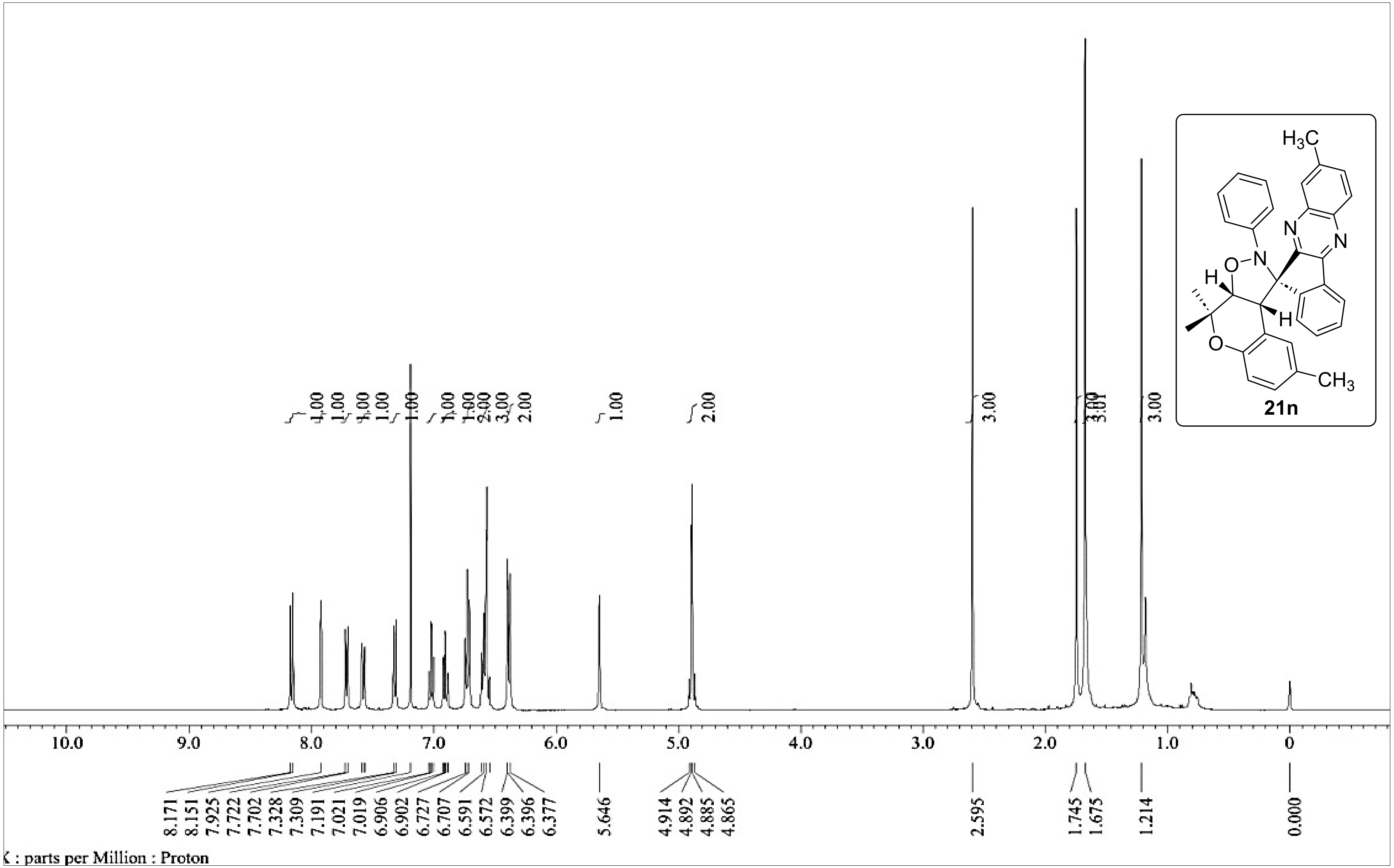

Fig. S61: HRMS of 4,4,8-trimethyl-2-phenyl-3a,9b-dihydro-2H,4H-spiro[chromeno[4,3-d]isoxazole-1,11'-indeno[1,2-b]quinoxaline] (21m)

Fig. S62: ^{1}H NMR of 4,4,8,8'-tetramethyl-2-phenyl-3a,9b-dihydro-2H,4H-spiro[chromeno[4,3-d]isoxazole-1,11'-indeno[1,2-b]quinoxaline] (21n)

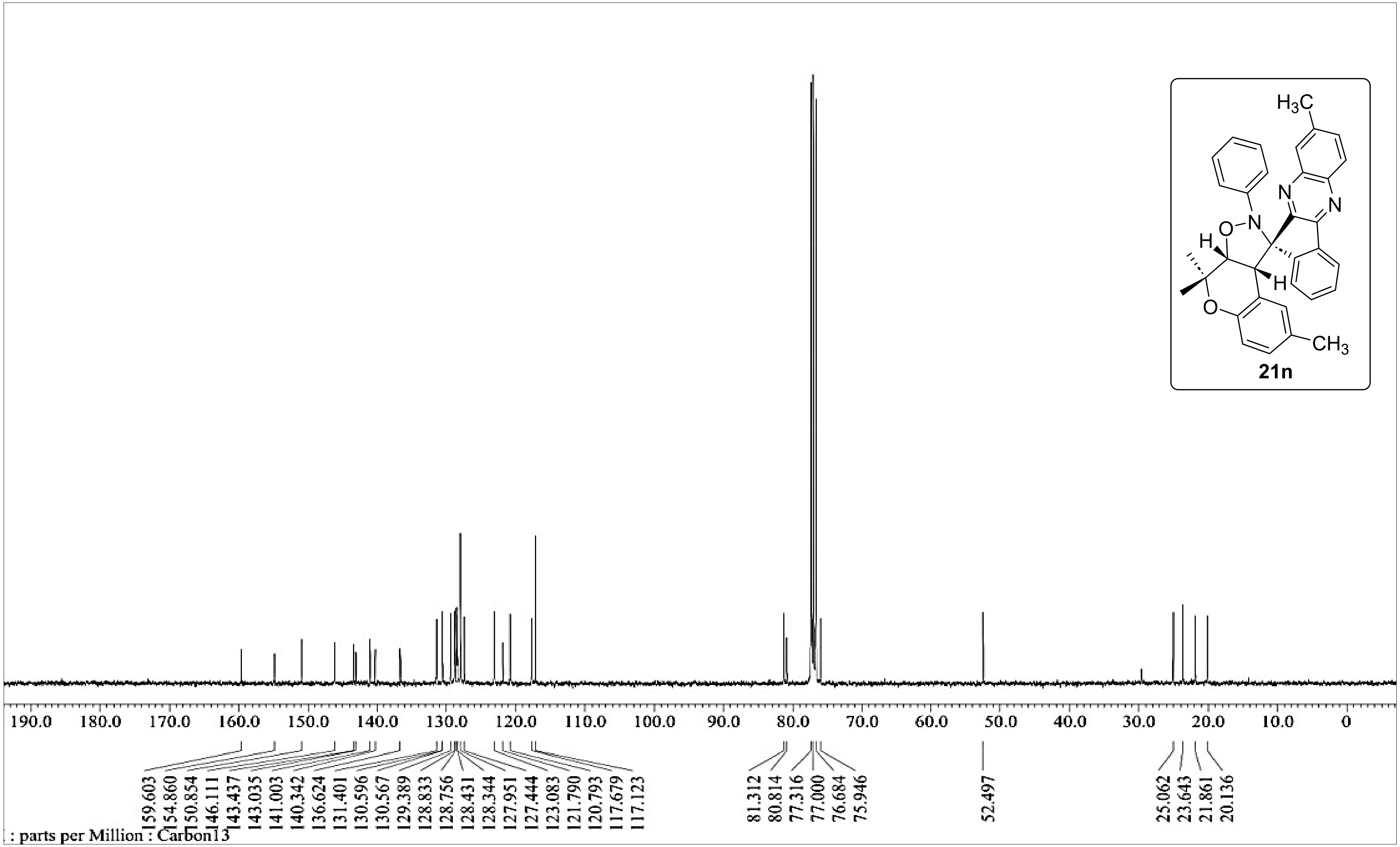

Fig. S63: ^{13}C NMR of 4,4,8,8'-tetramethyl-2-phenyl-3a,9b-dihydro-2H,4H-spiro[chromeno[4,3-d]isoxazole-1,11'-indeno[1,2-b]quinoxaline] (21n)

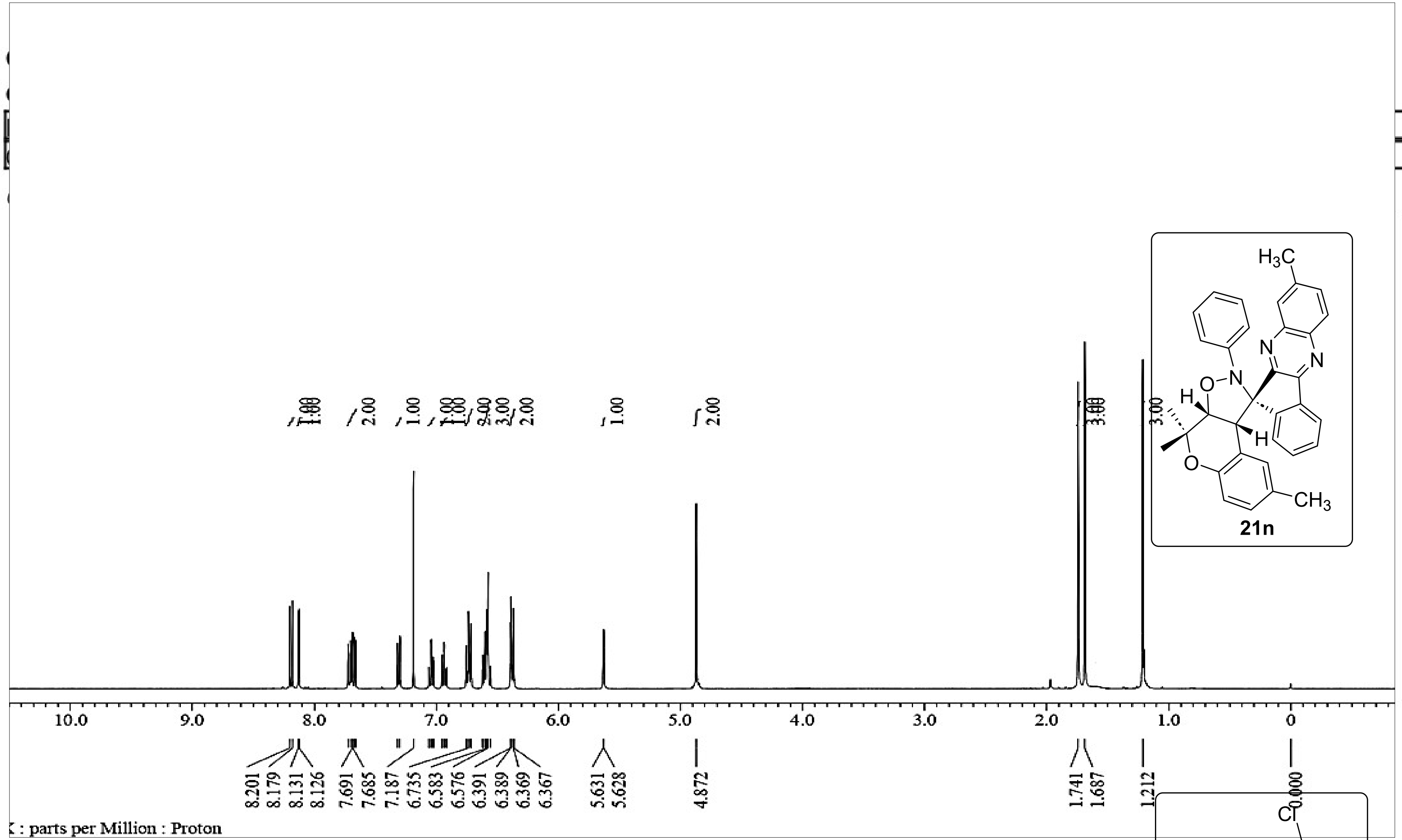

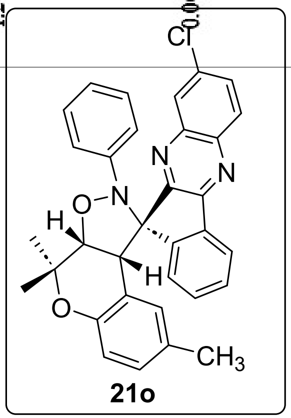

Fig. S64: HRMS of 4,4,8,8'-tetramethyl-2-phenyl-3a,9b-dihydro-2H,4H-spiro[chromeno[4,3-d]isoxazole-1,11'-indeno[1,2-b]quinoxaline] (21n)

Fig. S65: ^{1}H NMR of 8'-chloro-4,4,8-trimethyl-2-phenyl-3a,9b-dihydro-2H,4H-spiro[chromeno[4,3-d]isoxazole-1,11'-indeno[1,2-b]quinoxaline] (21o)

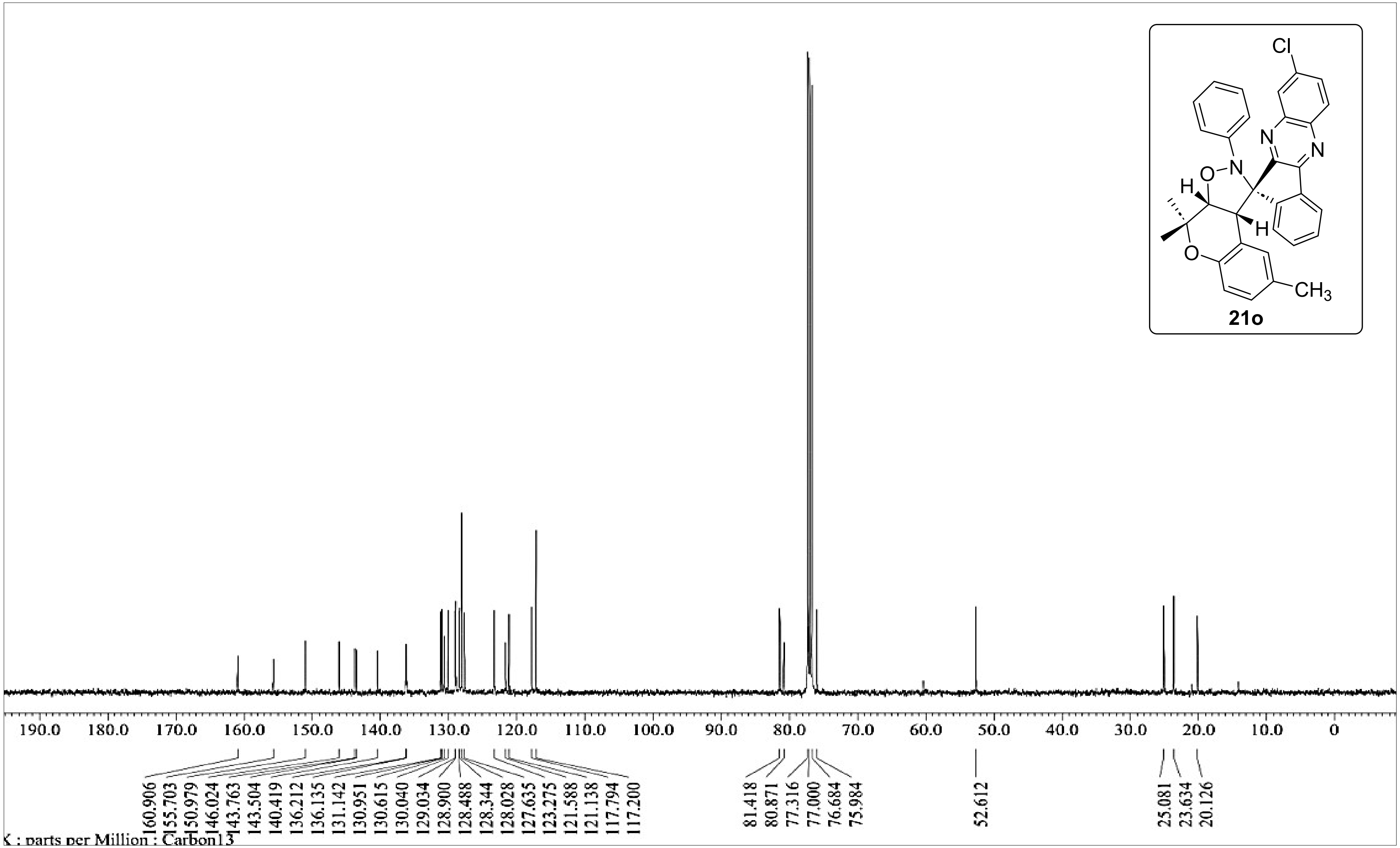

Fig. S66: ^{13}C NMR of 8'-chloro-4,4,8-trimethyl-2-phenyl-3a,9b-dihydro-2H,4H-spiro[chromeno[4,3-d]isoxazole-1,11'-indeno[1,2-b]quinoxaline] (21o)

Compound Details

Cpd. 1: C33 H26 Cl N3 O2

Formula	m/z	Observed M/Z	Difference_Da	Difference_PPM	Score
C33 H26 Cl N3 O2	532.1785	532.178466899423	-0.0178556059609036	-0.0336155287730422	97.98

Compound Spectra (Zoomed)

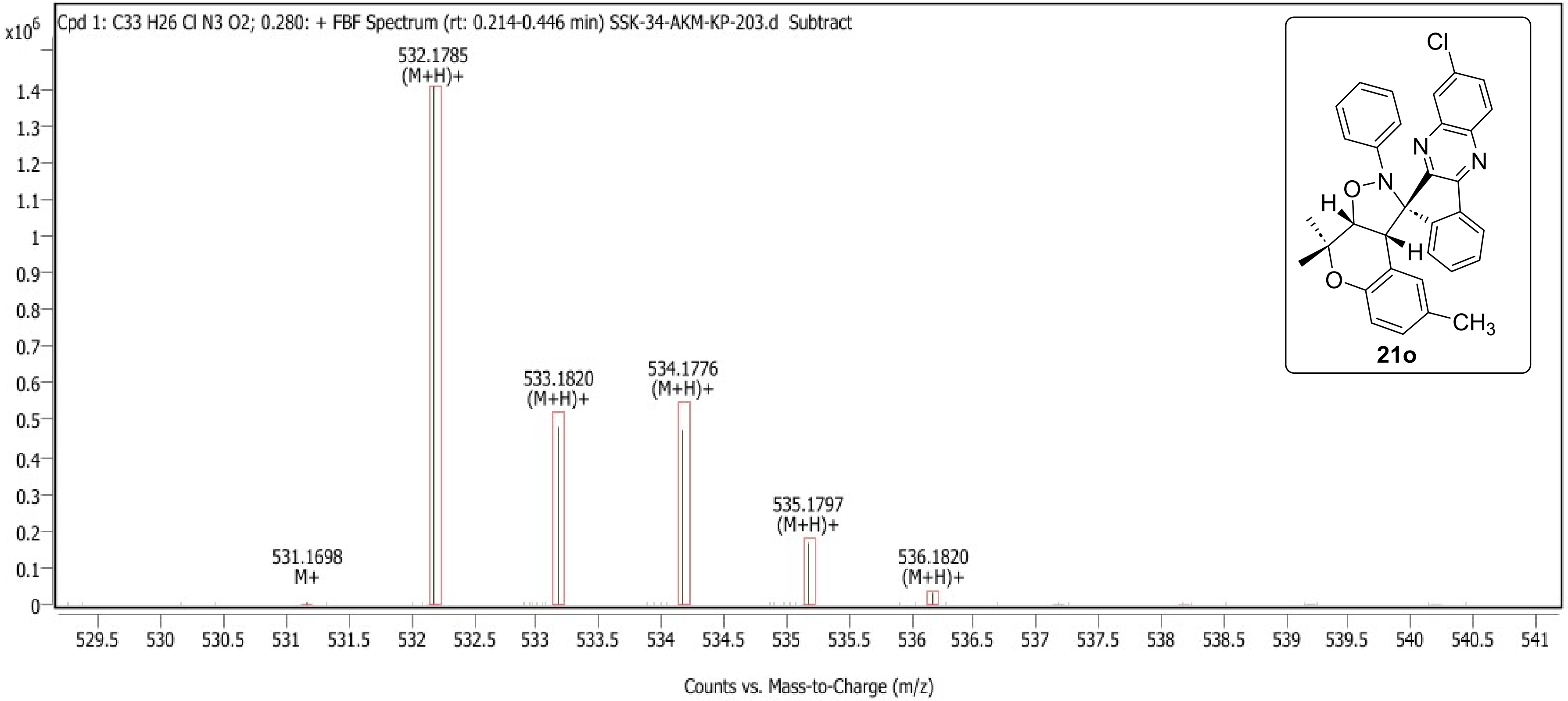

Fig. S67: HRMS of 4,4,8,8'-tetramethyl-2-phenyl-3a,9b-dihydro-2H,4H-spiro[chromeno[4,3-d]isoxazole-1,11'-indeno[1,2-b]quinoxaline] (21o)

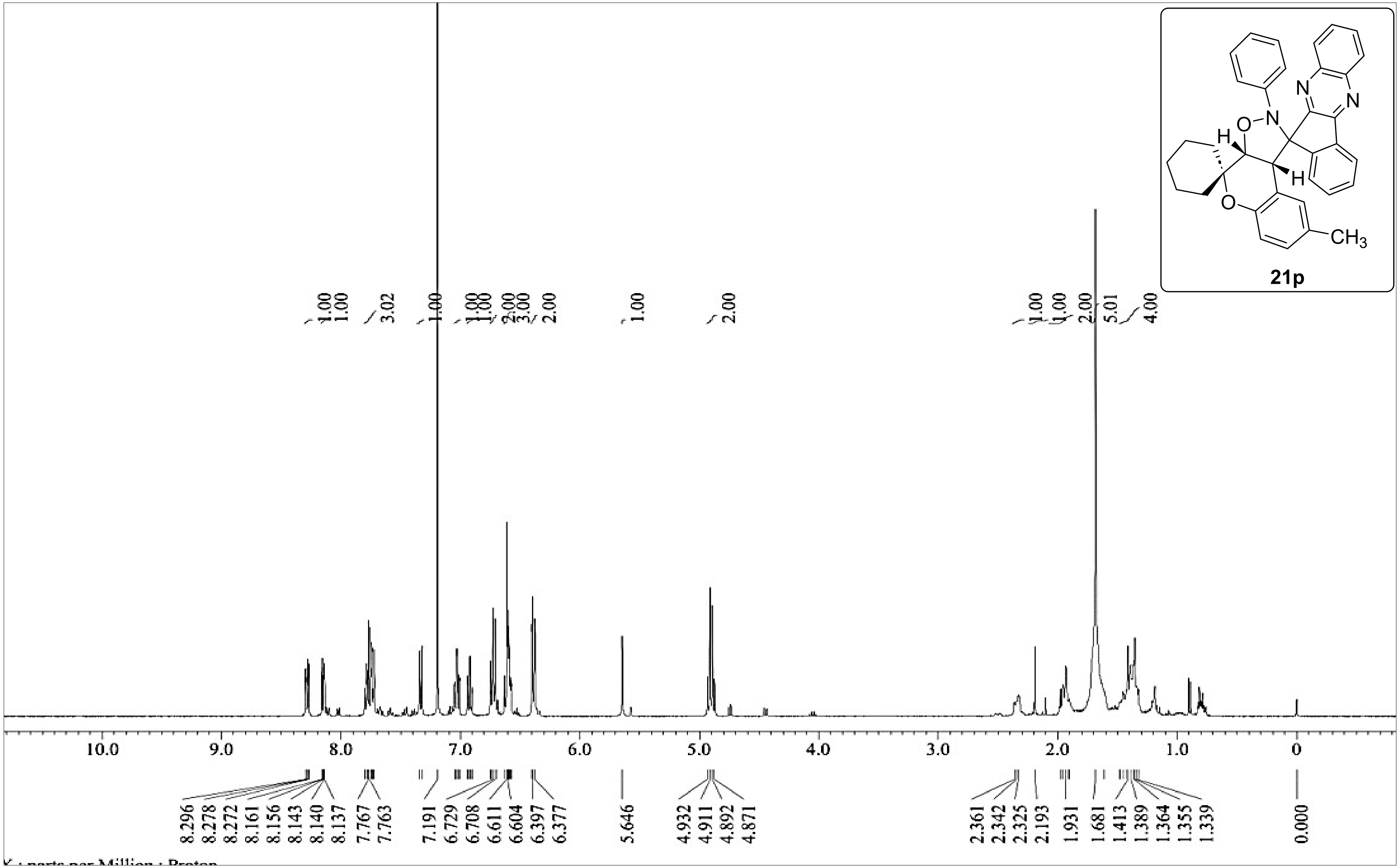

Fig. S68: ^{1}H NMR of of 8'-methyl-2'-phenyl-3a',9b'-dihydro-2'H-dispiro[cyclohexane-1,4'-chromeno[4,3-d]isoxazole-1',11''-indeno[1,2-b]quinoxaline] (21p)

Compound Details

Cpd. 1: C36 H31 N3 O2

Formula	m/z	Observed M/Z	Difference_Da	Difference_PPM	Score
C36 H31 N3 O2	538.2497	538.249655090324	0.571067115060941	1.06296140523962	98.86

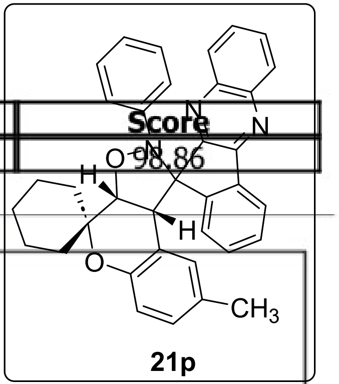

Compound Spectra (Zoomed)

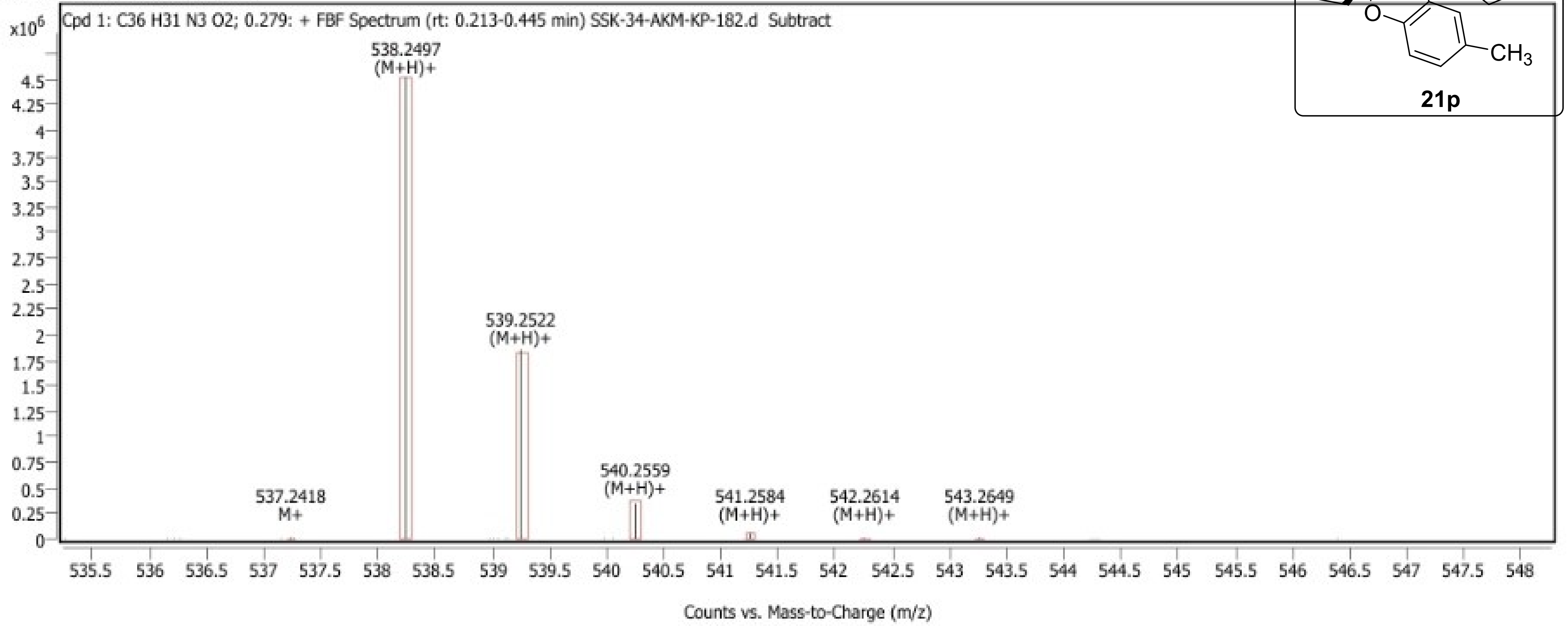

: parts per Million : Carbon13

Fig. S69: ^{13}C NMR of 8'-methyl-2'-phenyl-3a',9b'-dihydro-2'H-dispiro[cyclohexane-1,4'-chromeno[4,3-d]isoxazole-1',11''-indeno[1,2-b]quinoxaline] (21p)

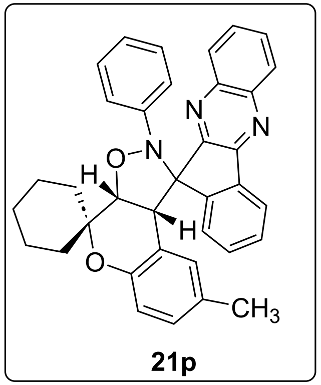

Fig. S70: HRMS of 8'-methyl-2'-phenyl-3a',9b'-dihydro-2'H-dispiro[cyclohexane-1,4'-chromeno[4,3-d]isoxazole-1',11''-indeno[1,2-b]quinoxaline] (21p)

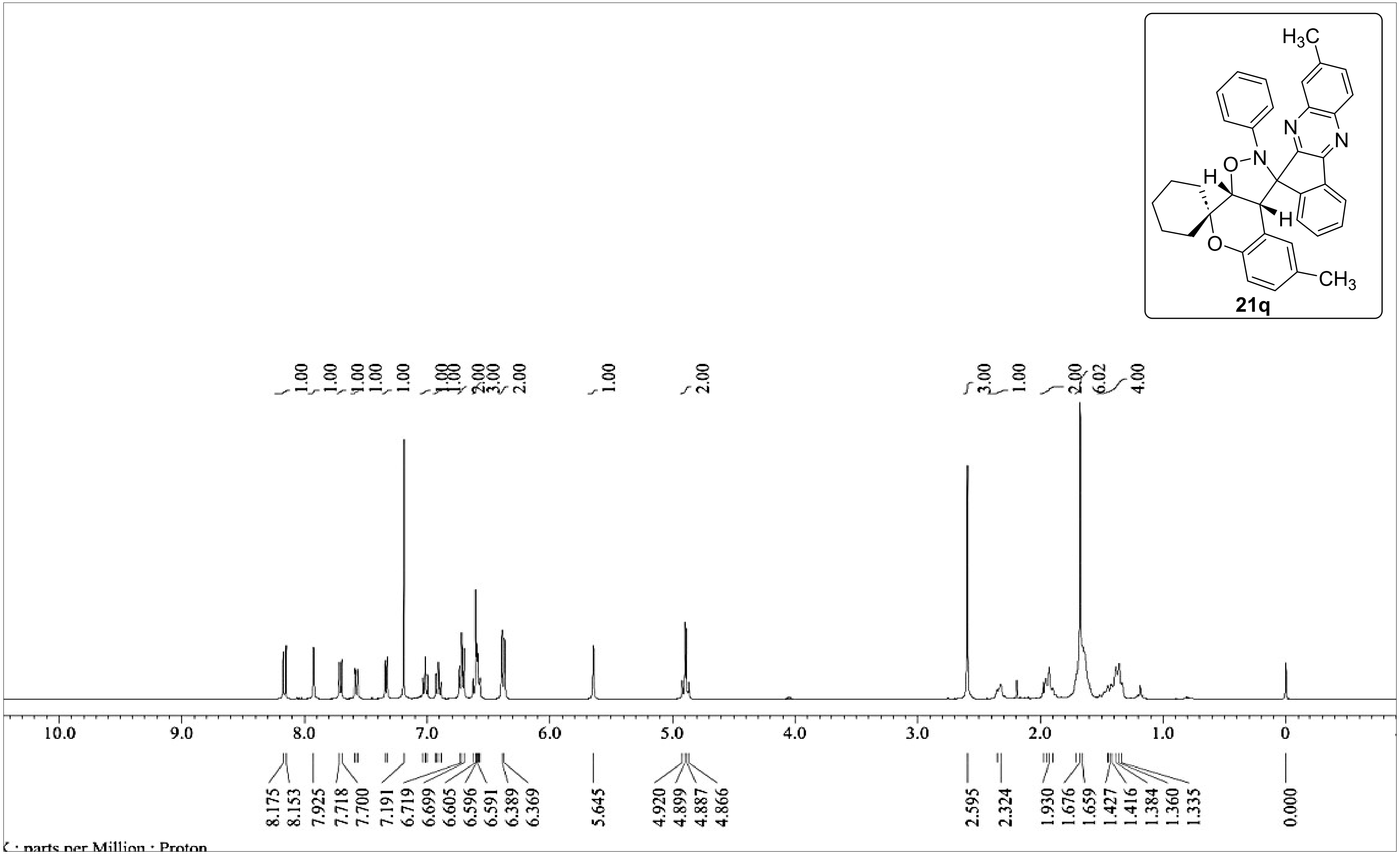

Fig. S71: ^{1}H NMR of 8',8''-dimethyl-2'-phenyl-3a',9b'-dihydro-2'H-dispiro[cyclohexane-1,4'-chromeno[4,3-d]isoxazole-1',11''-indeno[1,2-b]quinoxaline] (21q)

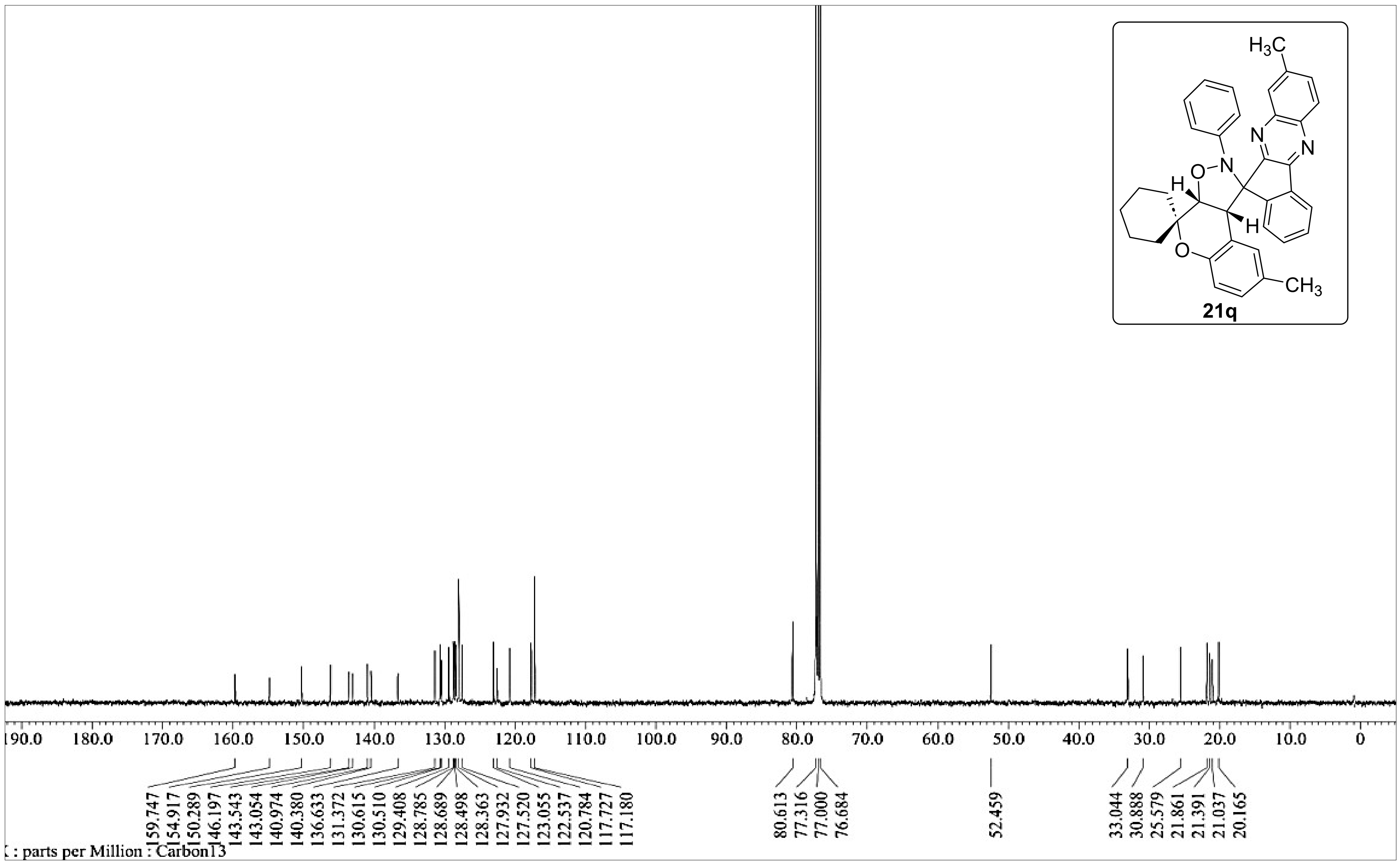

Fig. S72: ^{13}C NMR of 8',8''-dimethyl-2'-phenyl-3a',9b'-dihydro-2'H-dispiro[cyclohexane-1,4'-chromeno[4,3-d]isoxazole-1',11''-indeno[1,2-b]quinoxaline] (21q)

Compound Details

Cpd. 1: C37 H33 N3 O2

Formula	m/z	Observed M/Z	Difference Da	Difference PPM	Score
C37 H33 N3 O2	552.2658	552.265826027224	0.982693893433861	1.78264112577748	97.18

Compound Spectra (Zoomed)

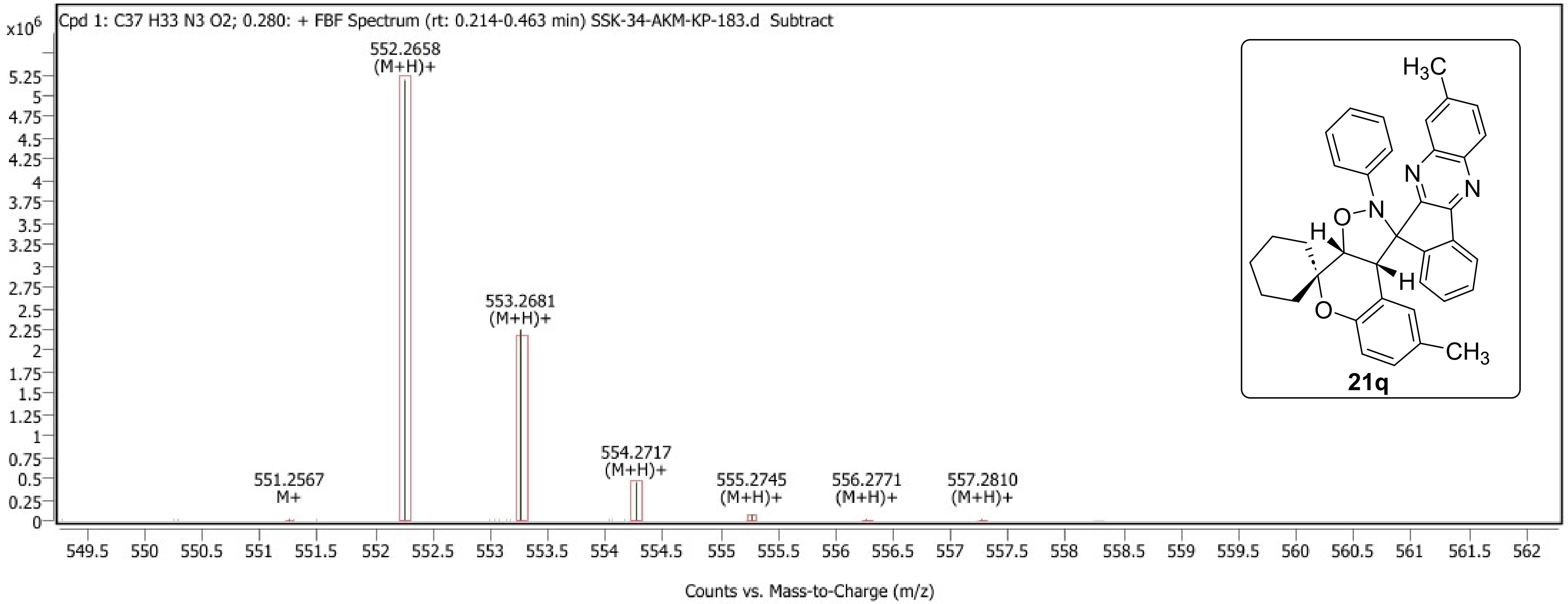

Fig. S73: HRMS of of 8',8''-dimethyl-2'-phenyl-3a',9b'-dihydro-2'H-dispiro[cyclohexane-1,4'-chromeno[4,3-d]isoxazole-1',11''-indeno[1,2-b]quinoxaline] (21q)

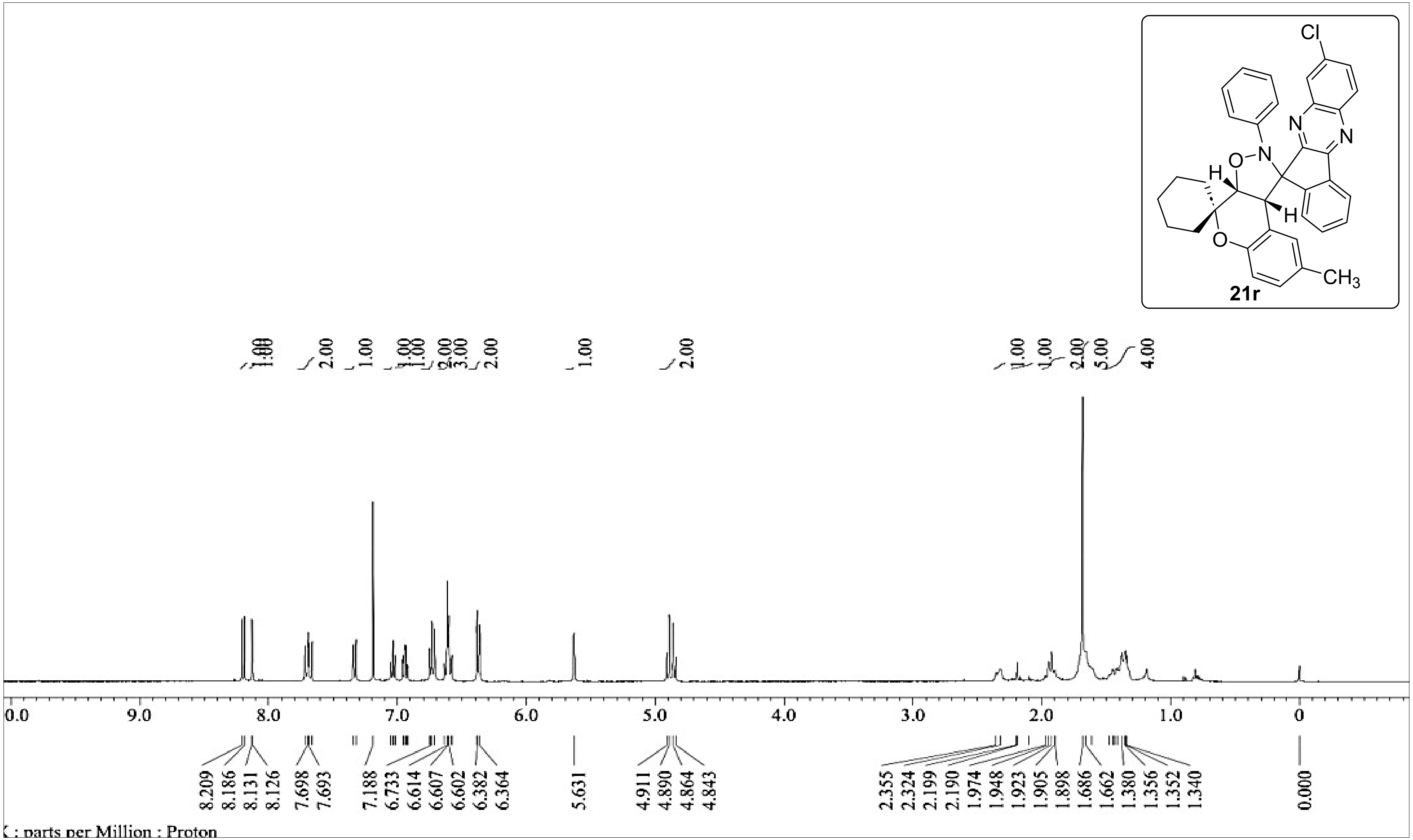

Fig. S74: ^{1}H NMR of 8''-chloro-8'-methyl-2'-phenyl-3a',9b'-dihydro-2'H-dispiro[cyclohexane-1,4'-chromeno[4,3-d]isoxazole-1',11''-indeno[1,2-b]quinoxaline] (21r)

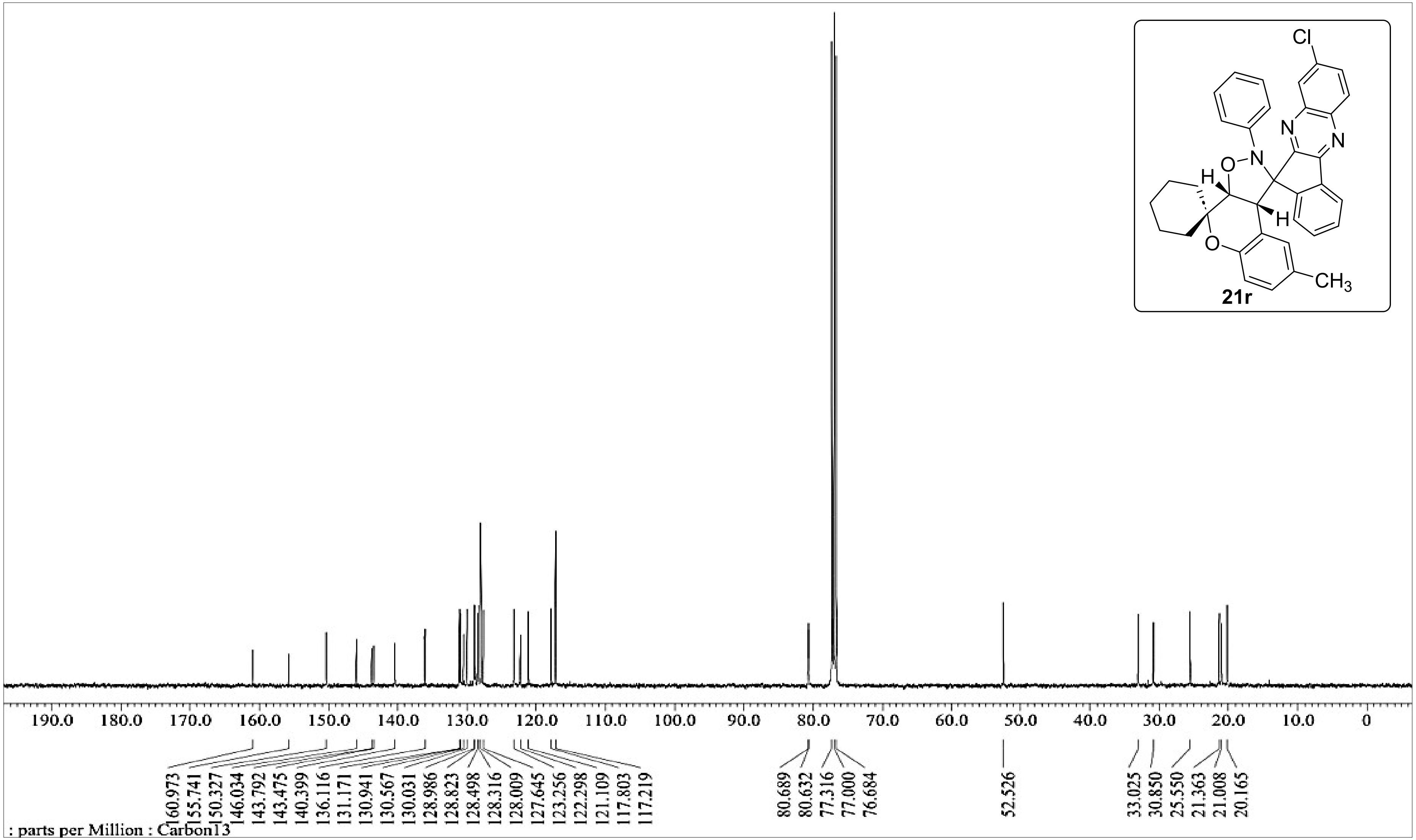

Fig. S75: ^{13}C NMR of 8''-chloro-8'-methyl-2'-phenyl-3a',9b'-dihydro-2'H-dispiro[cyclohexane-1,4'-chromeno[4,3-d]isoxazole-1',11''-indeno[1,2-b]quinoxaline] (21r)

Compound Details

Cpd. 1: C36 H30 Cl N3 O2

Formula	m/z	Observed M/Z	Difference_Da	Difference_PPM	Score
C36 H30 Cl N3 O2	572.2096	572.209621233474	-0.169070966649087	-0.295991212906183	98.82

Compound Spectra (Zoomed)

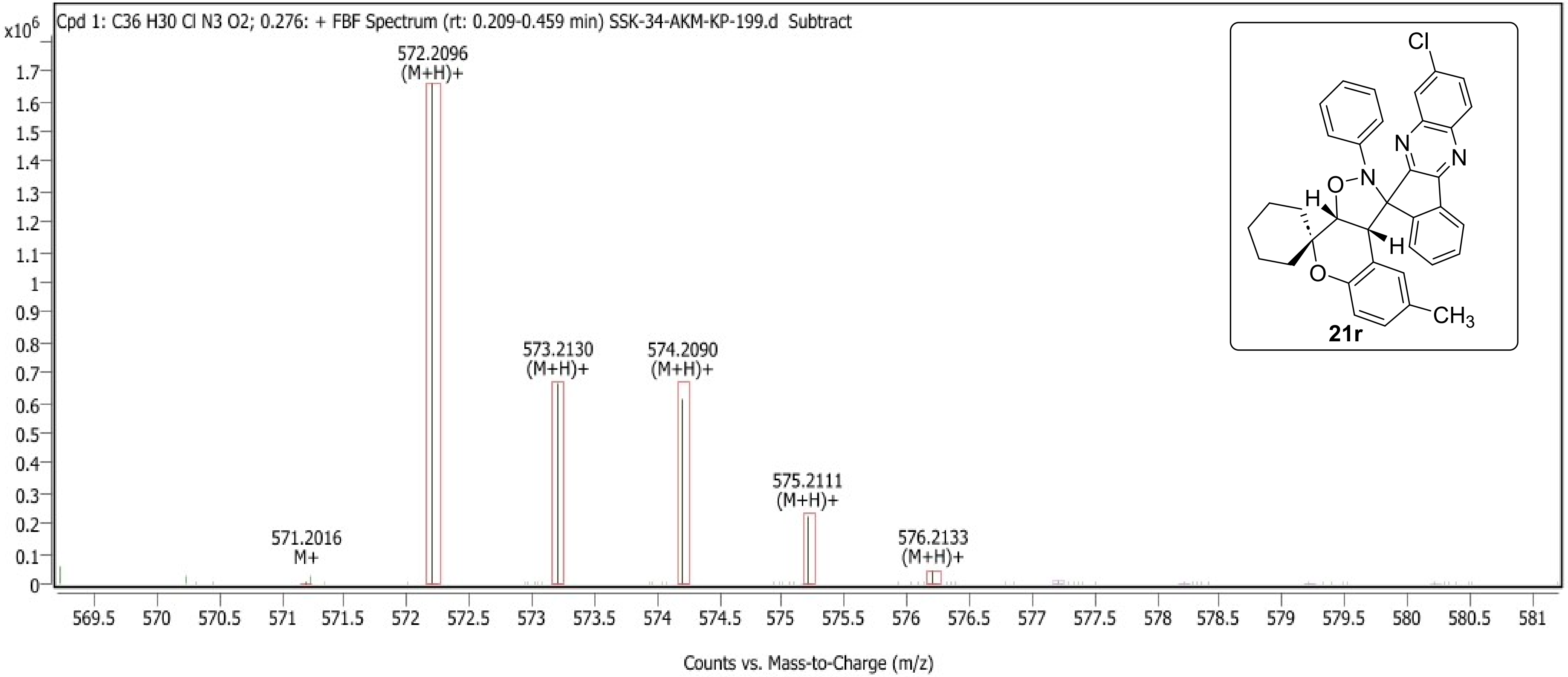

Fig. S76:HRMS of 8''-chloro-8'-methyl-2'-phenyl-3a',9b'-dihydro-2'H-dispiro[cyclohexane-1,4'-chromeno[4,3-d]isoxazole-1',11''-indeno[1,2-b]quinoxaline] (21r)

Crystal Data

Datablock: ska-kp-172_auto

Bond precision:	C-C = 0.0018 A		Wavelength=0.71073
Cell:	a=9.3947(4)	b=13.1348(6)	c=20.8649(11)
	alpha=90	beta=96.586(4)	gamma=90
Temperature :	100 K		

	Calculated	Reported
Volume	2557.7(2)	2557.7(2)
Space group	P 21/n	P 1 21/n 1
Hall group	-P 2yn	-P 2yn
Moiety formula	C34 H27 N3 O2	C34 H27 N3 O2
Sum formula	C34 H27 N3 O2	C34 H27 N3 O2
Mr	509.59	509.58
Dx,g cm-3	1.323	1.323
Z	4	4
Mu (mm-1)	0.083	0.083
F000	1072.0	1072.0
F000'	1072.42	
h,k,lmax	13,18,29	12,18,27
Nref	7915	6126
Tmin,Tmax	0.979,0.982	0.799,1.000
Tmin'	0.979	

Correction method= # Reported T Limits: Tmin=0.799 Tmax=1.000 AbsCorr = MULTI-SCAN

Data completeness= 0.774 Theta(max)= 30.669

R(reflections)= 0.0424(4831) wR2(reflections)= 0.1066(6126)

S = 1.065 Npar= 352

The following ALERTS were generated. Each ALERT has the format
test-name_ALERT_alert-type_alert-level.
Click on the hyperlinks for more details of the test.

```
The following ALERTS were generated. Each ALERT has the format
       test-name_ALERT_alert-type_alert-level.
Click on the hyperlinks for more details of the test.
```

Alert level B

```
PLAT910_ALERT_3_B Missing FCF Reflection(s) Below Theta(Min)[Deg]=       3.33 Note
                1  1  0,    0  2  0,   -1  0  1,    1  0  1,   -1  1  1,    0  1  1,
                1  1  1,    0  2  1,    0  0  2,   -1  1  2,    0  1  2,
```

Alert level C

```
PLAT911_ALERT_3_C Missing FCF Refl Between Thmin & STh/L=    0.600          6 Report
               -1  0  3,    4  0 16,    5  0 19,    5  1 19,    5  1 20,    5  0 21,
```

Alert level G

```
PLAT395_ALERT_2_G Deviating  X-O-Y   Angle From 120 for O1       .      101.9 Degree
PLAT793_ALERT_4_G Model has Chirality at C1          (Centro SpGr)          R Verify
```
And 2 other PLAT793 Alerts

```
PLAT793_ALERT_4_G Model has Chirality at C2          (Centro SpGr)          S Verify
PLAT793_ALERT_4_G Model has Chirality at C3          (Centro SpGr)          S Verify


PLAT912_ALERT_4_G Missing # of FCF Reflections Above STh/L=  0.600       1635 Note
PLAT941_ALERT_3_G Average HKL Measurement Multiplicity ...........        3.9 Low
PLAT952_ALERT_5_G Calculated (ThMax) and CIF-Reported Lmax Differ.          2 Units
PLAT958_ALERT_1_G Calculated (ThMax) and Actual (FCF) Lmax Differ.          2 Units
PLAT969_ALERT_5_G The 'Henn et al.' R-Factor-gap value ..........      2.216 Note
              Predicted wR2: Based on SigI**2  4.81 or SHELX Weight 10.01
PLAT978_ALERT_2_G Number C-C Bonds with Positive Residual Density.         19 Info
```

Datablock ska-kp-172_auto - ellipsoid plot

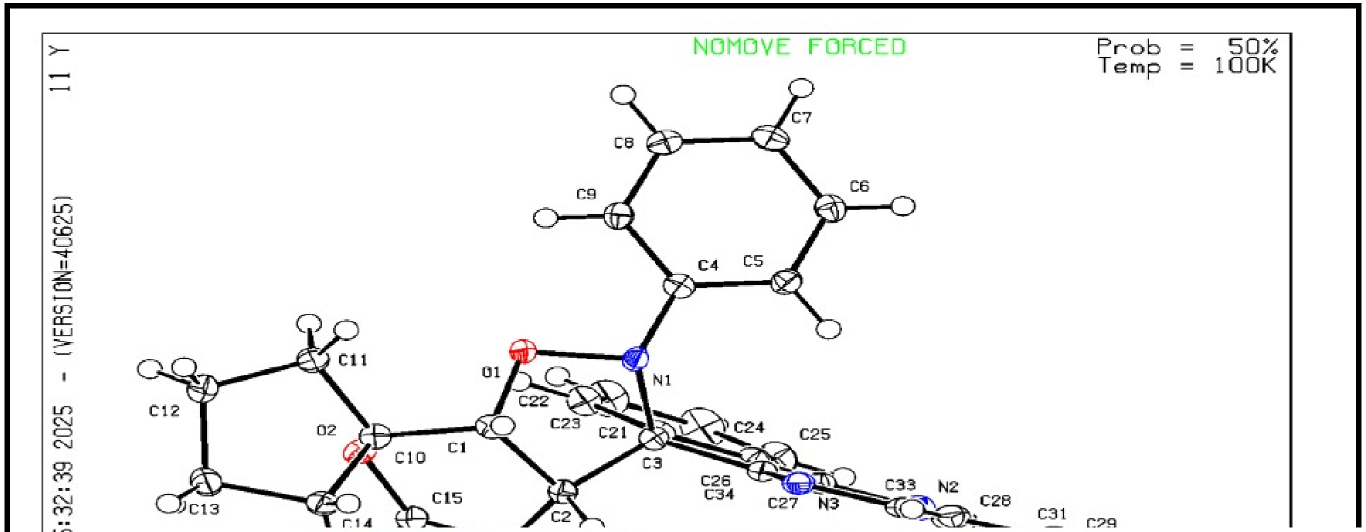

0 ALERT level A = Most likely a serious problem - resolve or explain
1 ALERT level B = A potentially serious problem, consider carefully
1 ALERT level C = Check. Ensure it is not caused by an omission or oversight
10 ALERT level G = General information/check it is not something unexpected

1 ALERT type 1 CIF construction/syntax error, inconsistent or missing data
2 ALERT type 2 Indicator that the structure model may be wrong or deficient
3 ALERT type 3 Indicator that the structure quality may be low
4 ALERT type 4 Improvement, methodology, query or suggestion
2 ALERT type 5 Informative message, check

Fig. S77: X- ray **crystal structure of 21c**

It is advisable to attempt to resolve as many as possible of the alerts in all categories. Often the minor alerts point to easily fixed oversights, errors and omissions in your CIF or refinement strategy, so attention to these fine details can be worthwhile. In order to resolve some of the more serious problems it may be necessary to carry out additional measurements or structure refinements. However, the purpose of your study may justify the reported deviations and the more serious of these should normally be commented upon in the discussion or experimental section of a paper or in the "special_details" fields of the CIF. checkCIF was carefully designed to identify outliers and unusual parameters, but every test has its limitations and alerts that are not important in a particular case may appear. Conversely, the absence of alerts does not guarantee there are no aspects of the results needing attention. It is up to the individual to critically assess their own results and, if necessary, seek expert

Publication of your CIF in other journals

Please refer to the *Notes for Authors* of the relevant journal for any special instructions relating to CIF submission.

PLATON version of 04/06/2025; check.def file version of 30/05/2025

Table S1 Molecular docking analysis of synthesized compounds **21(a-r)** and standard drug Doxorubicin as ligand with EGFR (PDB ID: 4HJO) and MDM2 (PDB ID: 5LAV) target proteins

**Ligands**	**EGFR (PDB ID: 4HJO)**		**MDM2 (PDB ID: 5LAV)**	
	**Docking score (kcal mol^{-1})**	**Molecular interaction type with amino acid residues**	**Docking score (kcal mol^{-1})**	**Molecular interaction type with amino acid residues**
**21a**	-8.21	Hydrogen bond interactions: CYS773 (Conventional-H bond), GLY772 (C-H bond); Hydrophobic interactions: VAL702, MET769, LEU768 (alkyl), LEU694 (π-σ), PHE771, ASP776 (π-alkyl)	-10.89	Hydrophobic interactions: VAL93, ILE61, ILE99 (π-alkyl), TYR67 (π-π T-shaped), PHE91 (π-π stacked), LEU54 (π-σ); Electrostatic interactions: HIS96 (π-cation)
**21b**	-8.56	Hydrogen bond interactions: CYS773 (Conventional-H bond), GLY772 (C-H bond); Hydrophobic interactions: VAL702, MET769, LEU768 (alkyl), LEU694, LEU820 (π-σ), PHE771, TYR777 (π-alkyl)	-10.91	Hydrogen bond interactions: GLY58 (C-H bond); Hydrophobic interactions: VAL75, VAL93, ILE61, ILE99, LEU57, LEU54 (alkyl), PHE91, PHE86, TYR67 (π-alkyl), LEU54 (π-σ); Electrostatic interactions: HIS96 (π-cation)
**21c**	-7.67	Hydrogen bond interactions: GLY772 (C-H bond); Hydrophobic interactions: VAL702, MET769, LEU768, ALA719 (alkyl), LEU694, LEU820 (π-σ), PHE771, TYR777 (π-alkyl), CYS773 (π-sulfur)	-10.48	Hydrogen bond interactions: GLY58 (C-H bond); Hydrophobic interactions: VAL93, LEU57, ILE99 (alkyl), ILE61, PHE91 (π-alkyl), TYR67 (π-π T-shaped), MET62 (π-sulfur); Electrostatic interactions: HIS96 (π-cation)
**21d**	-7.64	Hydrogen bond interactions: CYS773 (Conventional-H bond), GLY772 (C-H bond); Hydrophobic interactions: LEU694, LEU820 (π-σ), PHE771, TYR777 (π-alkyl), VAL702, MET769, LEU768, ALA719 (alkyl)	-10.41	Hydrogen bond interactions: GLY58 (C-H bond); Hydrophobic interactions: VAL93, ILE61, LEU54, LEU57, ILE99, ILE103 (alkyl), PHE55 (π-π stacked), PHE86, PHE91 (π-alkyl); Electrostatic interactions: HIS96 (π-cation)
**21e**	-8.06	Hydrogen bond interactions:	-10.54	Hydrogen bond interactions: GLN24 (Conventional-H bond); Hydrophobic interactions: MET62,

		PRO770 (C-H bond); Hydrophobic interactions: LEU694, PHE771, TYR777, HIS781 (π-alkyl)		LEU57, ILE61, ILE99 (alkyl), PHE86, PHE91 (π-alkyl), TYR86 (π-π T-shaped), LEU54 (π-σ); Electrostatic interactions: HIS96 (π-cation)
**21f**	-8.85	Hydrogen bond interactions: CYS773 (Conventional-H bond), GLY772 (C-H bond); Hydrophobic interactions: VAL702, MET769, LEU768, LEU820 (Alkyl), LEU694 (π-σ), PHE771, TYR777 (π-alkyl)	-11.23	Hydrogen bond interactions: GLN24 (Conventional-H bond); Hydrophobic interactions: MET62 (π-sulfur), ILE61, ILE99, LEU57, LEU54 (alkyl), PHE86, PHE91 (π-alkyl), LEU54 (Pi-Sigma); Electrostatic interactions: HIS96 (π-cation)
**21g**	-7.47	Hydrogen bond interactions: GLY772 (C-H bond); Hydrophobic interactions: VAL702, MET769, LEU768, ALA719 (alkyl), LEU694, LEU820 (π-σ), PHE771, TYR777 (π-alkyl), CYS773 (π-sulfur)	-10.33	Hydrogen bond interactions: GLY58 (C-H bond); Hydrophobic interactions: MET62, ILE61, ILE99, VAL93 (π-alkyl), TYR67 (π-π T-shaped), HIS96 LEU54 (amide-π stacked); Electrostatic interactions: (π-cation)
**21h**	-7.54	Hydrogen bond interactions: GLY772 (C-H bond); Hydrophobic interactions: VAL702, MET769, LEU768, ALA719 (alkyl), LEU694, LEU820 (π-σ), PHE771, TYR777, CYS773 (π-alkyl)	-10.26	Hydrogen bond interactions: GLN24 (Conventional-H bond); Hydrophobic interactions: LEU57, MET62, ILE61, ILE99 (alkyl), PHE86, PHE91 (π-alkyl), TYR86 (π-π T-shaped), LEU54 (π-σ); Electrostatic interactions: HIS96 (π-cation)
**21i**	-8.86	Hydrogen bond interactions: GLY772 (C-H bond); Hydrophobic interactions: CYS773, TYR777 (π-alkyl), LEU694 (π-σ)	-11.11	Hydrogen bond interactions: GLN24 (Conventional-H bond); Hydrophobic interactions: MET62, VAL93, LEU54 (alkyl), ILE61, ILE99, ILE103, PHE86, PHE91, LEU57 (π-alkyl), LEU54 (π-π T-shaped), PHE55 (π-π stacked); Electrostatic interactions: HIS96 (π-cation)
**21j**	-10.24	Hydrogen bond interactions: CYS773 (Conventional-H bond), GLY772 (C-H bond); Hydrophobic interactions: VAL702, MET769, LEU768, LEU820 (alkyl), LEU694 (π-σ), PHE771, TYR777 (π-alkyl); Electrostatic interactions: LYS692 (π-cation)	-12.05	Hydrogen bond interactions: GLN24 (Conventional-H bond); Hydrophobic interactions: VAL75, VAL93, ILE61, ILE99, LEU57, LEU54 (alkyl), PHE86, PHE91, TYR67 (π-alkyl), LEU54 (π-σ); Electrostatic interactions: (HIS96) π-cation

**21k**	-7.85	Hydrogen bond interactions: GLY772 (C-H bond); Hydrophobic interactions: VAL702, MET769, LEU768, ALA719 (alkyl), LEU694, LEU820 (π-σ), PHE771, TYR777 (π-alkyl), CYS773 (π-sulfur)	-10.57	Hydrogen bond interactions: GLY58 (C-H bond); Hydrophobic interactions: MET62, ILE61, ILE99, LEU54 (alkyl), VAL93, PHE91 (π-alkyl), TYR67 (π-π T-shaped), PHE55 (amide-π stacked); Electrostatic interactions: HIS96 (π-cation),
**21l**	-9.26	Hydrogen bond interactions: GLY772 (C-H bond); Hydrophobic interactions: VAL702, MET769, LEU768, ALA719 (alkyl), LEU694, LEU820 (π-σ), PHE771, TYR777 (π-alkyl), CYS773 (alkyl)	-11.62	Hydrogen bond interactions: GLN24 (Conventional-H bond); Hydrophobic interactions: MET62, LEU57, ILE61, ILE99 (alkyl), PHE86, PHE91 (π-alkyl), TYR86 (π-π T-shaped); Electrostatic interactions: HIS96 (π-cation)
**21m**	-8.25	Hydrogen bond interactions: GLY772 (C-H bond); Hydrophobic interactions: PHE771, TYR777 (π-alkyl); Electrostatic interactions: GLU780 (π-anion)	-10.76	Hydrogen bond interactions: GLY58 (C-H bond); Hydrophobic interactions: VAL93, ILE61, ILE99, LEU57, LEU54 (alkyl), PHE91, PHE86, ILE103 (π-alkyl), LEU54 (π-σ); Electrostatic interactions: HIS96 (π-cation)
**21n**	-9.17	Hydrogen bond interactions: GLY772 (C-H bond); Hydrophobic interactions: PHE771, TYR777, HIS781 (π-slkyl)	-11.56	Hydrogen bond interactions: GLY58 (C-H bond); Hydrophobic interactions: VAL93, VAL75, ILE61, ILE99, LEU57, LEU54 (alkyl), PHE91, PHE86, TYR67 (π-alkyl), LEU54 (π-σ), PHE55 (π-π stacked); Electrostatic interactions: HIS96 (π-cation)
**21o**	-9.62	Hydrophobic interactions: LEU820 (Alkyl), LEU694 (π-σ), PHE771 (Amide-π stacked), TYR777 (π-alkyl); Electrostatic interactions: LYS692 (π-cation)	-11.68	Hydrogen bond interactions: GLN24 (Conventional-H bond); Hydrophobic interactions: VAL93, ILE61, ILE99, LEU57 (Alkyl), PHE91, PHE86, ILE103 (π-alkyl)
**21p**	-8.33	Hydrogen bond interactions: CYS773 (Conventional-H bond), GLY772 (C-H bond); Hydrophobic interactions: LEU820, ALA719, MET769, VAL702, LEU768 (alkyl), LEU694 (π-σ), PHE771, ASP776 (π-	-10.87	Hydrogen bond interactions: GLY58 (C-H bond); Hydrophobic interactions: MET62, LEU54, LEU57, ILE61, ILE99, VAL93 (π-alkyl), TYR67 (π-π T-shaped)

		alkyl)		
**21q**	-8.52	Hydrogen bond interactions: GLY772 (C-H bond); Hydrophobic interactions: VAL702, MET769, LEU768, ALA719 (alkyl), PHE771, TYR777, HIS781 (π-alkyl)	-11.09	Hydrogen bond interactions: GLN24 (Conventional-H bond); Hydrophobic interactions: MET62, LEU57, ILE61, ILE99, PHE86, PHE91 (π-alkyl), VAL93 (alkyl)
**21r**	-10.11	Hydrogen bond interactions: GLY772 (C-H bond); Hydrophobic interactions: VAL702, MET769, LEU768, LEU820 (alkyl), PHE771, TYR777, HIS781 (π-alkyl)	-12.03	Hydrogen bond interactions: GLN24 (Conventional-H bond); Hydrophobic interactions: VAL75, VAL93, ILE61, ILE99, LEU57, LEU54 (alkyl), PHE86, PHE91, TYR67 (π-alkyl), LEU54 (π-σ); Electrostatic interactions: HIS96 (π-cation)
**Doxorubicin**	-8.49	Hydrogen bond interactions: LYS721, ASN 818, CYS773 (Conventional-H bond), GLY697 (C-H bond); Hydrophobic interactions: VAL702, LEU820 (π-σ), ALA719 (π-alkyl)	-8.87	Hydrogen bond interactions: HIS 96 (Conventional-H bond); Hydrophobic interactions: LEU57, LEU54 (alkyl), ILE99, GLN24 (π-alkyl), PHE55 (π-π T-shaped)

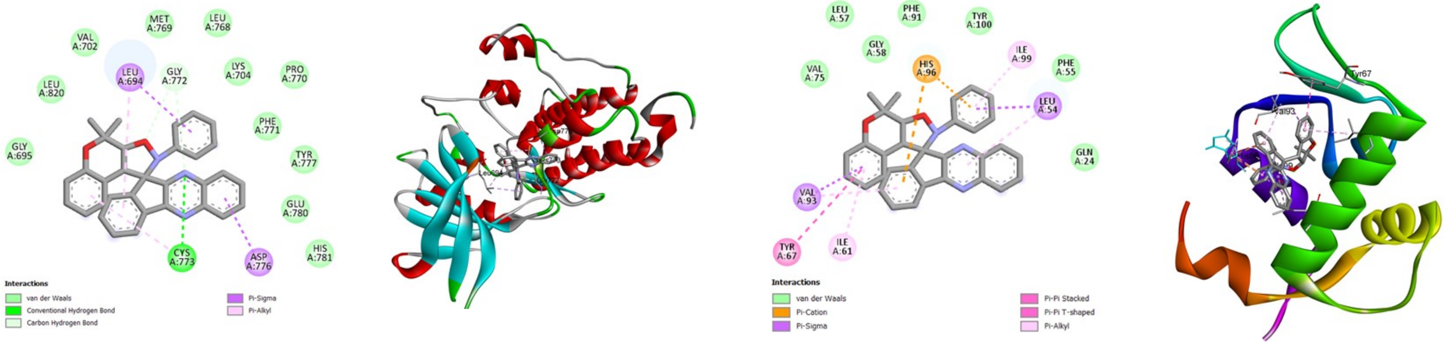

A **B**

Fig. S78: 2D and 3D docking illustrations of compound **21a** in the active sites EGFR protein **[A]** (PDB ID: 4HJO) and MDM2 protein **[B]** (PDB ID: 5LAV)

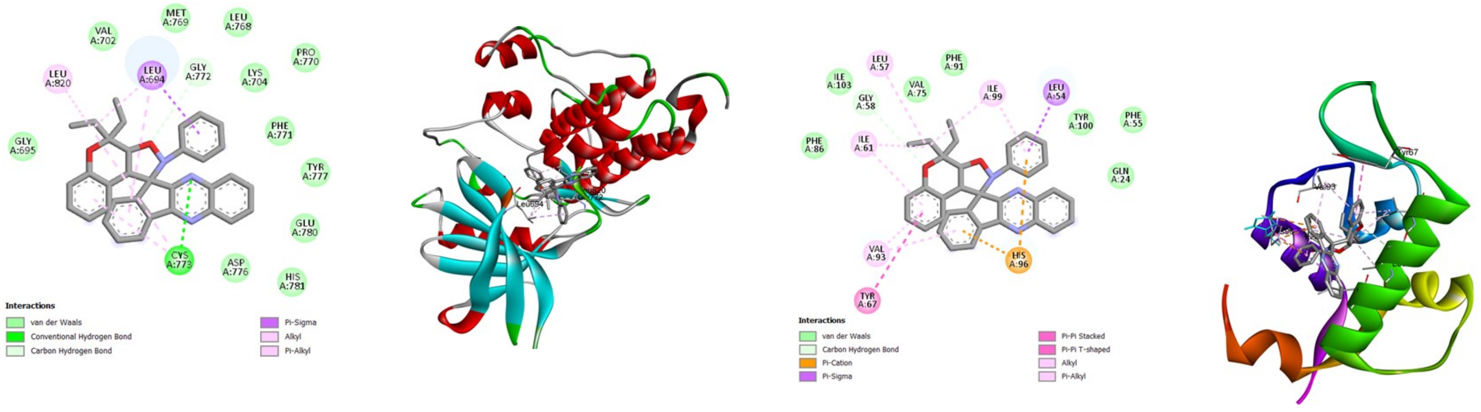

A B

Fig. S79: 2D and 3D docking illustrations of compound **21b** in the active sites EGFR protein **[A]** (PDB ID: 4HJO) and MDM2 protein **[B]** (PDB ID: 5LAV)

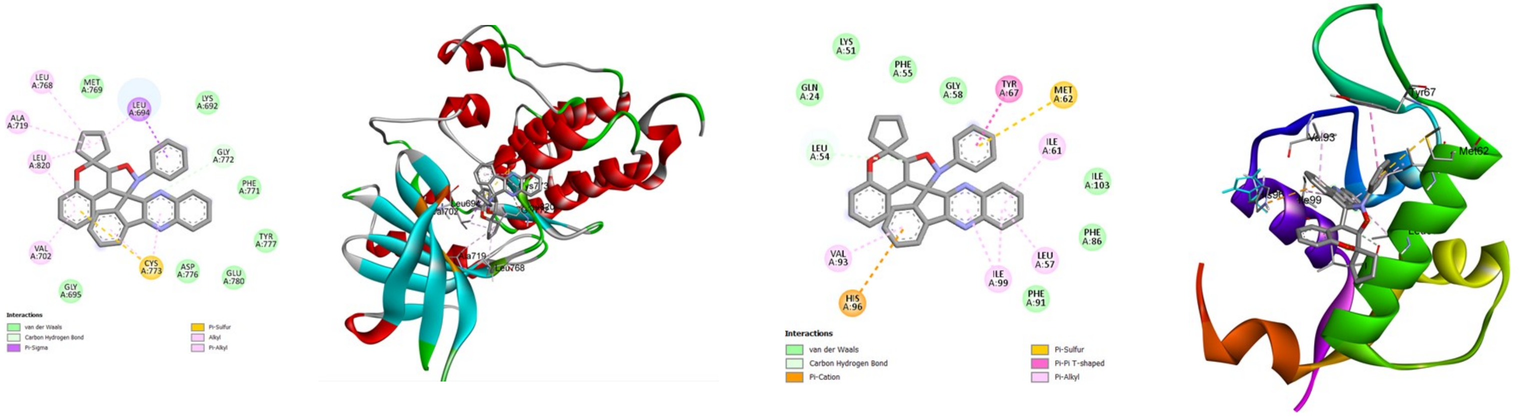

A B

Fig. S80: 2D and 3D docking illustrations of compound **21c** in the active sites EGFR protein **[A]** (PDB ID: 4HJO) and MDM2 protein **[B]** (PDB ID: 5LAV)

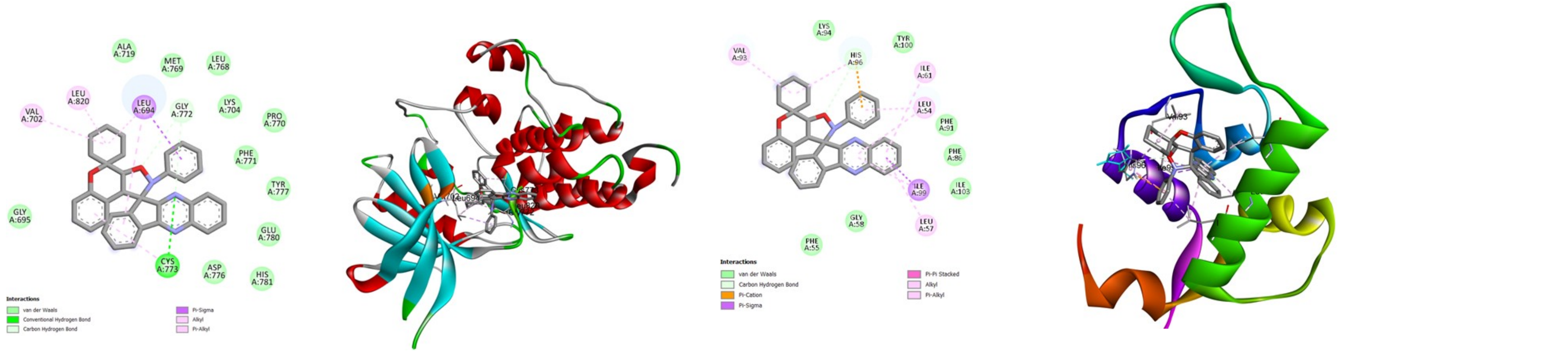

A **B**

Fig. S81: 2D and 3D docking illustrations of compound **21d** in the active sites EGFR protein **[A]** (PDB ID: 4HJO) and MDM2 protein **[B]** (PDB ID: 5LAV)

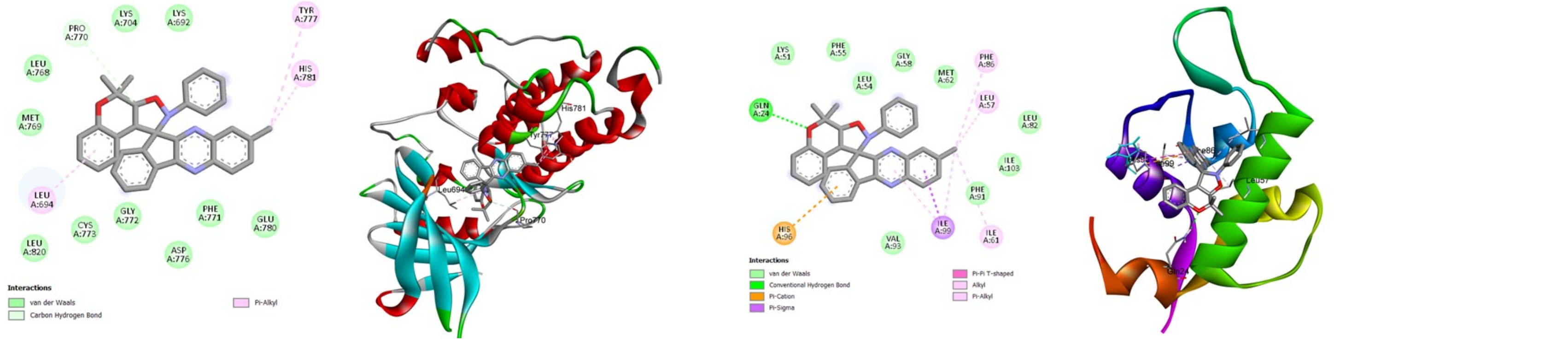

A **B**

Fig. S82: 2D and 3D docking illustrations of compound **21e** in the active sites EGFR protein **[A]** (PDB ID: 4HJO) and MDM2 protein **[B]** (PDB ID: 5LAV)

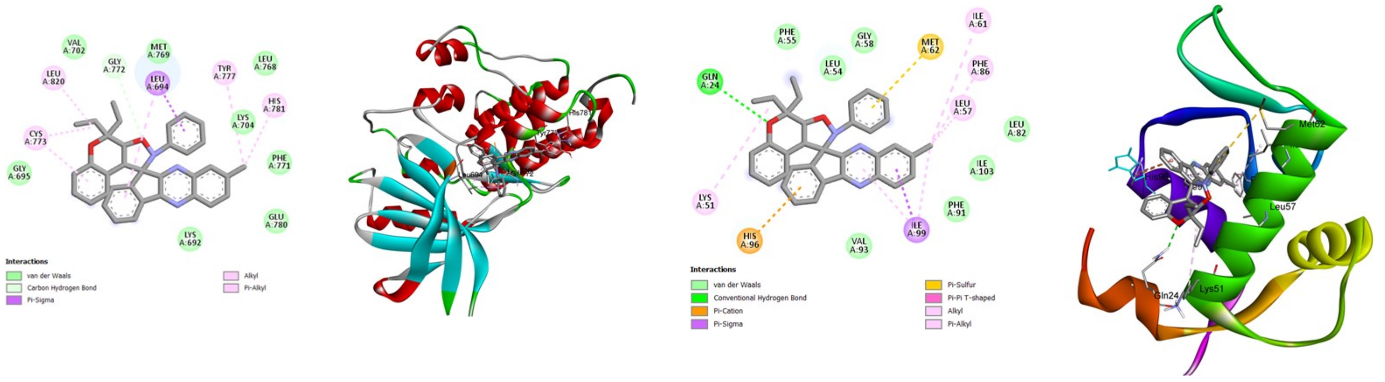

A **B**

Fig. S83: 2D and 3D docking illustrations of compound **21f** in the active sites EGFR protein **[A]** (PDB ID: 4HJO) and MDM2 protein **[B]** (PDB ID: 5LAV)

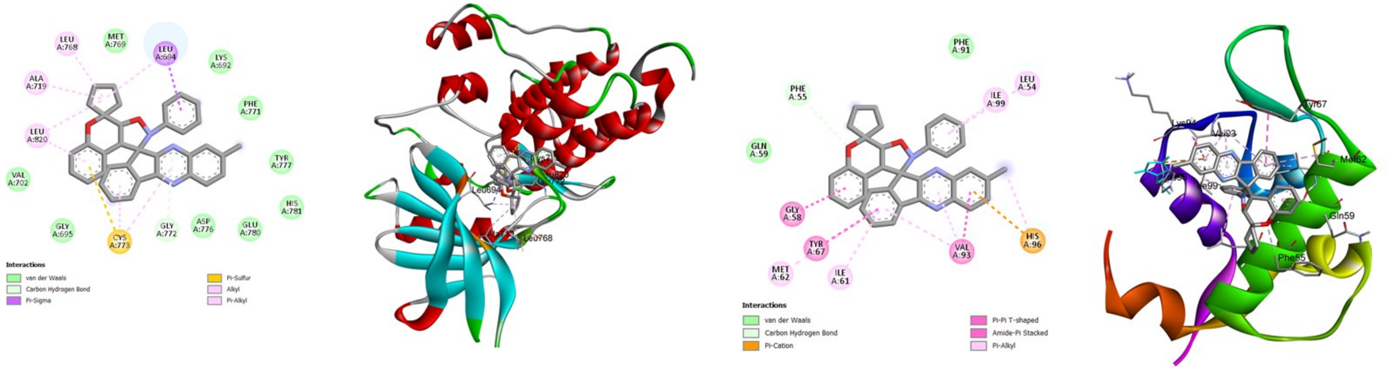

A **B**

Fig. S84: 2D and 3D docking illustrations of compound **21g** in the active sites EGFR protein **[A]** (PDB ID: 4HJO) and MDM2 protein **[B]** (PDB ID: 5LAV)

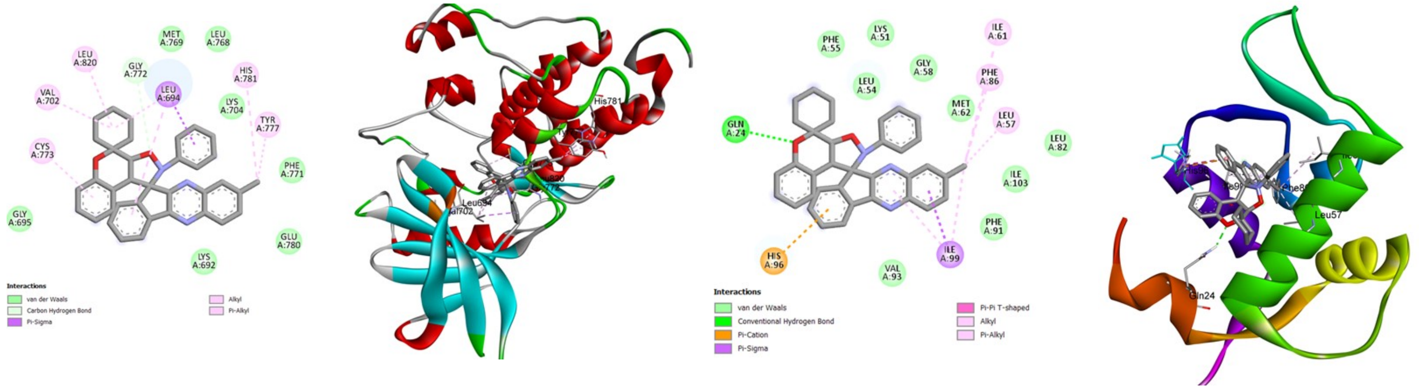

A B

Fig. S85: 2D and 3D docking illustrations of compound **21h** in the active sites EGFR protein **[A]** (PDB ID: 4HJO) and MDM2 protein **[B]** (PDB ID: 5LAV)

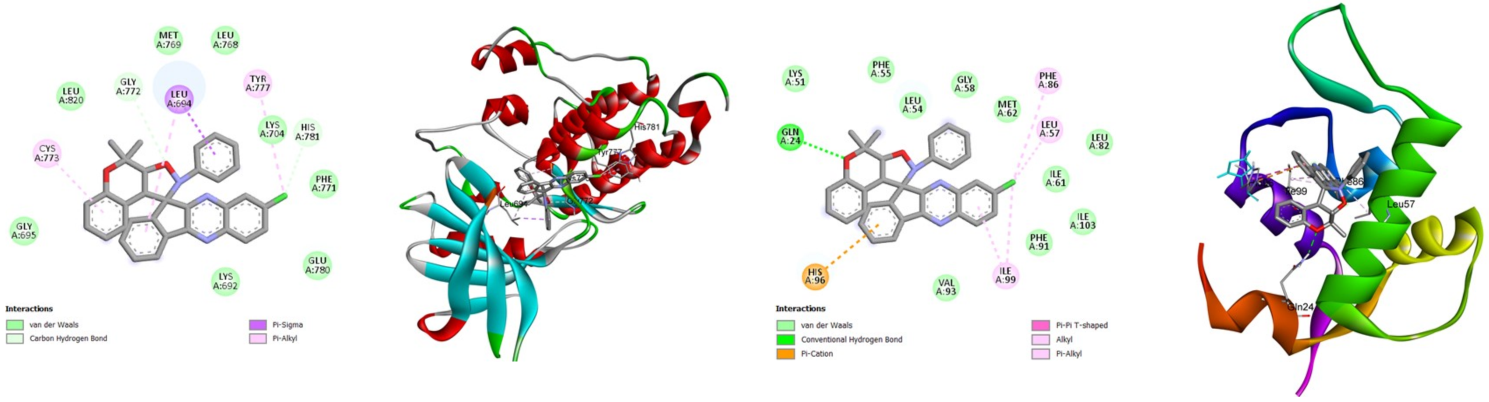

A B

Fig. S86: 2D and 3D docking illustrations of compound **21i** in the active sites EGFR protein **[A]** (PDB ID: 4HJO) and MDM2 protein **[B]** (PDB ID: 5LAV)

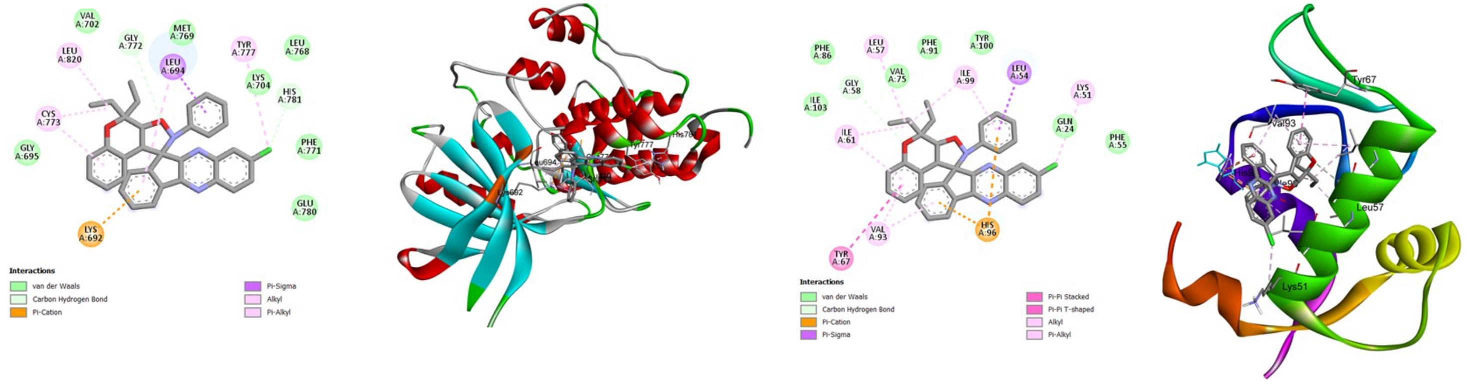

A **B**

Fig. S87: 2D and 3D docking illustrations of compound **21j** in the active sites EGFR protein **[A]** (PDB ID: 4HJO) and MDM2 protein **[B]** (PDB ID: 5LAV)

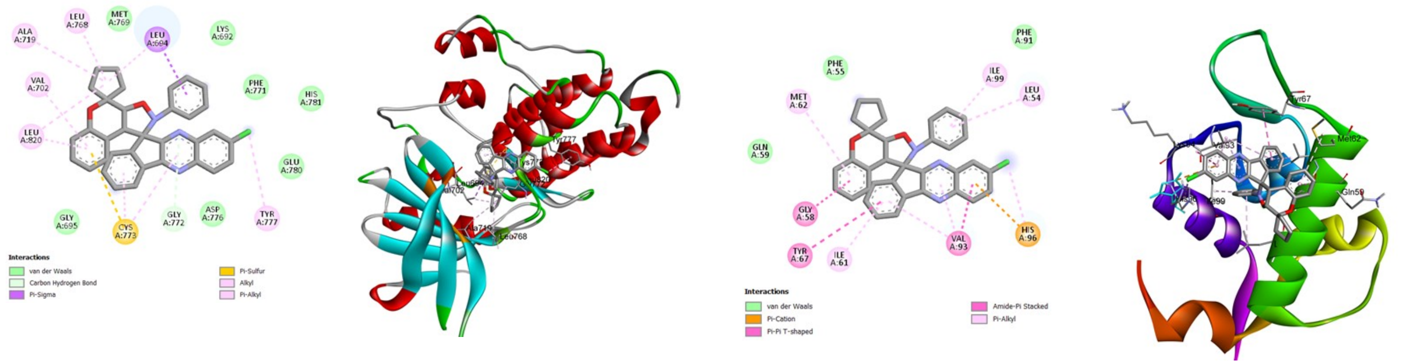

A **B**

Fig. S88: 2D and 3D docking illustrations of compound **21k** in the active sites EGFR protein **[A]** (PDB ID: 4HJO) and MDM2 protein **[B]** (PDB ID: 5LAV)

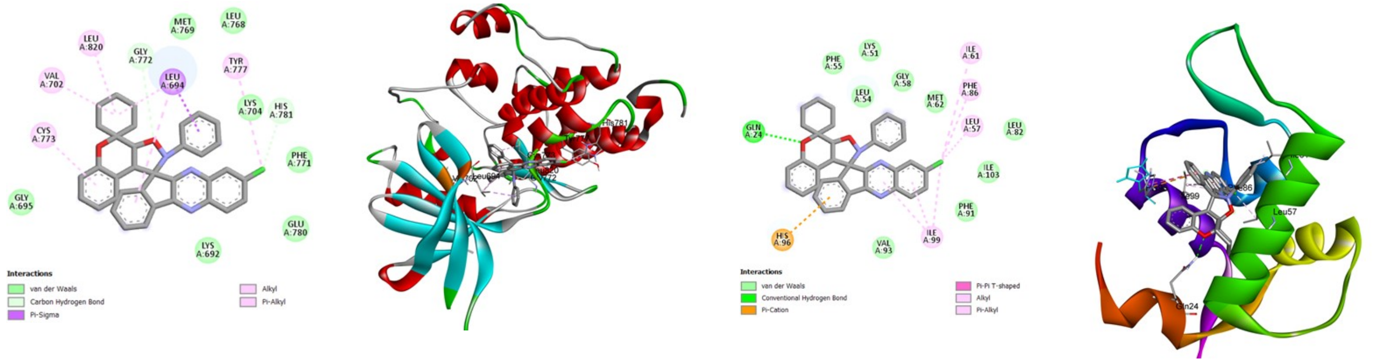

A **B**

Fig. S89: 2D and 3D docking illustrations of compound **21l** in the active sites EGFR protein **[A]** (PDB ID: 4HJO) and MDM2 protein **[B]** (PDB ID: 5LAV)

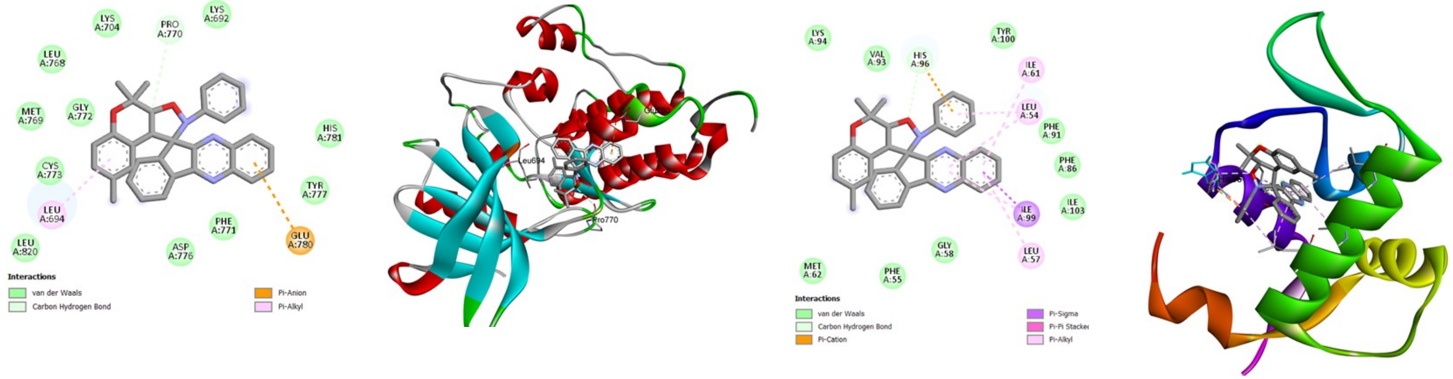

A **B**

Fig. S90: 2D and 3D docking illustrations of compound **21m** in the active sites EGFR protein **[A]** (PDB ID: 4HJO) and MDM2 protein **[B]** (PDB ID: 5LAV))

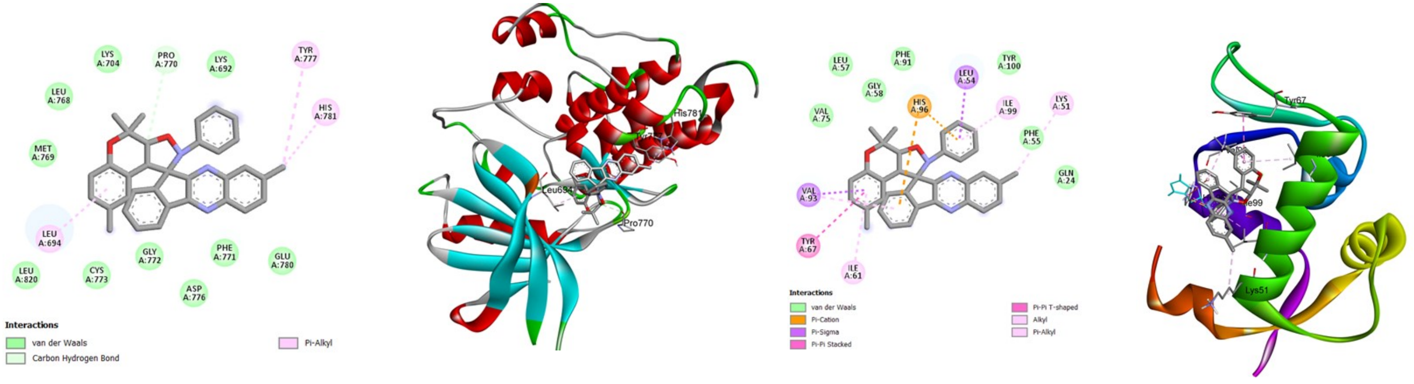

A **B**

Fig. S91: 2D and 3D docking illustrations of compound **21n** in the active sites EGFR protein **[A]** (PDB ID: 4HJO) and MDM2 protein **[B]** (PDB ID: 5LAV)

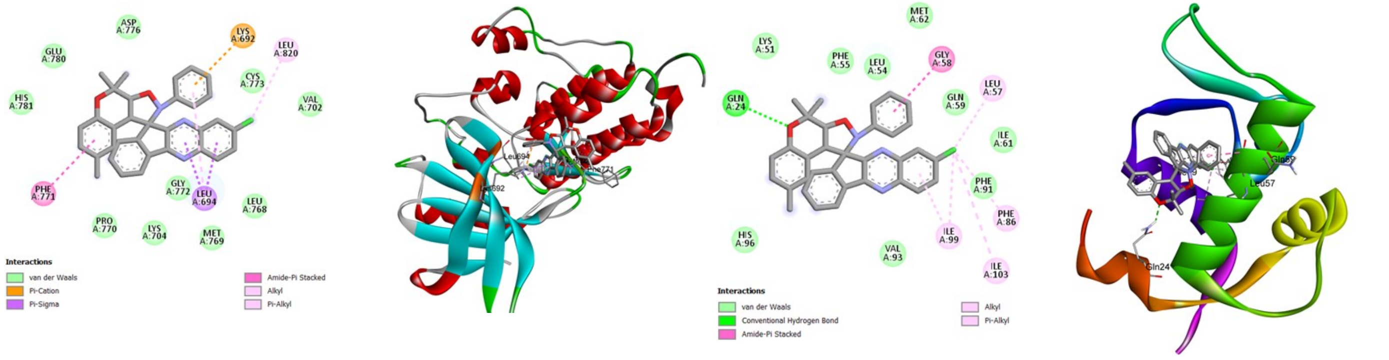

A **B**

Fig. S92: 2D and 3D docking illustrations of compound **21o** in the active sites EGFR protein **[A]** (PDB ID: 4HJO) and MDM2 protein **[B]** (PDB ID: 5LAV)

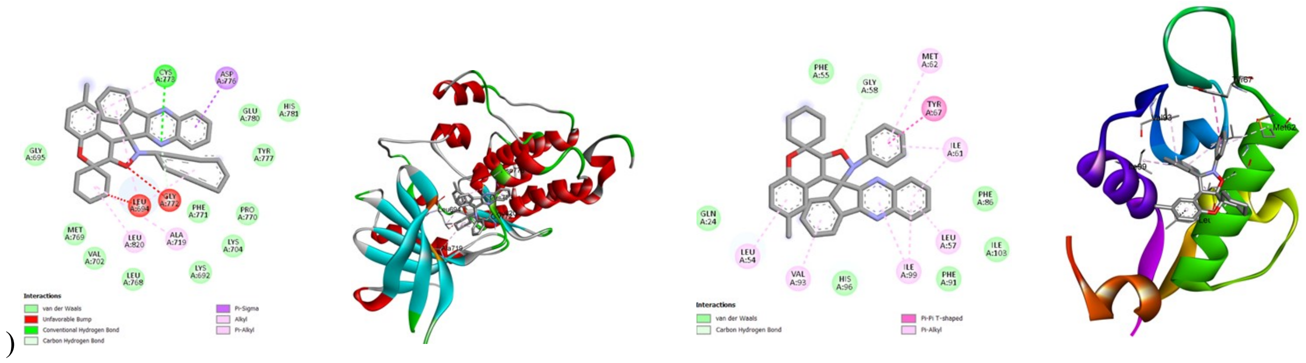

A B

Fig. S93: 2D and 3D docking illustrations of compound **21p** in the active sites EGFR protein **[A]** (PDB ID: 4HJO) and MDM2 protein **[B]** (PDB ID: 5LAV)

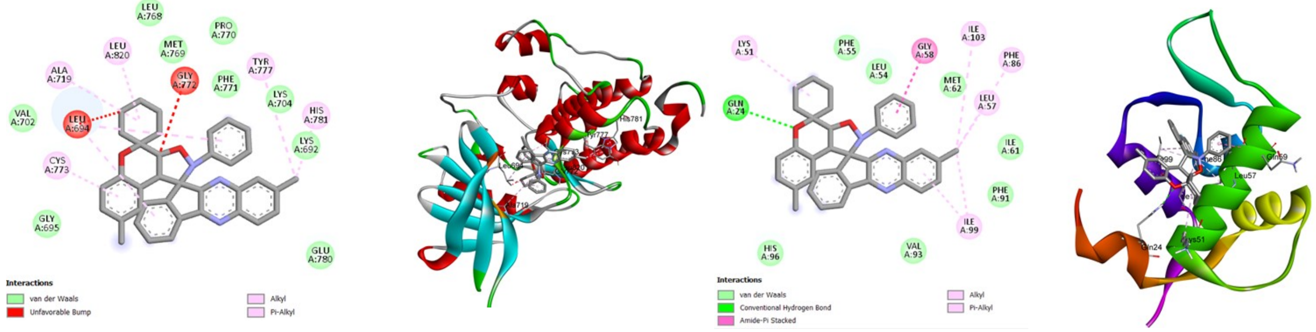

A B

Fig. S94: 2D and 3D docking illustrations of compound **21q** in the active sites EGFR protein **[A]** (PDB ID: 4HJO) and MDM2 protein **[B]** (PDB ID: 5LAV)

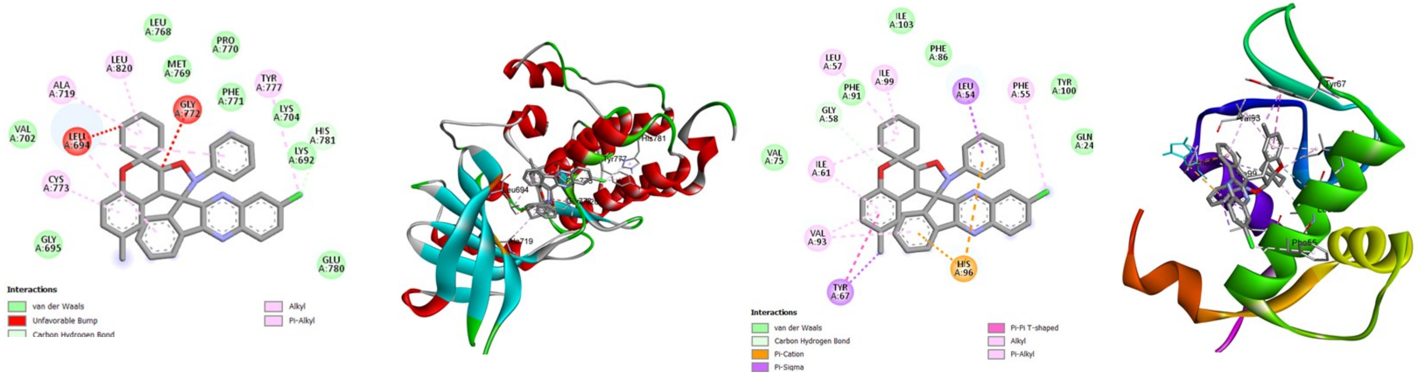

A **B**

Fig. S95: 2D and 3D docking illustrations of compound **21r** in the active sites EGFR protein **[A]** (PDB ID: 4HJO) and MDM2 protein **[B]** (PDB ID: 5LAV)

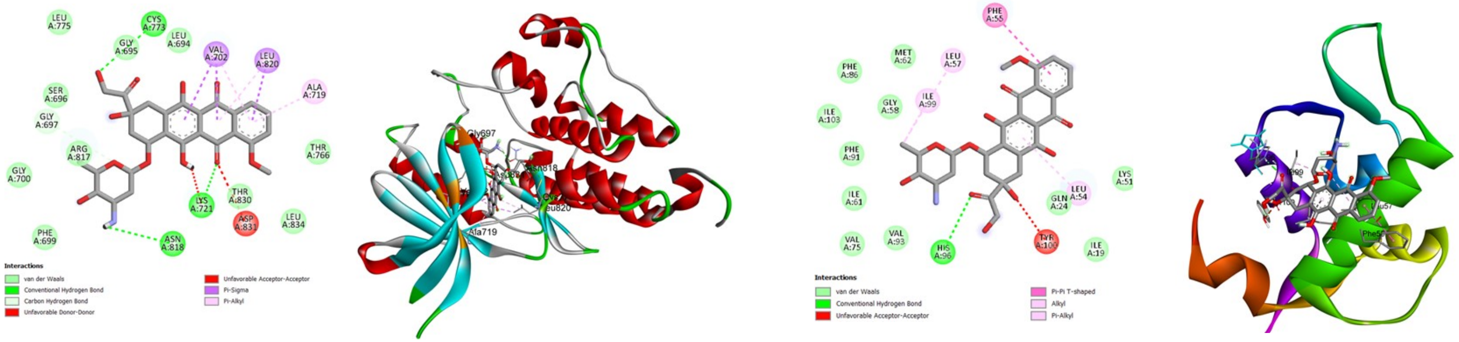

A **B**

Fig. S96: 2D and 3D docking illustrations of standard drug **Doxorubicin** in the active sites EGFR protein **[A]** (PDB ID: 4HJO) and MDM2 protein **[B]** (PDB ID: 5LAV)

Table S2 Molecular docking studies of bacterial DNA Gyrase with Compounds **21(a-r)**

Compounds	*E. coli* DNA Gyrase (PDBID- 1KZN)		*S. aureus* DNA Gyrase (PDBID- 3G7B)	
	Score (kcal mol^{-1})	Binding interactions	Score (kcal mol^{-1})	Binding interactions
21a	-9.7	Hydrophobic contacts: PRO68(Pi-alkyl), GLY66, THR142, GLY64, ASP62, ALA36, ILE67(Pi-alkyl), ARG65, ASP38, ASN35, HIS83, ALA84, GLY96, ILE78(Pi-sigma), VAL97, ALA74, GLU39	-8.7	Hydrophobic contacts: ASP34, SER32, ALA38, ASP58, THR127, GLY62, GLU35(Pi-anion), ASN31, ILE63(Pi-alkyl), ARG98, ARG61(Pi-cation), PRO64, ILE79(Pi-alkyl)
21b	-7.0	Hydrogen bond: ASN35<br>Hydrophobic contacts: MET79, ILE67(Pi-sigma), ILE78(Pi-sigma), VAL97, GLU39, ASP34, ALA84, ASP38, ALA42, ARG65, THR142, ASP62, ALA74, PRO68	-9.0	Hydrogen bond: PRO64<br>Hydrophobic contacts: ILE79(Pi-alkyl), ARG61, ASP34, ASN31, ALA38, ILE63(Pi-alkyl), GLU35, THR127, SER32, ASP58, GLY62
21c	-9.3	Hydrophobic contacts: PRO68, ILE78(Pi-sigma), VSL81, ALA84(Pi-alkyl), SER98, VAL95, HIS83, PHE88, GLY96, GLY94, VAL97, MET79, THR 142, ASN 35, ALA 36, ASP 38, ILE 67(Pi-alkyl), GLU 39, ALA 42(Pi-alkyl), ARG 65(Pi-cation)	-8.3	Hydrogen bond: GLU35<br>Hydrophobic contacts: ARG98, GLY62, PRO64(Pi-alkyl), ARG61(Pi-cation), ASP58, THR127, SER32, ILE63(Pi-alkyl), ILE129, ASN31, ILE79(alkyl), ASP34, ALA38
21d	-9.5	Hydrogen bond: ASN35<br>Hydrophobic contacts: ASP62, THR142, GLY66(Amide Pi), ARG65, ILE67(Pi-alkyl), GLU39, PRO68(Alkyl), ILE78, ALA42, ALA84, GLY85, GLY96, GLY86, GLU31, GLY94, ASP34, ASP38	-7.3	Hydrogen bond: ASN31<br>Hydrophobic contacts: ILE28, SER82, VAL85(Pi-alkyl), GLU27, VAL84, SER83, ILE63(Pi-alkyl), ILE79, ASP34, ASP30

21e	-9.3	Hydrophobic contacts: PRO68, GLY66, GLY64, THR142, ASP62, ALA36, ILE67, ARG69, ASP38, ASN35, ILE78(Pi-alkyl), HIS83, GLY96, VAL81, AL84(Pi-alkyl), SER 98, VAL97, GLU39	-8.8	Hydrophobic contacts: ASP34, ASN31, ALA38(Pi-alkyl), SER32, ASP58, THR127, GLY62, GLU35(Pi-anion), ARG61, ILE63(Pi-alkyl), ARG98, PRO64, ILE79
21f	-8.4	Hydrogen bond: ASN35<br>Hydrophobic contacts: ASP62, THR142, ALA36, ILE67(Pi-alkyl), GLY64, ILE78(Pi-sigma), GLU39, ARG65, VAL97, GLY96, ASP38, ALA84, ALA42, GLY66, PRO68(Pi-alkyl), ALA74	-8.5	Hydrophobic contacts: ASP34, ALA38(Pi-alkyl), SER32, ASP58, THR127, ASN31, ARG61(Pi-anion), GLU35(Pi-cation), GLY62, ILE63(Pi-alkyl), ARG98, PRO64, ILE79(Pi-alkyl)
21g	-9.2	Hydrophobic contacts: ALA42(Alkyl), GLY96, GLY94, GLU31, ARG65, ASP34, ASP38, ALA36, THR142, ASP62, ASN35(Amide pi-stacked), GLU39, ILE67(Pi-alkyl), ILE78, PRO68, HIS83, ALA84	-7.2	Hydrogen bond: ASN31<br>Hydrophobic contacts: ARG98, PRO64, GLY62, ILE79, GLU35(Pi-anion), ILE63, THR127, ILE129, LEU80, ASP34, ALA38(alkyl), ARG61(Pi-cation)
21h	-9.2	Hydrogen bond: PRO 68, ARG65<br>Hydrophobic contacts: VAL97, GLY96, ASN35, ASP38, ALA42(Pi-sigma), ALA36, GLU39(Pi-Anion), THR142, ILE67(Pi-alkyl), ALA75, ALA74, ILE78(Pi-sigma)	-7.7	Hydrophobic contacts:ASP34, ALA38, SER32, ASP58, THR127, GLU35(Pi-Anion), GLY62, ASN31, ARG61(Pi-cation), PRO64(Pi-alkyl), ILE63(Pi-alkyl), ARG98, ILE79
21i	-8.9	Hydrophobic contacts: GLY9, GLY96, HIS83, VAL97, ASN35, ALA84(Pi-alkyl), THR142, ILE67(Pi-sigma), ILE78, ASP62, ALA75, PRO68(Pi-alkyl), ALA74, GLU39, ARG65, ALA42, ASP38	-8.7	Hydrophobic contacts: ALA38(Pi-sigma), LEU37(Pi-sigma), ASP34, GLU35, ARG61, ARG98, PRO64, GLY62, SER32, SP58, ASN31(Amide Pi Stacked), THR127, ILE63, ILE79(Pi-sigma), ALA75

21j	-8.5	Hydrogen bond: ASN35<br>Hydrophobic contacts: VAL97, ILE78(Pi-alkyl), GLY96, ALA84, ASP38, ALA42(Pi-alkyl), ARG65, PRO68, GLU39, GLY66, GLY64, ASP62, THR142, ILE67(Pi-alkyl)	-7.5	Hydrophobic contacts:PRO64(Pi-alkyl), ARG61(Pi-alkyl), ARG98, ALA38(Pi-alkyl), GLU35, ASP4, ILE63, SER32, ASP58, THR127, ILE129, ASN31, ILE79
21k	-9.8	Hydrophobic contacts: PRO68, ILE78(Pi-sigma), VAL81, ALA84, SER98, VAL95, HIS83, PHE88, GLY96, GLY94, VAL97, ASN35, THR142, ALA36, ASP38, ILE67(Pi-alkyl), GLU39, ALA42(Pi-alkyl), ARG65(Pi-cation)	-8.2	Hydrogen bond: GLU35<br>Hydrophobic contacts: ARG98, PRO64, ARG61, ASP58, THR127, SER32, ILE63(Pi-alkyl), ASN31, GLY62(Amide pi-stacked), ILE129, ASP34, ALA38, ILE79(Pi-alkyl)
21l	-9.0	Hydrophobic contacts: PRO68, VAL81, ILE67(alkyl), HIS83, ALA84(Pi-alkyl), LEU82, ALA84, SER98, ILE78(Pi-alkyl), GLY96, VAL97, GLY86, LYS87, GLY85, GLU31, GLY94, ASN35, ASP38, ALA36, GLU39	-7.7	Hydrogen bond: PRO64, GLU35<br>Hydrophobic contacts: ARG98, GLY62, THR127, ASP58, SER32, ILE63(alkyl), ASN31, ALA38, ALA38, ASP34, ILE79(Pi-alkyl), ARG61
21m	-9.1	Hydrogen bond: ASN35<br>Hydrophobic contacts: PRO68(Pi-alkyl), ARG65, HIS44, ALA42(Pi-alkyl), GLU39, ASP38, ILE67(Pi-alkyl), THR142, SER98, VAL97, ALA84(Pi-alkyl), GLY96, HIS83, ILE78(Pi-sigma), ALA74	-7.7	Hydrophobic contacts: ALA75, ILE79(Pi-alkyl), ASN31, SER32, ASP58, THR127, GLU35, GLY62, ASP34, ILE63(Pi-alkyl), ARG98, ARG61, PRO64(Pi-alkyl)
21n	-8.7	Hydrophobic contacts: ASP38, ASP34, GLY64, ALA36, THR142, ASP62, GLU39(Pi-anion), ARG65, GLY66, PRO68, ILE67(Pi-sigma), VAL97, GLY96, ILE78(Pi-sigma), SER98, HIS83, ALA84, GLY86, GLY85	-8.8	Hydrophobic contacts: ASP34, ALA38(Pi-alkyl), ASN31, SER32, ASP58, THR127, GLY62, GLU35(Pi-cation), ARG61(Pi-anion), ILE63(Pi-alkyl), ARG98, PRO64, ILE79(Pi-alkyl)

21o	-8.6	Hydrophobic contacts: VAL97, SER98, GLY96, PHE88, HIS83, ALA84, ILE78(Pi-alkyl), ALA74(Pi-alkyl), PRO68(Pi-alkyl), ILE67(Pi-alkyl), ASP34, ASP38, ASN35, ARG65	-7.4	Hydrogen bond: ASN31<br>Hydrophobic contacts: VAL85, VAL84(Pi-alkyl), GLU27, SER83, SER82, ILE79(Pi-alkyl), ILE63, GLU35(Pi-anion), PRO64, ARG61, ALA38, ASP34
21p	-9.6	Hydrogen bond: ASN35<br>Hydrophobic contacts: SER98, VAL97, ILE67, THR142, ASP62, ALA36, GLY66, GLU39(Pi-anion), ARG65, ASP38, ALA42(Pi-alkyl), ALA74, PRO68, ILE78(Pi-sigma), GLU31, GLY94	-7.4	Hydrogen bond: GLU35<br>Hydrophobic contacts: ALA38, ASP34, ALA75, ILE79(Pi-sigma), ASN31, LEU80, ILE63(Pi-alkyl), PRO64(Pi-alkyl), ARG98, GLY62, ARG61
21q	-9.1	Hydrophobic contacts: GLY94, ASP34, ASN35, GLU31, ALA36, ASP38, ASP62, ILE67, THR142, GLU39(Pi-anion), GLY66, ARG65, VAL97, ALA84, GLY96, PRO68, SER98, HIS83, ILE78((Pi-sigma), GLY85	-8.5	Hydrophobic contacts:ASP34, ALA38, SER32, ASP58, THR127, GLY62, GLU35(Pi-anion), ASN31, ARG61(Pi-cation), ARG98, PRO64, ILE63, ILE79
21r	-9.4	Hydrophobic contacts: LEU41, THR142, ASP38, ASP62, ALA36, ASN35, ASP34, GLU31(Pi-anion), GLT94, GLY96, HIS83, ALA84(Pi-alkyl), ILE78, PRO68, VAL97, ILE67, GLU39, ARG65(Pi-cation), ALA42(Pi-alkyl)	-8.2	Hydrophobic contacts: ASP34(Pi-anion), ILE79(Pi-sigma), PRO64, GLY62, LEU80, ILE63(Pi-alkyl), ASN31 SER32, GLU35, ARG61, ALA38
Gentamicin	-6.2	Hydrogen bond: ALA82, ASN32, GLU28<br>Hydrophobic interactions: PRO65, GLY63, THR132, GLU36, ARG62, ASP59, ALA33, GLY86, ASP35, ASP31, GLY84, HIS81, VAL87,	-6.0	Hydrogen bond: SER82, GLU35, SER83<br>Hydrophobic interactions: GLU27, VAL85, ILE79, ASN31, ILE129(alkyl), THR127, SER32, ASP58, ILE63, GLY62, ARG61, PRO64, ASP34

	ILE76(Pi-alkyl), ILE64(Pi-alkyl)

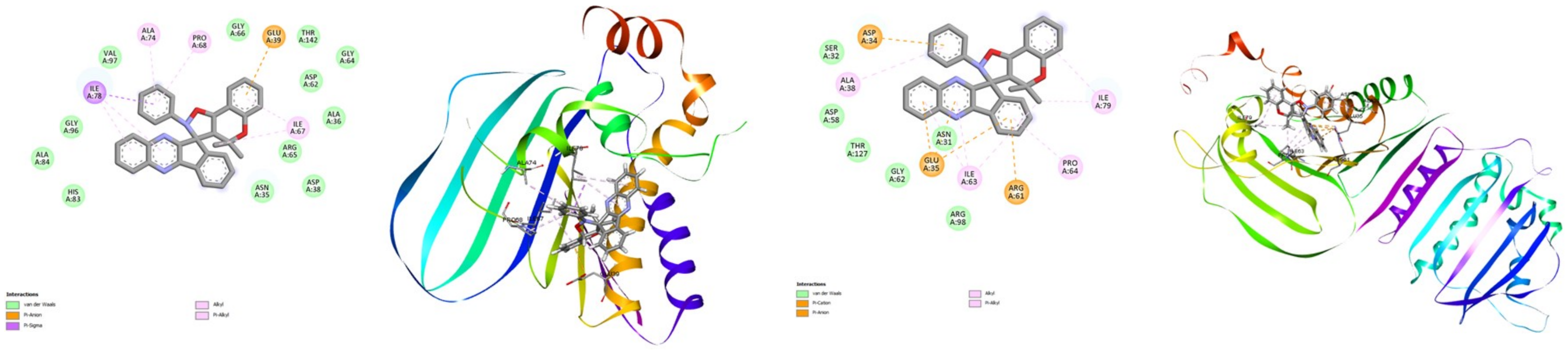

A **B**

Fig. S97. 2D Docking interaction and 3D binding image of compound **21a** with *E. coli* DNA gyrase **[A]** (PDB ID: 1KZN) and *S. aureus* DNA gyrase**[B]** *(*PDB ID: 3G7B)

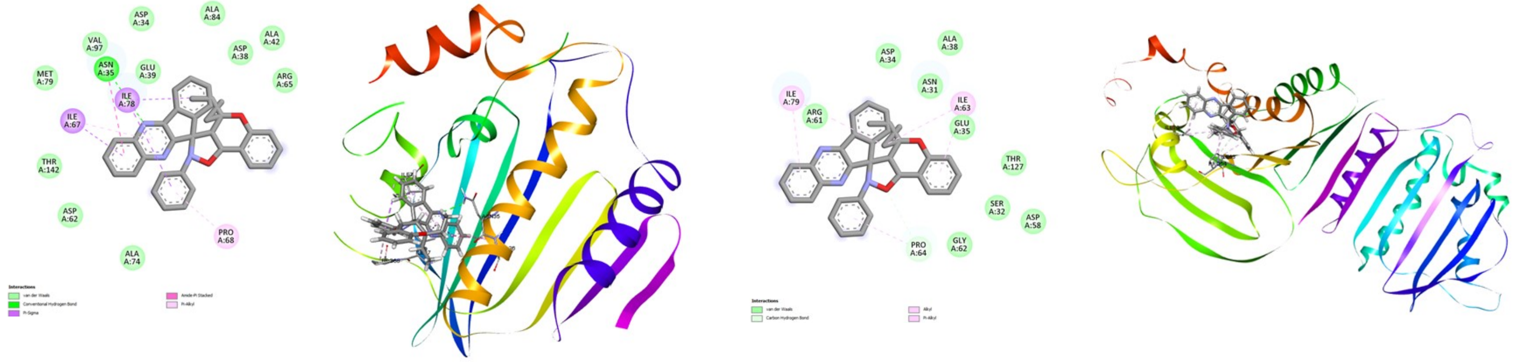

A **B**

Fig. S98: 2D Docking interaction and 3D binding image of compound **21b** with *E. coli* DNA gyrase **[A]** (PDB ID: 1KZN) and *S. aureus* DNA gyrase**[B]** *(*PDB ID: 3G7B)

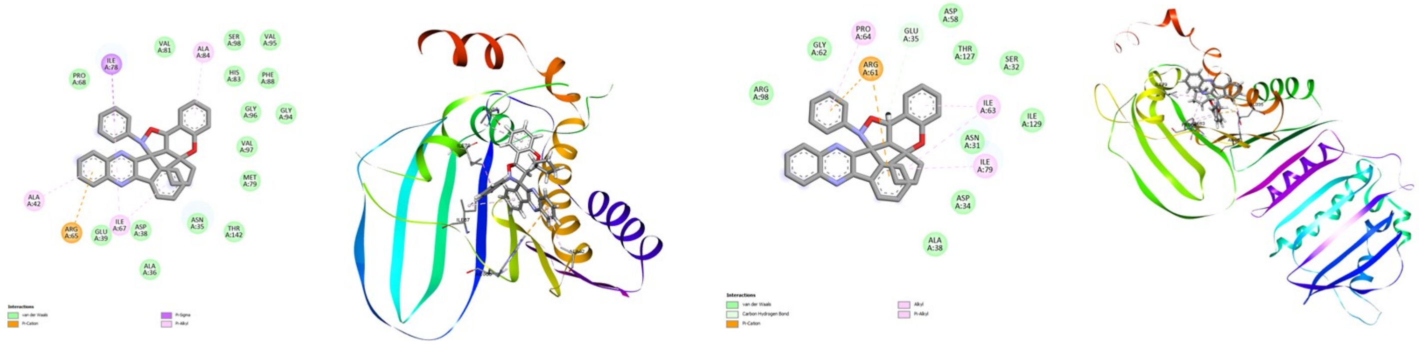

A **B**

Fig. S99: 2D Docking interaction and 3D binding image of compound **21c** with *E. coli* DNA gyrase **[A]** (PDB ID: 1KZN) and *S. aureus* DNA gyrase**[B]** *(*PDB ID: 3G7B)

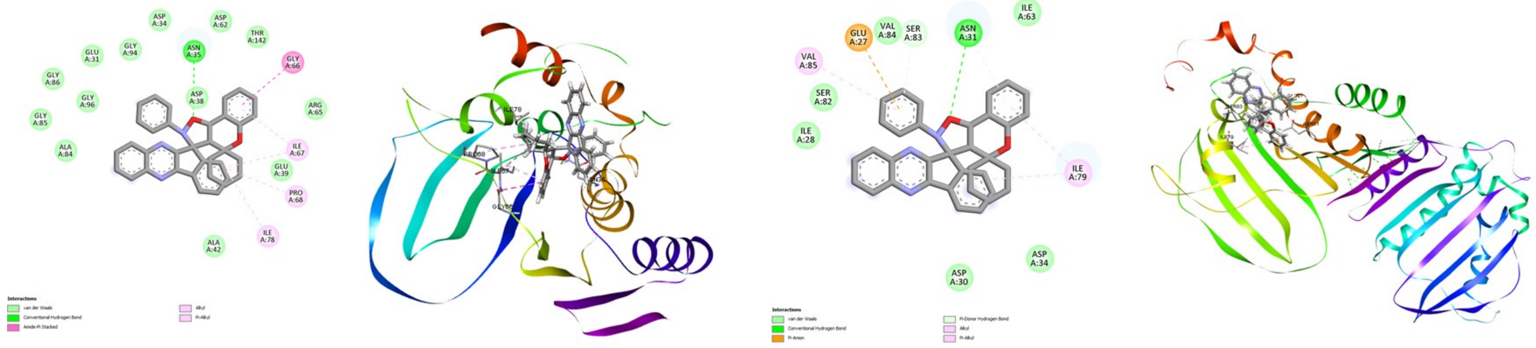

A **B**

Fig. S100: 2D Docking interaction and 3D binding image of compound **21d** with *E. coli* DNA gyrase **[A]** (PDB ID: 1KZN) and *S. aureus* DNA gyrase**[B]** *(*PDB ID: 3G7B)

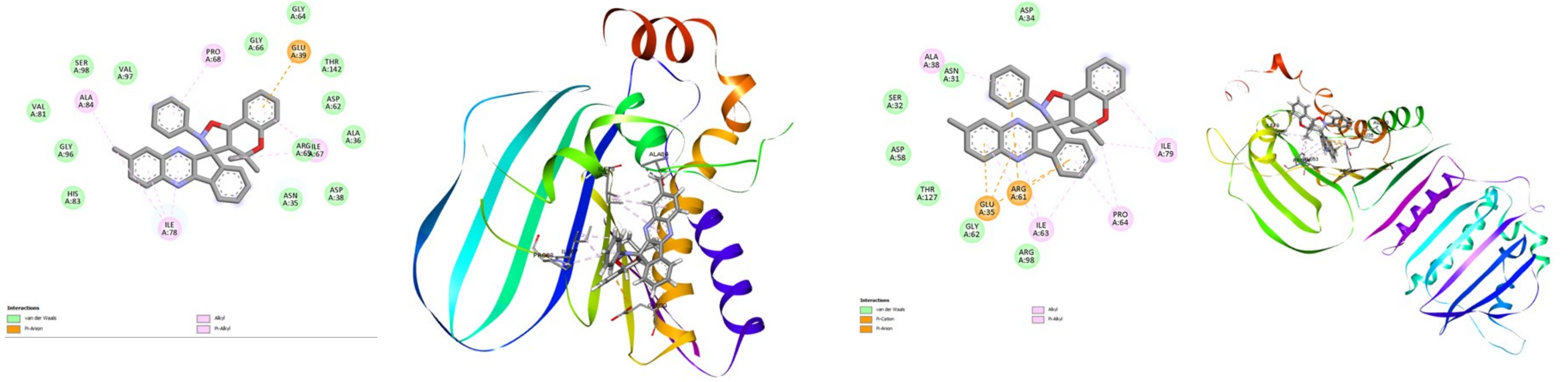

A **B**

Fig. S101: 2D Docking interaction and 3D binding image of compound **21e** with *E. coli* DNA gyrase **[A]** (PDB ID: 1KZN) and *S. aureus* DNA gyrase**[B]** *(*PDB ID: 3G7B)

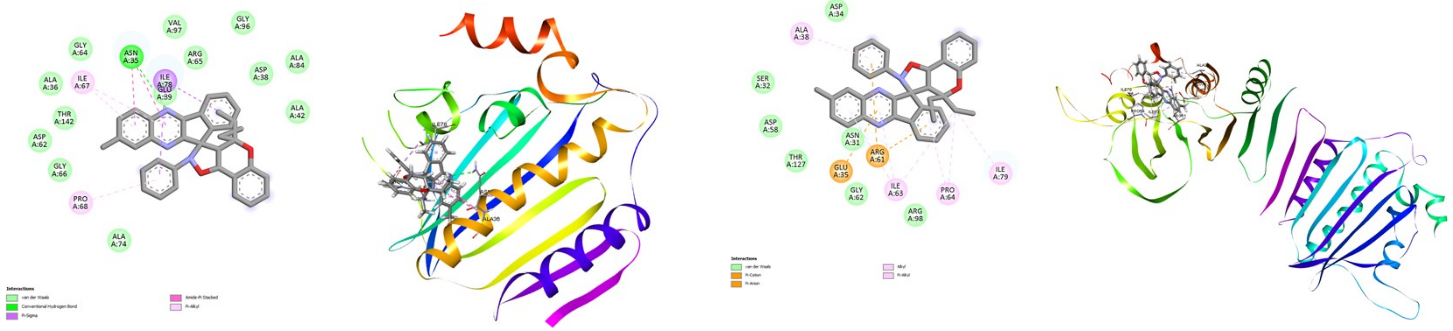

Fig. S102: 2D Docking interaction and 3D binding image of compound **21f** with *E. coli* DNA gyrase **[A]** (PDB ID: 1KZN) and *S. aureus* DNA gyrase**[B]** *(*PDB ID: 3G7B)

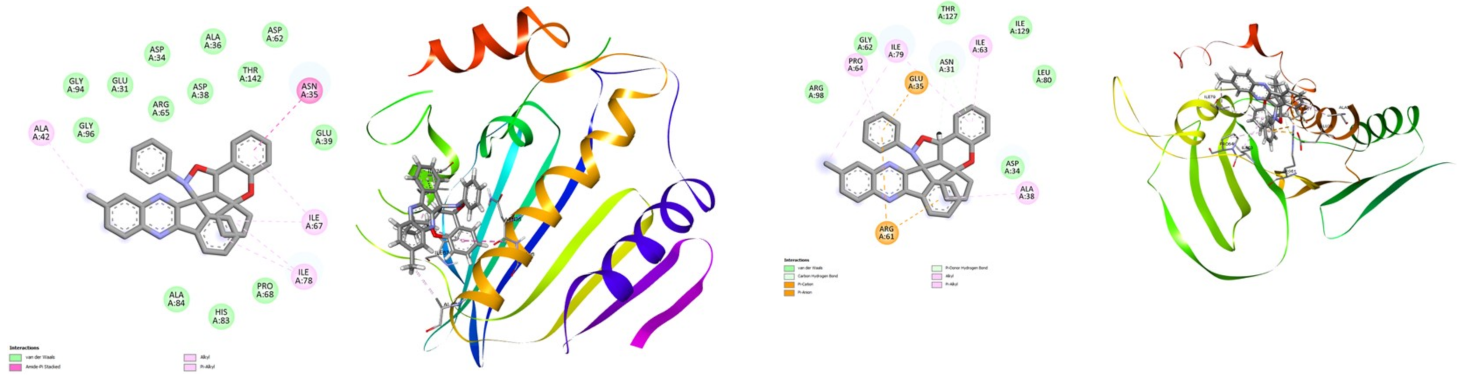

A **B**

Fig. S103: 2D Docking interaction and 3D binding image of compound **21g** with *E. coli* DNA gyrase **[A]** (PDB ID: 1KZN) and *S. aureus* DNA gyrase**[B]** *(*PDB ID: 3G7B)

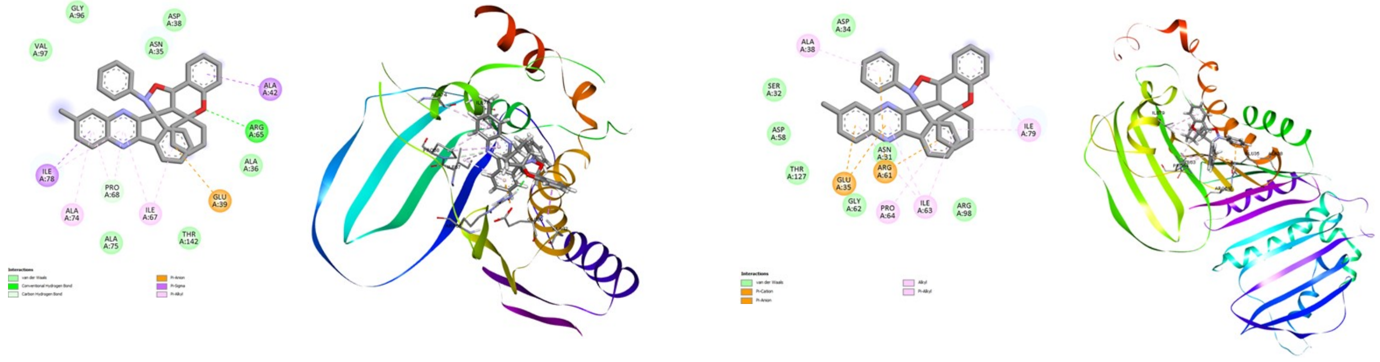

A **B**

Fig. S104: 2D Docking interaction and 3D binding image of compound **21h** with *E. coli* DNA gyrase **[A]** (PDB ID: 1KZN) and *S. aureus* DNA gyrase**[B]** *(*PDB ID: 3G7B)

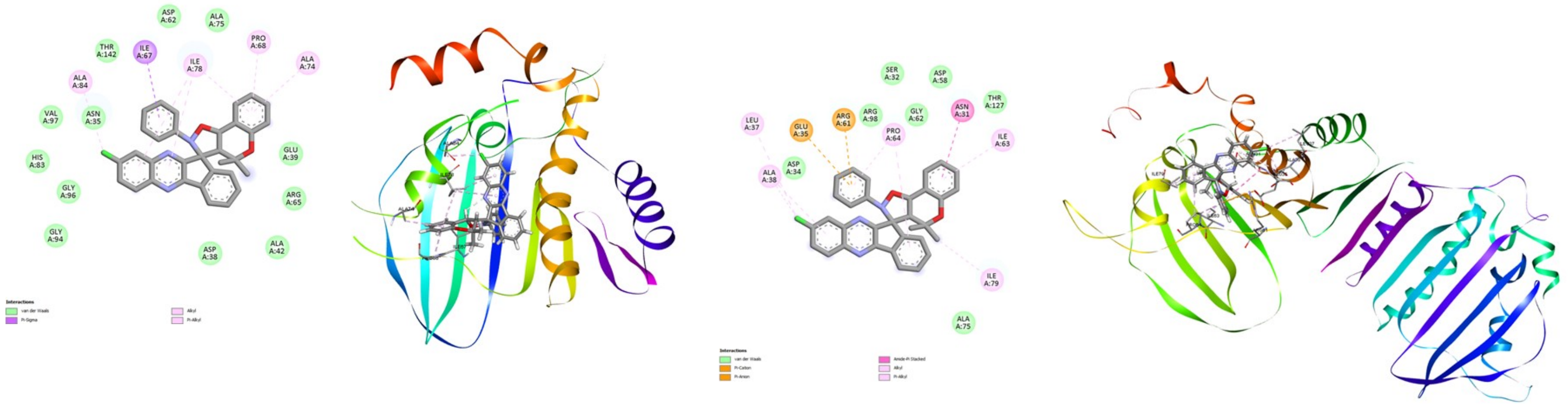

A **B**

Fig. S105: 2D Docking interaction and 3D binding image of compound **21i** with *E. coli* DNA gyrase **[A]** (PDB ID: 1KZN) and *S. aureus* DNA gyrase**[B]** *(*PDB ID: 3G7B)

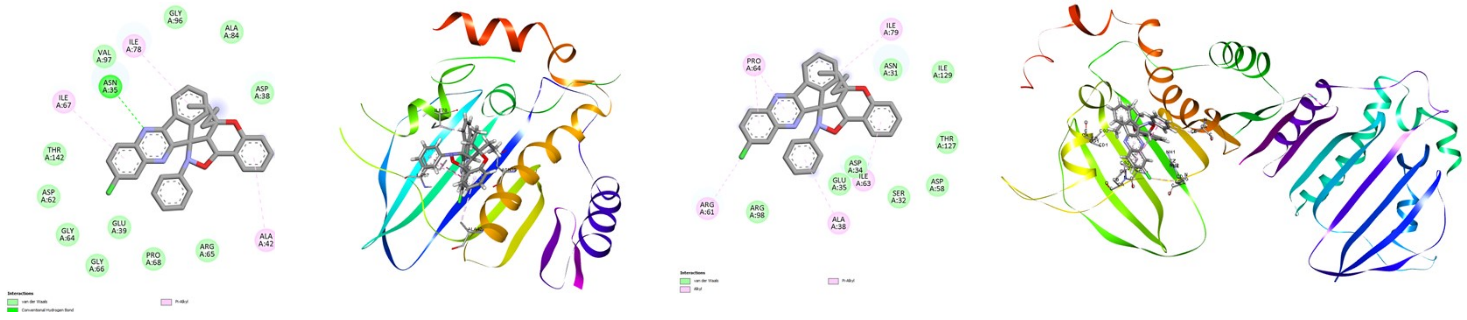

A **B**

Fig. S106: 2D Docking interaction and 3D binding image of compound **21j** with *E. coli* DNA gyrase **[A]** (PDB ID: 1KZN) and *S. aureus* DNA gyrase**[B]** *(*PDB ID: 3G7B)

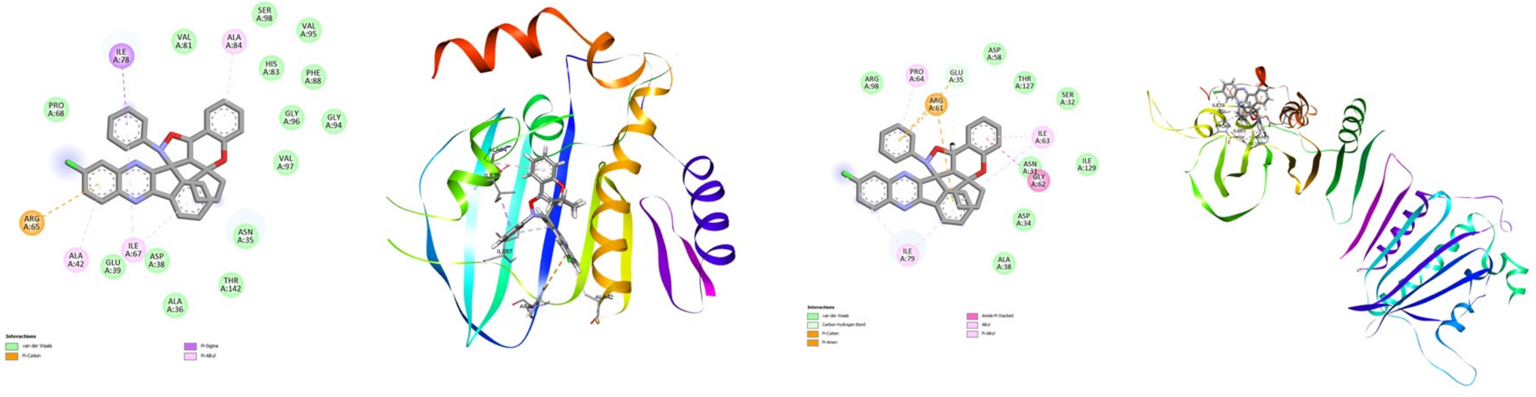

A B

Fig. S107: 2D Docking interaction and 3D binding image of compound **21k** with *E. coli* DNA gyrase **[A]** (PDB ID: 1KZN) and *S. aureus* DNA gyrase**[B]** *(*PDB ID: 3G7B)

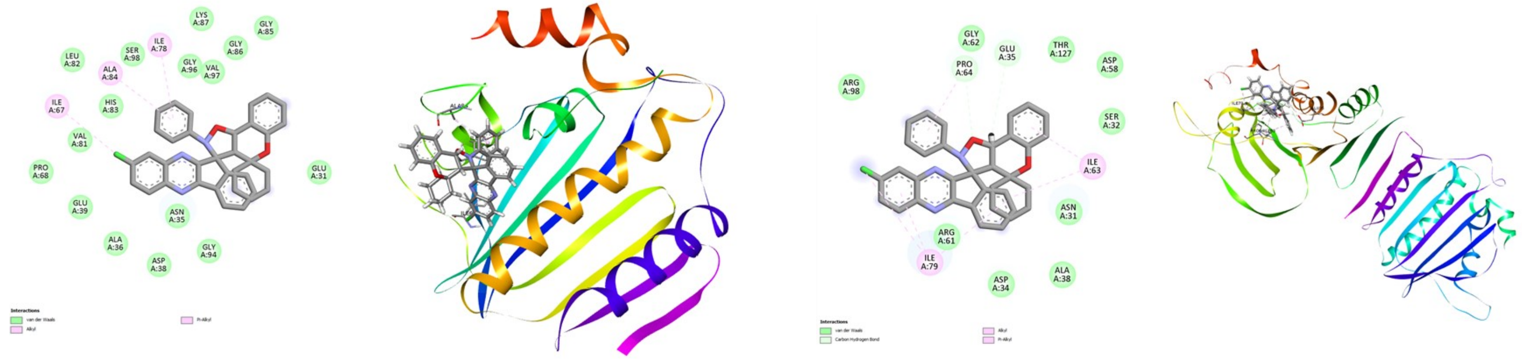

A B

Fig. S108: 2D Docking interaction and 3D binding image of compound **21l** with *E. coli* DNA gyrase **[A]** (PDB ID: 1KZN) and *S. aureus* DNA gyrase**[B]** *(*PDB ID: 3G7B)

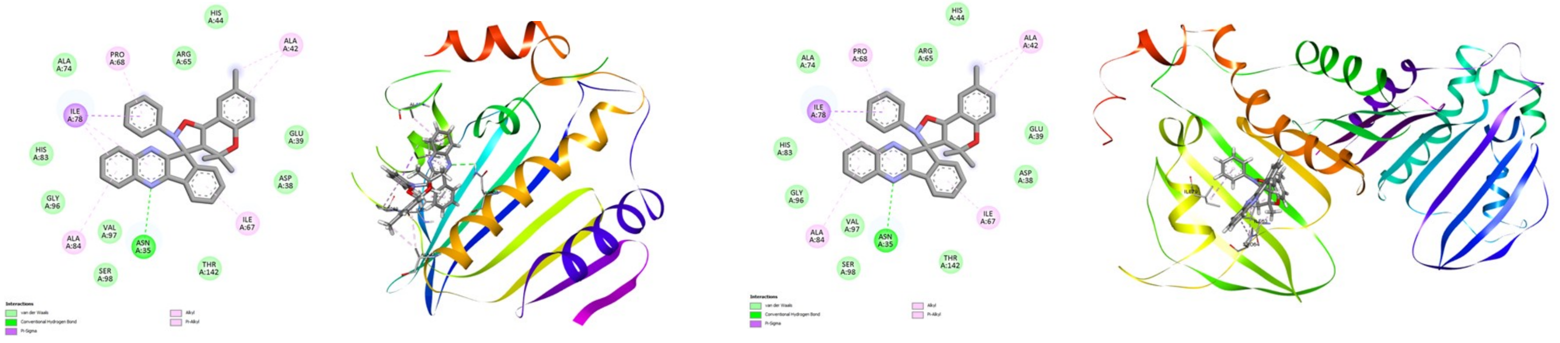

A **B**

Fig. S109: 2D Docking interaction and 3D binding image of compound **21m** with *E. coli* DNA gyrase **[A]** (PDB ID: 1KZN) and *S. aureus* DNA gyrase**[B]** *(*PDB ID: 3G7B)

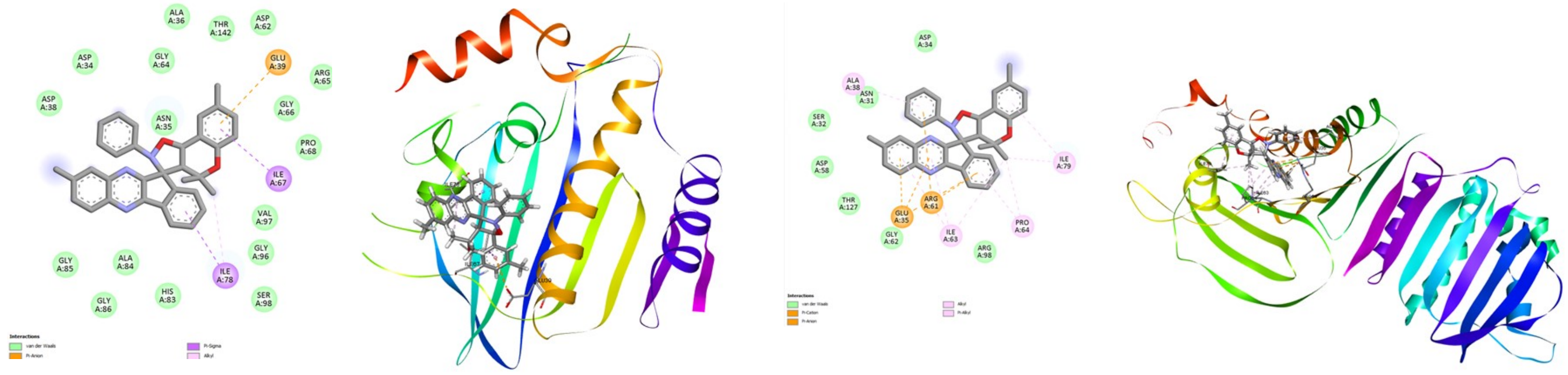

A **B**

Fig. S110: 2D Docking interaction and 3D binding image of compound **21n** with *E. coli* DNA gyrase **[A]** (PDB ID: 1KZN) and *S. aureus* DNA gyrase**[B]** *(*PDB ID: 3G7B)

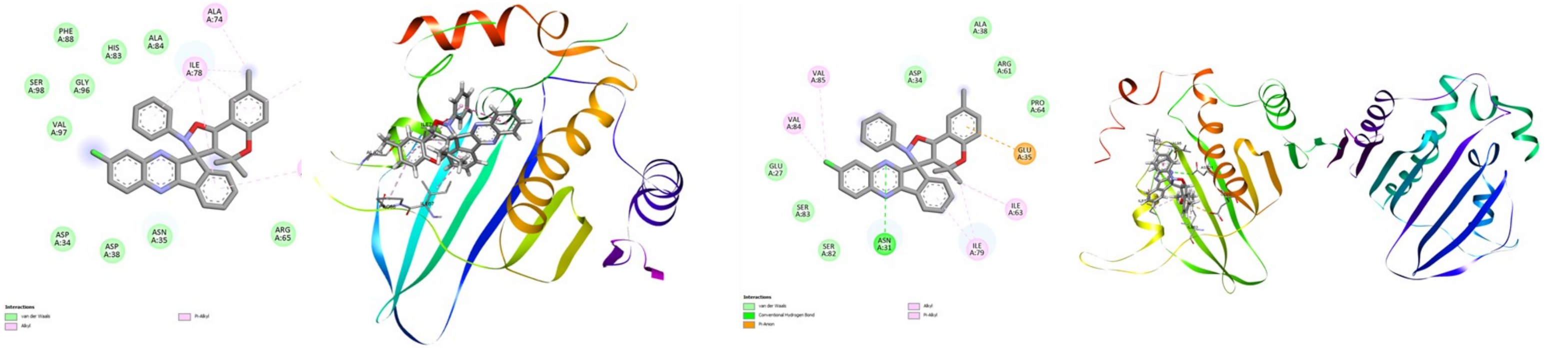

A **B**

Fig. S111: 2D Docking interaction and 3D binding image of compound **21o** with *E. coli* DNA gyrase **[A]** (PDB ID: 1KZN) and *S. aureus* DNA gyrase**[B]** *(*PDB ID: 3G7B)

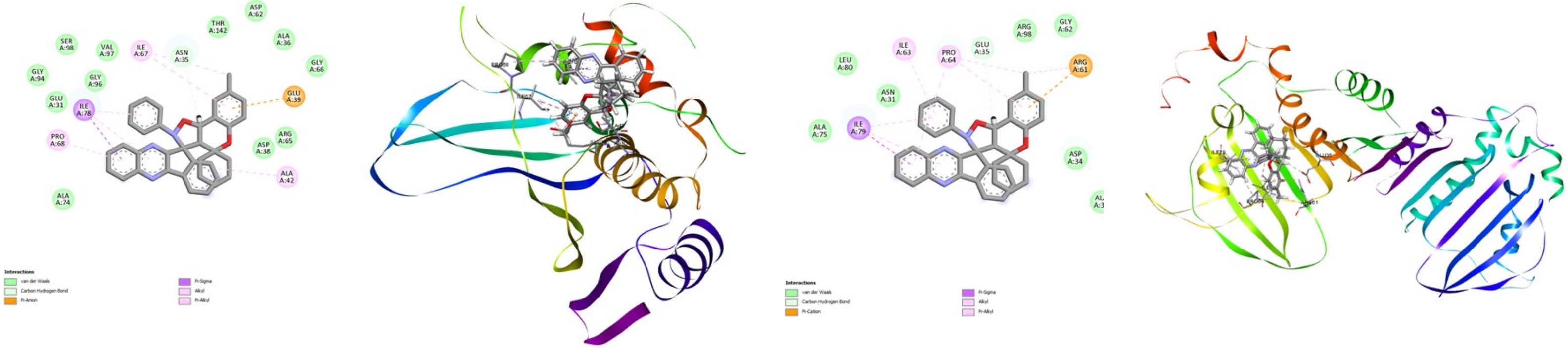

A **B**

Fig. S112: 2D Docking interaction and 3D binding image of compound **21p** with *E. coli* DNA gyrase **[A]** (PDB ID: 1KZN) and *S. aureus* DNA gyrase**[B]** *(*PDB ID: 3G7B)

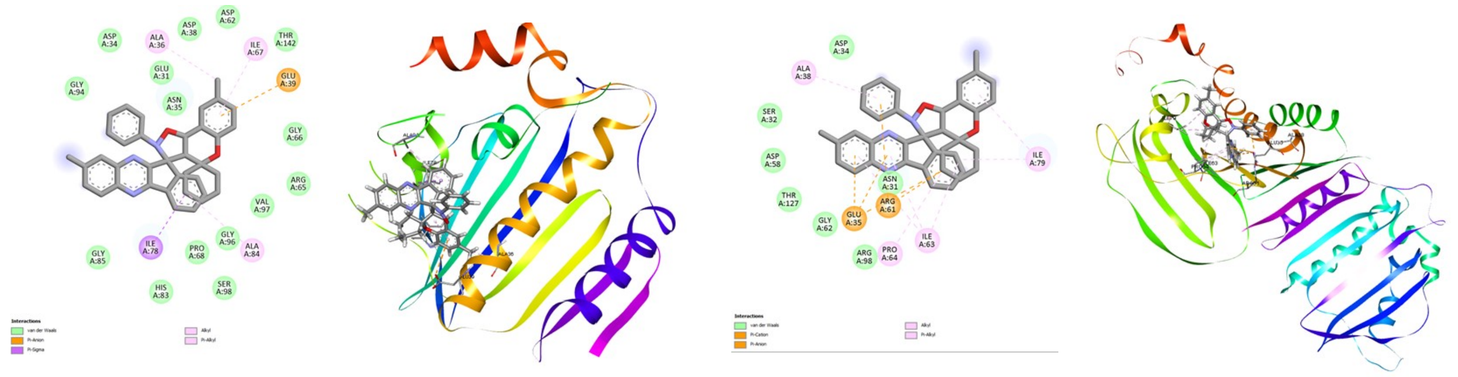

A **B**

Fig. S113: 2D Docking interaction and 3D binding image of compound **21q** with *E. coli* DNA gyrase **[A]** (PDB ID: 1KZN) and *S. aureus* DNA gyrase**[B]** *(*PDB ID: 3G7B)

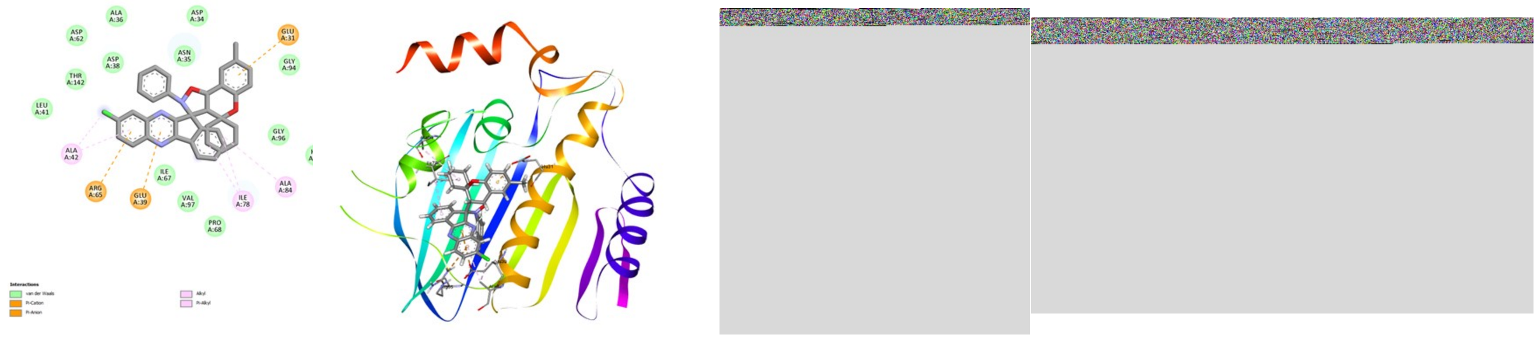

Fig. S114. 2D Docking interaction and 3D binding image of compound **21r** with *E. coli* DNA gyrase (PDB ID: 1KZN) [A] and *S. aureus DNA gyrase (*PDB ID: 3G7B)[B]

Table S3. Physicochemical properties, medicinal chemistry properties and physicochemical properties-based drug likeness rules, bioavailability score and drug-likeness model score of the four potent compounds (**21k**, **21b**, **21j**, and **21r)** and two standard drugs (Gentamicin and Doxorubicin) calculated using SwissADME and ADMETlab 3.0 web tools

**Properties**	**Compounds**				**Gentamicin**	**Doxorubicin**	**Recommended Limit**
	**21k**	**21b**	**21j**	**21r**			
**Physicochemical**							
MW	543.17	511.23	545.19	571.2	477.60	543.17	100 to 600 g/mol
^{n}HA	5.0	5.0	5.0	5.0	12.0	12.0	0 to 12
^{n}HD	0.0	0.0	0.0	0.0	8.0	7.0	0 to7
^{n}Ring	9.0	8.0	8.0	9.0	3.0	5.0	0 to 6
^{f}Char	0.0	0.0	0.0	0.0	0.0	0.0	-4 to 4
^{n}Het	6.0	5.0	6.0	6.0	12.0	12.0	1 to 15
^{n}Rig	46.0	41.0	41.0	47.0	18.0	30.0	0 to 30
^{Max}Ring	17.0	17.0	17.0	17.0	6.0	18.0	0 to 18
Log D	4.75	4.75	5.07	5.19	0.44	1.72	1 to 3
Log S	-8.62	-7.40	-8.452	-9.11	-1.40	-2.879	-4 to 0.5 mol/l
Log P	6.597	6.247	7.039	7.485	-0.168	1.219	0 to 5 log octanol/water
Csp^{3}	0.24	0.24	0.24	0.28	1.0	0.44	≥ 0.42
MR	159.34	156.44	161.45	169.11	118.31	132.66	40 to 130 m^{3}/mol
TPSA	47.48	47.48	47.48	47.48	199.73	206.07	0 to 140 Å^{2}
^{n}RB	1	3	1	3	7	5	0 to11
%ABS	92.62%	92.62%	92.62%	92.62%	40.09%	37.91%	> 50%

**Medicinal Chemistry**							
PAINS	0	0	0	0	0	1	0 alerts
MCE-18	201.52	176.0	180.19	206.391	66.29	121.154	≥ 45
SA Score	6.15	5.98	5.96	6.38	6.51	5.81	≤ 6
NP Score	0.02	0.28	0.05	0.0	1.68	1.81	-5 to 5
BA Score	0.17	0.17	0.17	0.17	0.17	0.17	≥ 0.5
**Drug likeness rules**							
Lipinski's rule	Rejected (^{n}Vio = 2)	Rejected (^{n}Vio = 2)	Rejected (^{n}Vio = 2)	Rejected (^{n}Vio = 2)	Rejected (^{n}Vio = 2)	Rejected (^{n}Vio = 3)	MW ≤ 500, Log P ≤ 5, ^{n}HA ≤ 10, ^{n}HD ≤ 5, ^{n}Vio ≤ 1
Veber's rule	Accepted	Accepted	Accepted	Accepted	Rejected (^{n}Vio = 1)	Rejected (^{n}Vio = 1)	^{n}RB ≤ 10, TPSA ≥ 140 Å^2
Egan's rule	Accepted	Rejected (^{n}Vio = 1)	Rejected (^{n}Vio = 1)	Rejected (^{n}Vio = 1)	Rejected (^{n}Vio = 1)	Rejected (^{n}Vio = 1)	Log P ≤ 3, TPSA ≥ 75 Å^2

MW = Molecular weight (g/mol), ^{n}RB = Number of rotatable bonds, ^{n}HA = Number of hydrogen bond acceptors, ^{n}HD = Number of hydrogen bond donors, Log P = The logarithm of the n-octanol/water distribution coefficients at 7.4 pH, TPSA = Topological Polar Surface Area (Å^2), Csp^3 = sp^3 hybridized fraction of carbon atoms in a molecule, MR = Molar Refractivity (m^3/mol), Log S = The logarithm of aqueous solubility value, %ABS = Percentage of absorption rate, SA Score = Synthetic Accessibility Score, BA Score = Bioavailability Score, PAINS = Pan Assay Interference Structures, MCE-18 = Medicinal Chemistry Evolution, ^{n}Vio = The number of violations.

Table S4 ADMET properties of the four potent compounds (**21k**, **21b**, **21j**, and **21r)** and standard drugs (Gentamicin and Doxorubicin) calculated using ADMETlab 3.0, SwissADME, ProTox 3.0 and pkCSM web tools

ADMET Properties	Compounds				Gentamicin	Doxorubicin	Recommended Limit
	**21k**	**21b**	**21j**	**21r**			
**Absorption**							
Caco-2 Permeability	-4.728	-4.898	-4.822	-4.797	-5.716	-6.259	≥ -5.15 log unit
MDCK Permeability	-4.734	-4.708	-4.736	-4.763	-5.343	-5.441	≤ 20 × 10^{-6} cm/s
Pgp-inhibitor	0.955	0.992	0.991	0.993	0.0	0.001	0 to 0.5
Pgp-substrate	0.003	0.061	0.022	0.001	1.0	0.993	0 to 0.5
HIA	0.0	0.0	0.0	0.0	1.0	0.999	0 to 1
GI absorption	Low	Low	Low	Low	Low	Low	High/Low
**Distribution**							
PPB	88.988	88.129	88.915	89.481	14.166	84.416	≤ 90%
BBB	1.0	1.0	1.0	1.0	0.0	0.0	< 0.5
VDss	0.279	0.308	0.329	0.374	-0.458	1.455	0.04 to 20 l/kg
Fu	0.395	1.354	0.503	0.23	83.152	15.574	≥ 20%
**Metabolism**							
CYP1A2 inhibitor	1.0	0.661	0.528	0.75	0.0	0.0	0 to 0.5
CYP1A2 substrate	0.95	0.31	0.09	0.562	0.0	0.002	0 to 0.5
CYP2C19 inhibitor	0.999	0.999	0.994	1.0	0.0	0.0	0 to 0.5
CYP2C19 substrate	0.896	0.997	0.994	0.984	1.0	0.06	0 to 0.5
CYP2C9 inhibitor	1.0	1.0	1.0	1.0	0.0	0.0	0 to 0.5
CYP2C9 substrate	0.999	0.933	0.882	0.98	0.0	0.0	0 to 0.5
CYP2D6 inhibitor	0.647	0.007	0.004	0.057	0.0	0.0	0 to 0.5

CYP2D6 substrate	0.974	0.037	0.032	0.987	1.0	0.0	0 to 0.5
CYP3A4 inhibitor	0.864	0.920	0.628	0.988	0.0	0.001	0 to 0.5
CYP3A4 substrate	0.516	0.759	0.973	0.992	1.0	0.143	0 to 0.5
CYP2B6 inhibitor	0.833	0.645	0.757	0.996	0.0	0.0	0 to 0.5
CYP2B6 substrate	0.837	0.039	0.01	0.069	0.003	0.0	0 to 0.5
CYP2C8 inhibitor	1.0	1.0	1.0	1.0	0.0	0.0	0 to 0.5
**Excretion**							
CL_{plasma}	3.124	5.205	4.191	3.114	1.048	13.032	5 to 15 ml/min/kg
$T_{1/2}$	0.677	0.344	0.498	0.583	1.992	3.308	> 4h
**Toxicity**							
AMES toxicity	No	No	No	No	No	No	No
Max. tolerated dose	0.451	0.45	0.449	0.437	1.304	0.081	≥ 1.0 log mg/kg/day
hERG I inhibitor	No	No	No	No	No	No	No
hERG II inhibitor	Yes	Yes	Yes	Yes	No	Yes	No
Hepatotoxicity	No	No	No	No	No	Yes	No
LD_{50}	1000	1000	1000	1000	5000	205	≥ 2000 mg/kg
Toxicity Class	4	4	4	4	5	3	≥ 4

Caco-2 Permeability = Colon Adenocarcinoma Cell Line Permeability, MDCK = Madin-Darby Canine Kidney Cell Permeability, Pgp-inhibitor = P-glycoprotein Inhibitor, Pgp-substrate = P-glycoprotein Substrate, HIA = Human Intestinal Absorption, GI absorption = Gastrointestinal absorption, VDss = Volume of Distribution at Steady State, Fu = Fraction Unbound (in plasma), PPB = Plasma Protein Binding, BBB = Blood-Brain Barrier, CYP = Cytochrome, CL_{plasma} = Plasma Clearance, $T_{1/2}$ = Half-Life Time, AMES = Ames Test for Mutagenicity, hERG I = human Ether-à-go-go-Related Gene Channel 1, hERG II = human Ether-à-go-go-Related Gene Channel 2, LD_{50} = Lethal Dose for 50% of the test population.

Experimental Section

DFT studies

Density functional theory (DFT) calculations were performed using Gaussian 09W and GaussView 6.0 software [1,2]. The geometry of the reactants and products were optimized at the B3LYP/6-31G(d,p) level basic set [3,4]. Frontier molecular orbital energies (E_{HOMO} and E_{LUMO}) were used to derive global reactivity descriptors within the framework of conceptual DFT using Koopman's approximation [5]. These global reactivity descriptors include the ionization potential (I), electron affinity (A), electronegativity (χ), chemical potential (μ), global hardness (η), global softness (S), global electrophilicity index (ω), global nucleophilicity index (ε), and extra electronic charge (ΔN). The HOMO-LUMO energy gap (ΔE_g) and Gibbs free energy change (ΔG) were computed to evaluate the kinetic and thermodynamic favorability of the reactions. All the global reactivity descriptors, HOMO-LUMO energy gap and Gibbs free energy change have been computed using following mathematical equations (Eqs. 1-11) [6-9].

Energy Gap (ΔE_g) = $E_{LUMO} - E_{HOMO}$ (1)

Ionization Potential (I) = $- E_{HOMO}$ (2)

Electron Affinity (A) = $- E_{LUMO}$ (3)

Electronegativity (χ) = $- (E_{LUMO} + E_{HOMO})/2$ (4)

Chemical potential (μ) = $(E_{LUMO} + E_{HOMO})/2$ (5)

Global Hardness (η) = $(E_{LUMO} - E_{HOMO})/2$ (6)

Global Softness (S) = $1/2\eta$ (7)

Global Electrophilicity index (ω) = $\mu^2/2\eta$ (8)

Global Nucleophilicity index (ε) = $1/\omega$ (9)

Extra electronic charge (ΔN) = $- \mu/\eta$ (10)

Gibbs free energy change (ΔG) = $G_{product} - G_{reactant}$ (11)

Results and Discussion

DFT studies

To elucidate the mechanistic basis underlying the experimentally observed variation in reaction rates, a detailed DFT investigation was conducted using two representative substituent-containing compounds: (i) compound **21g** bearing a methyl group (-CH_3, electron-donating) and (ii) compound **21k** bearing a chloro group (-Cl, electron-withdrawing).

Frontier molecular orbital (FMO) analysis revealed a clear distinction between the two systems. The Cl-substituted compound **21k** exhibited a lower HOMO-LUMO energy gap (ΔE_g = 3.4849 eV) than the CH_3-substituted compound **21g** (ΔE_g = 3.6087 eV) (Table 5), indicating enhanced electronic softness and a greater propensity for orbital interactions. A reduced energy gap facilitates a more efficient redistribution of electron density between the reacting partners, thereby promoting bond formation and enhancing overall reactivity.

This trend is consistently supported by the global reactivity descriptors (Table 5). The Cl-substituted compound **21k** displayed lower chemical hardness (η = 1.7425 eV) and higher softness (S = 0.2869 eV^{-1}) than the CH_3-substituted compound **21g** (η = 1.8044 eV, S = 0.2771 eV^{-1}), reflecting increased polarizability and reduced resistance to electronic reorganization. Furthermore, the electrophilicity index (ω) was significantly higher for the Cl-substituted compound **21k** (4.7123 eV) than for the CH_3-substituted compound **21g** (4.1489 eV), indicating a stronger ability to accept electron density. This is further supported by the larger charge-transfer parameter (ΔN = 2.3257 vs. 2.1445), confirming that the Cl-substituted compound **21k** accommodates electronic redistribution more effectively during the reaction.

Gibbs free energy calculations further differentiated the two systems. The Cl-substituted reaction was more exergonic (ΔG = -12.1064 kcal/mol) than the CH_3-substituted analog (ΔG = -9.3667 kcal/mol) (Table 5), indicating greater stabilization of the product and overall reaction pathway. This enhanced stabilization arises from the electron-withdrawing nature of the Cl-substituent, which promotes electron delocalization and lowers the overall energy of the system.

Therefore, a direct correlation can be established between the substituent electronic properties and the observed reaction rates. The Cl-substituted compound **21k** simultaneously reduced the HOMO-LUMO energy gap, increased electrophilicity, enhanced softness, improved charge-transfer capability, and provided greater thermodynamic stabilization. These combined effects facilitate more efficient electronic interactions during the reaction, resulting in faster reaction rates. In contrast, the electron-donating methyl-substituted compound **21g** lead to a relatively larger energy

gap, lower electrophilicity, and diminished charge-transfer ability, thereby reducing electronic responsiveness and slowing the reaction. Accordingly, the substituent-dependent reactivity followed the trend: Cl-substituted compound **21k** > CH_3-substituted compound **21g**.

Table S5. Calculated HOMO-LUMO energy gap, gibbs free energy change and global electronic properties of synthesized CH_3-substituted and Cl-substituted dispiro-chromeno indenoquinoxaline compounds (**21g** and **21k**).

**Compound**	**E_{LUMO} (eV)**	**E_{HOMO} (eV)**	**ΔE_g (eV)**	**I (eV)**	**A (eV)**	**χ (eV)**	**μ (eV)**	**η (eV)**	**S (eV^{-1})**	**ω (eV)**	**ε (eV^{-1})**	**ΔN**	**ΔG (Kcal/mol)**
**21g**	-2.0650	-5.6737	3.6087	5.6738	2.0650	3.8694	-3.8694	1.8044	0.2771	4.1489	0.2410	2.1445	-9.3667
**21k**	-2.3099	-5.7948	3.4849	5.7948	2.3099	4.0524	-4.0524	1.7425	0.2869	4.7123	0.2122	2.3257	-12.1064

DFT studies

Optimized co-ordinates of compound **15b**, **15c**, **20c**, **21g** and **21k**

<u>Compound **15b**</u>

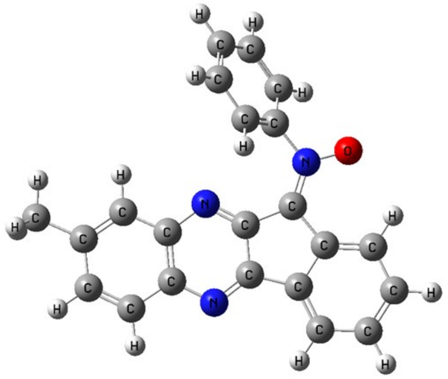

Coordinates (Angstrom)

Atom	X	Y	Z
C	4.473073	-2.398303	0.045835
C	3.694935	-3.565147	0.104992
C	2.295269	-3.487865	0.107124
C	1.700694	-2.230661	0.052902
C	2.484568	-1.040472	-0.002932
C	3.883066	-1.127384	-0.014979
C	0.293628	-1.848679	0.034184
C	0.209277	-0.401561	-0.010191
C	1.584008	0.118311	-0.024789
N	-0.772110	-2.630325	0.038394
C	-1.995125	-1.985303	-0.014136
C	-2.082673	-0.555591	-0.076639
N	-0.948305	0.236556	-0.067958
C	-3.189324	-2.744925	-0.012285
C	-4.415198	-2.111897	-0.074645
C	-4.517640	-0.689937	-0.142394
C	-3.353803	0.064033	-0.141439
N	2.000415	1.384054	-0.107307

C	1.135855	2.549824	0.018028
O	3.273063	1.677074	-0.312927
C	1.308092	3.574699	-0.917065
C	0.553383	4.742695	-0.792426
C	-0.341143	4.890498	0.275582
C	-0.480593	3.866533	1.220138
C	0.257879	2.686491	1.095357
C	-5.879433	-0.038553	-0.213021
H	5.555413	-2.478634	0.040140
H	4.181073	-4.534582	0.144708
H	1.680055	-4.379891	0.148301
H	4.479140	-0.228943	-0.075321
H	-3.103131	-3.824595	0.036663
H	-5.326581	-2.702909	-0.074219
H	-3.381256	1.147390	-0.190902
H	2.030650	3.448517	-1.713424
H	0.670676	5.538007	-1.520588
H	-0.919599	5.803096	0.376394
H	-1.159508	3.985916	2.057629
H	0.157965	1.892747	1.823882
H	-6.436595	-0.376687	-1.096045

H	-6.487245	-0.289897	0.665690
H	-5.798334	1.051049	-0.264599

Compound **15c**

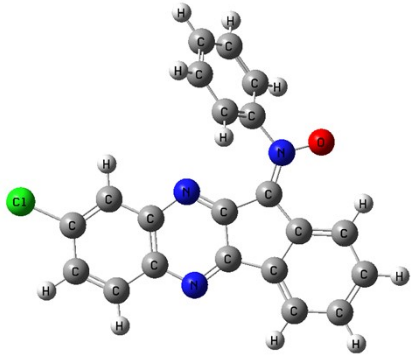

	Coordinates (Angstrom)		
Atom	X	Y	Z
C	4.779862	-2.293354	0.022377
C	4.026572	-3.472809	0.083875
C	2.631266	-3.421964	0.095494
C	2.014345	-2.176335	0.047472
C	2.771182	-0.976423	-0.012068
C	4.169150	-1.037372	-0.031573
C	0.598690	-1.826850	0.038814

C	0.481412	-0.381722	-0.003959
C	1.847439	0.161214	-0.023807
N	-0.435368	-2.632545	0.049939
C	-1.656118	-2.013549	0.006394
C	-1.774508	-0.586038	-0.056247
N	-0.676572	0.232695	-0.056936
C	-2.832691	-2.799872	0.016943
C	-4.075931	-2.208515	-0.035799
C	-4.174742	-0.799064	-0.101628
C	-3.058696	0.007110	-0.112307
N	2.239803	1.432937	-0.113566
C	1.333026	2.565213	0.018955
O	3.455057	1.755763	-0.318683
C	1.425486	3.569032	-0.944335
C	0.628814	4.703579	-0.814473
C	-0.222642	4.840196	0.284841
C	-0.280140	3.838676	1.255507
C	0.497086	2.688681	1.125652
Cl	-5.773787	-0.073280	-0.168765
H	5.864146	-2.353777	0.010071
H	4.532357	-4.433012	0.118614

H	2.030427	-4.324538	0.139325
H	4.750523	-0.128284	-0.091031
H	-2.723185	-3.878162	0.065553
H	-4.979595	-2.807068	-0.028869
H	-3.130575	1.087041	-0.161597
H	2.118686	3.450905	-1.768791
H	0.680552	5.485141	-1.565876
H	-0.834338	5.731158	0.389245
H	-0.927931	3.951608	2.119065
H	0.464651	1.908044	1.875927

<u>Compound **20c**</u>

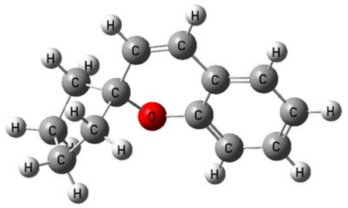

Coordinates (Angstrom)			
Atom	X	Y	Z
C	3.731474	-0.262214	0.194028

C	3.131872	-1.508136	-0.036616
C	1.751786	-1.602528	-0.246871
C	0.972951	-0.443888	-0.222747
C	1.556897	0.822080	-0.002594
C	2.944011	0.892593	0.205611
O	-0.386633	-0.572594	-0.484808
C	-1.330768	0.517932	-0.086012
C	-0.651344	1.861712	-0.111041
C	0.685068	1.993229	-0.041319
C	-1.934084	0.136496	1.291130
C	-2.973000	-0.979850	0.994055
C	-3.260431	-0.908025	-0.541537
C	-2.524119	0.354883	-1.046949
H	4.801523	-0.192951	0.358272
H	3.737383	-2.408754	-0.051013
H	1.267416	-2.554422	-0.430634
H	3.401359	1.863900	0.373656
H	-1.303534	2.730055	-0.143710
H	1.148329	2.975797	-0.024583
H	-2.421000	1.024866	1.714926
H	-1.147849	-0.169120	1.988136

H	-2.572903	-1.962644	1.261593
H	-3.884109	-0.831790	1.584085
H	-4.332453	-0.869765	-0.762055
H	-2.852955	-1.792405	-1.040601
H	-2.186052	0.278501	-2.083824
H	-3.163968	1.243774	-0.960606

Compound **21g**

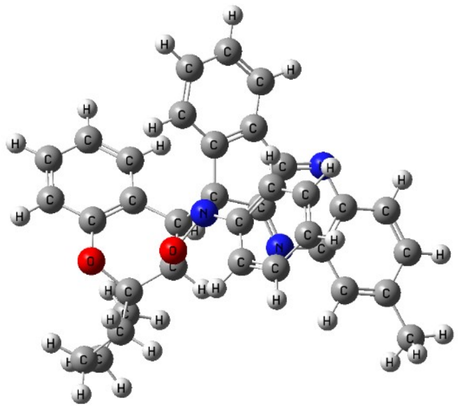

Atom	Coordinates (Angstrom) X	Y	Z
C	-0.991157	-2.752701	-0.464126
C	0.164242	-2.261480	0.174262
C	-1.862428	-1.623081	-0.790020

C	-1.232330	-0.419382	-0.326181
C	0.164415	-0.735137	0.233947
N	-3.033668	-1.641117	-1.382744
C	-3.631312	-0.413934	-1.512592
C	-3.004535	0.789209	-1.046240
N	-1.762181	0.764692	-0.452937
C	-4.901948	-0.320381	-2.125341
C	-5.520357	0.903992	-2.263425
C	-4.913476	2.101726	-1.802751
C	-3.666718	2.024979	-1.203799
N	0.462596	-0.244408	1.604947
O	1.378875	0.854802	1.516365
C	1.372929	1.319052	0.165817
C	1.221447	0.036223	-0.666853
C	-0.581521	0.009744	2.531574
C	-0.632989	1.196912	3.276563
C	-1.636574	1.381659	4.226921
C	-2.599600	0.399745	4.456189
C	-2.537432	-0.787872	3.724862
C	-1.536204	-0.992968	2.779426
C	2.680560	2.063386	-0.065979

O	3.792723	1.162146	0.143888
C	3.753526	-0.033474	-0.514697
C	2.554813	-0.626998	-0.948567
C	2.928307	3.230949	0.900297
C	4.002116	4.063628	0.185841
C	3.559167	4.039627	-1.293845
C	2.774758	2.708555	-1.476720
C	4.987845	-0.649035	-0.759359
C	5.043601	-1.839036	-1.475081
C	3.869832	-2.408618	-1.976971
C	2.646102	-1.799057	-1.714789
C	1.103419	-3.143725	0.697850
C	0.895363	-4.516817	0.537265
C	-0.237400	-5.002301	-0.130345
C	-1.197350	-4.123251	-0.630959
C	-5.617403	3.429370	-1.961182
H	-5.366195	-1.236631	-2.475405
H	-6.497875	0.960233	-2.734958
H	-3.163194	2.913848	-0.834253
H	0.516541	1.983217	-0.007860
H	0.730496	0.258006	-1.619204

H	0.114148	1.960202	3.108322
H	-1.661321	2.309846	4.791535
H	-3.378237	0.551033	5.197314
H	-3.268317	-1.573219	3.896509
H	-1.485112	-1.936200	2.248578
H	2.000823	3.808475	1.001718
H	3.215735	2.869647	1.889665
H	4.973471	3.572438	0.300209
H	4.092677	5.077964	0.585566
H	2.901808	4.890569	-1.503327
H	4.406123	4.117860	-1.981241
H	3.267034	2.019541	-2.167706
H	1.772164	2.897389	-1.875672
H	5.885291	-0.161404	-0.393210
H	6.004907	-2.307737	-1.664700
H	3.907453	-3.319142	-2.566493
H	1.735758	-2.243713	-2.104746
H	1.973758	-2.769090	1.224812
H	1.621653	-5.217586	0.938078
H	-0.375545	-6.073585	-0.243002
H	-2.095298	-4.482810	-1.122982

H	-6.574388	3.315591	-2.476882
H	-5.815400	3.891835	-0.987766
H	-5.009199	4.136622	-2.535745

Compound **21k**

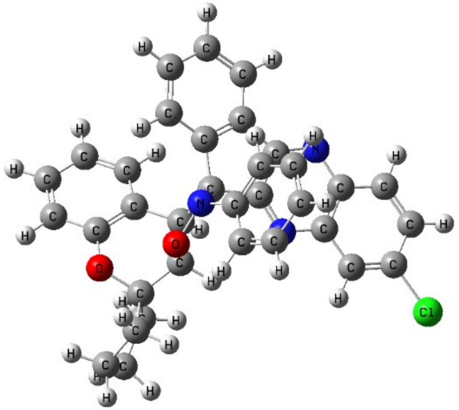

Coordinates (Angstrom)

Atom	X	Y	Z
C	-0.473586	-2.903291	-0.604200
C	0.643715	-2.312657	0.017605
C	-1.497363	-1.877230	-0.798330
C	-0.999074	-0.637670	-0.271613

C	0.453187	-0.808539	0.202043
N	-2.689221	-2.004440	-1.334801
C	-3.442449	-0.860106	-1.340710
C	-2.945795	0.376498	-0.813155
N	-1.681130	0.473284	-0.280708
C	-4.748257	-0.894779	-1.886901
C	-5.531770	0.237865	-1.910884
C	-5.024927	1.448352	-1.384496
C	-3.760679	1.530546	-0.843348
N	0.752990	-0.390969	1.596709
O	1.501348	0.832176	1.562969
C	1.375800	1.388289	0.253968
C	1.356971	0.162582	-0.672559
C	-0.274053	-0.353441	2.578299
C	-0.475027	0.769377	3.393688
C	-1.453637	0.744090	4.386951
C	-2.243159	-0.387090	4.589889
C	-2.030137	-1.509969	3.788081
C	-1.050186	-1.505159	2.798817
C	2.563879	2.317321	0.040998
O	3.791814	1.557615	0.138518

C	3.880106	0.423534	-0.616356
C	2.751462	-0.292426	-1.053924
C	2.697742	3.425525	1.096263
C	3.597752	4.464108	0.412701
C	3.064818	4.504338	-1.034992
C	2.510911	3.078373	-1.314866
C	5.171930	0.002022	-0.956699
C	5.350977	-1.111130	-1.769273
C	4.240615	-1.795712	-2.271826
C	2.960804	-1.379518	-1.915899
C	1.715457	-3.100120	0.424848
C	1.674611	-4.472930	0.164128
C	0.577103	-5.052734	-0.487401
C	-0.513352	-4.273075	-0.871182
Cl	-6.046549	2.876214	-1.430335
H	-5.109194	-1.838408	-2.282111
H	-6.532405	0.215152	-2.327264
H	-3.370311	2.455613	-0.436188
H	0.433714	1.943867	0.161504
H	0.798389	0.387733	-1.586154
H	0.139059	1.647312	3.247418

H	-1.596810	1.625302	5.006359
H	-3.003225	-0.398889	5.364757
H	-2.622684	-2.408076	3.938344
H	-0.876012	-2.399449	2.212138
H	1.705155	3.850919	1.290789
H	3.088788	3.028710	2.035088
H	4.633812	4.110915	0.428189
H	3.571499	5.441895	0.902653
H	2.256078	5.239222	-1.114393
H	3.832755	4.798938	-1.755702
H	3.099536	2.539741	-2.061748
H	1.483277	3.123044	-1.691715
H	6.013050	0.578039	-0.585336
H	6.355790	-1.428465	-2.032478
H	4.370457	-2.645300	-2.934601
H	2.099983	-1.912623	-2.307443
H	2.558761	-2.653841	0.939662
H	2.505456	-5.099952	0.473401
H	0.570498	-6.121561	-0.679114
H	-1.384194	-4.710397	-1.348652

Table S6. LUMO energy, HOMO energy, HOMO-LUMO energy gap, Gibbs free energy, and Gibbs free energy change data of compound **15b**, **15c**, **20c**, **21g** and **21k**.

**Compound**	**E_{LUMO} (Hartree)**	**E_{HOMO} (Hartree)**	**ΔE_g (Hartree)**	**G (Hartree)**	**ΔG (Hartree)**
**15b**	--	--	--	-1086.569485	--
**15c**	--	--	--	-1506.878873	--
**20c**	--	--	--	-578.767802	--
**21g**	-0.07589	-0.20851	0.13262	-2085.661602	-0.019293
**21k**	-0.08489	-0.21296	0.12807	-1665.35658	-0.014927

Fig. S115: Optimized structures, HOMO and LUMO of the synthesized CH_3-substituted and Cl-substituted dispiro-chromeno indenoquinoxaline compounds (**21g** and **21k**).

**Compound**	**Optimized structure**	**LUMO**	**HOMO**
**21g**	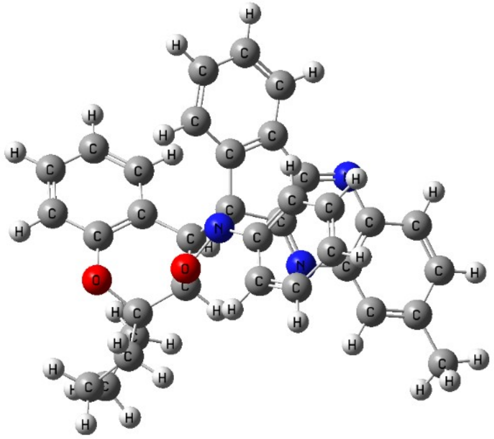	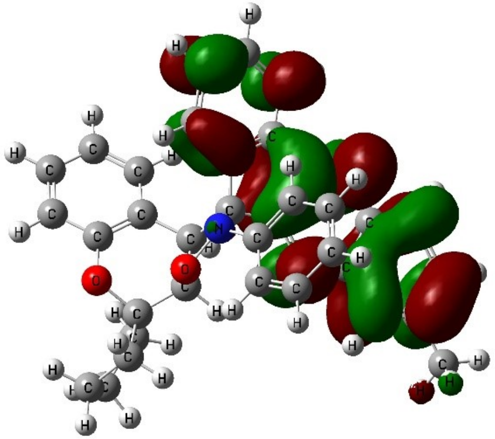	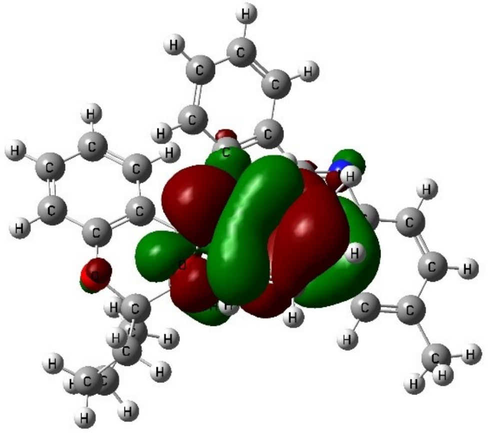

21k

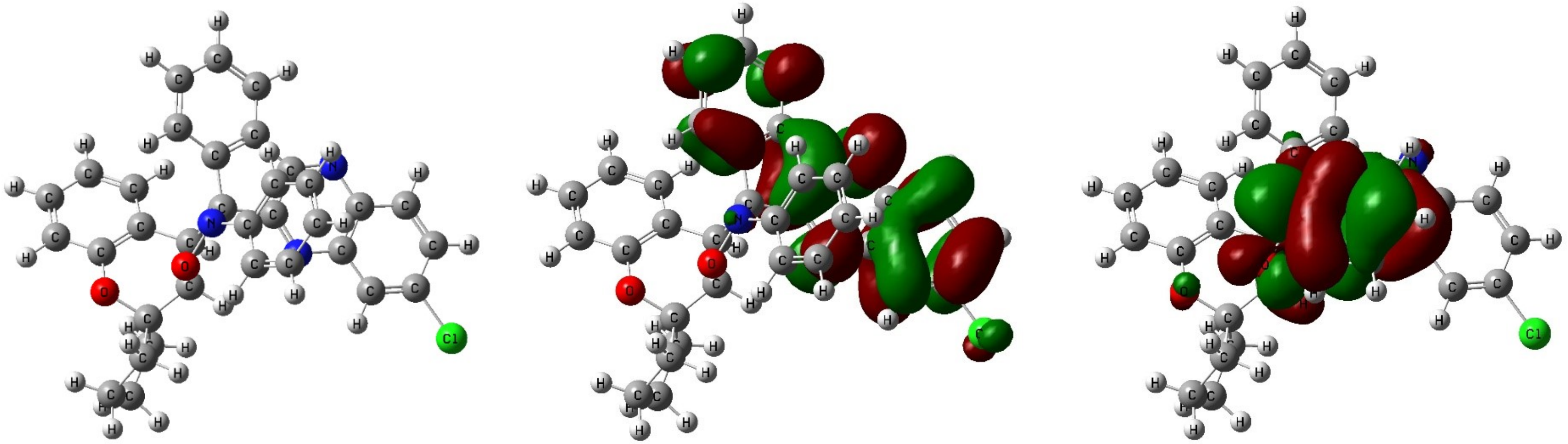

References

1. Frisch, M. J.; Trucks, G. W.; Schlegel, H. B.; Scuseria, G. E.; Robb, M. A.; Cheeseman, J. R.; Scalmani, G.; Barone, V.; Mennucci, B.; Petersson, G. A.; Nakatsuji, H.; Caricato, M.; Li, X.; Hratchian, H. P.; Izmaylov, A. F.; Bloino, J.; Zheng, G.; Sonnenberg, J. L.; Hada, M.; Ehara, M.; Toyota, K.; Fukuda, R.; Hasegawa, J.; Ishida, M.; Nakajima, T.; Honda, Y.; Kitao, O.; Nakai, H.; Vreven, T.; Montgomery, J. A., Jr.; Peralta, J. E.; Ogliaro, F.; Bearpark, M.; Heyd, J. J.; Brothers, E.; Kudin, K. N.; Staroverov, V. N.; Kobayashi, R.; Normand, J.; Raghavachari, K.; Rendell, A.; Burant, J. C.; Iyengar, S. S.; Tomasi, J.; Cossi, M.; Rega, N.; Millam, N. J.; Klene, M.; Knox, J. E.; Cross, J. B.; Bakken, V.; Adamo, C.; Jaramillo, J.; Gomperts, R.; Stratmann, R. E.; Yazyev, O.; Austin, A. J.; Cammi, R.; Pomelli, C.; Ochterski, J. W.; Martin, R. L.; Morokuma, K.; Zakrzewski, V. G.; Voth, G. A.; Salvador, P.; Dannenberg, J. J.; Dapprich, S.; Daniels, A. D.; Farkas, Ö.; Foresman, J. B.; Ortiz, J. V.; Cioslowski, J.; Fox, D. J. Gaussian 09W, Revision A.02. Gaussian, Inc., Wallingford CT, 2009.

2. R. Dennington, T.A. Keith, J.M. Millam, GaussView, Version 6 Semichem Inc, Shawnee Mission, KS, 2016.

3. Rajaraman, D., Anthony, L. A., Sundararajan, G., Shanmugam, M., & Arunkumar, A. (2022). Synthesis, NMR, anti-oxidant, anti-cancer activity, Molecular docking, DFT Calculations and in silico ADME analysis of 3′-benzoyl-4′-phenyl-5′-(piperazin-1-ylmethyl) spiro [indoline-3, 2′-pyrrolidin]-2-one derivatives. Journal of Molecular Structure, 1267, 133551. https://doi.org/10.1016/j.molstruc.2022.133551.

4. Chouchène, N., Toumi, A., Boudriga, S., Edziri, H., Sobeh, M., Abdelfattah, M.A., Askri, M., Knorr, M., Strohmann, C., Brieger, L. and Soldera, A. (2022). Antimicrobial activity and DFT studies of a novel set of spiropyrrolidines tethered with thiochroman-4-one/chroman-4-one scaffolds. Molecules, 27(3), 582. https://doi.org/10.3390/molecules27030582.

5. P. Geerlings, F. De Proft, W. Langenaeker, Conceptual density functional theory, Chem. reviews 103(5) (2003) 1793-1874. https://doi.org/10.1021/cr990029p.

6. K. Periyasamy, P. Sakthivel, G. Venkatesh, V. Palanisamy, Y.S. Mary, Synthesis and design of carbazolebased organic sensitizers for DSSCs applications: experimental and theoretical approaches, Chem. Pap. 78 (2024) 447-461, https://doi.org/10.1007/s11696-023-03101-x.

7. M. Brinzei, A. Stefaniu, O. Iulian, O. Ciocirlan, Density functional theory (DFT) and thermodynamics calculations of amino acids with polar uncharged side chains, Chem. Proc. 3 (1) (2020) 56, https://doi.org/10.3390/ecsoc-24-08420.

8. B.S. Panda, M.A. Ahemad, S. Mohapatra, E. Naik, S. Nayak, S. Mohapatra, P.K. Naik, D. Bhattacharya, C.R. Sahoo, M.K. Sahoo, Microwave-assisted click synthesis, characterisation, and *In silico* studies of novel 2H-chromene-1,2,3-triazolyl glycoconjugates as potent anticancer and antibacterial agents, J. Mol. Struct. 1318 (2024) 139323, https://doi.org/10.1016/j.molstruc.2024.139323.

9. B.S. Panda, B. Samanta, E. Naik, S. Nayak, P. Pragyandipta, S. Mohapatra, P.K. Naik, Design, synthesis, characterization and biological evaluation of 2H-chromene based [1, 2-c] pyrazolone derivatives as promising anticancer agents: In silico computational studies. J. Mol. Struct., 2025, 1347, 143205; DOI: https://doi.org/10.1016/j.molstruc.2025.143205
